# Effect of the [Fe(salen)]_2_‑μ-oxo Catalyst Electronic Structure on Reductive Hydroamination

**DOI:** 10.1021/acs.inorgchem.5c05628

**Published:** 2025-12-20

**Authors:** Emily Pocock, Nathan J. Buxton, Martin Diefenbach, Andrew D. Bond, Simon E. Lewis, Vera Krewald, Ruth L. Webster

**Affiliations:** † Department of Chemistry, 1555 University of Bath, Claverton Down, Bath BA2 7AY, U.K.; ‡ Yusuf Hamied Department of Chemistry, 2152 University of Cambridge, Cambridge CB2 1EW, U.K.; § Department of Chemistry, TU Darmstadt, Peter-Grünberg-Str. 4, Darmstadt 64287, Germany

## Abstract

Salen ligands are privileged scaffolds in transition metal catalysis due to their electronic tunability and capacity to stabilize diverse oxidation states. Herein, we report the synthesis and comparative study of three electronically differentiated [Fe­(salen)]_2_(μ-oxo) complexes and their application in catalytic reductive hydroamination (HA) of nitroarenes with alkenes. A mechanistic framework involving iron-hydride intermediates and hydrogen atom transfer (HAT) was developed, revealing that modulation of the salen ligand electronics significantly impacts product distribution and catalytic efficiency. Systematic investigation of substrate LUMO energies and precatalyst UV–vis spectroscopy, cyclic voltammetry, along with DFT calculations on the key HAT step, was undertaken. Notably, the complex bearing *para*-CF_3_ substituents outperformed its analogues across a range of olefin partners. These findings underscore the critical role of ligand electronics in tuning HAT-based catalysis.

## Graphical abstract

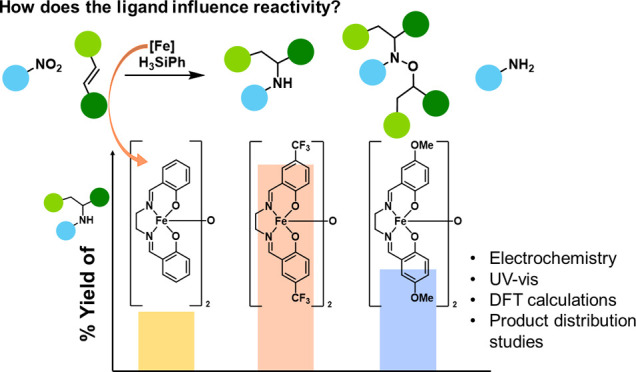

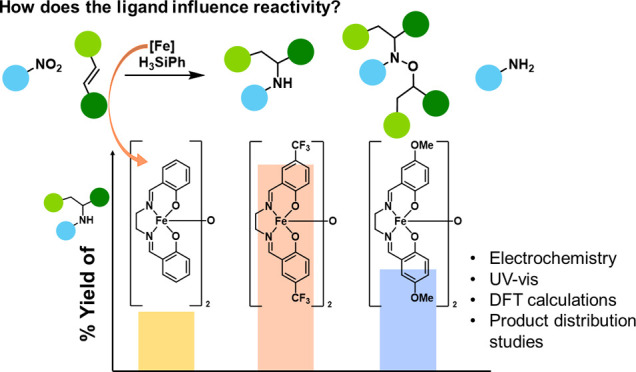

## 1 Introduction

Salen complexes are ubiquitous in a wide range of catalytic transformations. This is largely due to the ability of the pro-ligand to form complexes with a large number of transition metals and to stabilize a range of oxidation states.^1,2^ Salen ligands are also inexpensive and easy to synthesize, and their sterics and electronics are readily tuned. These often-subtle changes to the ligand have, in many cases, been reported to bear a significant effect on reaction outcome.^3,4^

A principal example of a salen ligand being key to reactivity can be seen in the use of Mn­(salen) complexes in enantioselective epoxidations.^5^ During Jacobsen’s studies into ligand effects, steric bulk in the *ortho*-position of the phenyl ring was crucial to obtain high enantioselectivities (Scheme 1a).^5,6^ Alongside this, electron-donating substituents in the *para*-position also led to increased asymmetric induction, with the opposite being true for electron-withdrawing substituents. Other key examples come from Darensbourg and co-workers, who investigated how variations to the readily available (salen)­CrX complex (where X = Cl or N_3_) could influence the rate of CO_2_/cyclohexene oxide copolymer production;^7^ the use of a modified salen complex containing electron-donating groups on the phenoxy fragment led to an increased rate of copolymerization of 65.6 h^–1^ using *para*-OMe from 35.5 h^–1^ using *para*-*^t^* Bu.^7,8^ This report indicates the impact on the metal center (and thus overall reactivity) that can occur from simple changes to ligand electronics (Scheme 1b).^7,9^ Shaver, Storr, and co-workers explored a series of Co­(II) salen complexes with varied *para*-ring substituents for the reversible-termination organometallic-mediated radical polymerization of styrene, methyl methacrylate, and vinyl acetate. The change of electronics in this position has a significant influence on the electron density at the metal center, suggesting that cobalt–carbon bond strength varies with the ligand substitution.^2^ More recently, Shenvi and co-workers reported cyclization and alkene retrocycloisomerization using a Co­(salen) complex (Co­(salen^*t*^Bu,^*t*^Bu)­Cl). The mechanism is believed to be a radical isomerization via a reversible HAT.^10^ When investigating the efficiency of cyclodimerization, the group reported that reaction efficiency seems proportional to the persistence of an intermediate carbon-centered radical.^10^ To explore this further, a variety of Co­(salen) complexes were synthesized with varying electronics on the ligand (Scheme 1c). Electron-deficient ligands led to poor conversion and a low ratio of cycloisomerization; electronically neutral ligands showed moderate selectivity for cyclization, while the use of an electron-rich ligand significantly increased the desired cyclization reaction. These observations were consistent with those reported for the equivalent changes in reversible HAT and metal–carbon radical collapse in polymerizations mediated by Co­(salen) complexes. Electron-deficient complexes appear to terminate the carbon-centered radical, while these radicals are stabilized by electron-rich complexes.^2,10^

**Scheme 1:**
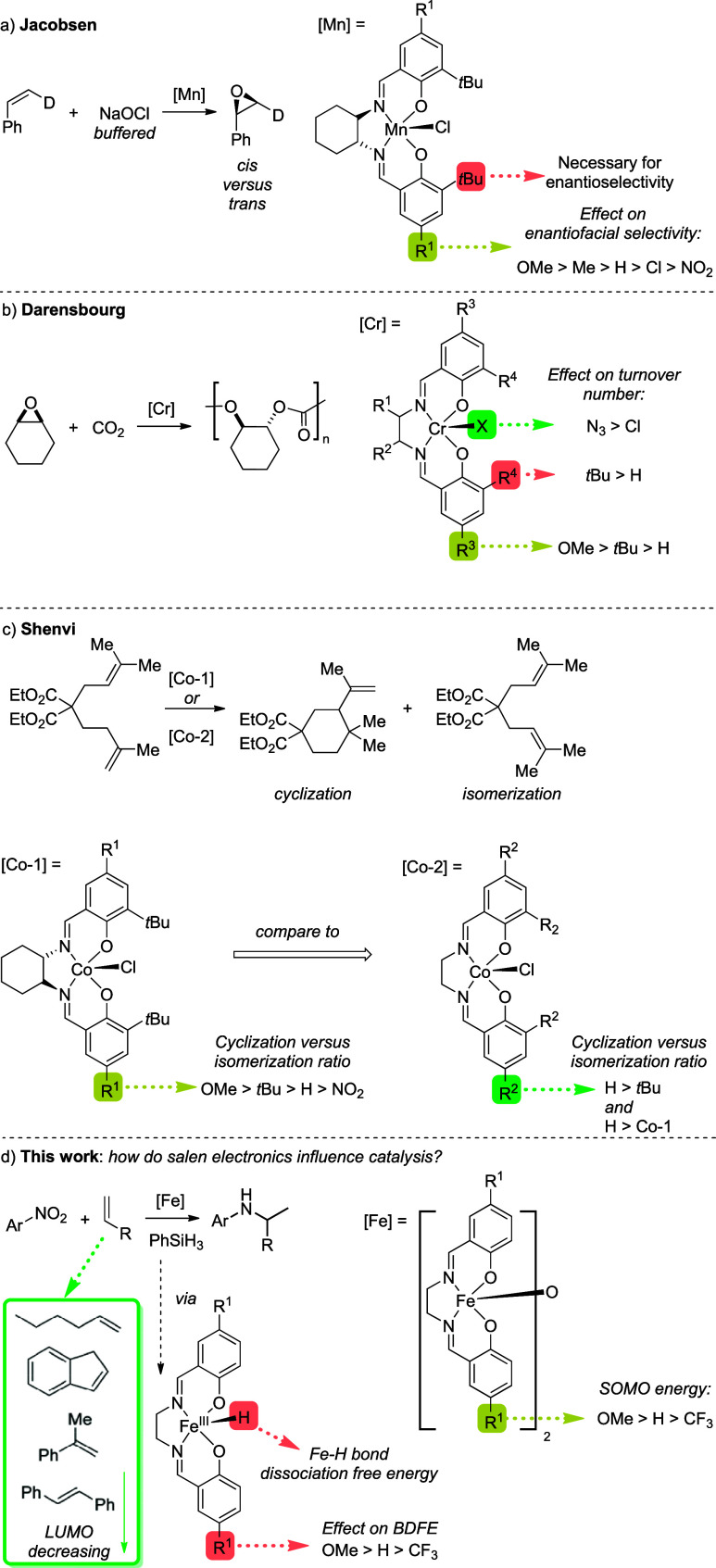
a) Jacobsen’s Seminal Work on Enantioselective Epoxidation Showed That Salen Electronics Influenced Selectivity; (b) Darensbourg’s Variation of Sterics and Electronics Led to Vast Changes in Turnover Number (TON) and Turnover Frequency; (c) Co-Salen Catalysts Synthesized by Shenvi and Co-Workers for the Investigation of How Ligand Electronics Affects Isomerization; (d) In This Work, We Show That Changes to Electronics Affect SOMO Energy and Bond Dissociation Free Energy (BDFE) of a Postulated Intermediate, with a Range of Outcomes Observed Based on the Electronics of the Olefin Coupling Partner

Our own studies on [Fe­(salen)]_2_-μ-oxo complexes have focused on the simplest ligand system. The resultant precatalyst (**1a**, see Schemes 1d and 2) is highly active in hydrophosphination, hydroboration, and alkyne and phosphaalkyne cyclotrimerization.^11–15^ It is important to note that steric bulk in the *ortho*-position, for our catalytic reactions, often results in very low rates of reaction.^14^ More recently, we extended this set of transformations to include the reduction of nitro-compounds.^16^ An extensive mechanistic study was conducted, which revealed the reaction is likely to proceed via a nitroso intermediate and the generation of an on-cycle iron-hydride (**2a**, Scheme 2). Observations made in the seminal work reported by Baran and co-workers,^17^ along with there being similar intermediates investigated in analogous studies with iron precatalysts,^18–27^ meant that we were also able to extend our catalyst system to reductive hydroamination (HA) reactions using nitro compounds and alkenes. Using a nitro compound in HA allows us to bypass a separate reduction step to form an amine (cf. classical HA methodology) and, in theory, allows the use of substrates containing both nitro and amine functionality without functionalization of the latter. However, reductive HA of nitro compounds comes with a wide variety of challenges based on the postulated mechanism: the nitro-compound must be partially reduced to a reactive nitroso intermediate and then trapped by the alkyl radical; the alkyl radical is formed by the same iron hydride species via a HAT reaction. Therefore, the relative rates of both of these transformations should be controlled to selectively generate HA product (**A**, Scheme 2) or an alkyl-hydroxylamine species (**B**) while avoiding over-reduction to aniline (**C**) and other undesired radical reactions such as radical (alkene) polymerization.

**Scheme 2:**
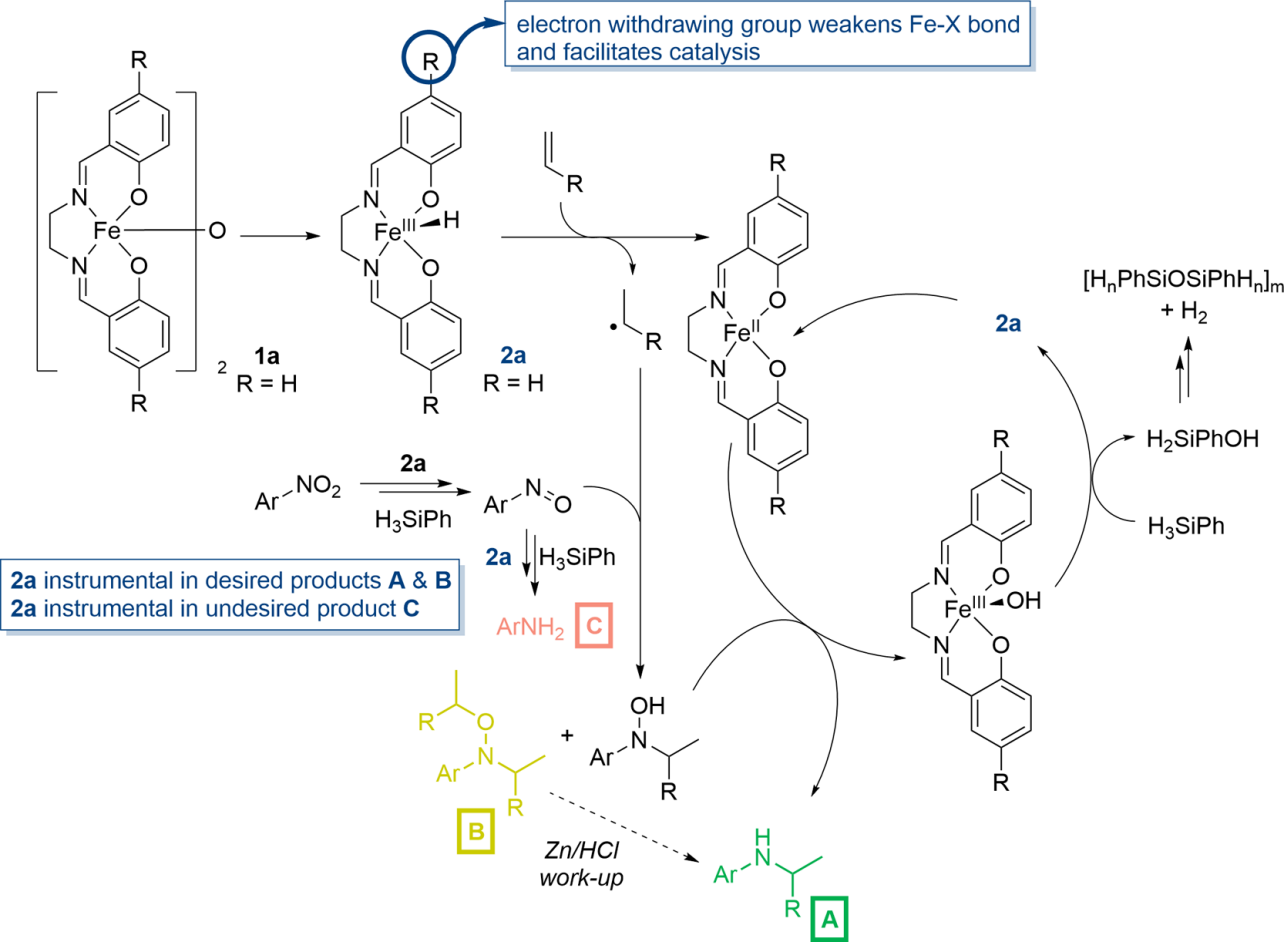
Simplified Hypothetical Catalytic Cycle for the [Fe­(salen)]_2_-μ-oxo Catalyzed HA Reaction, Which Is the Basis of the Experimental and Theoretical Studies Described Herein

In our original study,^16^ we linked HA reactivity to the magnitude of the alkene’s lowest unoccupied molecular orbital (LUMO) energy. Little to no HA reactivity was observed when alkenes with a positive LUMO eigenvalue were employed in the reaction. For example, 1-hexene did not undergo the desired HA reaction, and the reduction to aniline **C** dominated, indicating the energy gap between the alkene LUMO and the Fe–H SOMO (which is the alkyl radical forming reaction) is too large (Table 1, entry 1). In contrast, when indene was employed, the HA reaction dominated, generating a large amount of desired product **A** (entry 2). This is also true for α-methylstyrene, where a large amount of product **B** was observed, which is indicative of the HA reaction taking place (entry 3). However, when the LUMO energy becomes significantly more negative, such as when *trans*-stilbene was employed, a loss of selectivity for the HA reaction was observed (entry 4). We therefore envisioned that the HA reaction could be further explored by varying the ligand’s electronics to modulate the key HAT step and therefore change the product distribution.

**Table 1:** Summary of LUMO Energy and HA Product Distribution

entry	alkene	A (%)	B (%)	C (%)	LUMO (eV)
1	1-hexene	5	0	87	+0.21
2	Indene	77	0	0	–0.91
3	α-methylstyrene	0	83	0	–1.16
4	*trans*-stilbene	37	14	44	–1.84

## 2 Results and Discussion

### 2.1 Synthesis and Analysis of Precatalysts

Three [Fe­(salen)]_2_-μ-oxo complexes (**1a** to **1c**, Figure 1) were synthesized in good yields by varying the salicylaldehyde used in the ligand formation step.^14^

**Figure 1:**
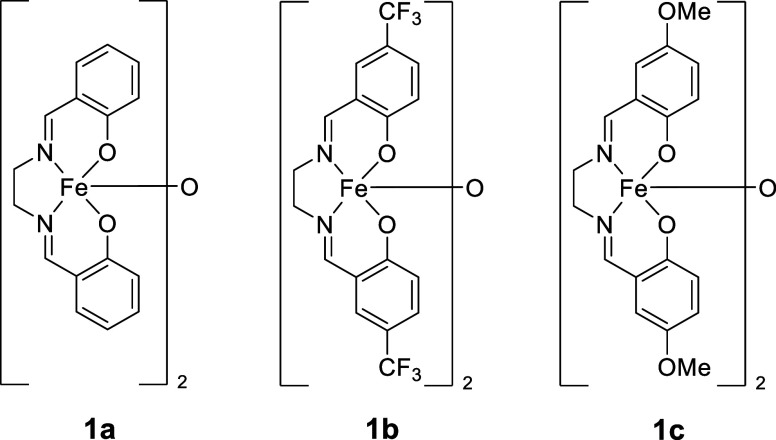
Fe­(salen) complexes synthesized to investigate how varied ligand electronics could affect product distribution in reductive HA.

We initiated studies by probing the precatalysts to see if we could make a link between the electronic nature of **1a** to **1c** and the outcomes of HA. Using UV–visible spectroscopy, the band of greatest interest is the ligand-to-metal charge transfer (LMCT) band observed between 400 and 530 nm.^28–30^ Altering the functional groups in the *para*-position of the phenolate units (complexes **1a**, **1b**, and **1c**) results in a significant change of the energy of the LMCT bands (Table 2 and the Supporting Information). Complex **1c** has a band within the in-plane LMCT region present at 524 nm; thus, it has the lowest energy gap for the LMCT transition. **1b** proved challenging to analyze using UV–visible spectroscopy, but there may be a high-frequency band below 400 nm (tentatively assigned at 346 nm), which would be consistent with a significantly larger SOMO-LUMO gap. Consistent with these data, **1a** has an intermediate absorption band at 480 nm.

**Table 2:** UV–Visible Spectroscopy and Cyclic Voltammetry Data for Complexes **1a** to **1c**

complex	λ_max_ (nm)	E_1/2_ (V vs Fc^+^/Fc)
**1a**	480	–0.95
**1b**	346	–1.23
**1c**	524	–0.98

Cyclic voltammetry studies reveal that **1b** has the lowest formal electrode potential at −1.23 V, while **1a** (with no donating/withdrawing groups) has a formal electrode potential of −0.95 V, and **1c** (with *para*-OMe groups) is similar at −0.98 V (Table 2 and the Supporting Information). CF_3_ groups alter the formal electrode potential such that **1b** is the most prone to oxidation, and accessing an on-cycle Fe­(II) species may occur less readily, **1a** and **1c** are very similar, and they may behave similarly in catalysis. The complexes all appear to show reversible redox cycles, which are important for the HA reaction, which is believed to proceed via an Fe­(III)/(II) cycle. The calculated Nicholson parameters indicate that the redox processes are reversible, as their values are approximately equal to 1 (1.14–1.33).^31^ This is also consistent with the theory that there is no significant geometry change around the metal center with any change in oxidation state.^32^ At this stage, we were ready to investigate whether trends in the electronic nature of these precatalysts would be borne out in discernible trends in reductive HA catalysis.

### 2.2 Study of Precatalysts 1a to 1c in Reductive HA

The four olefins, 1-hexene, indene, α-methylstyrene, and *trans*-stilbene, with LUMO energies ranging from 0.21 to −1.84 eV, were employed in the HA reaction using our previously optimized conditions. The challenges associated with in situ analysis of these reaction mixtures mean that isolated yields are reported. It is also worth noting that there is very little unreacted nitro starting material observed in any of the reactions studied.

Indene was initially investigated as the olefin coupling partner because it was the most selective for the desired HA product when precatalyst **1a** was used (**3**, Table 3). In comparison, **1b** is less selective, with modest amounts of a double HA product forming (**5**, 14%) in addition to 72% **3**. **1c** shifts reactivity away from the formation of **3** (46%), and the indenyl-hydroxylamine forms in modest amounts (**4**, 14%) along with trace amounts of a new double HA product, **5**, and aniline **6** (from the undesired over reduction of the nitro starting material).

**Table 3:**
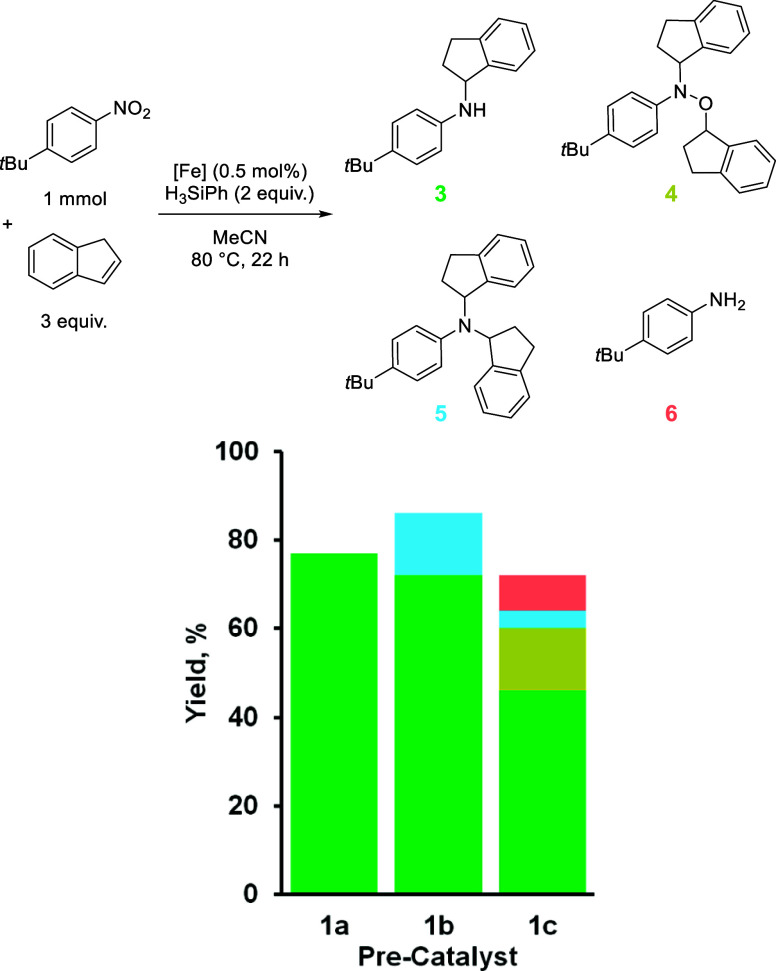
Product Distribution from the Reaction of Indene and *tert*-Butyl-4-nitrobenzene Using Precatalysts **1a** to **1c**

	1a	1b	1c
Combined Yield, %	77	86	72
**3**, %	77	72	46
**4**, %	0	0	14
**5**, %	0	14	4
**6**, %	0	0	8

The formation of compound **5** is unexpected. To investigate this, a test reaction was conducted where HA product **3** was subjected to the standard reaction conditions using catalyst **1b** (Scheme 3). Monitoring the reaction by thin-layer chromatography and ^1^H NMR spectroscopy shows no further reaction of **3**, indicating that this is not the route to formation of product **5**.

**Scheme 3:**
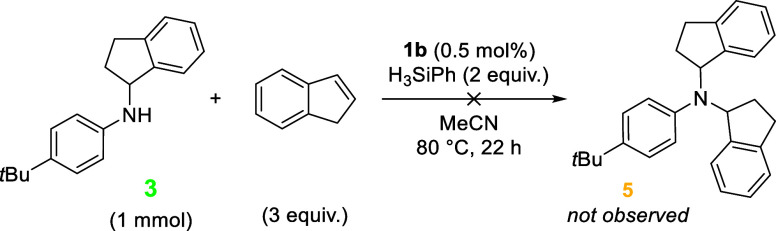
Attempted Reaction of **3** under Standard Reaction Conditions Using Precatalyst **1b**

Based on the proposed HA mechanism (Scheme 2), **5** could be forming from a side reaction of a hydroxylamine intermediate (Scheme 4). The hydroxylamine intermediate reacts with Fe^II^(salen) to form an aminyl radical. While these are reportedly short-lived species, in this specific case the radical is likely to be resonance stabilized due to the presence of the aromatic ring.^33^ The resultant aminyl radical could then undergo a termination process by combining with the corresponding indenyl radical that has formed during the HAT step.

**Scheme 4:**
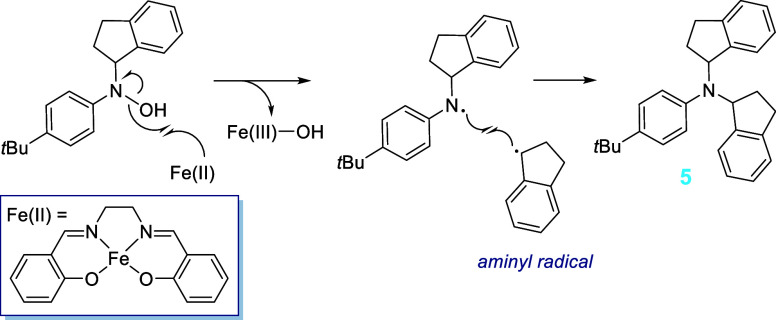
Proposed Radical Mechanism for the Formation of Product **5**

Indene is a good substrate for this chemistry; we therefore turned our attention to less reactive HA substrates. α-Methylstyrene was investigated next because it is selective for the formation of alkyl-hydroxylamine **8** using **1a**, and we questioned how precatalyst electronics could affect reaction selectivity (Table 4). In the case of **1b** and **1c**, the yield of **8** is reduced, and moderate amounts of the desired HA product are obtained (21% and 14% from **1b** and **1c**, respectively). As with **1a**, aniline **6** is not formed.

**Table 4:**
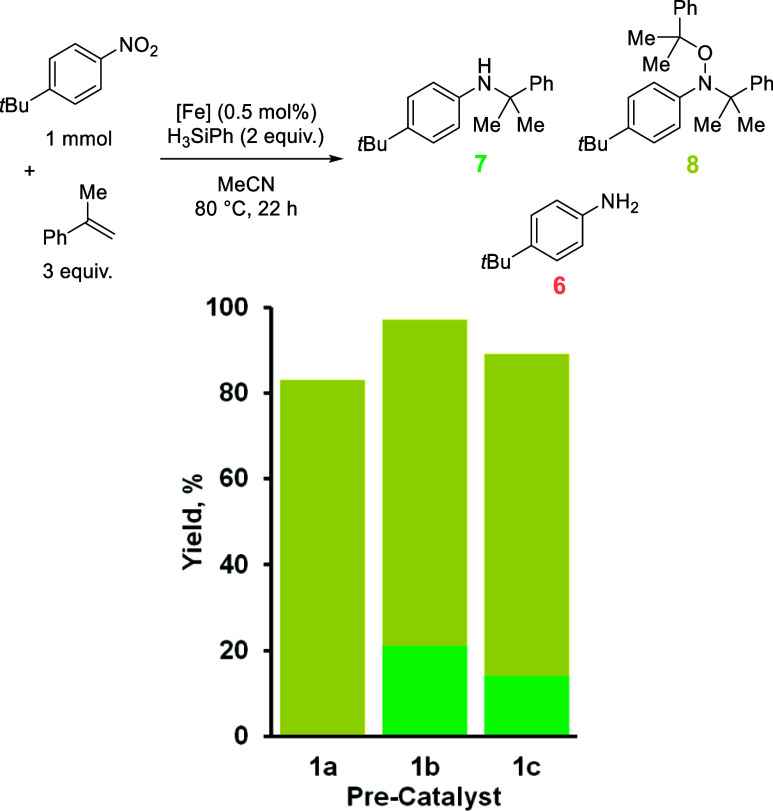
Product Distribution from the Reaction of α-Methylstyrene and *tert*-Butyl-4-nitrobenzene Using Precatalysts **1a** to **1c**

	1a	1b	1c
combined yield, %	83	97	89
**7**, %	0	21	14
**8**, %	83	76	75
**6**, %	0	0	0

Trace amounts of **7′** are also obtained using precatalyst **1c** (Scheme 5). **7′** could form when the alkyl radical reacts with a second equivalent of α-methylstyrene. In turn, this resultant radical species could react with the nitroso radical, which can then re-enter the standard catalytic cycle. This is likely to be a disfavored process due to the longevity of the alkyl radicals and the reactive nature of the nitroso-intermediate, which provides an explanation as to why **7′** has only ever been isolated in trace amounts, and in this case only.^34^ This result also highlights the propensity for oligomerization during this reaction and hence the reduction in the combined isolated yields reported.

**Scheme 5:**
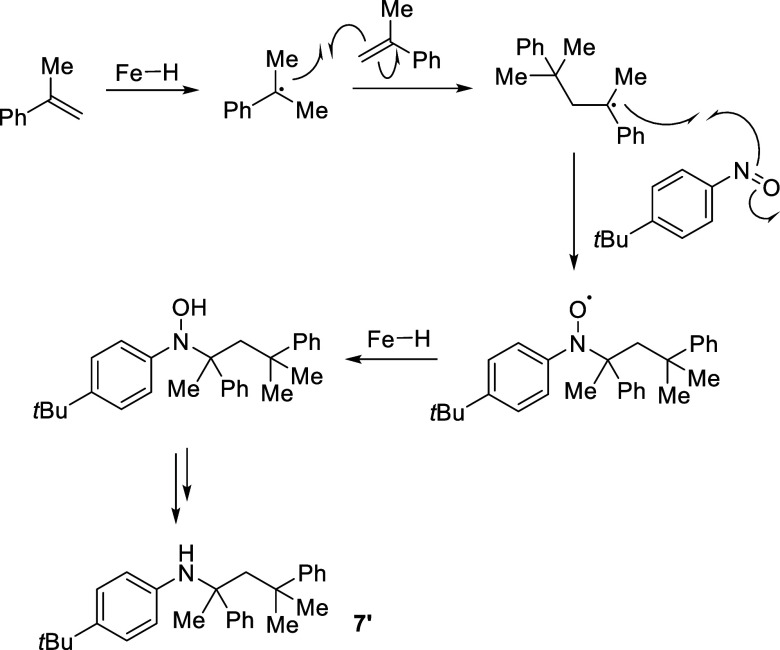
Proposed Radical Mechanism for the Formation of Product **7′**

The reaction of 1-hexene with **1a** was unselective for HA, with a large amount of aniline **6** being generated. However, using **1b**, the yield of **9** increases to 33% (compared to 5% using **1a**, Table 5). The increase in the yield of **9** occurs concomitantly with a decrease in the yield of **6** (87% using **1a**, 42% using **1b**). Although the yield of **9** using **1b** could be described as a moderate yield, this is a greater than 6-fold increase in yield compared to our previous report. **1c** generates only 58% **6**, 12% **9** (doubling the yield compared to **1a**), and 8% of the azo compound **11**. The reaction is clearly finely balanced, with different reaction pathways being switched on based on the change in catalyst. However, compounds **1a** and **1c** behave fairly similarly in terms of the quantity of compound **9** formed.

**Table 5:**
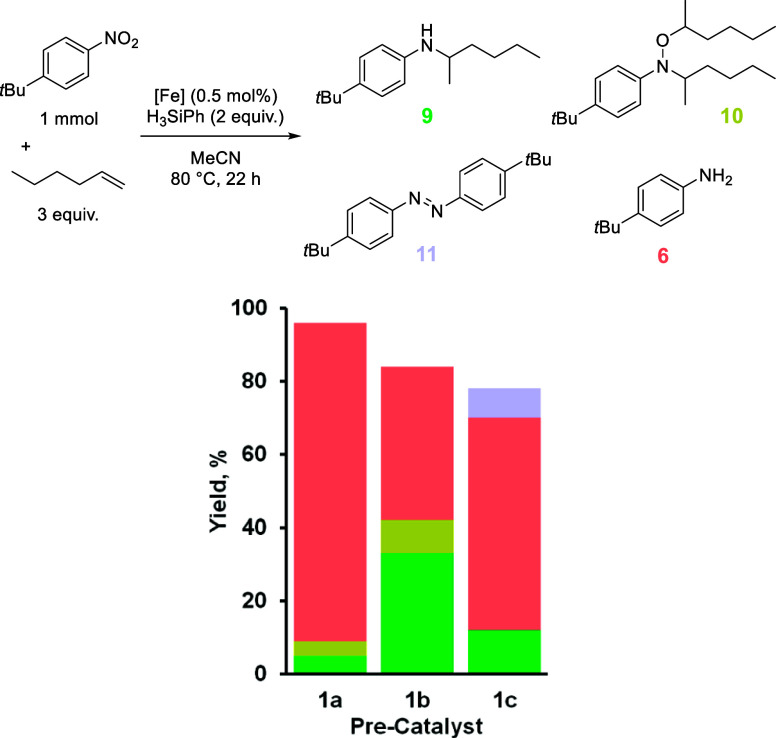
Product Distribution from the Reaction of 1-Hexene and *tert*-Butyl-4-nitrobenzene Using Precatalysts **1a** to **1c**

	1a	1b	1c
combined yield, %	96	84	78
**9**, %	5	33	12
**10**, %	4	9	0
**6**, %	87	42	58
**11**, %	0	0	8

Finally, the HA reaction was examined by using *trans*-stilbene as the olefin coupling partner. When the reaction was conducted using precatalyst **1a**, we noted that this reaction has the widest product distribution, highlighting a system that might be highly susceptible to catalyst-driven shifts (Table 6). The yield of **12** ostensibly doubles using **1b** (71% using **1b** versus 37% using **1a**), while the formation of **6** is reduced by two-thirds (15% using **1b** versus 44% using **1a**). No alkylhydroxylamine **13** is obtained using **1b**. Employing **1c** seems to limit the formation of **13** (2% **13** and 48% **12**), but the formation of **6** is a dominant reaction pathway (32%).

**Table 6:**
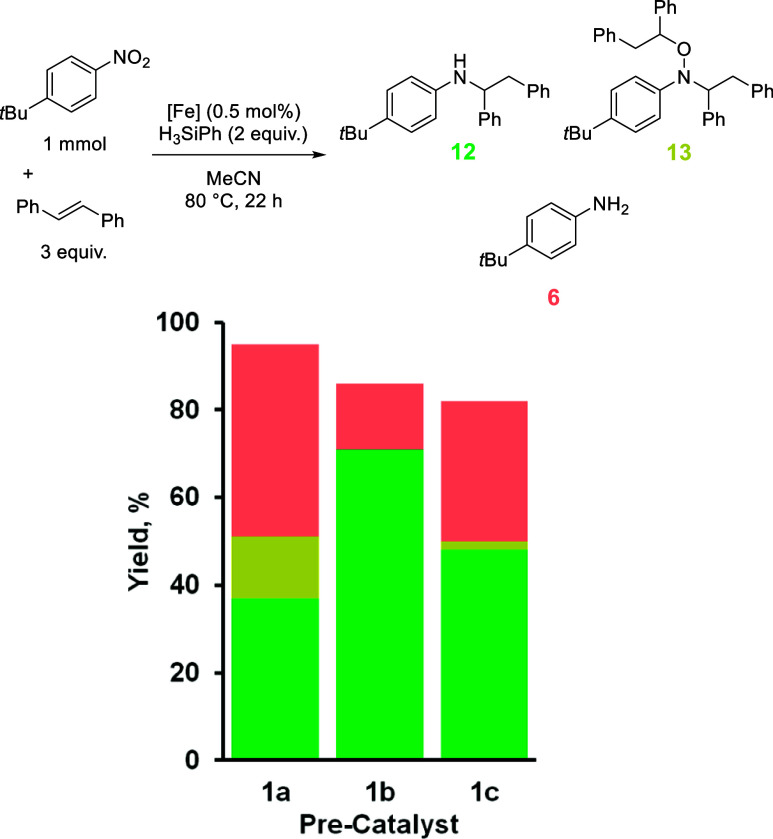
Product Distribution from the Reaction of *trans*-Stilbene and *tert*-Butyl-4-nitrobenzene Using Precatalysts **1a** to **1c**

	1a	1b	1c
combined yield, %	95	86	82
**12**, %	37	71	48
**13**, %	14	0	2
**6**, %	44	15	32

To summarize the findings thus far: precatalyst **1b** is generally a better catalyst than **1a** and **1c**, often generating more of the desired HA products and minimizing the reduction to **6**.

### 2.3 Computational Investigation into the Reaction Results

We next turned to simple density functional theory (DFT) calculations to determine whether there were trends in HA reactivity and catalyst energetics. Although operationally simple, our initial studies showed that a simple comparison of the SOMO energies of **2a** to **2c** (Figure 2) would provide an inadequate link to the synthetic data (see the Supporting Information for these calculations).

**Figure 2:**
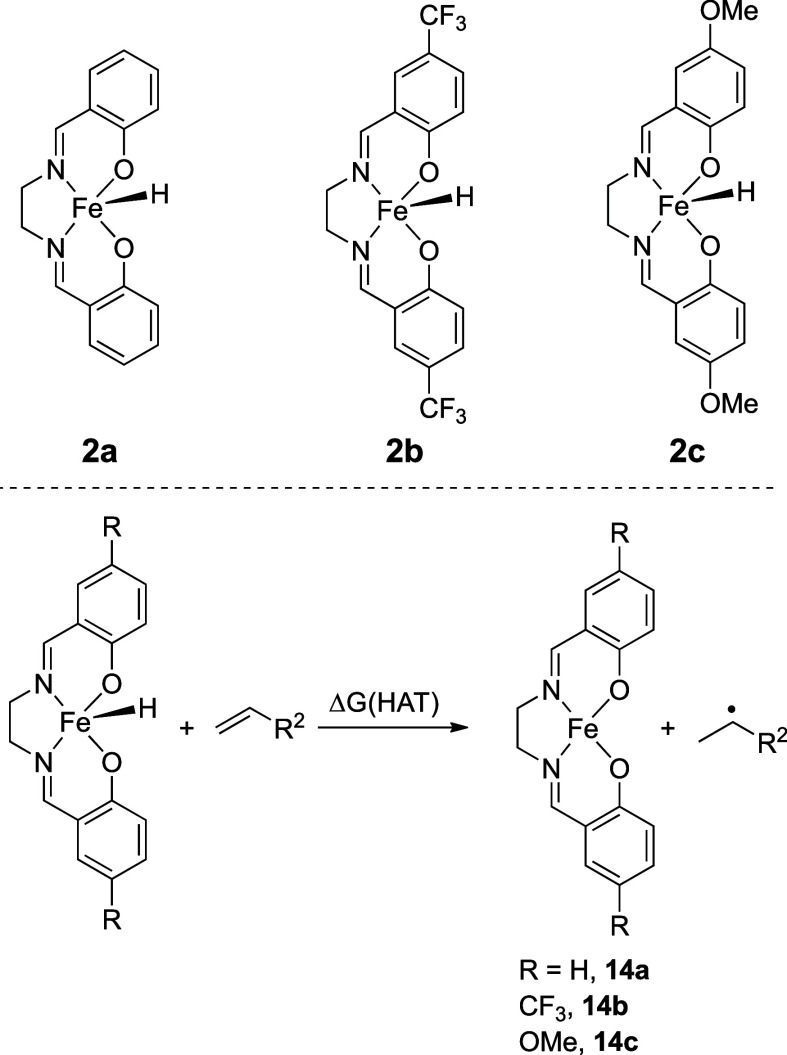
Structures of the Fe­(II) hydrides (**2a** to **2c**) formed from the activation of precatalysts **1a** to **1c** (top) and the isodesmic reaction enthalpies for complexes **2** reacting with alkene via a HAT process to generate an alkyl radical and the Fe­(II) complexes **14a** to **14c** (bottom).

Thus, a more exacting approach was needed, and we calculated the isodesmic HAT values for the reaction of the iron hydride (**2a** to **2c**) with the alkene (Figure 2). First, the different potential spin states were calculated for complexes **2** and **14** to determine the ground state among the three spin states, i.e., low-spin doublet (LS), intermediate-spin quartet (IS), and high-spin sextet (HS). From our previous studies, **2a** was found to have an HS ground state at the DFT level PBE0-D3/def2-TZVP//PBE-D3/def2-SVP, which was benchmarked against HF-DLPNO-CCSD­(T). The HS state is also the lowest in energy for **2b** and **2c** (Table 7) using PBE0-D3/def2-TZVP//PBE-D3/def2-SVP. The spin states of the corresponding Fe^II^(salen) complexes were determined (**14a** to **14c**, Table 8) at the same level, which also confirmed the HS configuration as the ground state.

**Table 7:** Spin Expectation Values and Relative Energies for **2a** to **2c** on the HS, IS and LS Surfaces Computed at the PBE0-D3/def2-TZVP//PBE-D3/def2-SVP Level

spin state	⟨*S* ^2^⟩	*H*_rel_(kcal mol^–1^)	*G*_rel_(kcal mol^–1^)
**2a**			
HS	8.762	0.00	0.00
IS	3.819	7.11	8.13
LS	0.801	1.90	5.15
**2b**			
HS	8.761	0.00	0.00
IS	3.869	6.25	9.16
LS	0.8	1	5.65
**2c**			
HS	8.763	0.00	0.00
IS	3.814	7.74	9.49
LS	0.802	0.49	4.05

**Table 8:** Spin Expectation Values and Relative Energies for Complexes **14a** to **14c** on the HS, IS, and LS Surfaces Computed at the PBE0-D3/def2-TZVP//PBE-D3/def2-SVP Level

spin state	⟨*S* ^2^⟩	*H*_rel_(kcal mol^–1^)	*G*_rel_(kcal mol^–1^)
**14a**			
HS	6.033	0	0
IS	2.035	6.41	7.67
LS	0	44.12	46.39
**14b**			
HS	6.032	0	0
IS	2.035	6.66	7.94
LS	0	45.8	47.68
**14c**			
HS	6.036	0	0
IS	2.039	6.48	7.75
LS	0	40.92	43.53

A plot of the reaction enthalpies versus the combined yield of HA product and hydroxylamine shows an excellent trend where the combined yield increases with increasing HAT reaction enthalpies (see Figure 3).^35^

**Figure 3:**
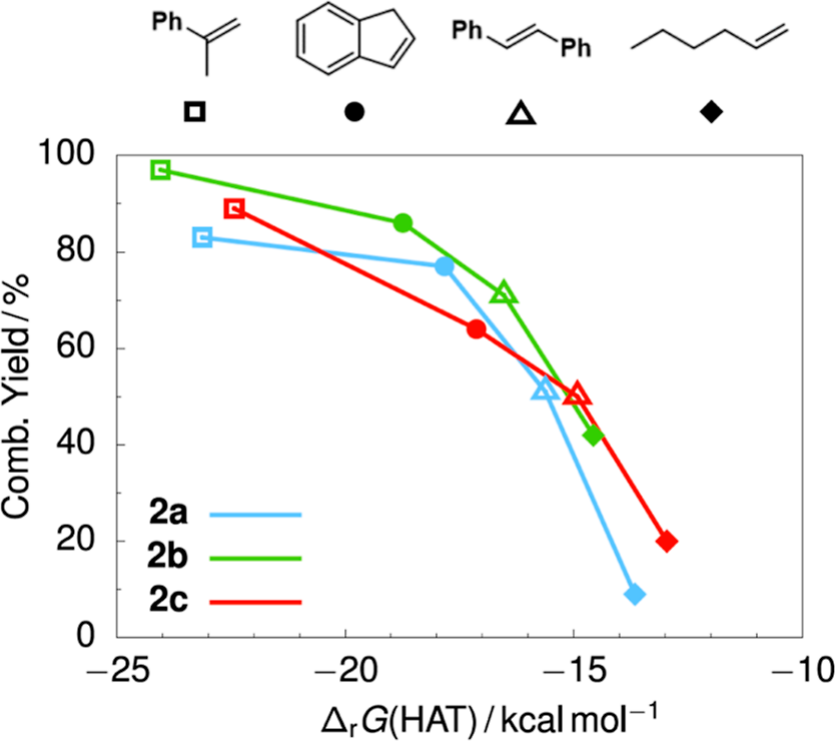
Plot of reaction enthalpy for Fe–H + olefin → Fe­(salen) + alkyl radical versus combined yield of the HA product + hydroxylamine product.

## 3 Reaction of Stilbenes

Finally, based on the improvement in yield for the HA reaction using *trans*-stilbene, different stilbene derivatives were investigated in the reaction using catalyst **1b** (Figure 4).

**Figure 4:**
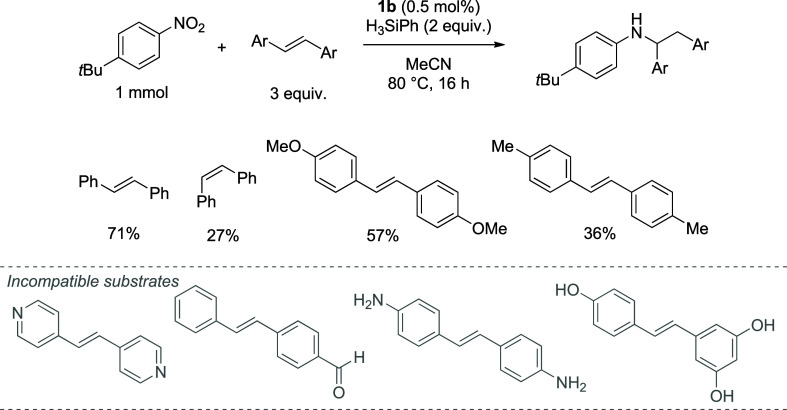
Reaction scope for the HA reaction of *trans*-stilbene derivatives using **1b** as the precatalyst.

Stilbene derivatives have HA yields varying from 71% to 27%. Unfortunately, the optimized catalytic conditions could not be easily transferred to the *trans*-stilbene derivatives, with many exhibiting poor solubility under the reaction conditions, e.g., free amines or alcohols, which were highly insoluble at elevated temperatures and in multiple solvents. The reaction with a pyridine-based stilbene gave only unreacted nitro (90% recovered). In the case of the aldehyde functionalized derivative, although initially soluble, precipitation occurred after a few hours at the reaction temperature. This solid could be a result of reductive amination occurring between the aniline and the aldehyde.

## 4 Methods

### 4.1 Precatalyst Synthesis

Fe­(OAc)_2_ (1 equiv) was weighed into a round-bottom flask and dissolved in EtOH, resulting in a dark brown solution. To this, a solution of proligand (1.2 equiv, see the Supporting Information for methods of synthesis) in EtOH was added, yielding a red solution. The resulting red solution was stirred at 80 °C for 2 h. The flask was then allowed to cool to room temperature before the resulting precatalyst (**1a**–**1c**) was isolated via vacuum filtration.

### 4.2 Electrochemistry Procedure

Electrochemical measurements were performed in a 0.2 M solution of tetrabutylammonium hexafluoride with a 0.2 mM concentration of complexes **1a** to **1c** and a 0.2 mM standard of ferrocene in acetonitrile. A three-electrode cell was used for the electrochemical experiments employing a separate working, counter, and reference electrode. Cyclic voltammograms were cycled three or four times to obtain steady-state conditions. The working electrode was a polished glassy carbon disc (3 mm diameter) electrode. The electrode was polished and cleaned between each separate experiment. A platinum (Pt(0)) wire was used as the counter electrode. A silver (Ag(0)) wire was used as the reference electrode. The reference and counter electrodes were cleaned with isopropanol and flamed between each experiment. All electrochemical measurements were run under an inert nitrogen atmosphere, where the solutions were degassed by sparging with nitrogen for 15 min. Between measurements, the solutions were stirred briefly to renew the layer of solution near the surface of the electrodes.

### 4.3 Synthetic Procedure for Reductive Hydroamination

A mixture of precatalyst (0.005 mmol) and *p*-*tert*-butylnitrobenzene (1.0 mmol, 1 equiv) in dry acetonitrile (0.5 mL) was added to an ampule with a magnetic stirrer bar under nitrogen. Olefin (3 mmol, 3 equiv) was added to the solution, followed by phenyl silane (2 mmol, 2 equiv). The reaction mixture was then stirred at 80 °C under a positive flow of nitrogen for 22 h. After the reaction had gone to completion, the solvents were removed under vacuum. The resulting crude product was then purified by flash column chromatography on silica using hexane/dichloromethane.

### 4.4 Computational Methods

Molecular geometries were optimized at the DFT level employing the generalized gradient approximation (GGA) via the PBE^36^ functional in conjunction with the D3 atom-pairwise dispersion correction without damping^37^ and an implicit polarizable continuum solvent model^38^ utilizing acetonitrile as the solvent. The split-valence double-ζ def2-SVP basis set^39^ was used together with the corresponding auxiliary Coulomb-fitting basis set of Weigend.^40^ At this GGA DFT level, abbreviated as PBE-D3­(PCM)/def2-SVP, frequency calculations were performed on the optimized stationary points to characterize minima and transition structures and to extract thermal contributions to enthalpies and Gibbs energies at 298.15 K. For improved relative energies, single-point energy calculations were performed at the hybrid DFT level with 25% admixture of Fock exchange via the PBE0 functional^41,42^ employing the triple-ζ valence polarized def2-TZVP basis set^39^ and the same dispersion and solvent corrections as above. The final relative Gibbs energies and orbital eigenvalues are thus reported at the PBE0-D3­(PCM)/def2-TZVP//PBE-D3­(PCM)/def2-SVP level.

## 5 Summary

Investigations showed that variations in the electronics of a salen ligand could result in a shift in product distribution for an iron-catalyzed HA reaction. The precatalysts were examined using UV–vis spectroscopy and cyclic voltammetry, and this showed that *para*-OMe gave a more reducible metal center and higher energy SOMO. A *para*-CF_3_ group had the opposite effect on the electronics at iron.

Each new precatalyst was examined in the HA reactions using *tert*-butyl nitrobenzene and the olefin coupling partners 1-hexene, indene, α-methylstyrene, and *trans*-stilbene. These HA reactions led to the discovery that the presence of an electron-withdrawing group (precatalyst **1b**) improved the selectivity for HA reactions compared to the standard unsubstituted system.

To further explore the trends observed in reactivity, computational analysis was performed on the various iron hydride species to determine both the SOMO value and the BDFE of the intermediate iron hydrides. While these results cannot fully explain all changes in the reactivity profiles that were observed, they do act as a guide to help understand the difference in the reactivity of these new catalysts. Based on the complexity of this reaction, where there are three interlinked catalytic cycles (one being the unproductive nitroso to aniline process), it is clear that a deeper study of the mechanism, including kinetics of the individual cycles, is needed to develop an efficient set of catalysts that span a range of alkene substrates. This work is ongoing in our laboratories.

## Supplementary Material



## Notes

Additional experimental details, analysis data, NMR spectra, and computational methods (PDF)

## References

[1] Cozzolino M., Leo V., Tedesco C., Mazzeo M., Lamberti M. (2018). Salen, salan and salalen
iron­(III) complexes as catalysts for CO/epoxide reactions and ROP
of cyclic esters. Dalton Trans..

[2] Chiang L., Allan L. E., Alcantara J., Wang M. C., Storr T., Shaver M. P. (2014). Tuning ligand electronics
and peripheral substitution
on cobalt salen complexes: structure and polymerisation activity. Dalton Trans..

[3] Whiteoak C. J., Salassa G., Kleij A. W. (2012). Recent advances with π-conjugated
salen systems. Chem. Soc. Rev..

[4] Kleij A. W. (2009). Nonsymmetrical
Salen Ligands and Their Complexes: Synthesis and Applications. Eur. J. Inorg. Chem..

[5] Jacobsen E. N., Zhang W., Muci A. R., Ecker J. R., Deng L. (1991). Highly Enantioselective
Epoxidation Catalysts Derived from 1,2-Diaminocyclohexane. J. Am. Chem. Soc..

[6] Palucki M., Finney N. S., Pospisil P. J., Güler M. L., Ishida T., Jacobsen E. N. (1998). The mechanistic basis for electronic
effects on enantioselectivity in the (salen)­Mn­(III)-catalyzed epoxidation
reaction. J. Am. Chem. Soc..

[7] Darensbourg D. J., Mackiewicz R. M., Phelps A. L., Billodeaux D. R. (2004). Copolymerization
of CO_2_ and epoxides catalyzed by metal salen complexes. Acc. Chem. Res..

[8] Darensbourg D. J., Mackiewicz R. M., Rodgers J. L., Fang C. C., Billodeaux D. R., Reibenspies J. H. (2004). Cyclohexene Oxide/CO_2_ Copolymerization Catalyzed
by Chromium­(III) Salen Complexes and *N*-Methylimidazole:
Effects of Varying Salen Ligand Substituents and Relative Cocatalyst
Loading. Inorg. Chem..

[9] Darensbourg D. J., Mackiewicz R. M., Rodgers J. L. (2005). Role of the cocatalyst in the copolymerization
of CO_2_ and cyclohexene oxide utilizing chromium salen complexes. J. Am. Chem. Soc..

[10] Crossley S. W., Barabe F., Shenvi R. A. (2014). Simple, chemoselective, catalytic
olefin isomerization. J. Am. Chem. Soc..

[11] Gallagher K. J., Webster R. L. (2014). Room temperature
hydrophosphination using a simple
iron salen pre-catalyst. Chem. Commun..

[12] Lau S., Provis-Evans C. B., James A. P., Webster R. L. (2021). Hydroboration of
aldehydes, ketones and CO_2_ under mild conditions mediated
by iron­(III) salen complexes. Dalton Trans..

[13] Provis-Evans C. B., Lau S., Krewald V., Webster R. L. (2020). Regioselective Alkyne Cyclotrimerization
with an In Situ-Generated [Fe­(II)­H­(salen)]·Bpin Catalyst. ACS Catal..

[14] Hood T. M., Lau S., Diefenbach M., Firmstone L., Mahon M., Krewald V., Webster R. L. (2023). The Complex
Reactivity of [(salen)­Fe]_2_(μ-O)
with HBpin and Its Implications in Catalysis. ACS Catal..

[15] Espinal-Viguri M., King A. K., Lowe J. P., Mahon M. F., Webster R. L. (2016). Hydrophosphination
of Unactivated Alkenes and Alkynes Using Iron­(II): Catalysis and Mechanistic
Insight. ACS Catal..

[16] Pocock E., Diefenbach M., Hood T. M., Nunn M., Richards E., Krewald V., Webster R. L. (2024). Synthetic and Mechanistic
Studies
into the Reductive Functionalization of Nitro Compounds Catalyzed
by an Iron­(salen) Complex. J. Am. Chem. Soc..

[17] Gui J., Pan C.-M., Jin Y., Qin T., Lo J. C., Lee B. J., Spergel S. H., Mertzman M. E., Pitts W. J., La Cruz T. E., Schmidt M. A., Darvatkar N., Natarajan S. R., Baran P. S. (2015). Practical olefin hydroamination with
nitroarenes. Science.

[18] Crotti C., Cenini S., Rindone B., Tollari S., Demartin F. (1986). Deoxygenation
reactions of *ortho*-nitrostyrenes with carbon monoxide
catalysed by metal carbonyls: a new route to indoles. J. Chem. Soc. Chem. Commun..

[19] Zhu K., Shaver M. P., Thomas S. P. (2016). Chemoselective
nitro reduction and
hydroamination using a single iron catalyst. Chem. Sci..

[20] Shevlin M., Guan X., Driver T. G. (2017). Iron-Catalyzed
Reductive Cyclization
of *o*-Nitrostyrenes Using Phenylsilane as the Terminal
Reductant. ACS Catal..

[21] Song H., Yang Z., Tung C.-H., Wang W. (2020). Iron-Catalyzed
Reductive
Coupling of Nitroarenes with Olefins: Intermediate of Iron–Nitroso
Complex. ACS Catal..

[22] Driver T. G. (2022). Unlocking
Electrophilic *N*-Aryl Intermediates from Aryl Azides,
Nitroarenes, and Aryl Amines in Cyclization–Migration Reactions. Synlett.

[23] Tran C., Abdallah A., Duchemann V., Lefèvre G., Hamze A. (2023). Iron-catalyzed reductive cyclization of nitroarenes: Synthesis of
aza-heterocycles and DFT calculations. Chin.
Chem. Lett..

[24] Vu V., Powell J. N., Ford R. L., Patel P. J., Driver T. G. (2023). Development
and Mechanistic Study of an Iron-Catalyzed Intramolecular Nitroso
Ene Reaction of Nitroarenes. ACS Catal..

[25] Waheed M., Alsharif M. A., Alahmdi M. I., Mukhtar S., Parveen H. (2023). Iron-catalyzed
intramolecular reductive cyclization of *o*-nitroarenes
to indoles under visible light irradiation. Tetrahedron Lett..

[26] Zou D., Wang W., Hu Y., Jia T. (2023). Nitroarenes and nitroalkenes
as potential amino sources for the synthesis of *N*-heterocycles. Org. Biomol. Chem..

[27] Qi S., Yin D., Hu L., Huang C., Xie R., Lu G., Wang J. (2025). Iron-Catalyzed
Reductive Allylic C–H Amination of Olefin with
Nitroarenes via Intermolecular Nitroso Ene Reaction. Angew. Chem., Int. Ed..

[28] Andrea K. A., Brown T. R., Murphy J. N., Jagota D., McKearney D., Kozak C. M., Kerton F. M. (2018). Characterization
of Oxo-Bridged Iron
Amino-bis­(phenolate) Complexes Formed Intentionally or in Situ: Mechanistic
Insight into Epoxide Deoxygenation during the Coupling of CO2 and
Epoxides. Inorg. Chem..

[29] Cozzolino M., Leo V., Tedesco C., Mazzeo M., Lamberti M. (2018). Salen, salan and salalen
iron­(III) complexes as catalysts for CO_2_/epoxide reactions
and ROP of cyclic esters. Dalton Trans..

[30] Brisdon, A. K. ; Brisdon, A. K. Inorganic Spectroscopic Methods. In UV-Visible Spectroscopy; Oxford University Press, 2023.

[31] Nicholson R. S. (1966). Semiempirical
Procedure for Measuring with Stationary Electrode Polarography Rates
of Chemical Reactions Involving the Product of Electron Transfer. Anal. Chem..

[32] Zanello, P. ; Nervi, C. ; Fabrizi de Biani, F. Theory, Practice and Application. In Inorganic Electrochemistry; RSC, 2003.

[33] Shimizu D., Furukawa K., Osuka A. (2017). Stable Subporphyrin *meso*-Aminyl Radicals without Resonance Stabilization by
a Neighboring
Heteroatom. Angew. Chem., Int. Ed..

[34] Clayden, J. ; Greeves, N. ; Warren, S. Radical Reactions in Organic Chemistry; Oxford University Press, 2001; p 970.

[35] The yield of the HA product and alkyl hydroxylamine product were added because the latter is readily reduced using Zn/HCl to generate the HA product.

[36] Perdew J. P., Burke K., Ernzerhof M. (1996). Generalized
Gradient Approximation Made Simple. Phys. Rev.
Lett..

[37] Grimme S., Antony J., Ehrlich S., Krieg H. (2010). A consistent and accurate
ab initio parametrization of density functional dispersion correction
(DFT-D) for the 94 elements H-Pu. J. Chem. Phys..

[38] Tomasi J., Mennucci B., Cammi R. (2005). Quantum Mechanical Continuum Solvation
Models. Chem. Rev..

[39] Weigend F., Ahlrichs R. (2005). Balanced basis sets of split valence, triple zeta valence
and quadruple zeta valence quality for H to Rn: Design and assessment
of accuracy. Phys. Chem. Chem. Phys..

[40] Weigend F. (2006). Accurate Coulomb-fitting
basis sets for H to Rn. Phys. Chem. Chem. Phys..

[41] Perdew J. P., Ernzerhof M., Burke K. (1996). Rationale for mixing exact exchange
with density functional approximations. J. Chem.
Phys..

[42] Adamo C., Barone V. (1999). Toward reliable density
functional methods without
adjustable parameters: The PBE0 model. J. Chem.
Phys..

