# Supplementary material for: Effect of the [Fe(salen)]2‑μ-oxo Catalyst Electronic Structure on Reductive Hydroamination
Source: Inorg Chem. 2025 Dec 20;65(1):999–1007. doi: 10.1021/acs.inorgchem.5c05628 (PMC12801303; doi:10.1021/acs.inorgchem.5c05628)
Supplement: Supplementary file 1 [file ic5c05628_si_001.pdf]

Supporting Information

The Effect of [Fe(salen)]$_2$-μ-oxo Catalyst Electronic Structure on Reductive Hydroamination

Emily Pocock,[a] Nathan J. Buxton,[b‡] Martin Diefenbach,[c] Andrew D. Bond,[b] Simon E. Lewis,[a*] Vera Krewald,[c*] Ruth L. Webster[b*]

[a] Department of Chemistry, University of Bath, Claverton Down, Bath, BA2 7AY. UK
[b] Yusuf Hamied Department of Chemistry, University of Cambridge, Cambridge, CB2 1EW. UK
[c] Department of Chemistry, TU Darmstadt, Peter-Grünberg-Str. 4, 64287 Darmstadt, Germany

Corresponding Author Email:
vera.krewald@tu-darm-stadt.de sl288@bath.ac.uk rw740@cam.ac.uk

Contents

1. General Considerations 3
2. Synthesis of $[Fe(salen)]_2$-μ-oxo Catalyst 4
 2.1. General procedure for the synthesis of ligands 4
 2.2. General Method for the synthesis of Fe-salen Complexes[1] 13
 2.3. Cyclic Voltammetry 20
3. One-Pot Hydroaminations 28
 3.1. General Procedure for the One-Pot Hydroaminations 28
4. Quantum-Chemical Calculations 86
 4.1. General Considerations Computational Methods 86
5. One-Pot Hydroaminations Quantum Chemical Calculations 87
 5.1. Summary of SOMO energies and BDFE trends for catalysts 2a to 2c and 14a to 14c. 87
6. Single Crystal X-Ray Diffraction 107
7. References 110

1. General Considerations

Ligand and pre-catalyst synthesis were performed in air. All other manipulations were carried out using standard Schlenk-line and glovebox techniques under an inert atmosphere of argon (Ar). An MBraun MB200B glovebox was employed operating at <0.1 ppm O_2 and <0.1 ppm H_2O. Acetonitrile was dried over two batches of 3 Å molecular sieves, sparged with Ar and stored under an inert atmosphere. Benzene, toluene, and tetrahydrofuran (THF) were distilled from Na/benzophenone and stored over activated 3 Å molecular sieves. Acetonitirile-d_3 was dried over two batches of 3 Å molecular sieves, sparged with Ar and stored under an inert atmosphere. Benzene-d_6 was degassed and dried over sodium and freeze-pump-thaw degassed. Glassware was dried for 12 hours at 120°C prior to use.

^{1}H, ^{13}C{^{1}H}, ^{19}F{^{1}H} and ^{11}B{^{1}H} NMR spectra were recorded on Bruker Advance, Agilent, or Avance III 500, and 400 MHz NMR spectrometers. In CD_3CN, ^{1}H and ^{13}C{^{1}H} NMR chemical shifts are reported relative to CH_3CN at 1.94 ppm and 118.26 ppm, respectively; in $CDCl_3$, ^{1}H and ^{13}C{^{1}H} NMR chemical shifts are reported relative to $CHCl_3$ at 7.26 ppm and 77.16 ppm, respectively. For the assignment of the ^{1}H and ^{13}C{^{1}H} NMR spectra 2D NMR (COSY, HSQC, HMBC) experiments were also performed. Data were processed in MestReNova. Coupling constants (*J*) are reported in Hertz (Hz). Multiplicities are indicated by: br s (broad singlet), s(singlet), d (doublet), t (triplet), q (quartet) and m (multiplet) app. (apparent).

Infrared (IR) spectra of neat compounds were recorded at ambient temperature over the range 4000–650 cm^{-1} using a PerkinElmer Spectrum 100 ATR-FTIR spectrometer using a diamond ATR unit. Peaks are reported in cm^{-1}.

UV-Vis spectra were recorded on a Mettler Toledo UV5 spectrometer.

For mass spectrometry a microTOF electrospray time-of-flight (ESITOF) mass spectrometer (Bruker Daltonik GmbH, Bremen, Germany) was used. Data are reported in the form of *m/z*. The observed mass and isotope pattern matched the corresponding theoretical values as calculated from the expected molecular formula.

Electrochemical cyclic voltammetry data was obtained using either a EmStat3 Blue or a CompactStat.H.

Analytical thin-layer chromatography was performed on Merck silica gel 60 F254 aluminium-backed plates. Visualisation was accomplished with UV light (254 nm), and vanillin stain. Automated flash column chromatography (normal phase) was performed using a CombiFlash NextGen 300+ system equipped with UV and ELSD detectors, using 4 - 40g silica columns.

Unless otherwise stated, all chemicals were purchased from commercial sources, dried over molecular sieves and used as supplied.

2. Synthesis of $[Fe(salen)]_2$-μ-oxo Catalyst

2.1. General procedure for the synthesis of ligands

To a flask containing aldehyde (3.5 equiv.) in ethanol was added amine (0.31 g, 1 equiv.). The vessel was then heated at reflux for 2 hours. The resulting solution was then cooled to 0 °C and filtered to yield a bright yellow residue. The resultant yellow residue was washed with cold EtOH and dried in vacuo to give the desired product.[1]

2.1.1. N,N'-Bis(salicylidene)ethylenediamine ligand[1]

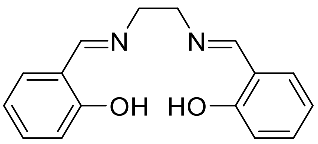

Following the general procedure and using salicylaldehyde (2.17 mL, 18.3 mmol) and ethylenediamine (0.0.35 mL, 5.2 mmol) in EtOH (40 mL). Yield: 1.31 g (94%) bright yellow solid. Data is consistent with literature.[1]

^{1}H NMR ($CDCl_3$, 500 MHz): *δ* 13.19 (s, 2H, Ar-OH), 8.36 (s, 2H, N=CH), 7.31-7.27 (m, 2H, Ar-H), 7.24-7.22 (m, 2H, Ar-H), 6.95-6.93 (m, 2H, Ar-H), 6.87-6.84 (m, 2H, Ar-H), 3.95 (s, 4H, N-CH2-CH2-N). Concordant with literature.

^{13}C NMR ($CDCl_3$, 101 MHz): δ 166.6, 161.1, 132.5, 131.6, 118.9, 117.1, 59.9.

HRMS (ESI+): calcd for $[M, C_{16}H_{17}N_2O_2]^+$ 269.1285, found 269.1294

FT-IR (cm^{-1}): 2901.7, 1610.6, 1576.5, 1495.5, 1281.5, 1149.5 cm^{-1}

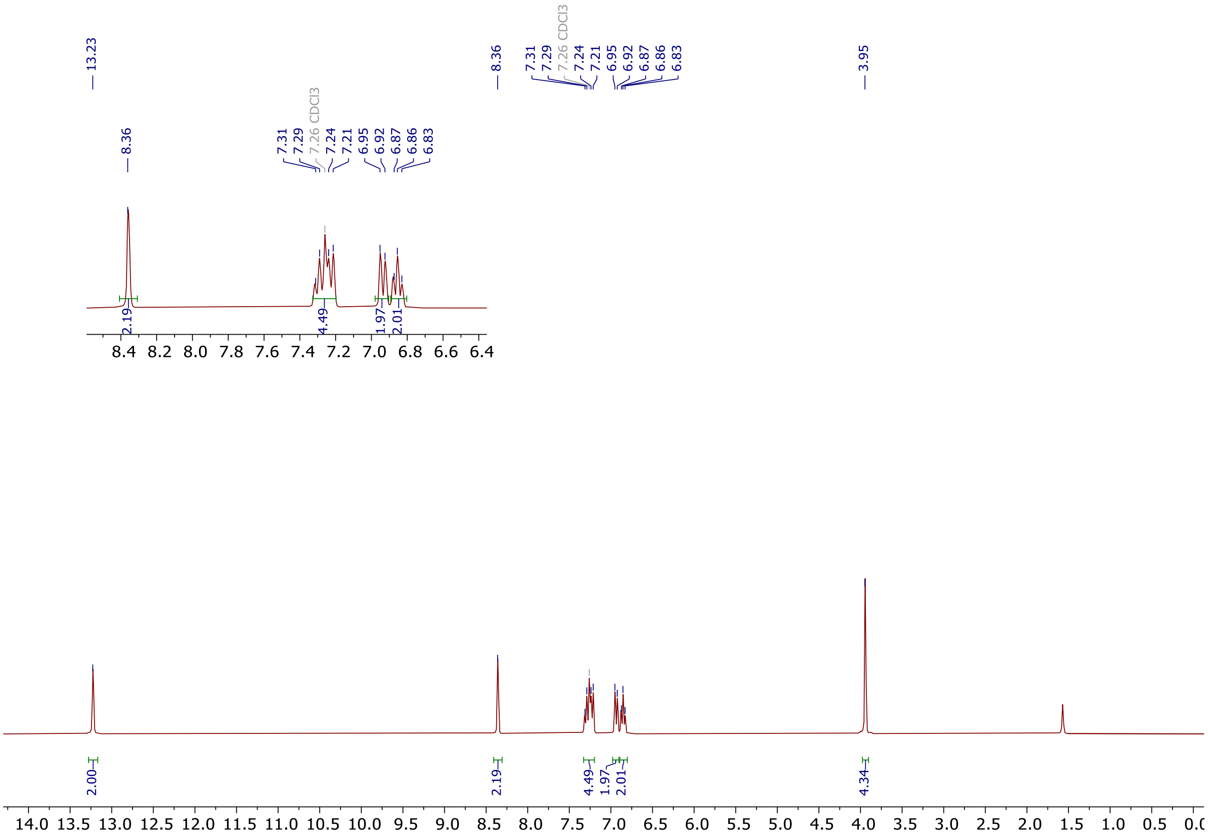

Figure S1: ^{1}H NMR Spectrum of *N,N'-Bis(salicylidene)ethylenediamine* in $CDCl_3$.

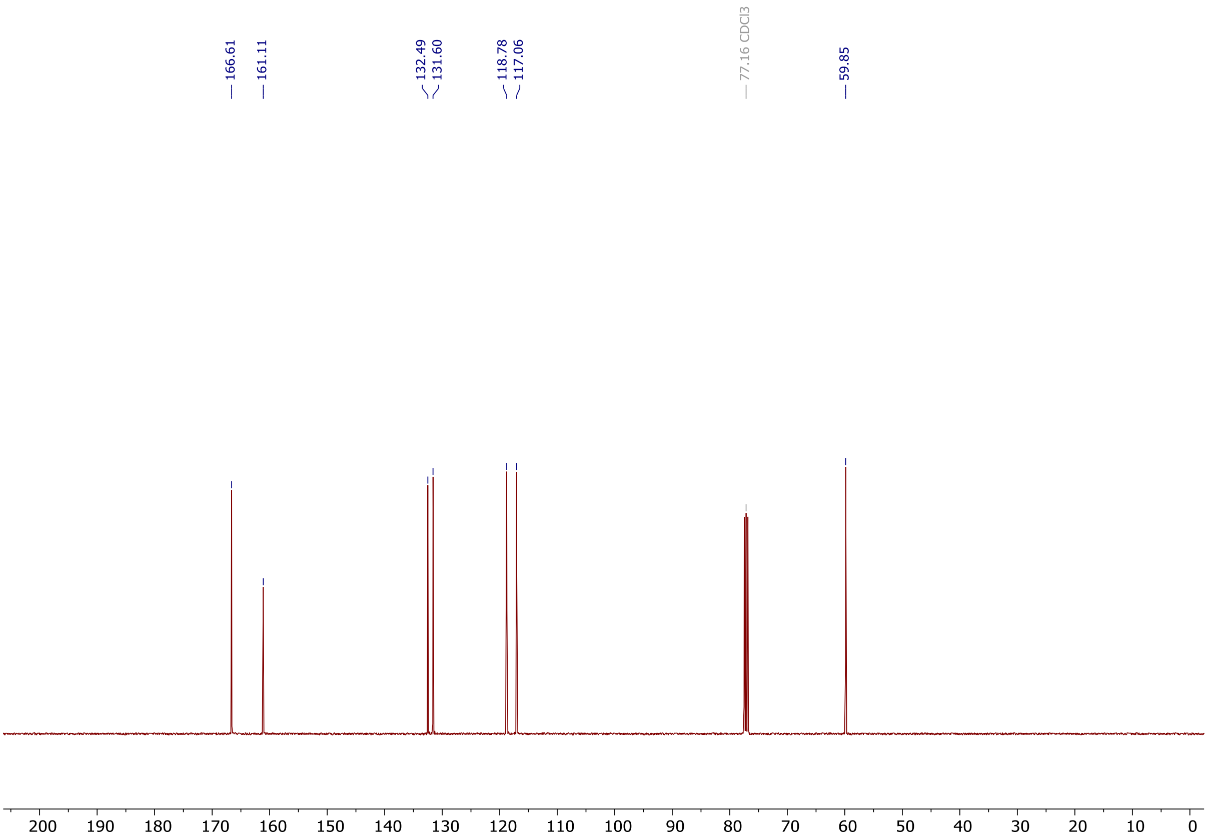

Figure S2: ^{13}C NMR Spectrum of *N,N'-Bis(salicylidene)ethylenediamine* in $CDCl_3$.

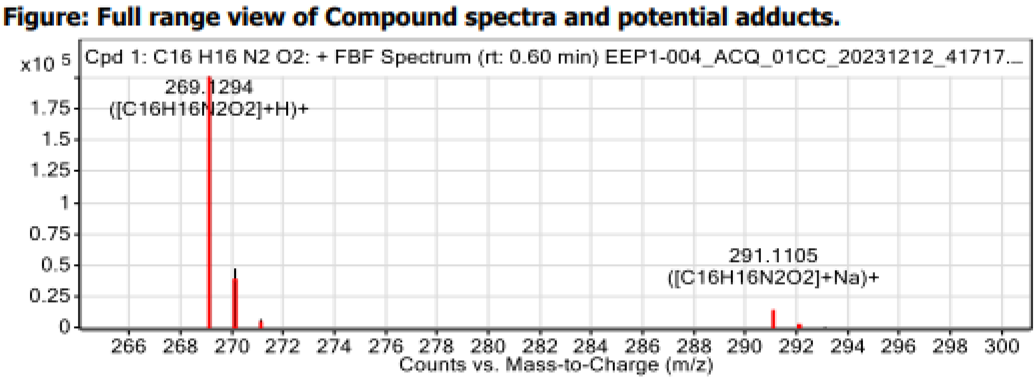

Figure S3: HRMS spectra for *N,N'-Bis(salicylidene)ethylenediamine.*

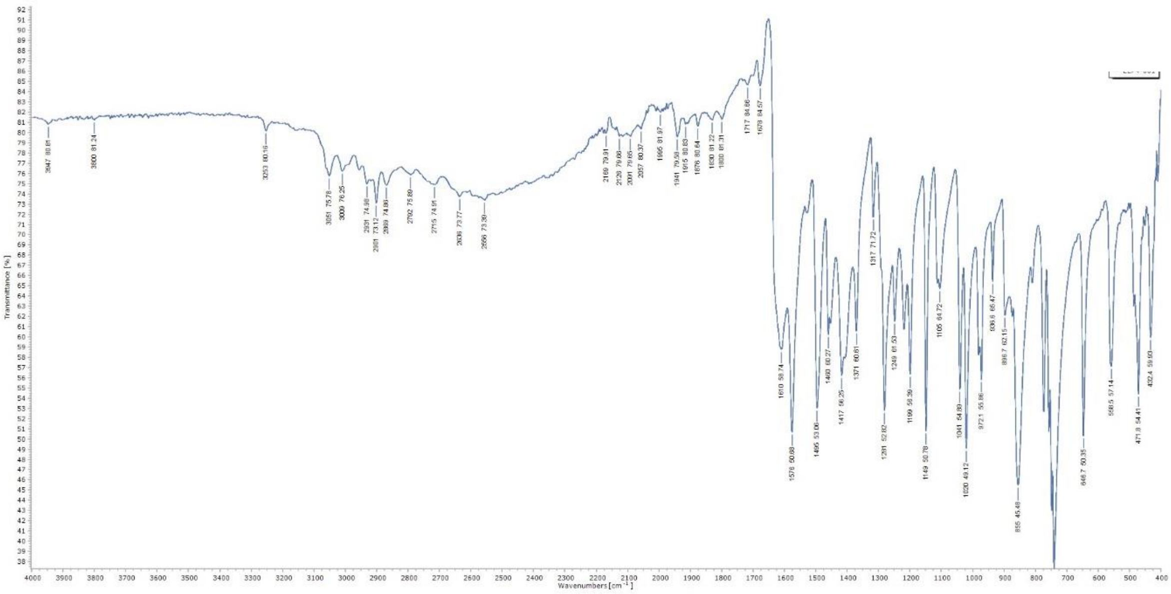

Figure S4: IR spectra for *N,N'-Bis(salicylidene)ethylenediamine.*

2.1.2. 2,2'-((1E,1'E)-(ethane-1,2-diylbis(azaneylylidene))bis(methaneylylidene))bis(4-(trifluoromethyl)phenol)

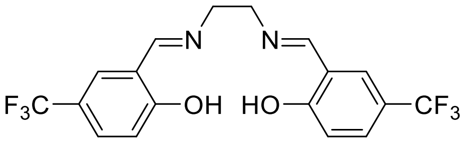

Following the general procedure and using 2-hydroxy-5-trifluoromethylbenzaldehyde (1.20 g, 5.8 mmol) and ethylenediamine (0.11 mL, 1.66 mmol) in EtOH (20 mL). Yield: 0.65 g (96%) bright yellow solid. Data is consistent with literature.[2]

^{1}H NMR ($CDCl_3$, 500 MHz): *δ* 13.6 (s, 2H, Ar-OH), 8.41(s, 2H, N=CH), 7.55 – 7.51 (m, 4H, Ar-H), 7.03 (d, J = 8.5 Hz, 2H, Ar-H), 4.00 (s, 4H, N-CH2-CH2-N).

^{13}C NMR ($CDCl_3$, 120 MHz): δ 165.92, 163.85, 129.48 (q, *J* = 3.4 Hz), 128.93 (q, *J* = 3.9 Hz), 124.24 (q, *J* = 271.0 Hz), 121.30 (q, *J* = 33.4 Hz), 118.10, 117.94, 59.62.

^{19}F NMR (CD_3CN, 471 MHz): *δ* -62.02.

HRMS (ESI+): calcd for [M, $C_{18}H_{15}F_6N_2O_2$]$^+$ 405.1032, found 405.1033.

FT-IR (cm^{-1}): 2909.8, 1626.2, 1519.5, 1316.8, 1154.8, 899.3 cm^{-1}

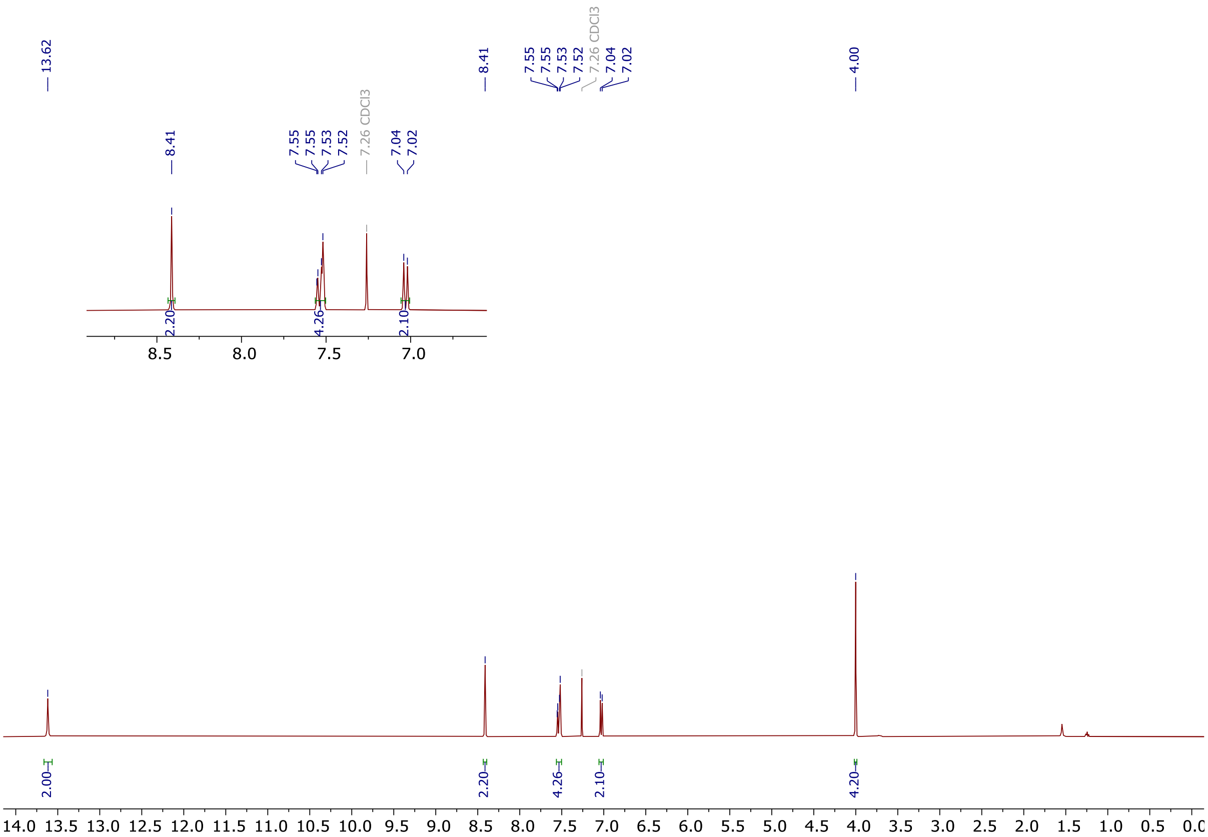

Figure S5: ^{1}H NMR Spectrum of *2,2'-((1E,1'E)-(ethane-1,2-diylbis(azaneylylidene))bis(methaneylylidene))bis(4-(trifluoromethyl)phenol)* in $CDCl_3$.

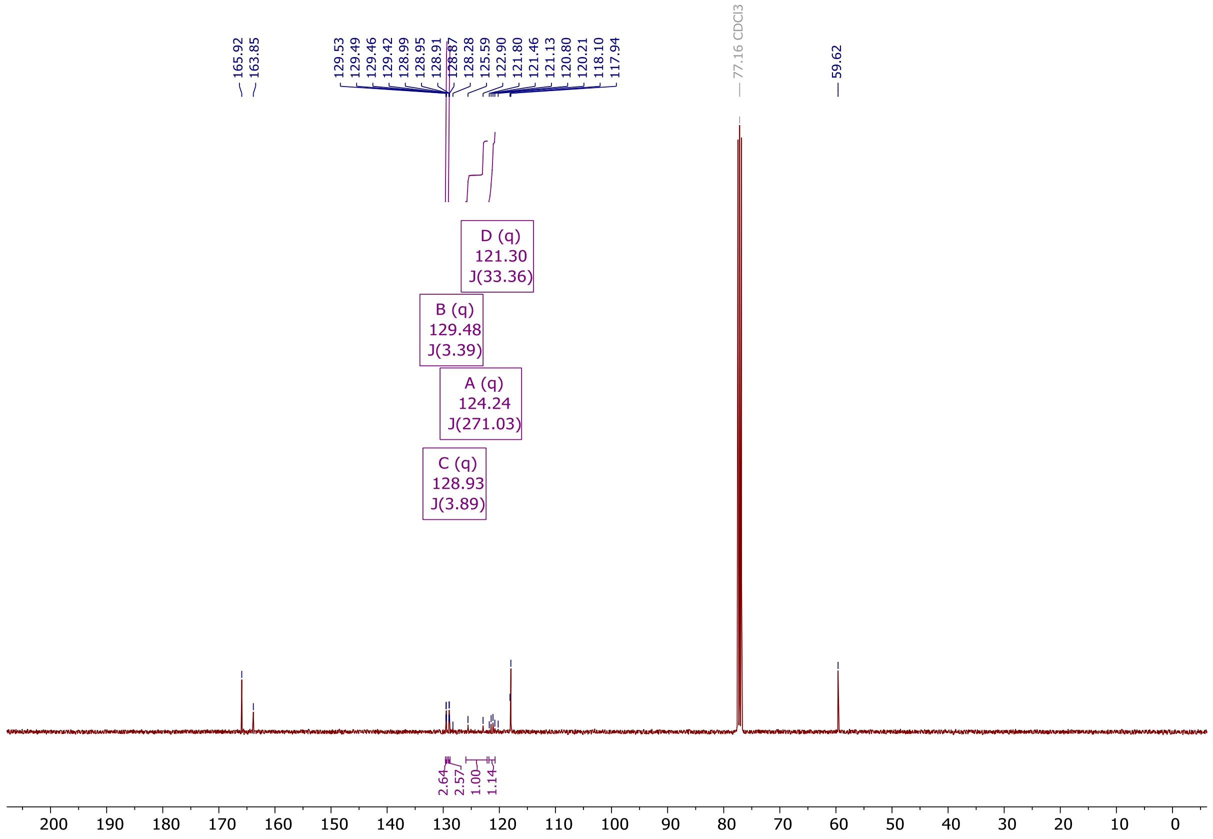

Figure S6: ^{13}C NMR Spectrum of *2,2'-((1E,1'E)-(ethane-1,2-diylbis(azaneylylidene))bis(methaneylylidene))bis(4-(trifluoromethyl)phenol)* in $CDCl_3$.

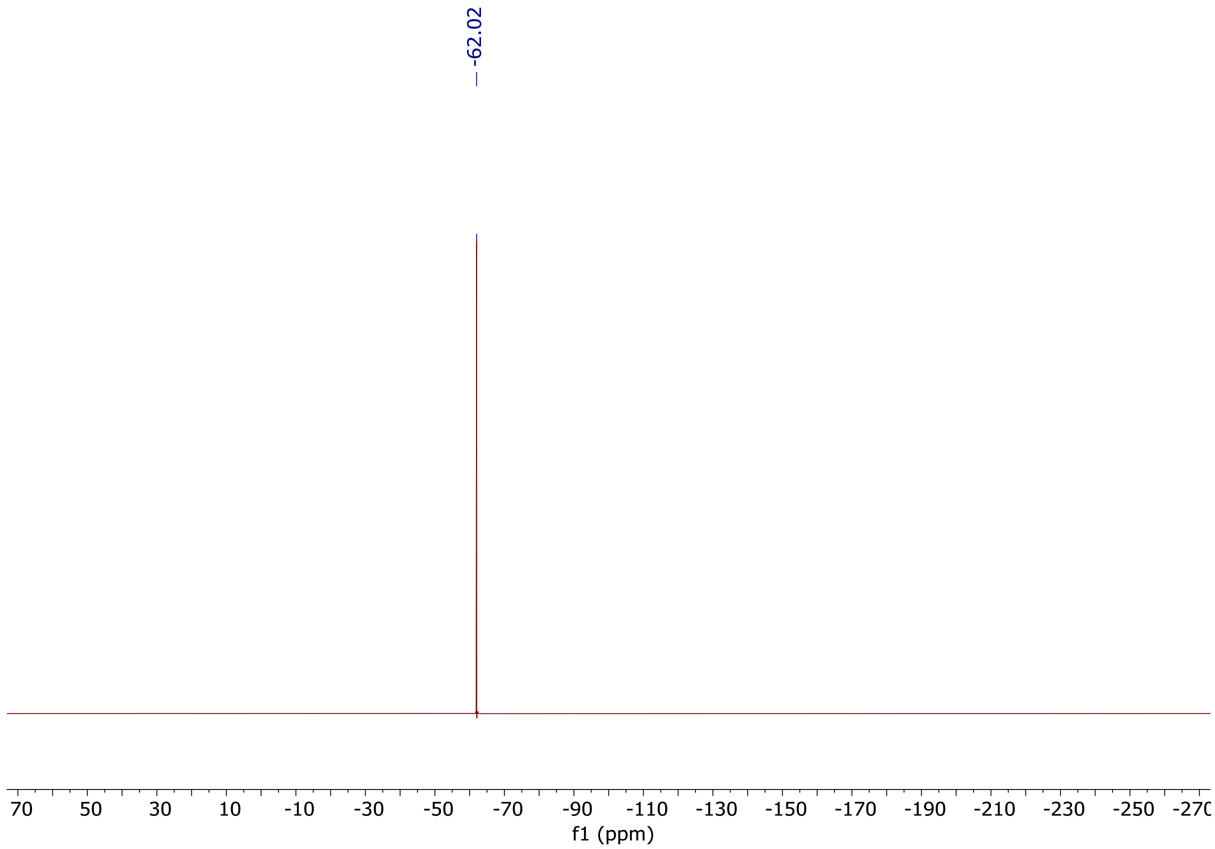

Figure S7: ^{19}*F NMR* Spectrum of *2,2'-((1E,1'E)-(ethane-1,2-diylbis(azaneylylidene))bis(methaneylylidene))bis(4-(trifluoromethyl)phenol)* in CD_3CN.

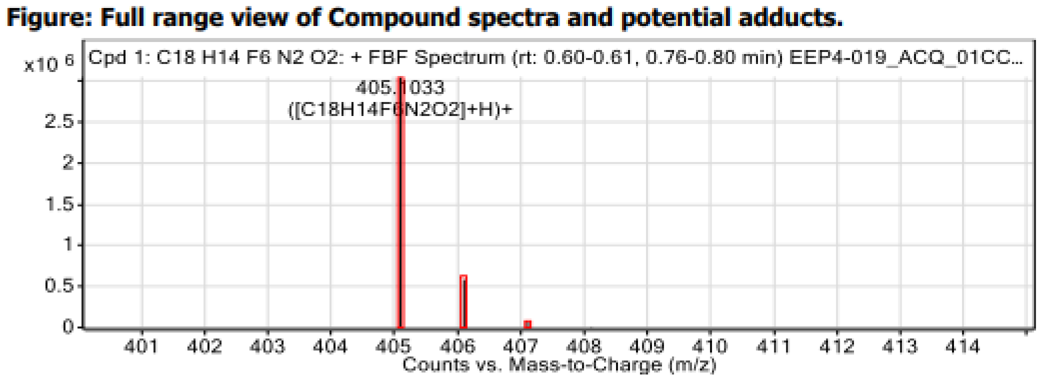

Figure S8: HRMS spectra for *2,2'-((1E,1'E)-(ethane-1,2-diylbis(azaneylylidene))bis(methaneylylidene))bis(4-(trifluoromethyl)phenol).*

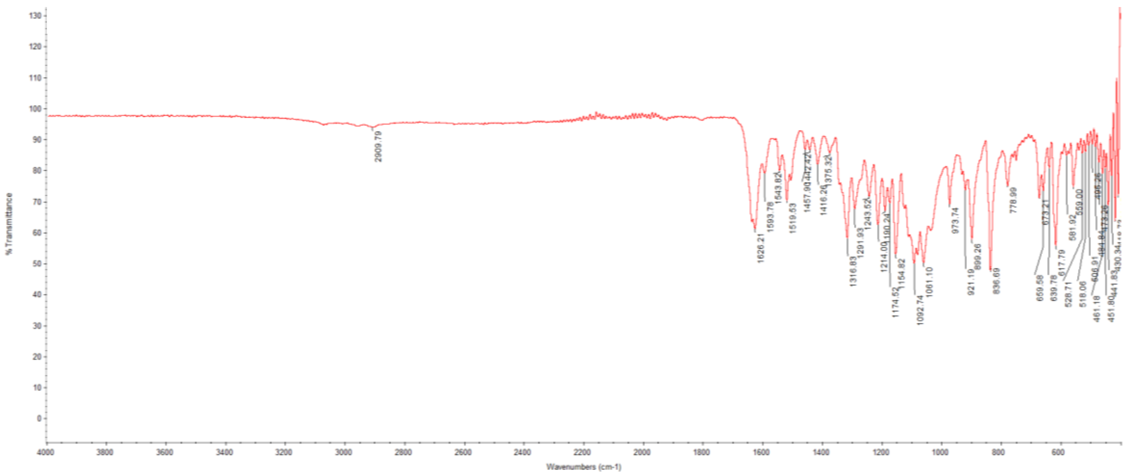

Figure S9: IR spectra for compound *2,2'-((1E,1'E)-(ethane-1,2-diylbis(azaneylylidene))bis(methaneylylidene))bis(4-(trifluoromethyl)phenol).*

2.1.3. 2,2'-((1E,1'E)-(ethane-1,2-diylbis(azaneylylidene))bis(methaneylylidene))bis(4-methoxyphenol)

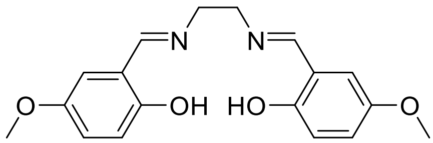

Following the general procedure and using 2-hydroxy-5-methoxybenzaldehyde (2.28 mL, 18.3 mmol) and ethylenediamine (0.35 mL, 5.2 mmol) in EtOH (40 mL). Yield: 1.68 g (98%) bright yellow solid. Data is consistent with literature.[3]

^{1}H NMR ($CDCl_3$, 500 MHz): *δ* 12.6 (s, 2H, Ar-OH), 8.31(s, 2H, N=CH), 6.92 – 6.86(m, 4H, Ar-H), 6.73 (d, J = 2.8 Hz, 2H, Ar-H), 3.94 (s, 4H, N-CH2-CH2-N) 3.75 (s, 6H, OCH_3).

^{13}C NMR ($CDCl_3$, 120 MHz): δ 166.41, 155.28, 152.17, 119.68, 118.39, 117.83, 115.07, 59.99, 56.07

HRMS (ESI+): calcd for [M, $C_{18}H_{21}N_2O_4$]$^+$ 329.1496, found 329.1498.

FT-IR (cm^{-1}): 2958.4, 1637.1, 1585.9, 1488.1, 1292.8, 1188.4, 1029.7 cm^{-1}

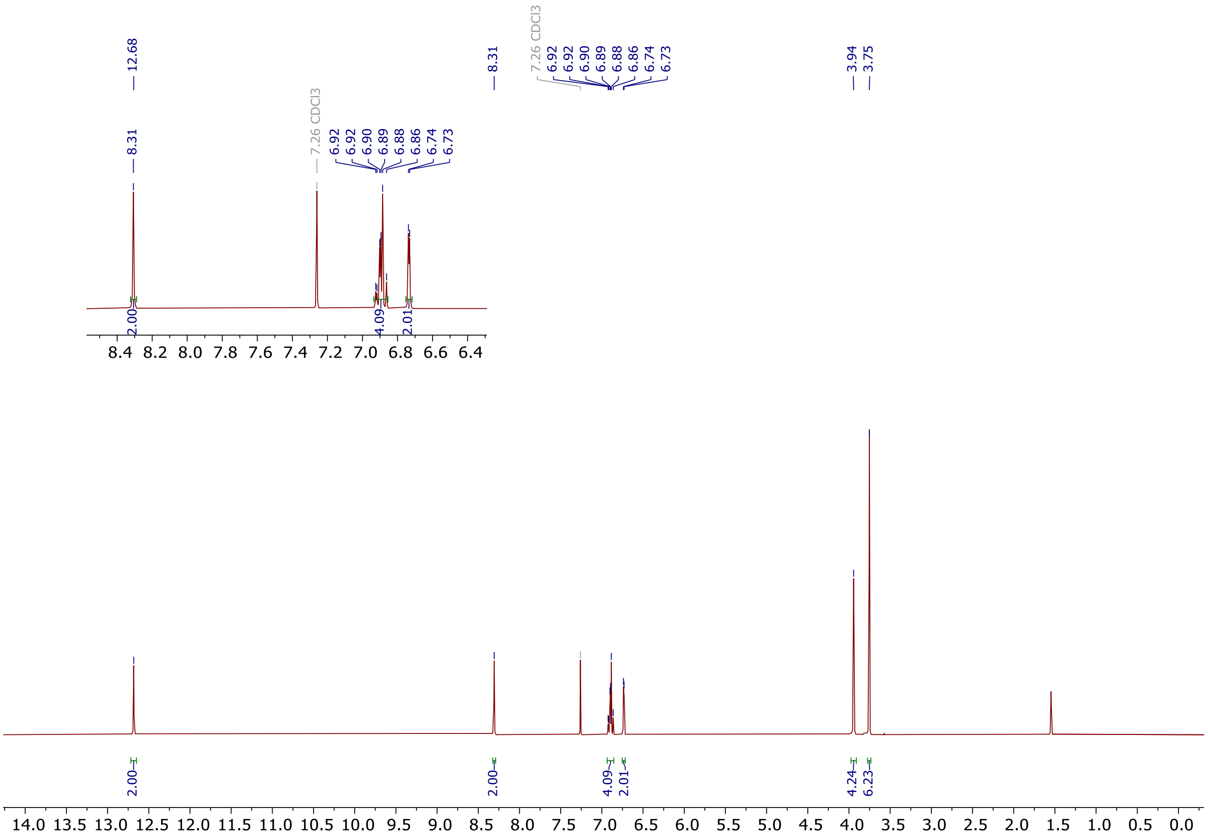

Figure S10: ^{1}H NMR Spectrum of *2,2'-((1E,1'E)-(ethane-1,2-diylbis(azaneylylidene))bis(methaneylylidene))bis(4-methoxyphenol)* in $CDCl_3$.

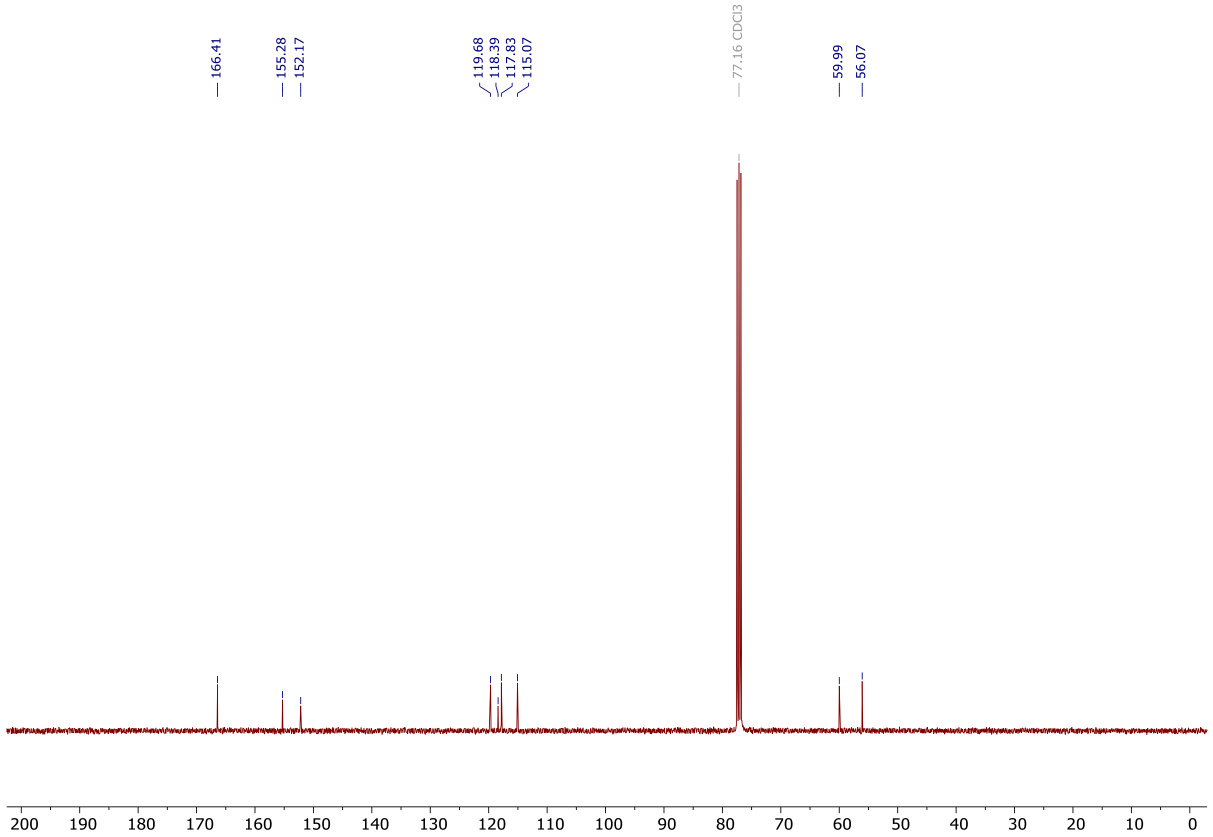

Figure S11: ^{13}C NMR Spectrum of *2,2'-((1E,1'E)-(ethane-1,2-diylbis(azaneylylidene))bis(methaneylylidene))bis(4-methoxyphenol)* in $CDCl_3$.

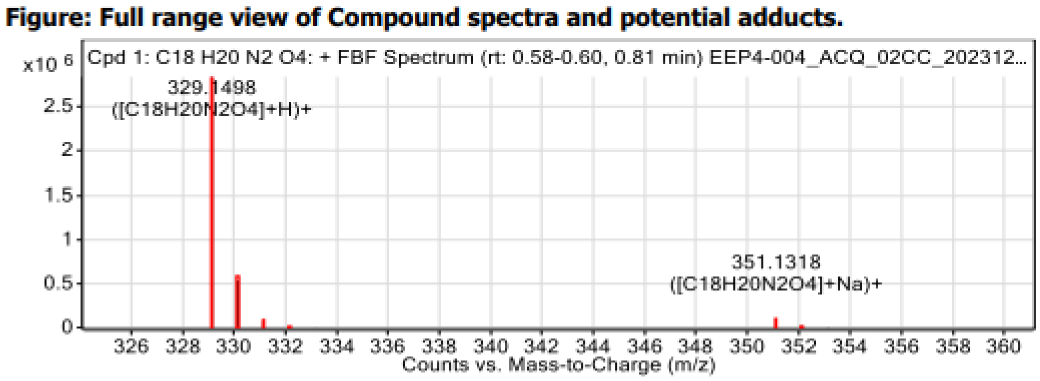

Figure S12: HRMS spectra for compound *2,2'-((1E,1'E)-(ethane-1,2-diylbis(azaneylylidene))bis(methaneylylidene))bis(4-methoxyphenol).*

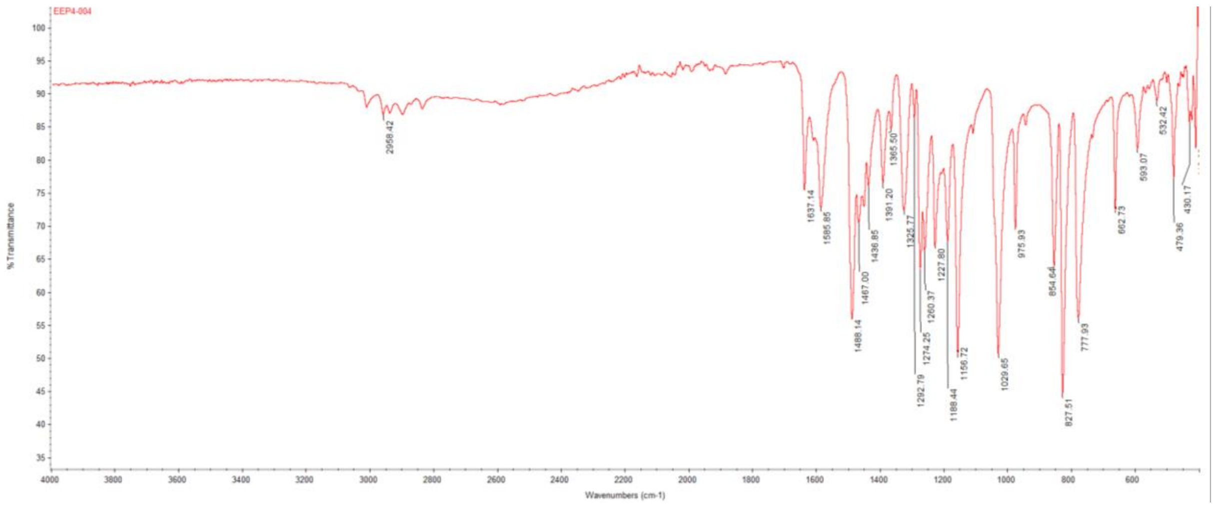

Figure S13: IR spectra for compound *2,2'-((1E,1'E)-(ethane-1,2-diylbis(azaneylylidene))bis(methaneylylidene))bis(4-methoxyphenol).*

2.2. General Method for the synthesis of Fe-salen Complexes[1]

$Fe(OAc)_2$ (1 equiv.) was weighed into a round bottom flask and dissolved in EtOH resulting in a dark brown solution. To this, a solution of ligand (1.2 equiv.) in EtOH was added yielding a red solution. The resulting red solution was stirred at 80 °C for 2 hours. The flask was then allowed to cool to room temperature before the resulting solid residue was isolated via vacuum filtration.

2.2.1. Complex **1a**[1]

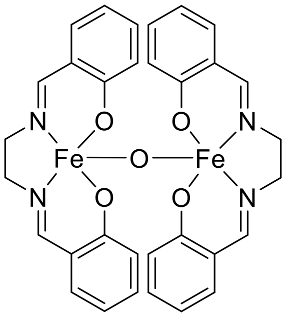

Following the general procedure and using N,N'-Bis(salicylidene)ethylenediamine (1.31 g, 4.55 mmol) and $Fe(OAc)_2$ (0.71 g, 3.9 mmol) and EtOH (50 mL). Isolated as a maroon red powder. Yield: 1.39 g (54%). Data is consistent with literature.[1]

^{1}H NMR ($CDCl_3$, 500 MHz): δ (all br.) 80.69, 49.60, 22.30, 19.76, 12.46, 9.71, 8.36, 6.91 – 6.83, 3.94, 2.77, 1.52, 1.21, 0.24, -73.35
HRMS (ESI+): calcd for $[M+H, C_{32}H_{29}O_5N_4Fe_2]^+$ 661.0831, found 661.0815.
FT-IR (cm^{-1}): 2910.9 (CH aromatic), 1626.3 (C=N), 858.7 (Fe-O-Fe), 774.1 (=CH) cm^{-1}.
UV-Vis (298 K, CH_3CN): $\lambda_{max,1}$ = 480 nm

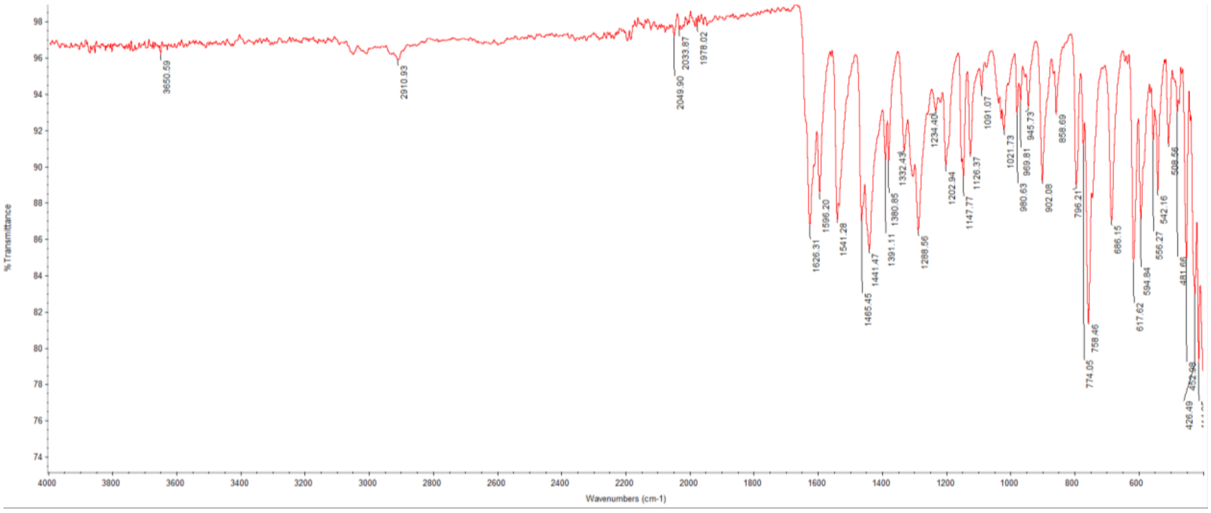

Figure S14: IR spectra for compound **1a**.

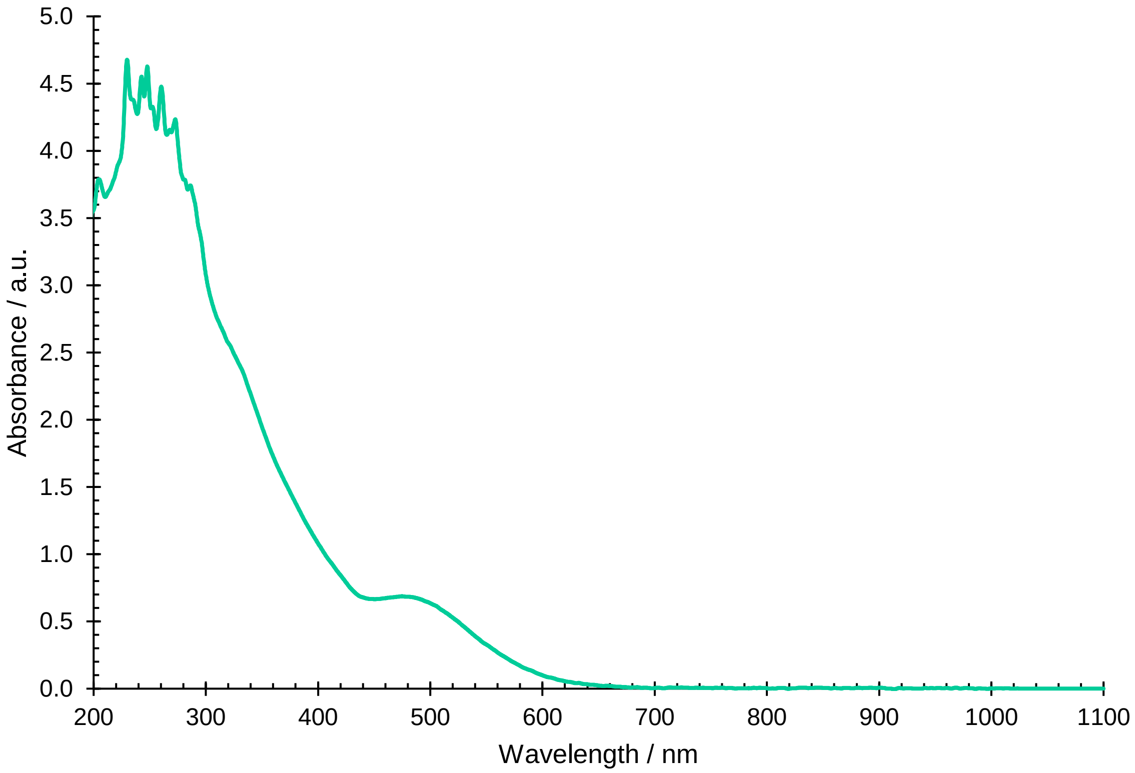

Figure S15: UV-vis spectra of compound **1a** in CH_3CN.

2.2.2. Complex **1b**

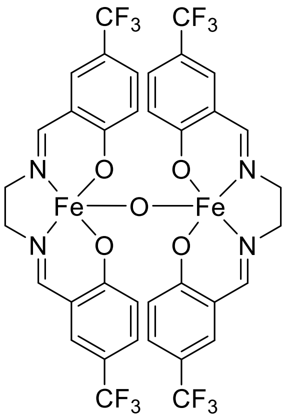

Following the general procedure using 2,2'-((1E,1'E)-(ethane-1,2-diylbis(azaneylylidene))bis(methaneylylidene))bis(4-(trifluoromethyl)phenol) (0.473 g, 1.17 mmol) and $Fe(OAc)_2$ (0.178 g, 0.98 mmol) and EtOH (30 mL). Isolated as a orange powder. Yield: 0.21 g (23%). Crystals suitable for x-ray diffraction studies were obtained through slow evaporation of MeCN.

^{1}H NMR ($CDCl_3$, 500 MHz): δ 40.89, 23.79, 19,67, 12.41, 10.03, 1.28 (all br.) .
HRMS (ESI+): calcd for $[M+H, C_{36}H_{25}N_4O_5F_{12}Fe_2]^+$ 933.0332, found 933.0319.
FT-IR (cm^{-1}): 2910.8 (CH aromatic), 1185.9 cm^{-1} (C-F), 1619.9 (C=N), 849.4 (Fe-O-Fe), 788.1 (=CH) cm^{-1}.
UV-Vis (298 K, CH_3CN): $\lambda_{max,1}$ = 230 nm, $\lambda_{max,2}$ = 242 nm, $\lambda_{max,3}$ = 248 nm, $\lambda_{max,4}$ = 260 nm, $\lambda_{max,5}$ = 286 nm, $\lambda_{max,6}$ = 346 nm.
XRD: see section crystallographic data.

Elemental Composition Report **Page 1**

Single Mass Analysis
Tolerance = 1000.0 PPM / DBE: min = -1.5, max = 50.0
Element prediction: Off
Number of isotope peaks used for i-FIT = 3

Monoisotopic Mass, Even Electron Ions
1152 formula(e) evaluated with 1 results within limits (up to 50 closest results for each mass)
Elements Used:
C: 0-36 H: 0-25 N: 0-4 O: 0-5 F: 0-12 Fe: 0-2
RW N WEBSTER NB071
RW N WEBSTER NB071 161 (3.154) AM (Cen,4, 80.00, Ar,10000.0,0.00,0.00); Cm (7:164) 1: TOF MS ES+ 1.19e+007

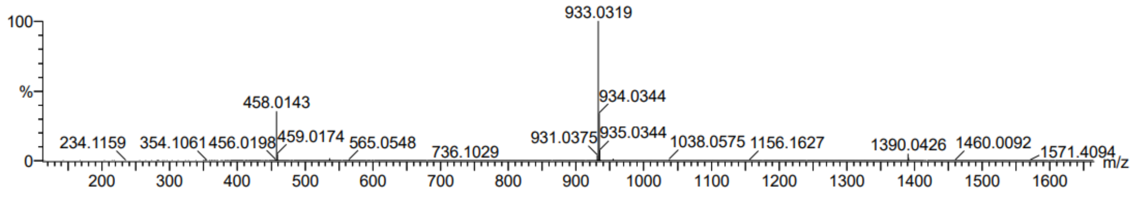

Minimum:				-1.5				
Maximum:		5.0	1000.0	50.0				
Mass	Calc. Mass	mDa	PPM	DBE	i-FIT	Norm	Conf(%)	Formula
933.0319	933.0332	-1.3	-1.4	20.5	262.8	n/a	n/a	C36 H25 N4 O5 F12 Fe2

Figure S16: HRMS spectra for compound **1b**.

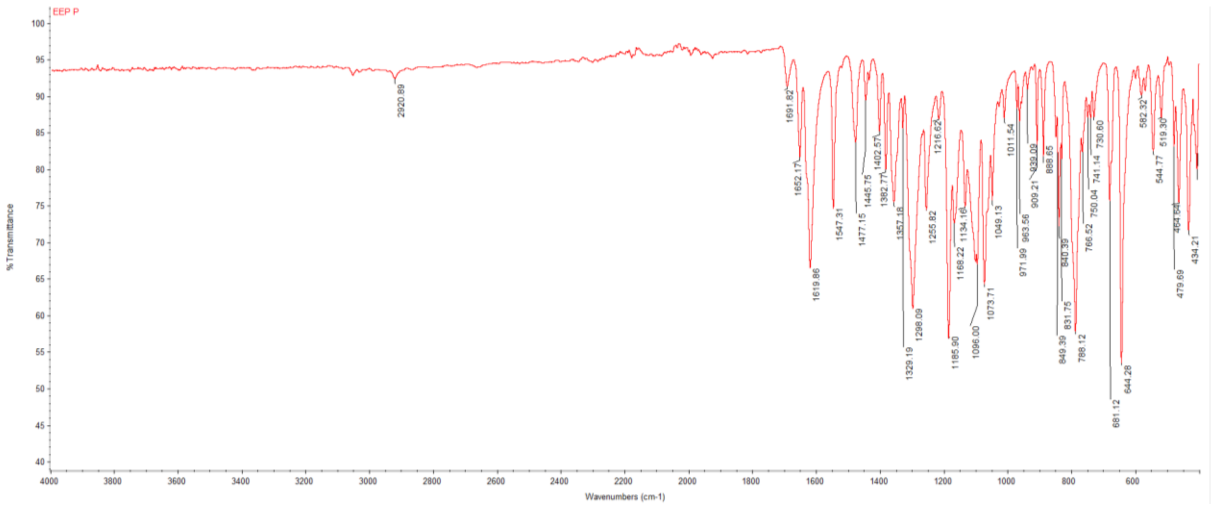

Figure S17: IR spectra for compound **1b**.

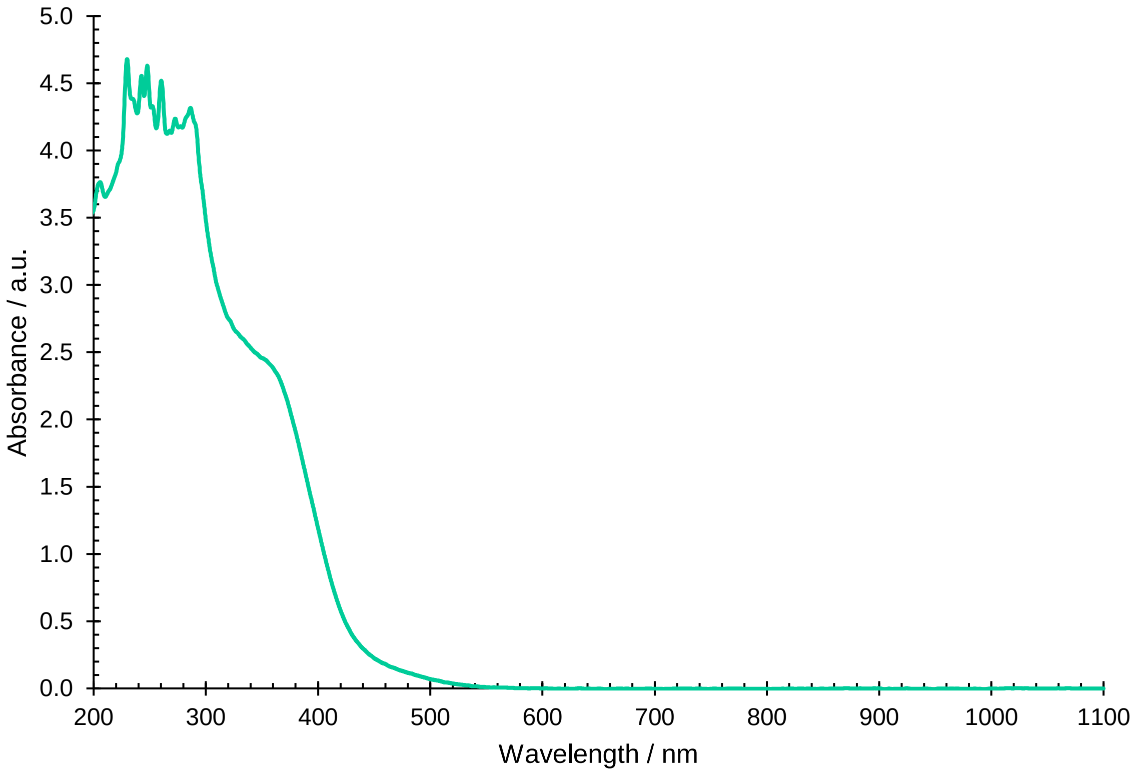

Figure S18: UV-vis spectra of compound **1b** in CH_3CN.

2.2.3. Complex 1c

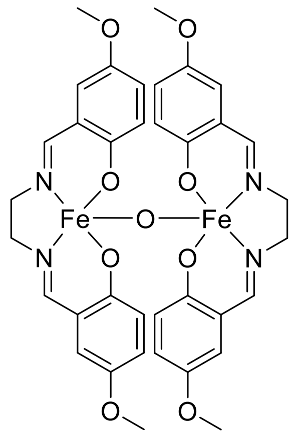

Following the general procedure and using 2,2'-((1E,1'E)-(ethane-1,2-diylbis(azaneylylidene))bis(methaneylylidene))bis(4-methoxyphenol) (1.49 g, 4.55 mmol) and $Fe(OAc)_2$ (0.71 g, 3.90 mmol) and EtOH (50 mL). Isolated as a dark purple powder. Yield: 1.069 g (35%). Crystals suitable for x-ray diffraction studies were obtained through slow evaporation of DCM.[4]

^{1}H NMR ($CDCl_3$, 500 MHz): δ (all br.) 73.6, 44.9, 13.2, 8.31, 7.27, 6.90, 3.76, 1.27, -72.2.
HRMS (ESI+): calcd for $[M+H, C_{36}H_{37}O_9N_4Fe_2]^+$ 781.1254, found 781.1268.
FT-IR (cm^{-1}): 2928.9 (CH aromatic), 1607.5 (C=N), 1282.5 ($O\text{-}CH_3$), 847.7(Fe-O-Fe), 776.0 (=CH) cm^{-1}.
UV-Vis (298 K, CH_3CN): $\lambda_{max,1}$ = 246 nm, $\lambda_{max,2}$ = 283 nm, $\lambda_{max,3}$ = 373 nm, $\lambda_{max,4}$ = 524 nm.
XRD: see section crystallographic data.

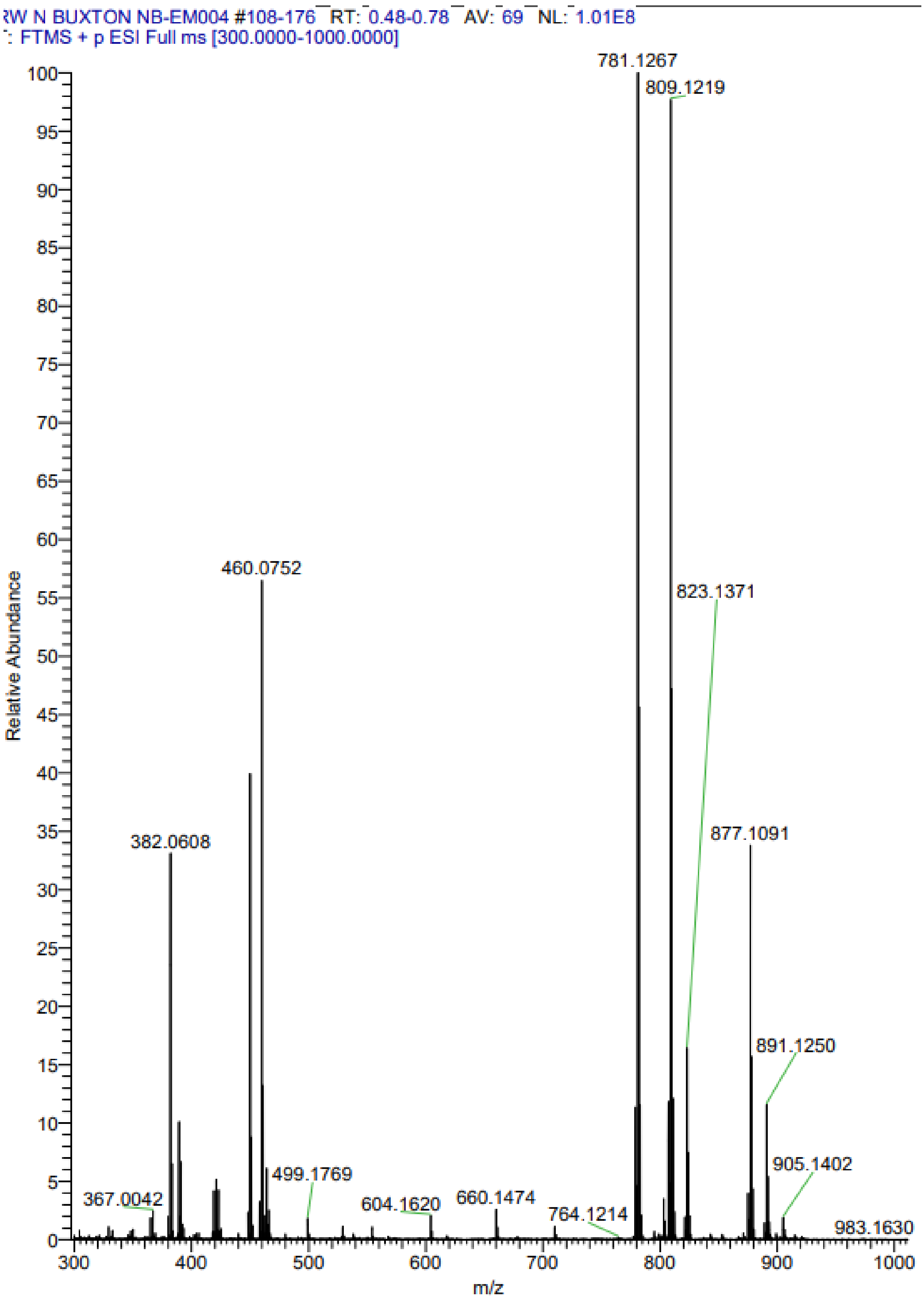

Figure S19: HRMS spectra for compound **1c**.

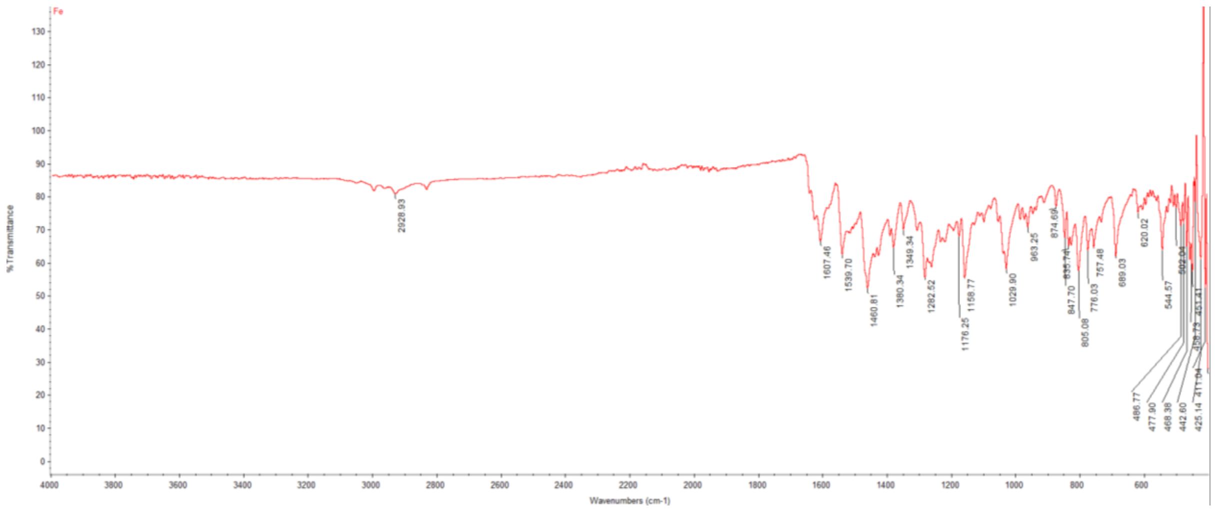

Figure S20: IR spectra for compound **1c**.

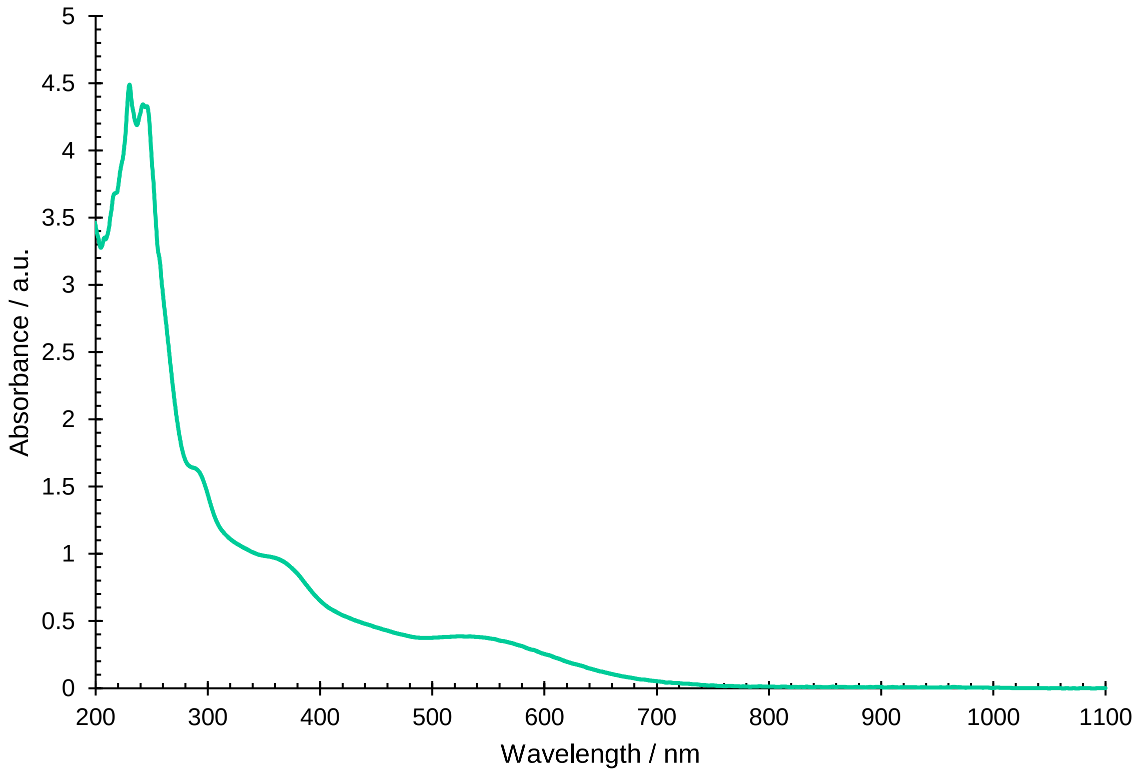

Figure S21: UV-vis spectra of compound **1c** in CH_3CN.

2.3. Cyclic Voltammetry

Electrochemical measurements were performed in a 0.2 M solution of tetrabutylammonium hexafluoride solution, with 0.2 mM concentration of iron µ-oxo(salen) complex (**1a-1c**) and a 0.2 mM standard of ferrocene in acetonitrile. A 3-electrode cell was used for the electrochemical experiments employing a separate working, counter and reference electrode. Cyclic voltammograms were cycled three or four times to obtain steady state conditions. The working electrode was a polished glassy carbon disc (3mm diameter) electrode. The electrode was polished and clean between each separate experiment. A platinum (Pt(0)) wire was used as the counter electrode. A silver (Ag(0)) wire was used as the reference electrode. The reference and counter electrode were cleaned with IPA and flamed between each experiment. All electrochemical measurements were ran under an inert nitrogen atmosphere, where the solutions were degassed by sparging with nitrogen for 15 minutes. Between measurements the solutions were stirred briefly to renew the layer of solution near the surface of the electrodes.

2.3.1 Complex **1a**

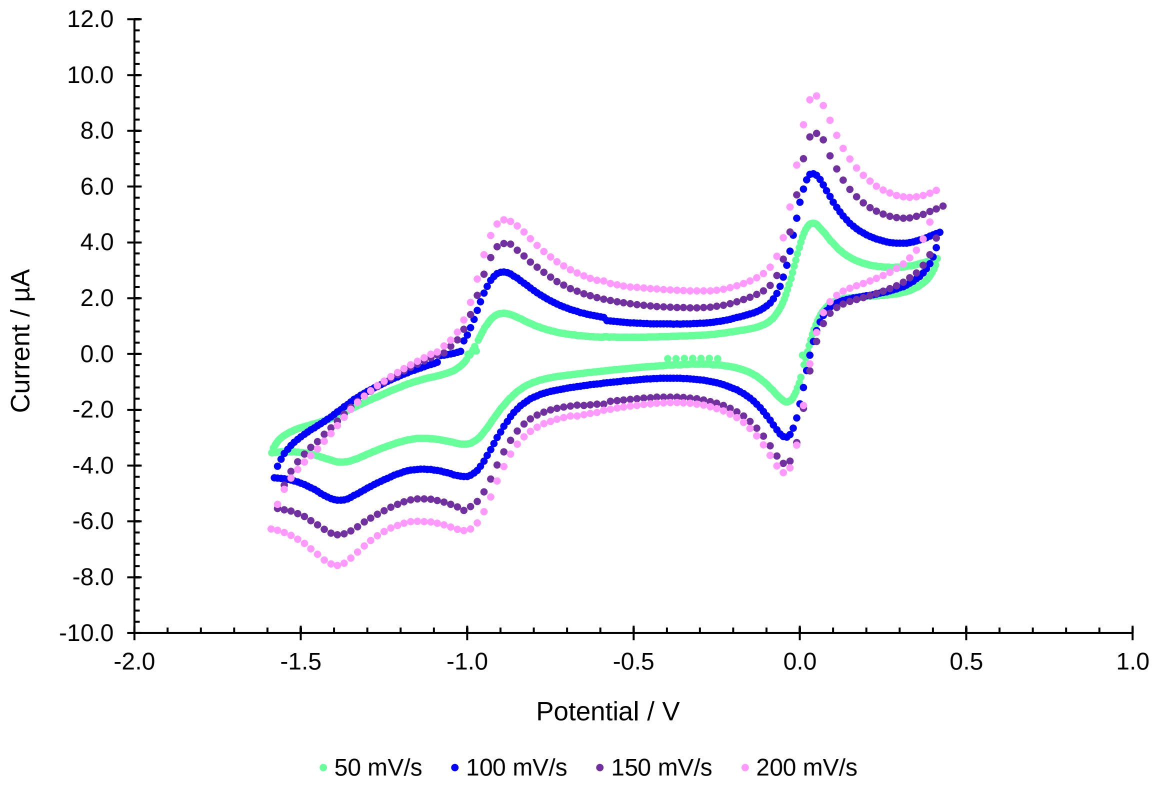

Figure S22: Cyclic voltammogram of complex **1a** at multiple scan rates, referenced to a ferrocene standard (0.2 mM).

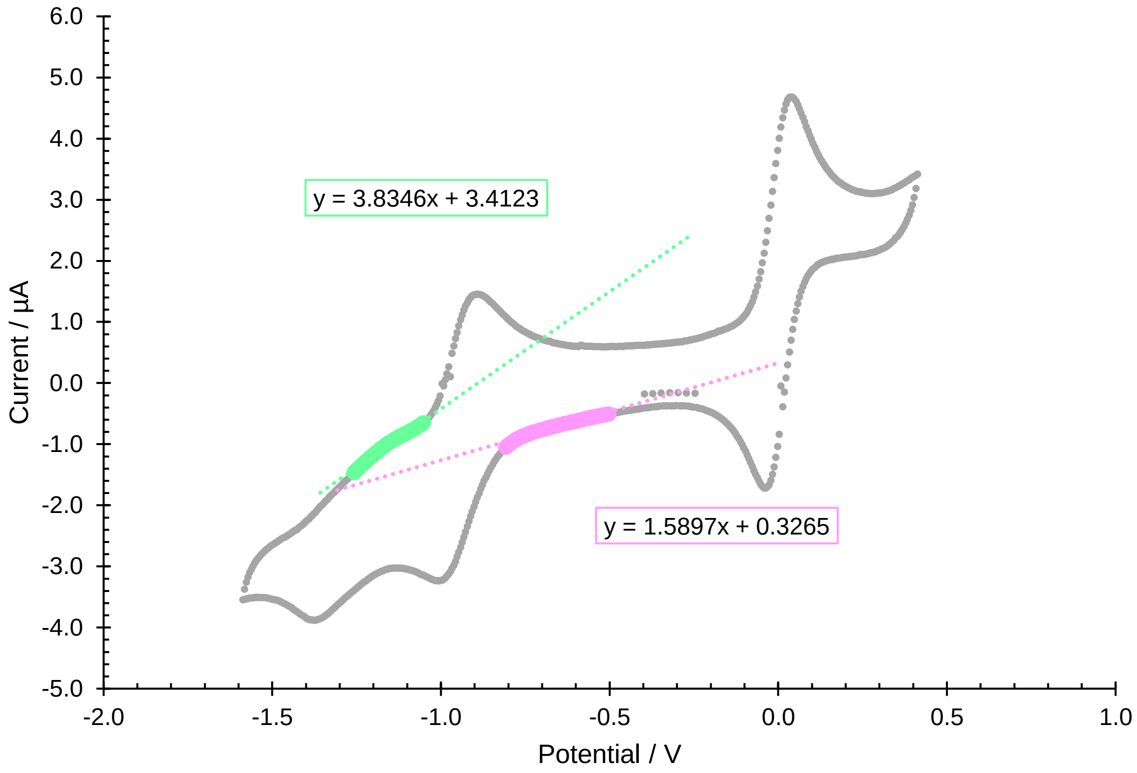

Figure S23: Cyclic voltammogram for complex **1a** at a scan rate of 50 mV/s, used to calculate the Nicholson parameter and the formal electrode potential.

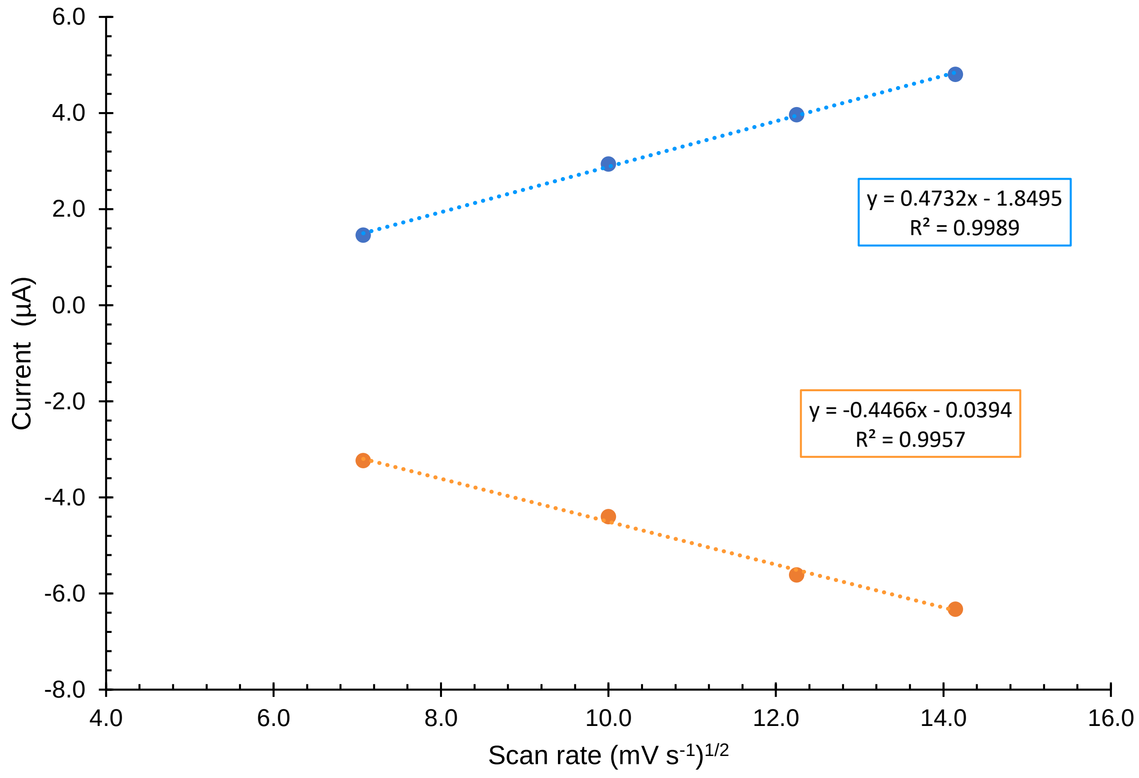

Figure S24: Randles-Sevcik plot for compound **1a**.

2.3.2. Complex **1b**

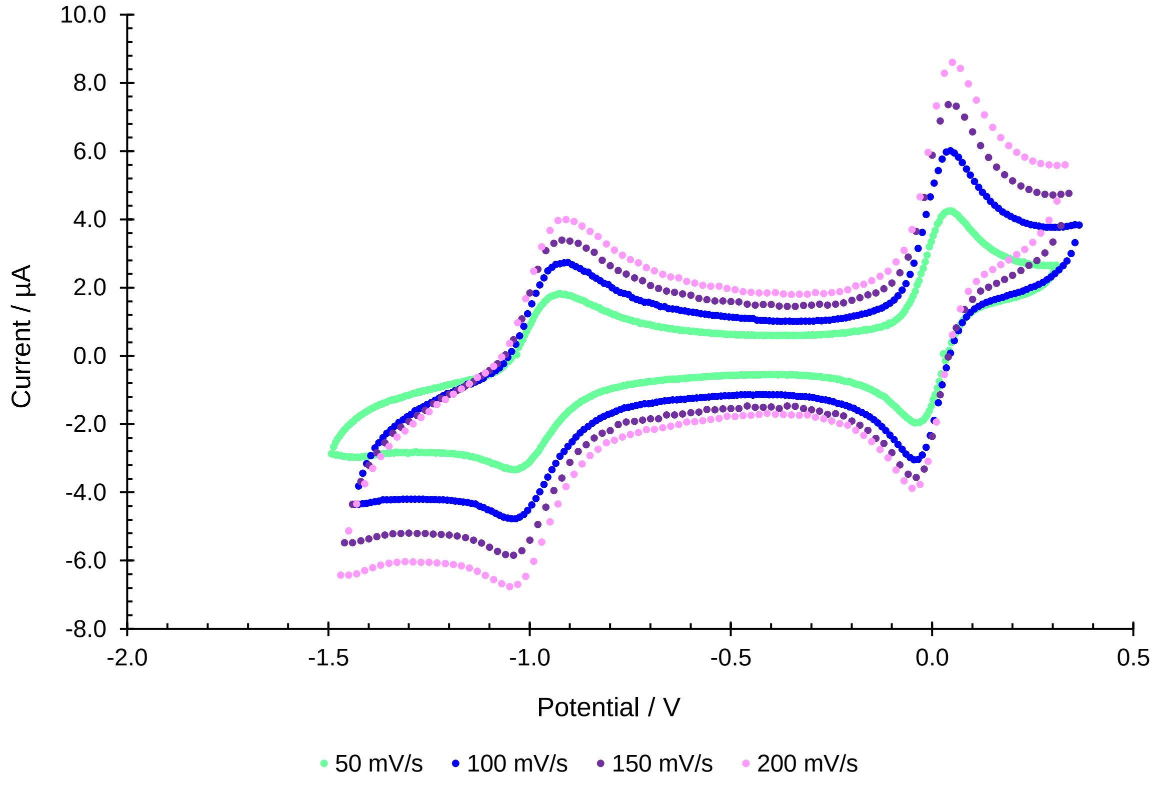

Figure S25: Cyclic voltammogram of complex **1b** at multiple scan rates.

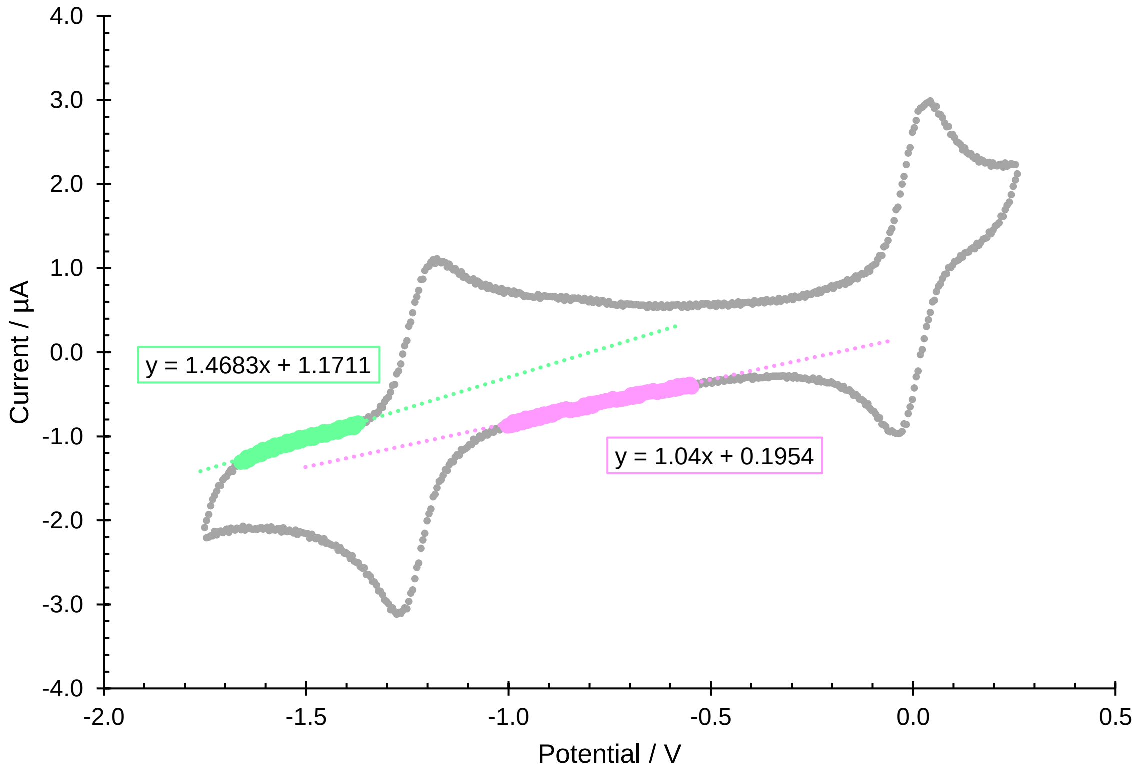

Figure S26: Cyclic voltammogram for complex **1b** at a scan rate of 50 mV/s, used to calculate the Nicholson parameter and the formal electrode potential.

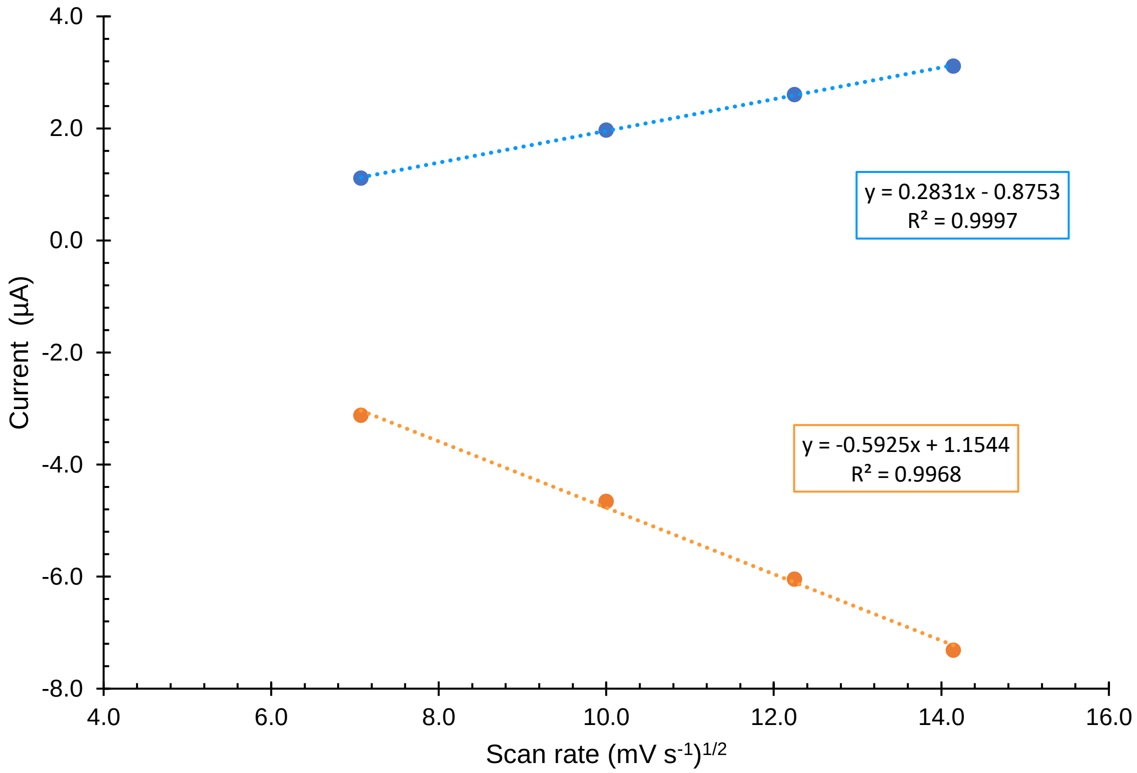

Figure S27: Randles-Sevcik plot for compound **1b**.

2.3.3. Complex **1c**

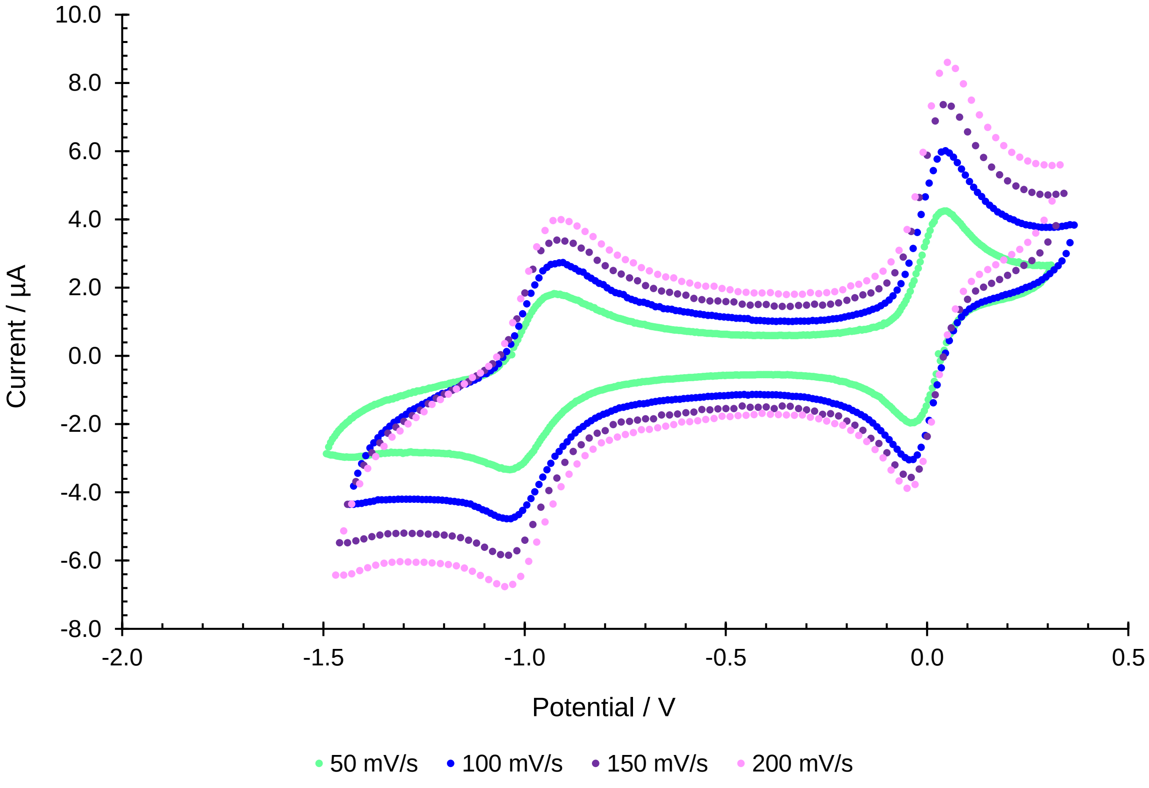

Figure S28: Cyclic voltammogram of complex **1c** at multiple scan rates.

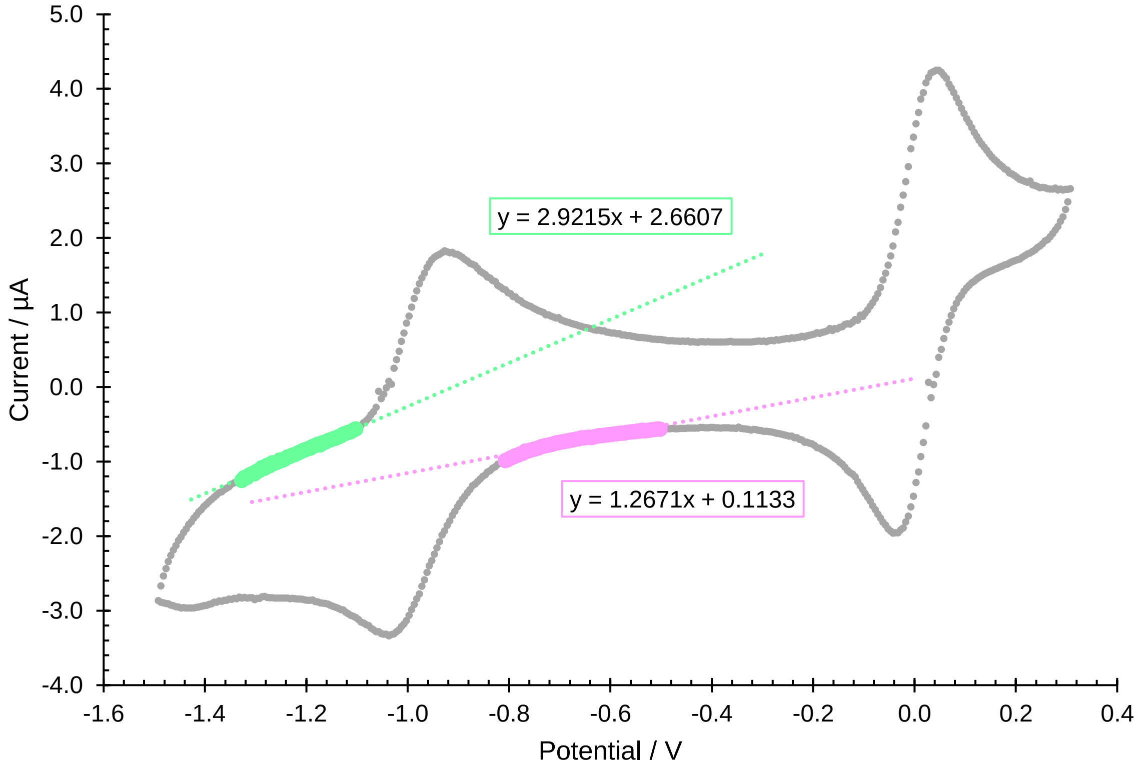

Figure S29: Cyclic voltammogram for complex **1c** at a scan rate of 50 mV/s, used to calculate the Nicholson parameter and the formal electrode potential.

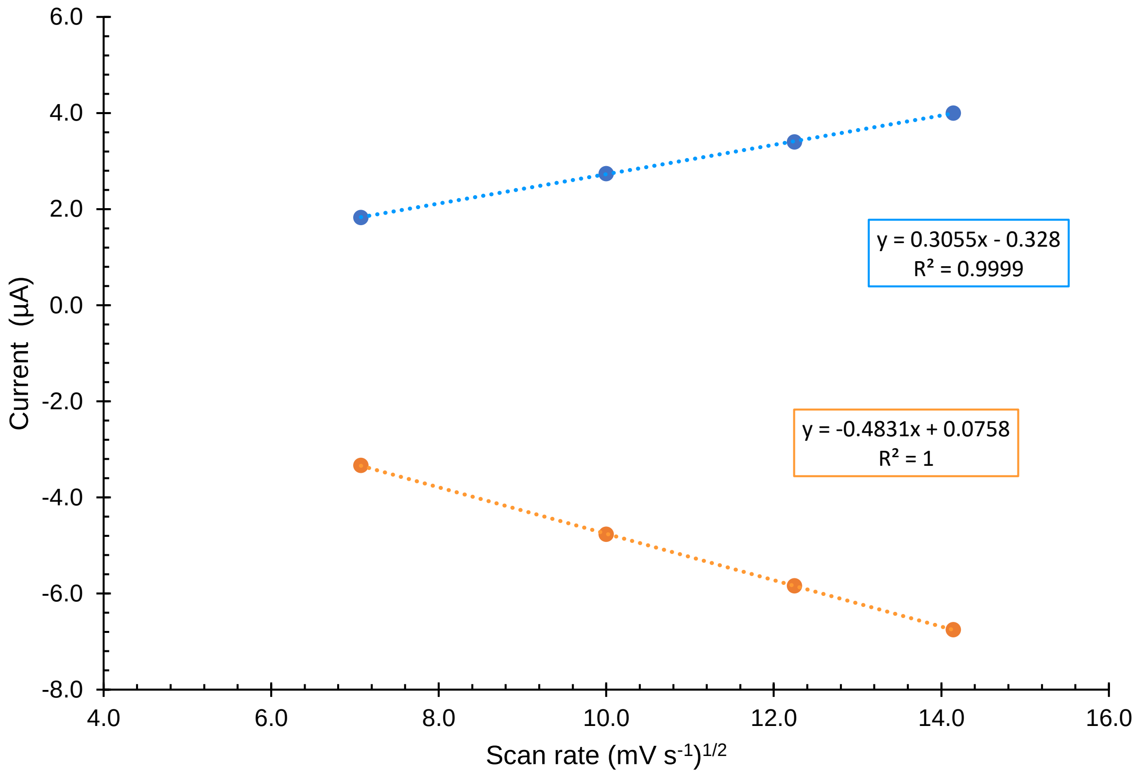

Figure S30: Randles-Sevcik plot for compound **1c**.

3. One-Pot Hydroaminations

3.1. General Procedure for the One-Pot Hydroaminations

To an ampule fitted with a magnetic stirrer bar under nitrogen was added a mixture of **Fe-salen pre-catalyst** (0.005 mmol) and nitro-compound (1.0 mmol, 1 equiv.) in dry acetonitrile (0.5 mL). To the solution was then added donor olefin (3 mmol, 3 equiv.) followed by phenyl silane (2 mmol, 2 equiv.) The reaction mixture was then stirred at 80 °C under a positive flow of nitrogen for 22h. After the reaction had gone to completion the solvents were removed under vacuum. The resulting crude product was then purified by flash column chromatography on silica using hexane/dichloromethane.

3.1.1. N-(4-(tert-butyl)phenyl)-2,3-dihydro-1H-inden-1-amine **3**

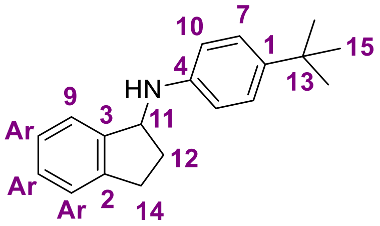

Yellow oil

R_f = 0.54 (30% DCM / 70% hexane)

^{1}H NMR ($CDCl_3$, 500 MHz): δ 7.43 (d, J = 7.2 Hz, 1H, C^9-H), 7.32 – 7.24 (m, 3H, Ar), 7.30 (app. d, J = 8.7 Hz, 2H, C^7-H), 6.72 (app. d, J = 8.7 Hz, 2H, C^{10}-H), 5.04 (app. t, J = 6.8 Hz, 1H, C^{11}-H), 3.85 (*br* s, 1H, NH), 3.06 (ddd, J = 15.9, 8.7, 4.3 Hz, 1H, C^{14}-H), 2.93 (dt, J = 15.9, 7.9 Hz, 1H, C^{14}-H), 2.65 – 2.59 (m, 1H, C^{12}-H), 2.00 – 1.92 (m, 1H, C^{12}-H), 1.37 (s, 9H, C^{15}-H).

^{13}C{^{1}H} NMR ($CDCl_3$, 126 MHz): δ 145.5 (C^4), 144.9 (C^2), 143.7 (C^3), 140.2 (C^1), 127.9 (Ar), 126.7 (Ar), 126.2 (C^7), 124.9 (Ar), 124.4 (C^9), 112.9 (C^{10}), 58.9 (C^{11}), 34.1 (C^{12}), 34.0 (C^{13}), 31.7 (C^{14}), 30.4 (C^{15}).

HRMS (ESI+): calcd for [M, $C_{19}H_{23}N$]$^+$ 266.1903, found 266.1902.

IR (Neat): 3397, 2957, 1614, 1517, 817, 740 cm^{-1}.

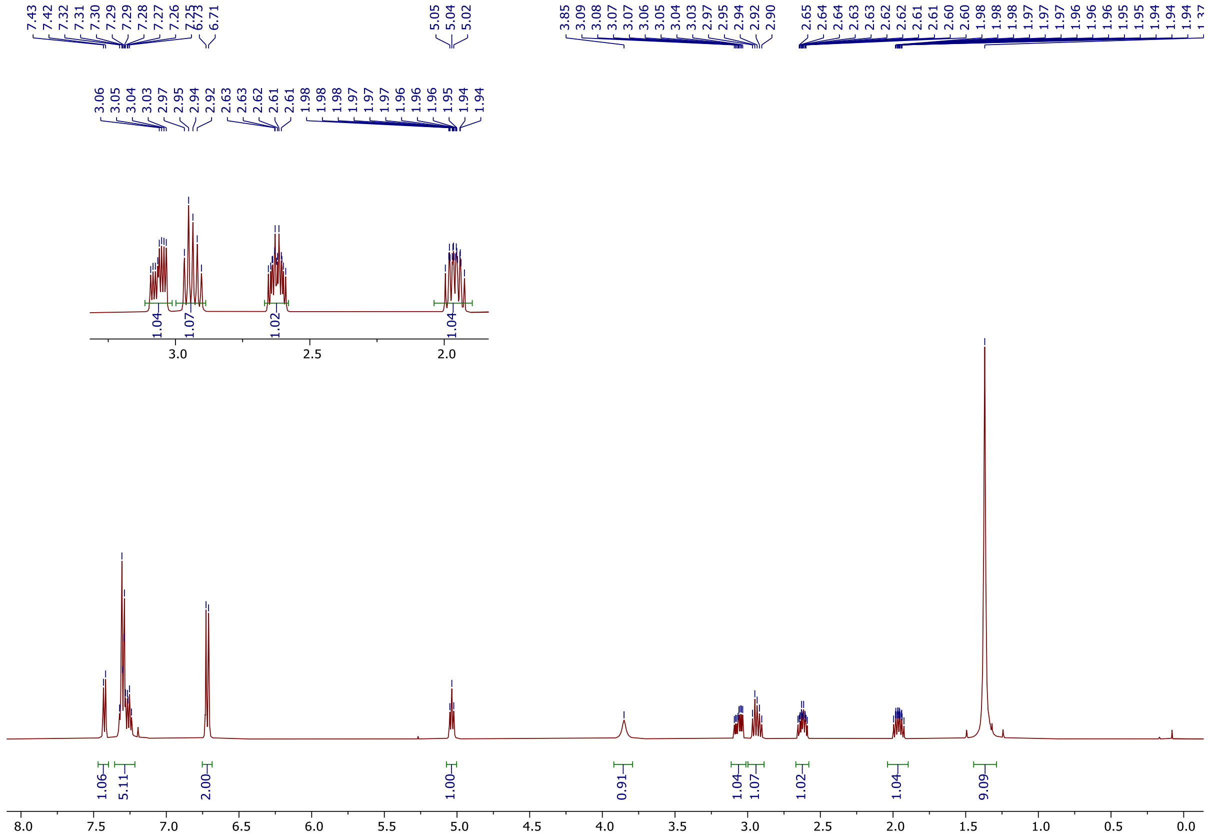

Figure S31: ^{1}H NMR Spectrum of **3** in $CDCl_3$ after isolation via column chromatography.

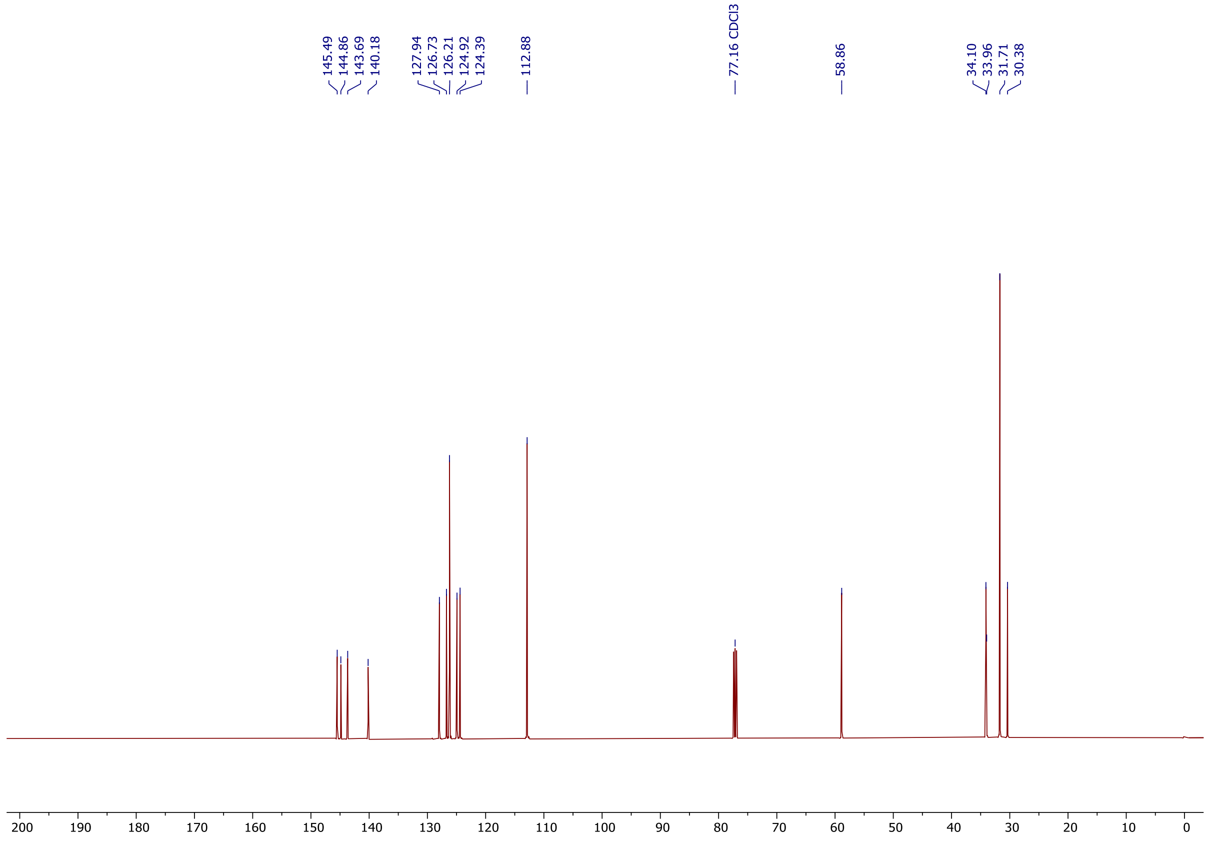

Figure S32: ^{13}C NMR Spectrum of **3** in $CDCl_3$ after isolation via column chromatography.

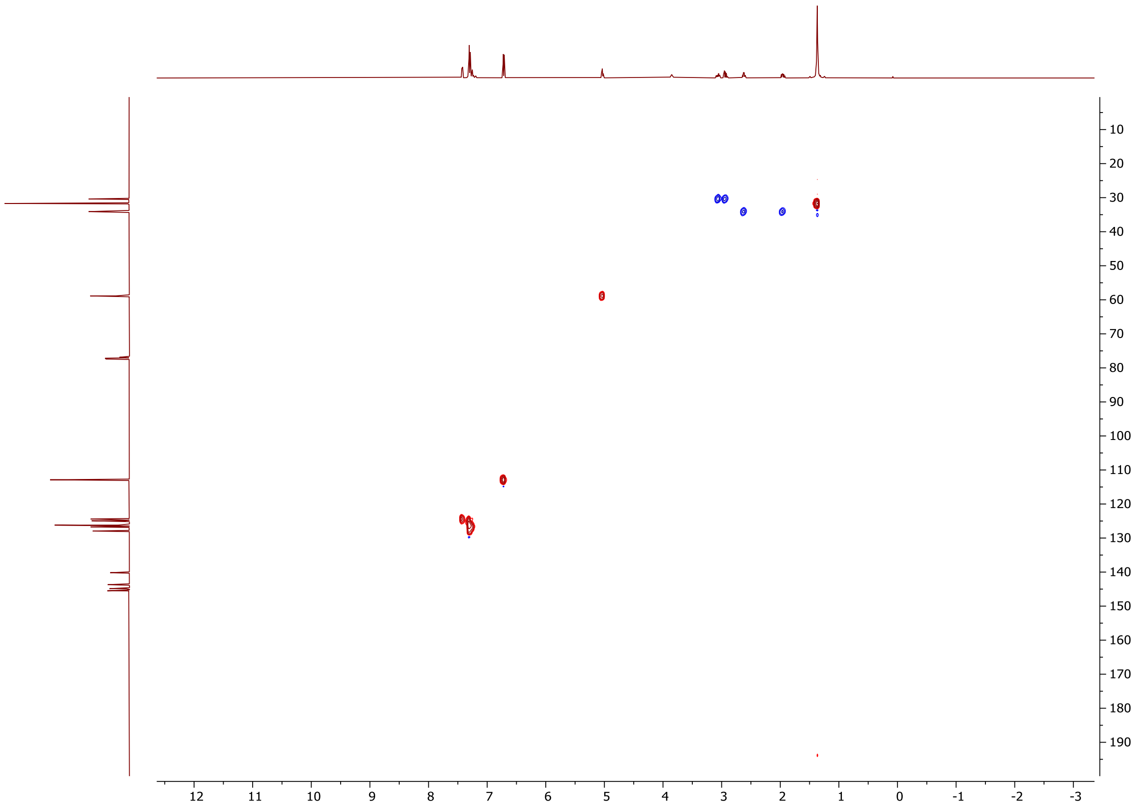

Figure S33: ^{1}H-^{13}C HSQC NMR Spectrum of **3** in $CDCl_3$ after isolation via column chromatography.

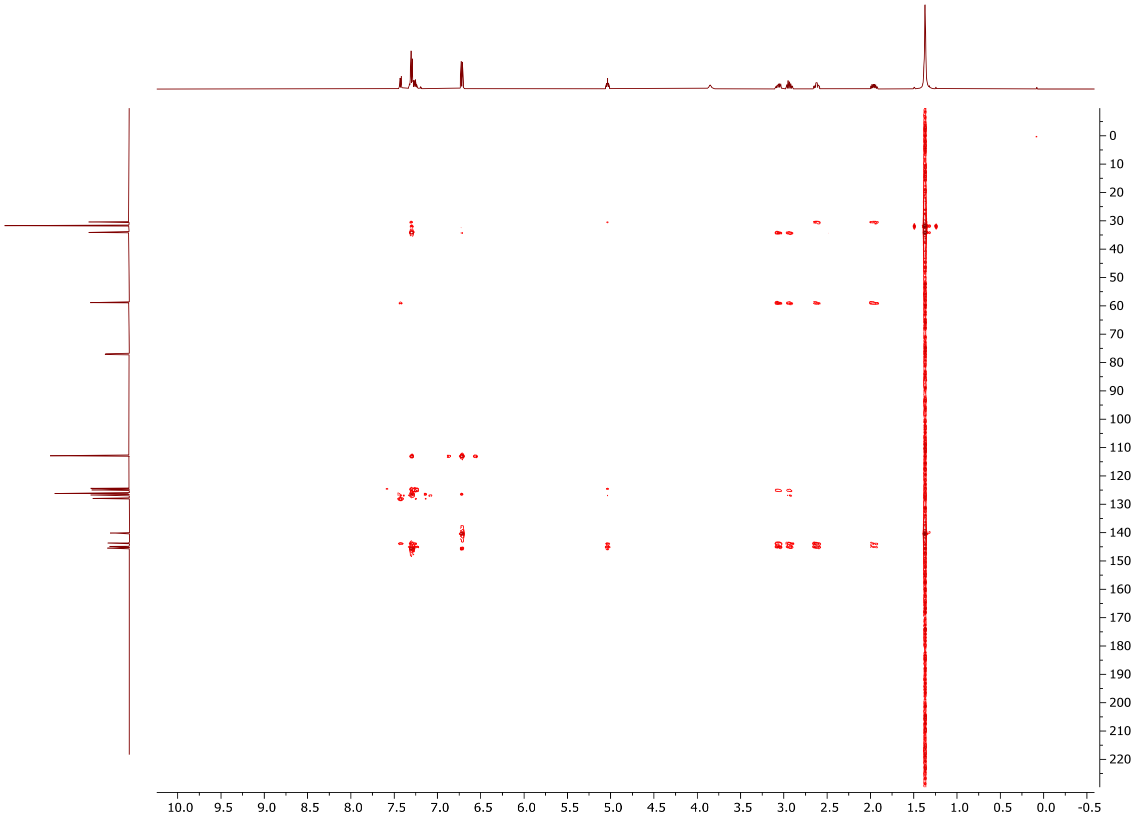

Figure S34: ^{1}H-^{13}C HMBC NMR Spectrum of **3** in $CDCl_3$ after isolation via column chromatography.

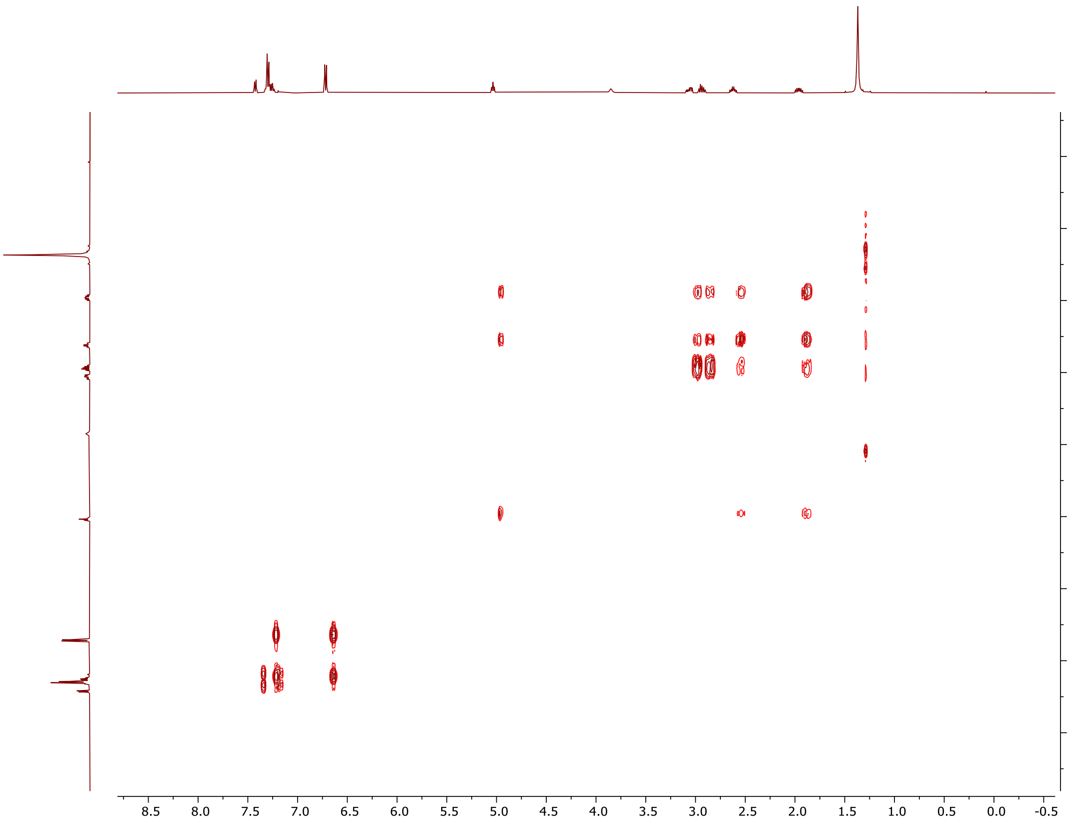

Figure S35: ^{1}H-^{1}H COSY NMR Spectrum of **3** in $CDCl_3$ after isolation via column chromatography.

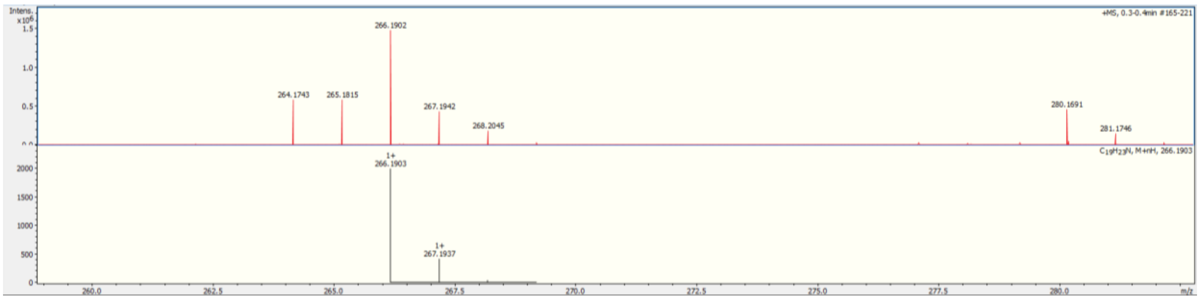

Figure S36: HRMS spectra for compound **3**.

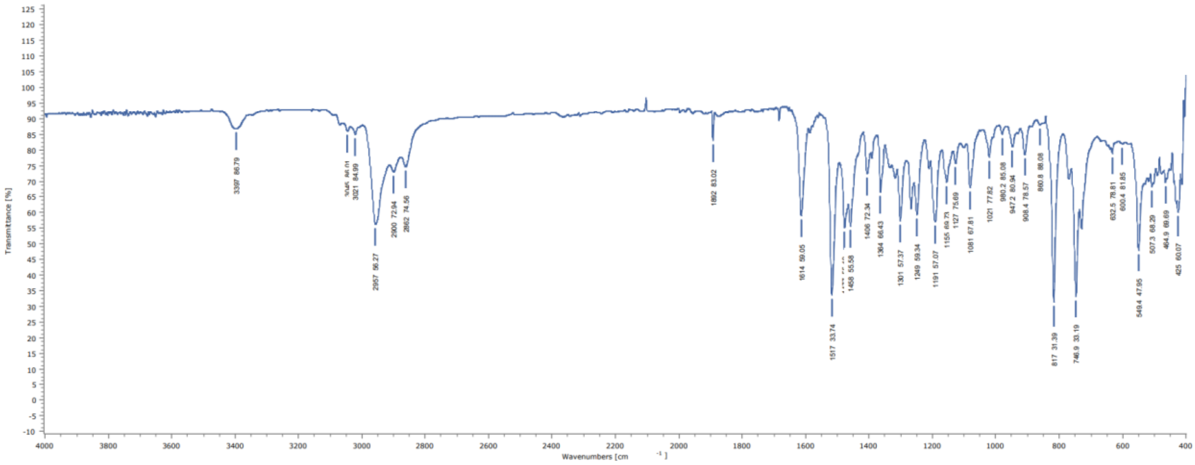

Figure S37: IR spectra for compound **3**.

3.1.2. N-(4-(tert-butyl)phenyl)-N,O-bis(2,3-dihydro-1H-inden-1-yl)hydroxylamine **4**
Mixture of Diastereoisomers

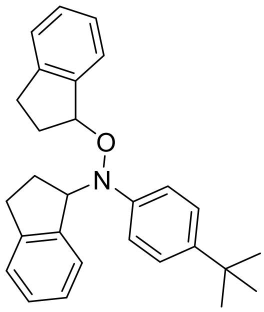

Light yellow oil

R_f = 0.68 (30% DCM / 70% hexane)

^{1}H NMR ($CDCl_3$, 500 MHz): δ 7.56 (d, *J* = 6.9 Hz, 1H), 7.46 (d, *J* = 7.3 Hz, 1H), 7.41 – 7.08 (m, 21H), 6.76 (d, *J* = 7.3 Hz, 1H), 5.03 (dd, *J* = 8.3, 2.9 Hz, 1H), 4.98 – 4.92 (m, 2H), 4.75 (dd, *J* = 5.2, 1.4 Hz, 1H), 3.01 – 2.68 (m, 9H), 2.32 – 2.25 (m, 2H), 2.16 – 2.09 (m, 1H), 2.02 – 1.78 (m, 4H), 1.37 (d, *J* = 1.4 Hz, 18H).

^{13}C{^{1}H} NMR ($CDCl_3$, 126 MHz): δ 149.93, 149.07, 146.11, 146.05, 145.78, 145.47, 145.29, 145.14, 142.39, 142.28, 141.50, 128.62, 128.44, 128.13, 127.98, 126.25, 126.23, 126.11, 126.05, 125.88, 125.46, 125.42, 124.75, 124.68, 119.33, 118.71, 87.40, 85.36, 74.01, 73.61, 34.35, 34.32, 31.76, 31.66, 31.59, 31.14, 30.44, 30.14, 29.85, 25.74.

HRMS (ESI+): calcd for [M, $C_{28}H_{32}NO]^+$ 398.2484, found 398.2481.

IR (Neat): 3024.6, 2944.8, 1618.5, 1506.1,1478.1, 1361.8 cm^{-1}

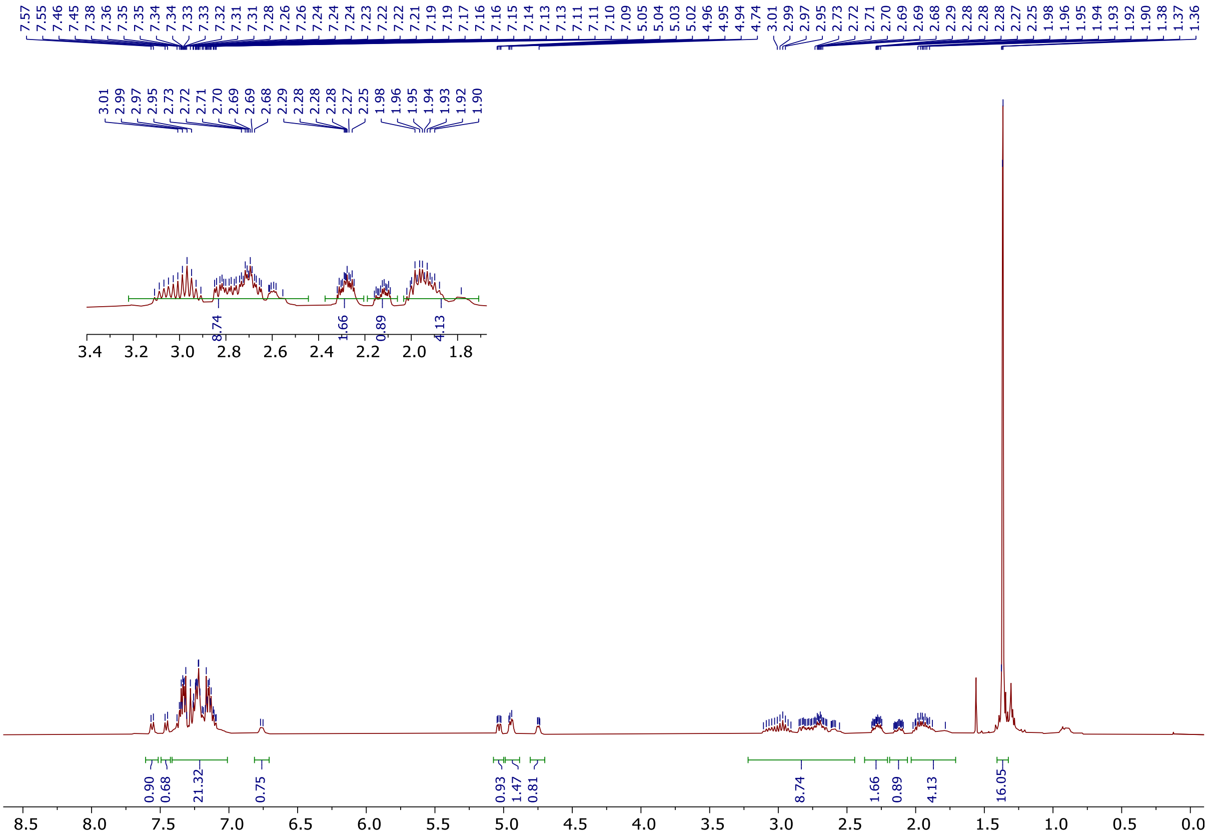

Figure S38: ^{1}H NMR Spectrum of **4** in $CDCl_3$ after isolation via column chromatography.

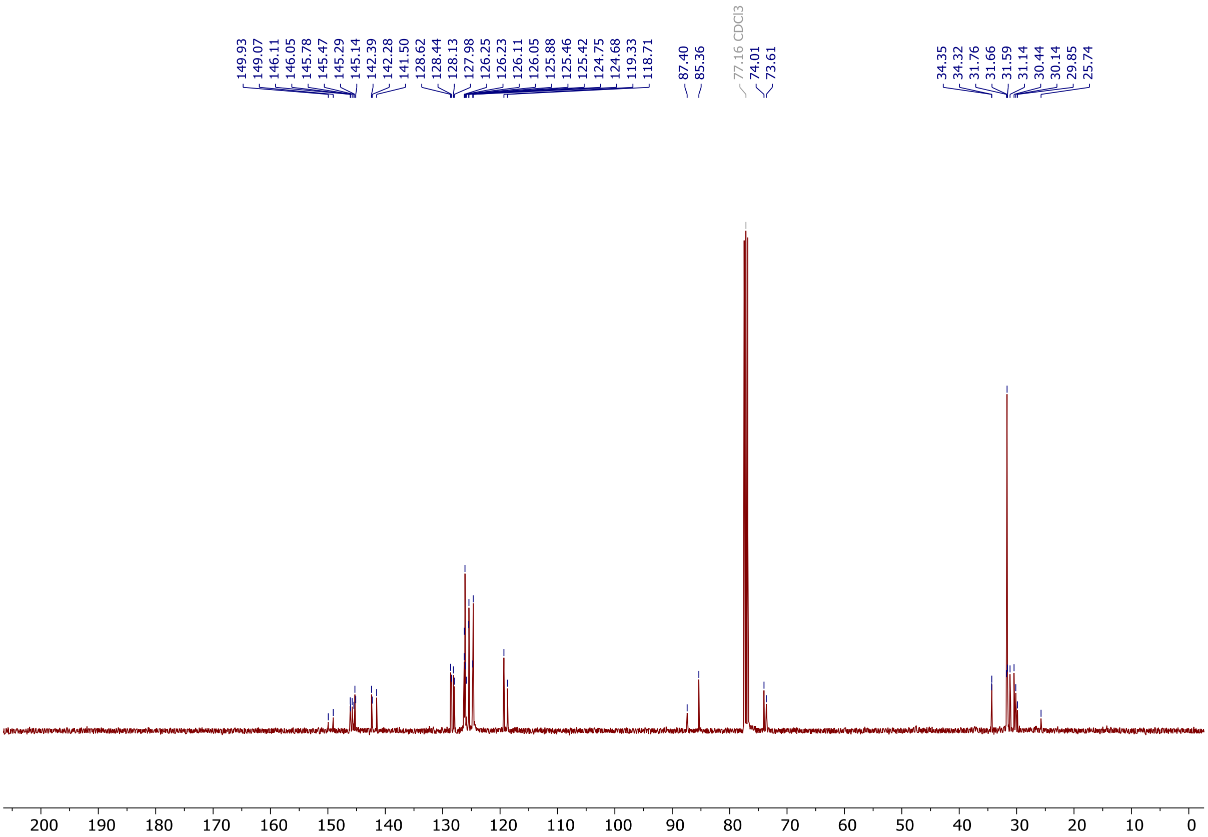

Figure S39: ^{13}C NMR Spectrum of **4** in $CDCl_3$ after isolation via column chromatography.

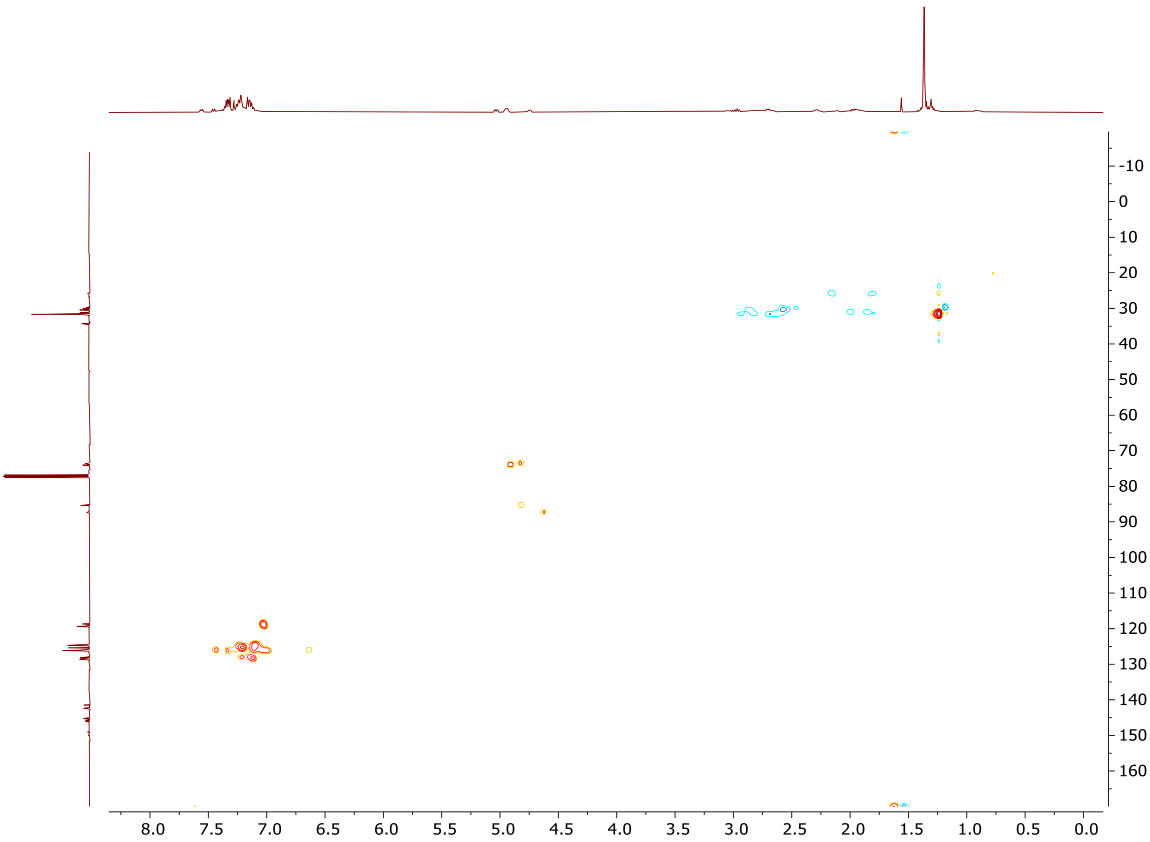

Figure S40: ^{1}H-^{13}C HSQC NMR Spectrum of **4** in $CDCl_3$ after isolation via column chromatography.

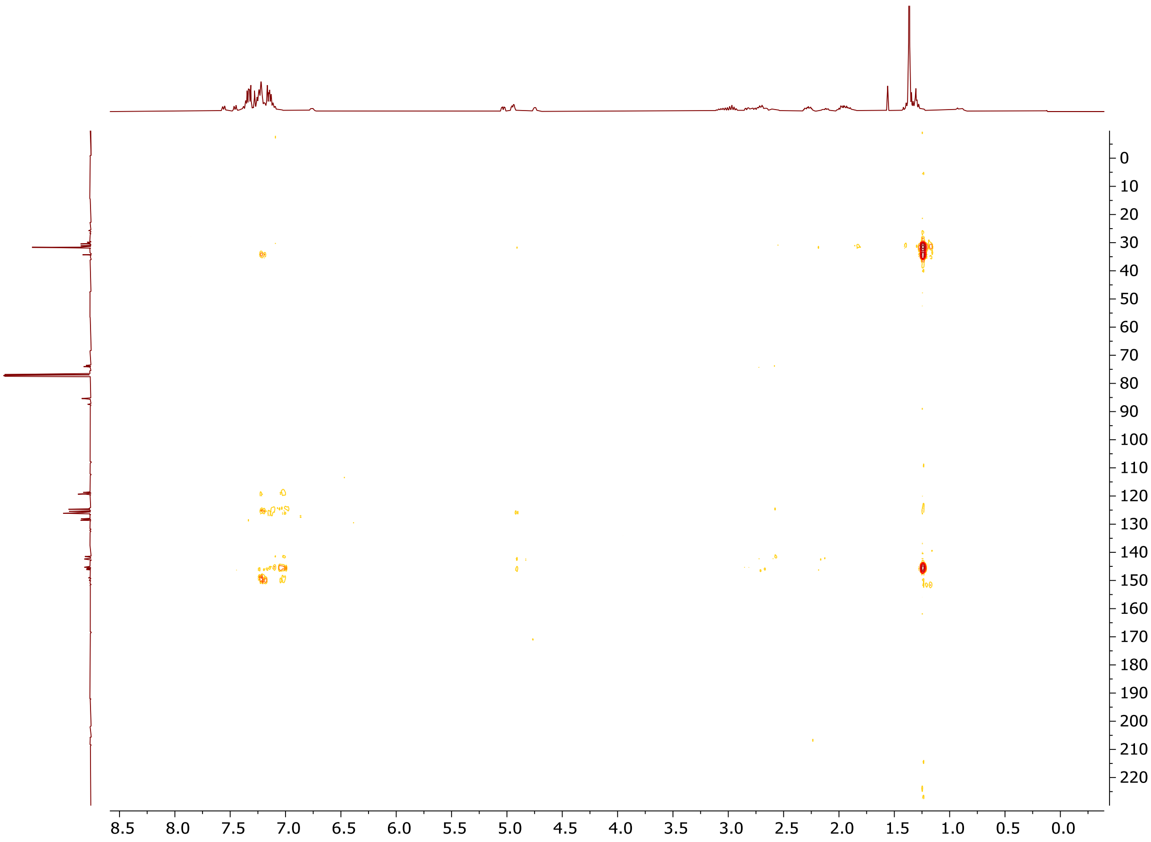

Figure S41: ^{1}H-^{13}C HMBC NMR Spectrum of **4** in $CDCl_3$ after isolation via column chromatography.

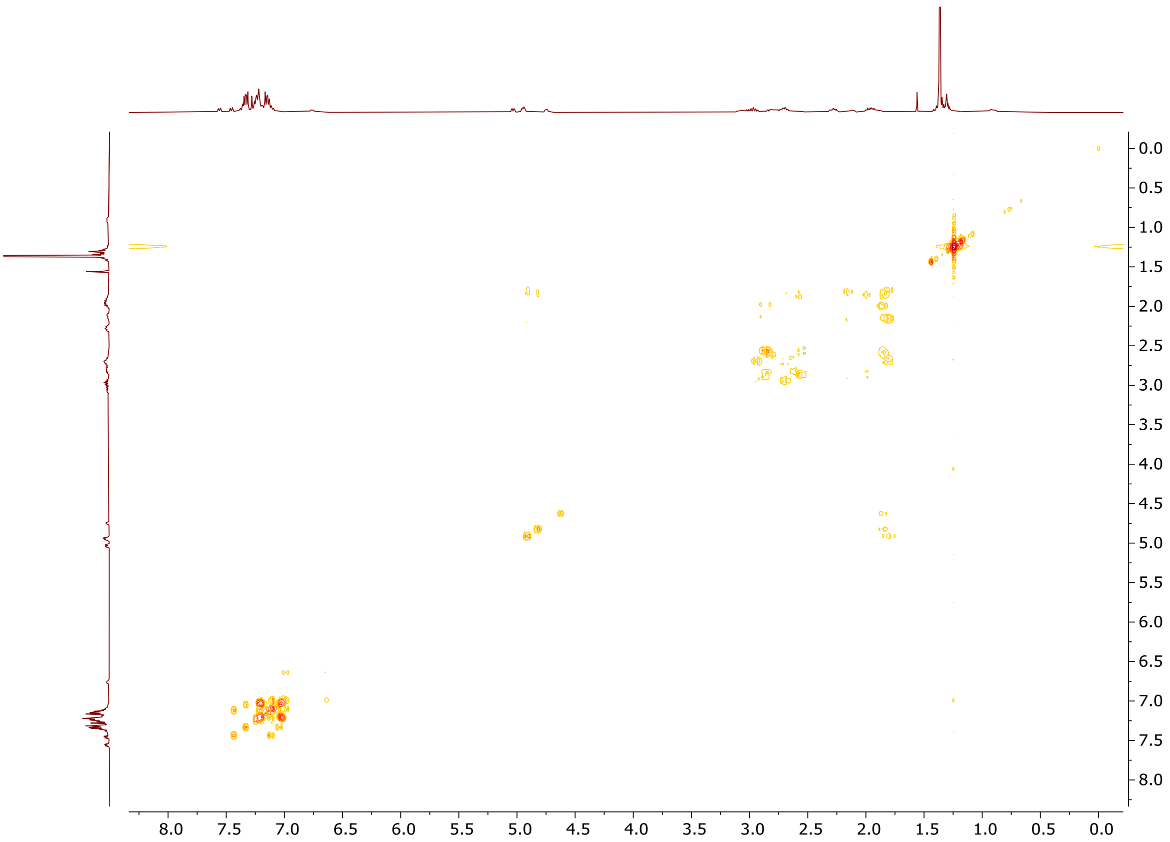

Figure S42: ^{1}H-^{1}H COSY NMR Spectrum of **4** in $CDCl_3$ after isolation via column chromatography.

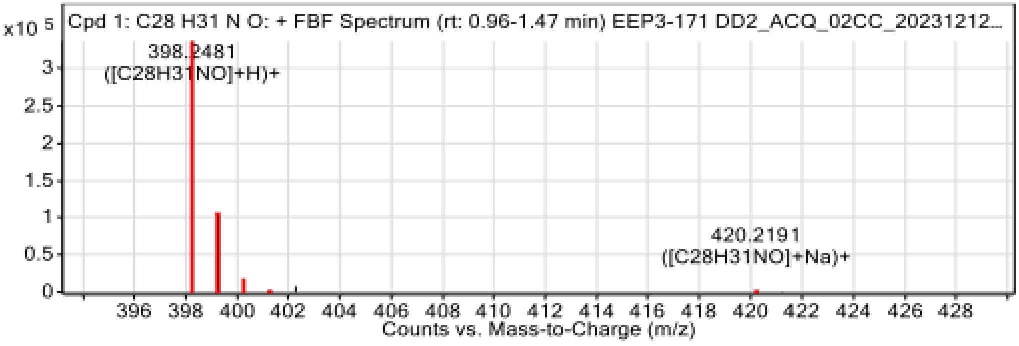

Figure S43: HRMS spectra for compound **4**.

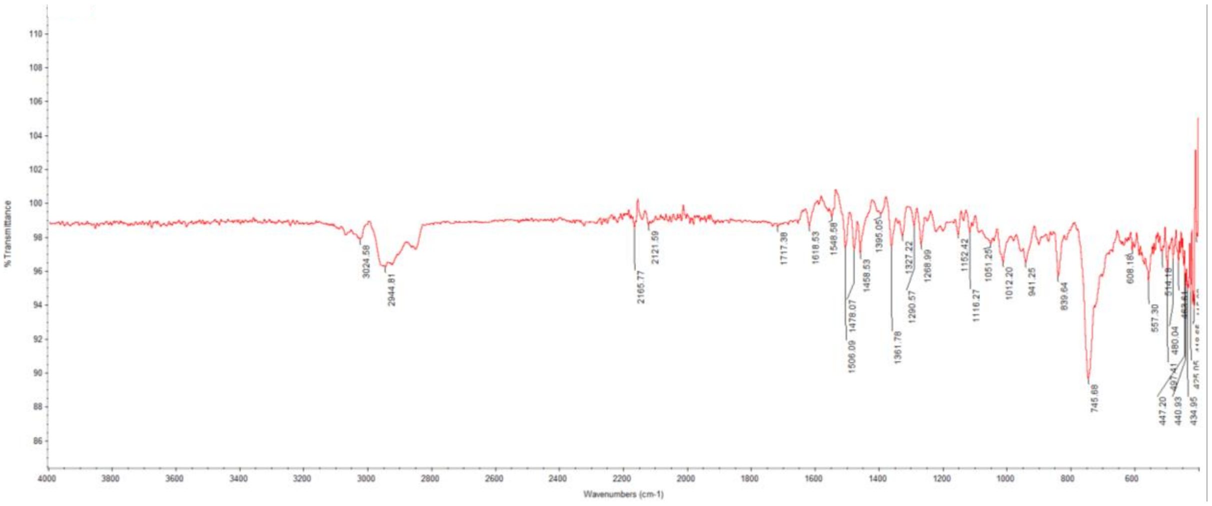

Figure S44: IR spectra for compound **4**.

3.1.3. N-(4-(tert-butyl)phenyl)-N-(2,3-dihydro-1H-inden-1-yl)-2,3-dihydro-1H-inden-1-amine **5**

Mixture of Diastereoisomers 1:0.9

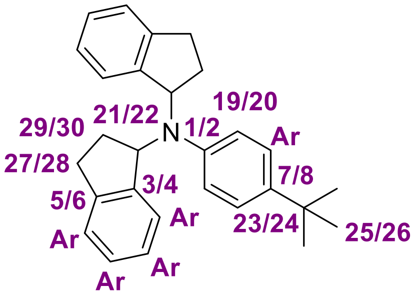

Light yellow oil

R_f = 0.66 (30% DCM / 70% hexane)

^{1}H NMR ($CDCl_3$, 400 MHz): δ 7.53 – 7.48 (m, 2H, Ar-H), 7.30 – 7.16 (m, 14H, Ar-H), 7.12 – 7.05 (m, 4H, Ar-H), 6.61 (app. dd, J = 8.9, 7.4 Hz, 4H, $C^{19/20}$-H), 5.21 (t, J = 8.7 Hz, 2H, $C^{21/22}$-H), 5.14 (t, J = 8.1 Hz, 2H, $C^{21/22}$-H), 2.99 (app. dddd, J = 16.2, 12.1, 9.5, 2.9 Hz, 4H, $C^{29/30}$-H), 2.84 (app. ddt, J = 24.4, 15.8, 8.8 Hz, 4H, $C^{29/30}$-H), 2.46 – 2.18 (m, 8H, $C^{27/28}$-H), 1.24 (app. d, J = 2.4 Hz, 18H, $C^{25/26}$-H).

^{13}C{^{1}H} NMR ($CDCl_3$, 126 MHz): δ 145.9 (C^1), 145.7 (C^2), 145.0 (C^3), 144.9 (C^4), 142.9 (C^5), 142.6 (C^6), 140.2 (C^7), 139.8 (C^8), 127.4 (Ar), 127.3 (Ar), 126.8 (Ar), 126.5 (Ar), 125.6 (Ar), 125.5 (Ar), 125.2 (Ar), 125.1 (Ar), 124.7 (Ar), 124.4 (Ar), 116.5 (C^{19}), 116.0 (C^{20}), 63.7 (C^{21}), 63.5 (C^{22}), 33.9 (C^{23}), 33.9 (C^{24}), 31.6 (C^{25}), 31.6 (C^{26}), 30.8 (C^{27}), 30.4 (C^{28}), 29.9 (C^{29}), 29.7 (C^{30}).

HRMS (ESI+): calcd for [M, $C_{28}H_{32}N]^+$ 382.2535, found 382.2530.

IR (Neat): 2956.6, 2850.6, 1610.8, 1515.6, 1362.6, 1253.4 cm^{-1}

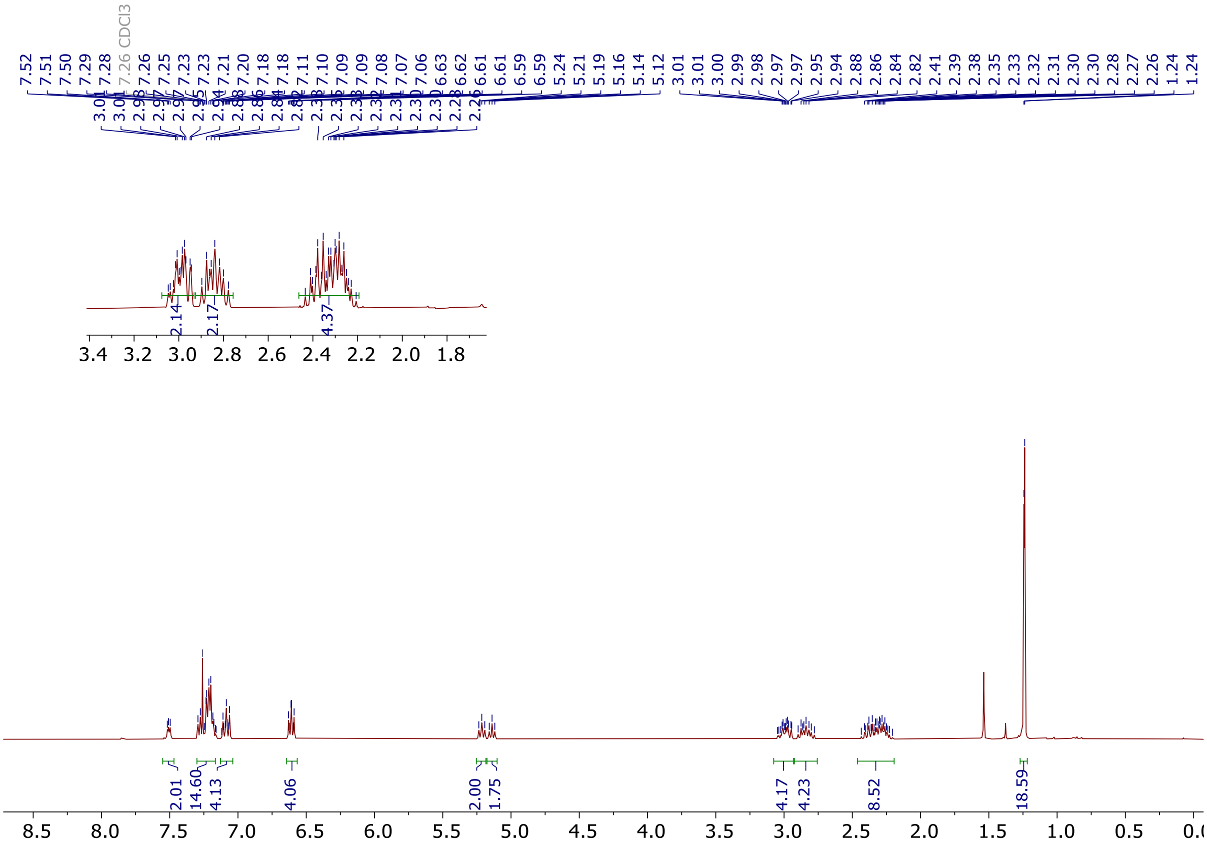

Figure S45: ^{1}H NMR Spectrum of **5** in $CDCl_3$ after isolation via column chromatography.

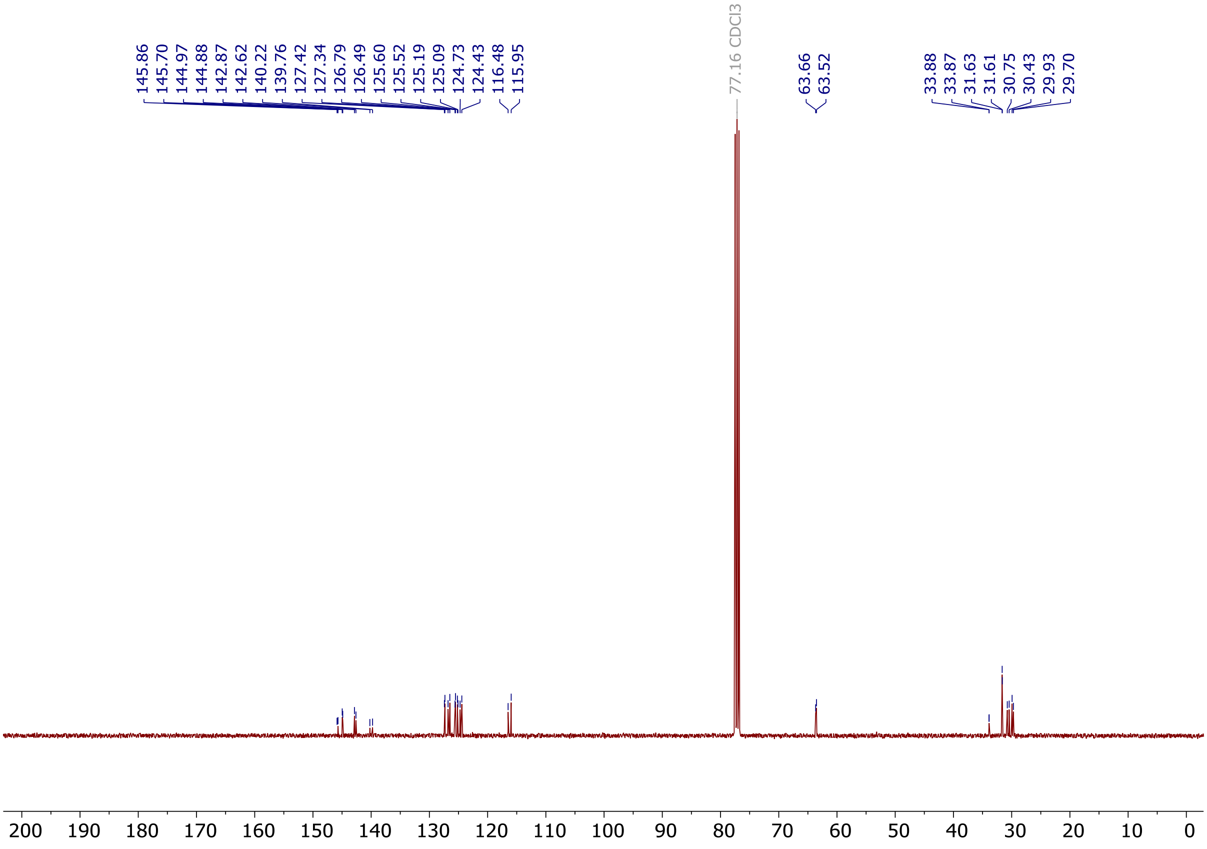

Figure S46: ^{13}C NMR Spectrum of **5** in $CDCl_3$ after isolation via column chromatography.

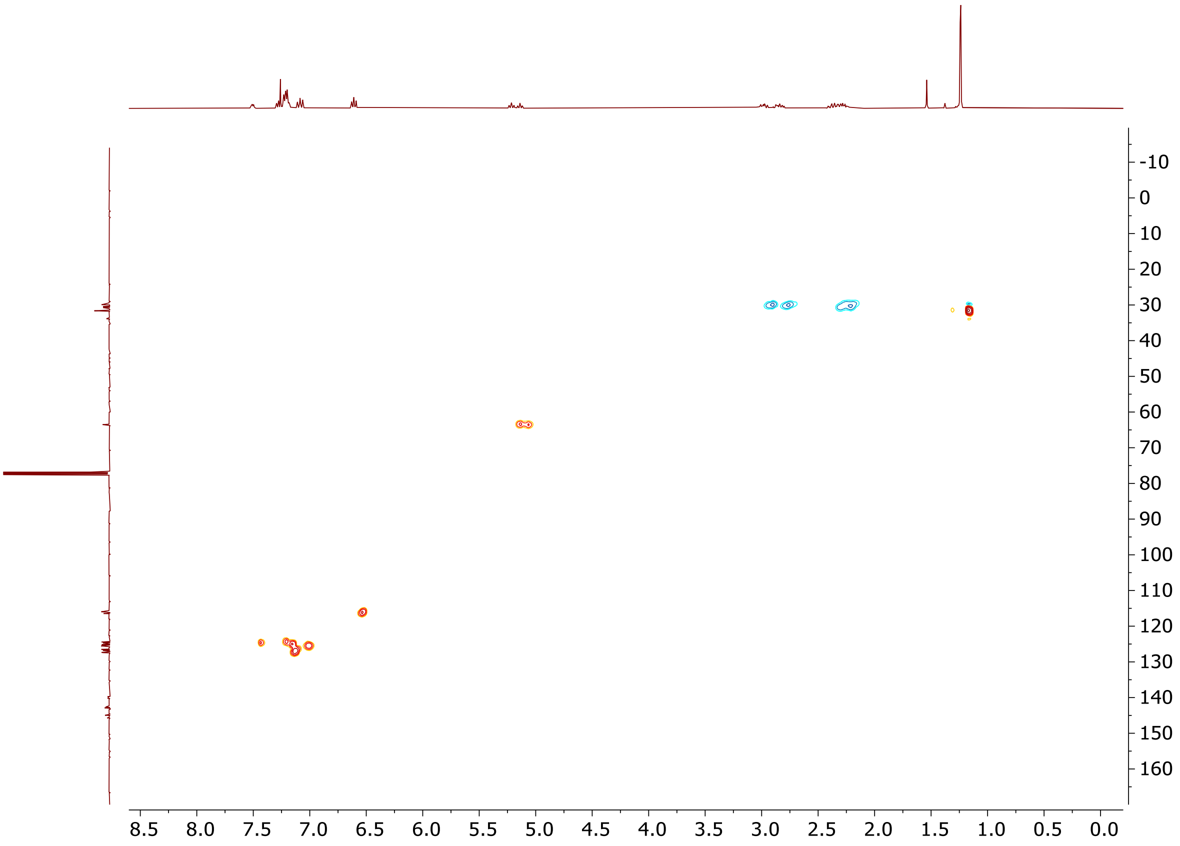

Figure S47: ^{1}H-^{13}C HSQC NMR Spectrum of **5** in $CDCl_3$ after isolation via column chromatography.

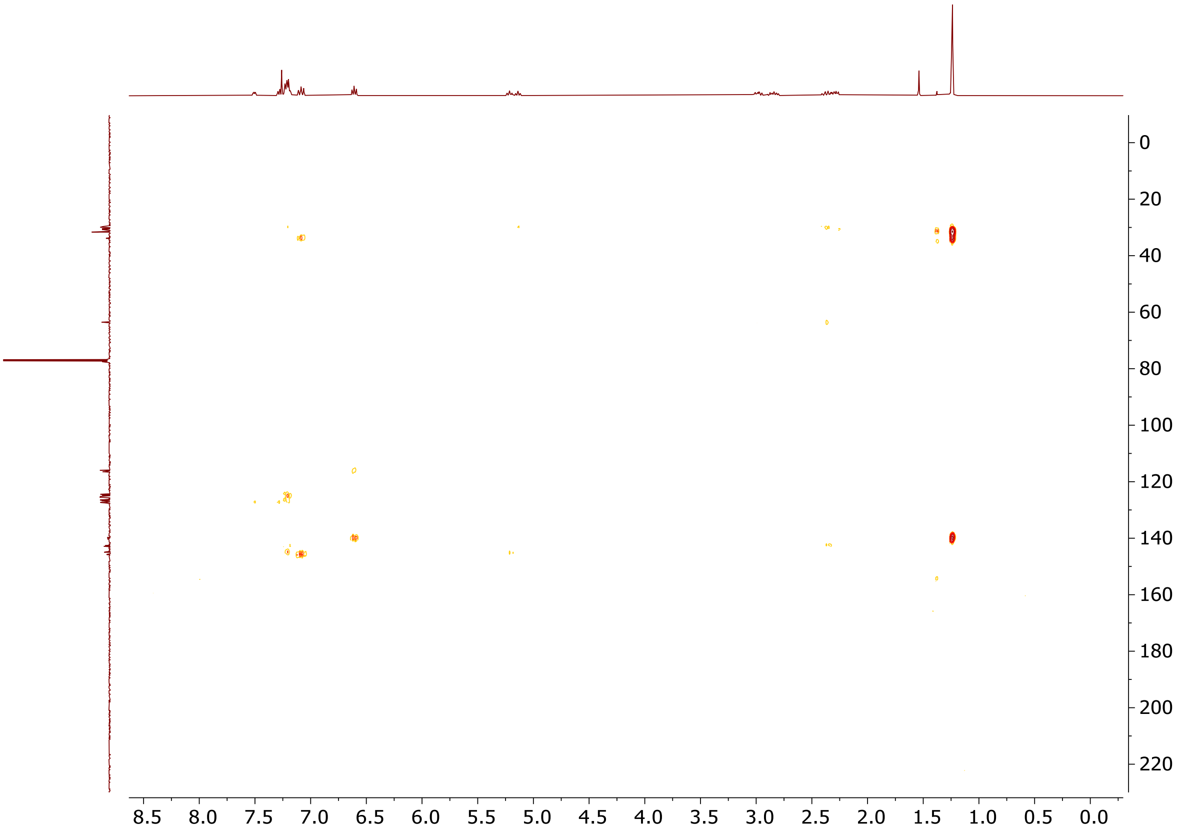

Figure S48: ^{1}H-^{13}C HMBC NMR Spectrum of **5** in $CDCl_3$ after isolation via column chromatography.

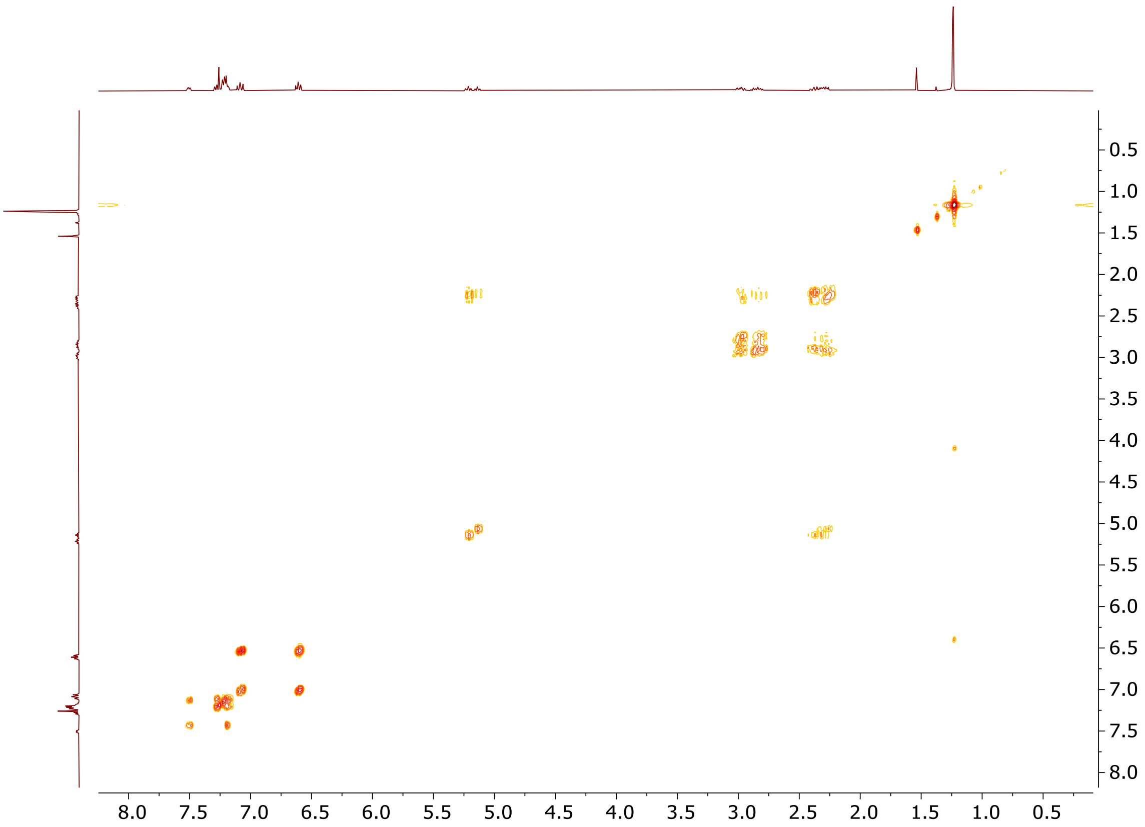

Figure S49: ^{1}H-^{1}H COSY NMR Spectrum of **5** in $CDCl_3$ after isolation via column chromatography.

Figure: Full range view of Compound spectra and potential adducts.

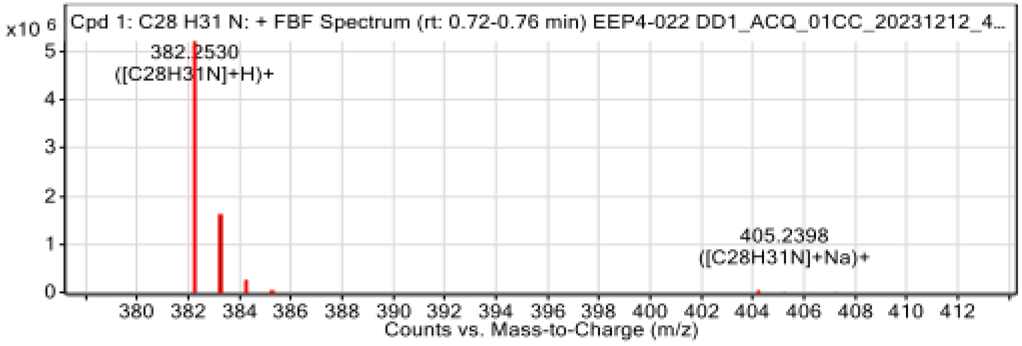

Figure S50: HRMS spectra for compound **5**.

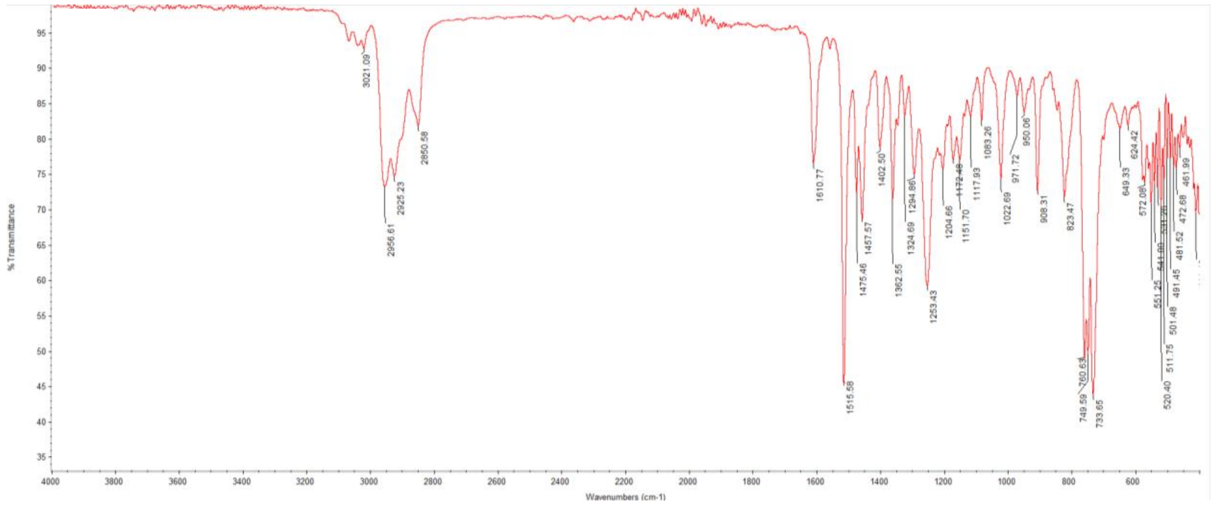

Figure S51: IR spectra for compound **5**.

3.1.4. 4-(tert-butyl)aniline **6**

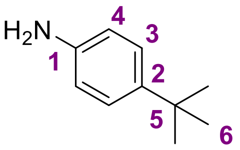

Light brown oil

R_f = 0.27 (50% DCM / 50% hexane)

^{1}H NMR ($CDCl_3$, 500 MHz): δ 7.23 (d, *J* = 8.5 Hz, 2H, C^3-H), 6.68 (d, *J* = 8.5 Hz, 2H, C^4-H), 3.58 (s, 2H, N-H), 1.33 (s, 9H, C^6-H).

^{13}C{^{1}H} NMR ($CDCl_3$, 126 MHz): δ 143.9 (C^1), 141.5 (C^2), 126.1 (C^3), 115.0 (C^4), 34.0 (C^5), 31.6 (C^6).

HRMS (ESI+): calcd for $[M, C_{10}H_{16}N]^+$ 150.1277, found 150.1277.

IR (Neat): 2957.7, 1618.2, 1514.9, 1264.6, 824.5 cm^{-1}.

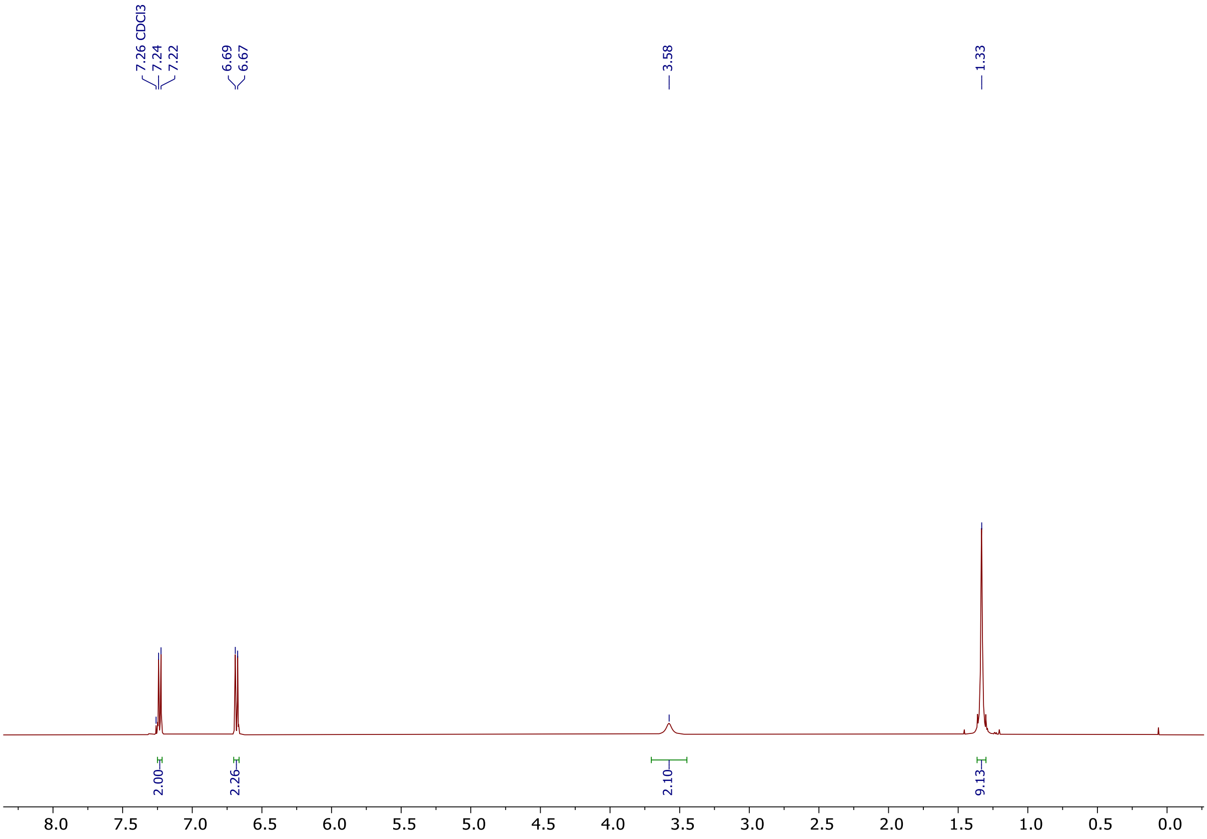

Figure S52: ^{1}H NMR Spectrum of **6** in $CDCl_3$ after isolation via column chromatography.

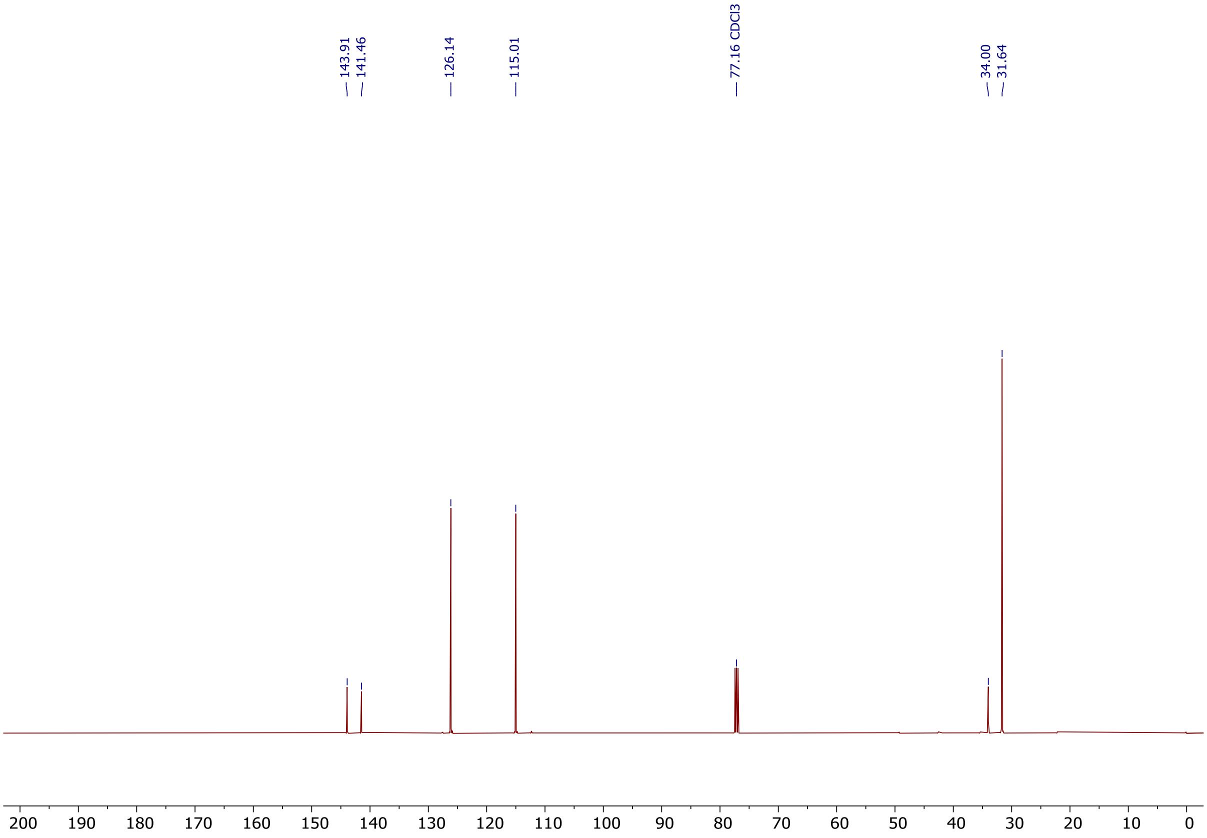

Figure S53: ^{13}C NMR Spectrum of **6** in $CDCl_3$ after isolation via column chromatography.

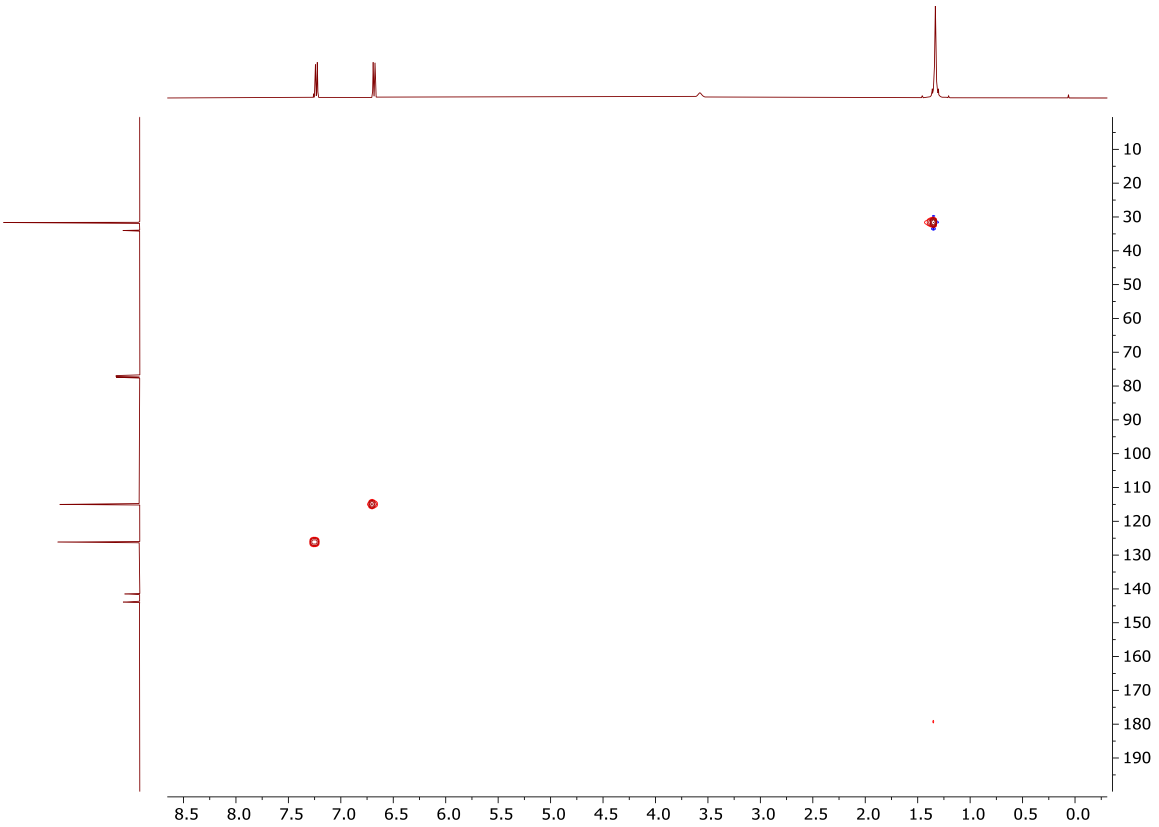

Figure S54: ^{1}H-^{13}C HSQC NMR Spectrum of **6** in $CDCl_3$ after isolation via column chromatography.

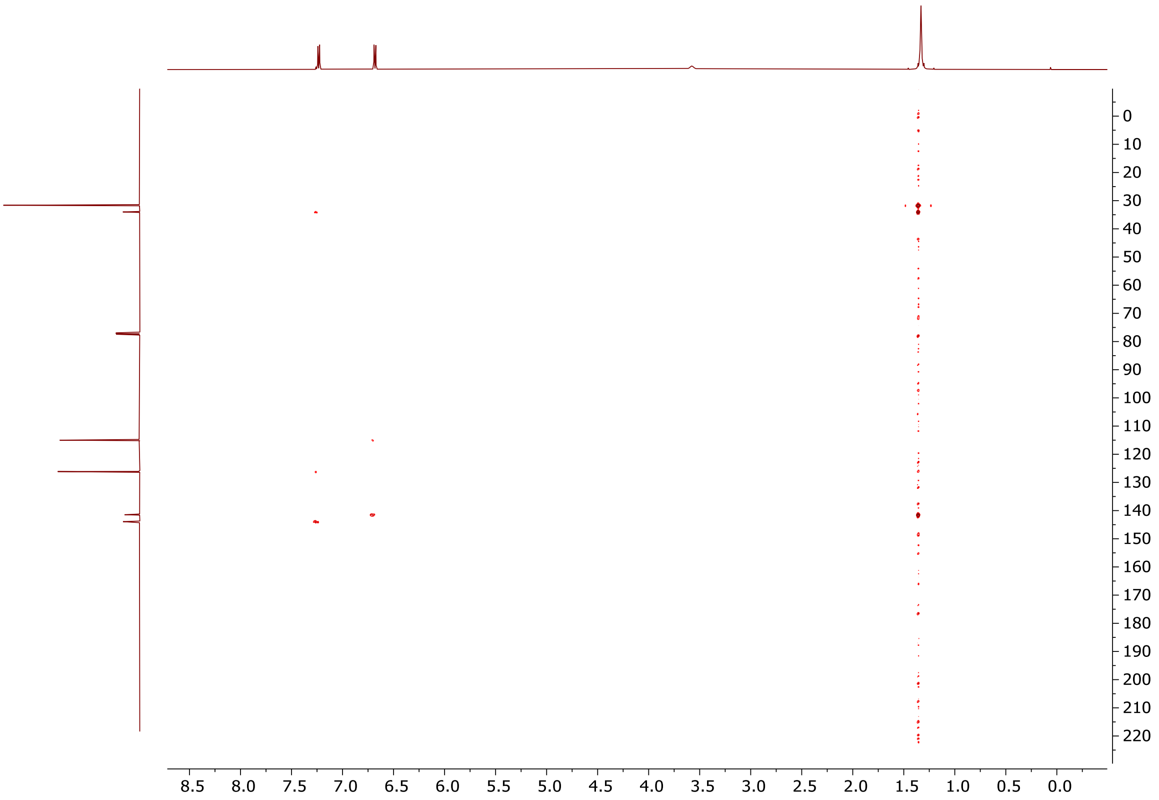

Figure S55: ^{1}H-^{13}C HMBC NMR Spectrum of **6** in $CDCl_3$ after isolation via column chromatography.

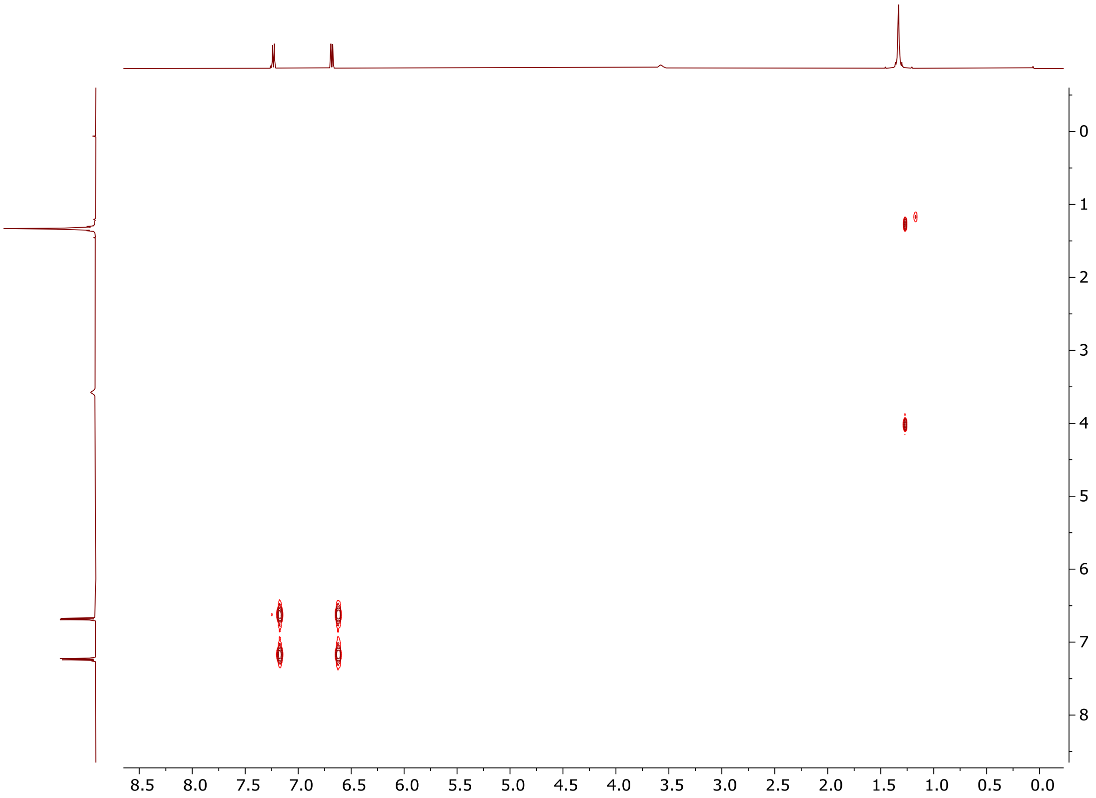

Figure S56: ^{1}H-^{1}H COSY NMR Spectrum of **6** in $CDCl_3$ after isolation via column chromatography.

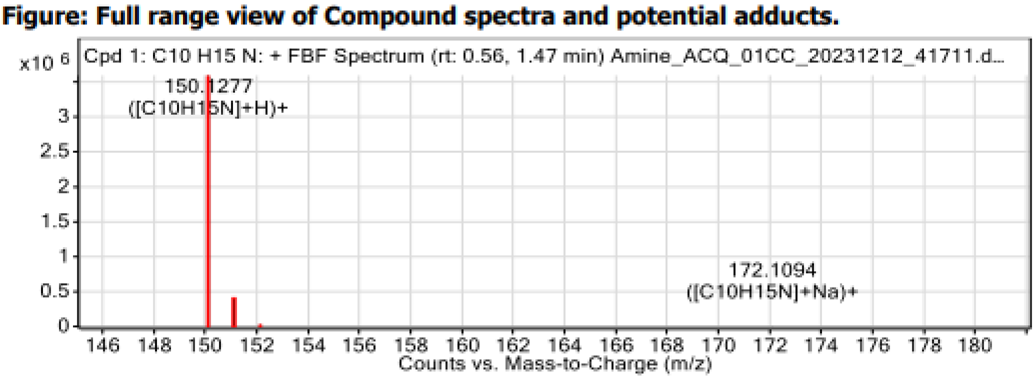

Figure S57: HRMS spectra for compound **6**.

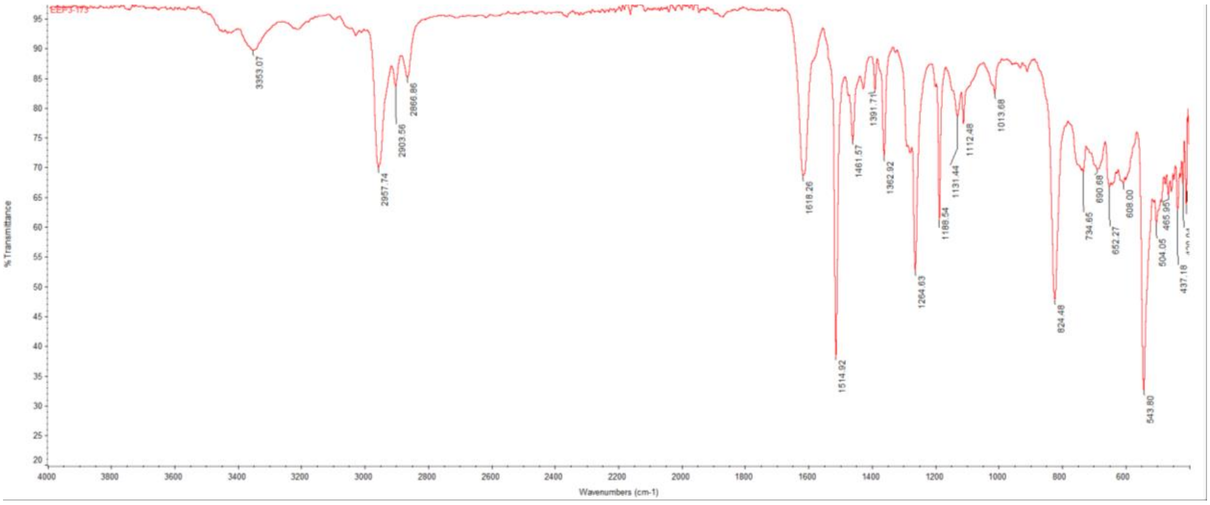

Figure S58: IR spectra for compound **6**.

3.1.5. 4-(tert-butyl)-N-(2-phenylpropan-2-yl)aniline **7**

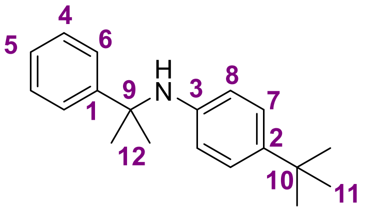

White oil.

R_f = 0.28 (30% DCM / 70% hexane)

Analytic data is in accordance with those reported in literature.

^{1}H NMR ($CDCl_3$, 500 MHz): δ 7.60 (d, J = 7.9 Hz, 2H, C^6-H), 7.39 (app. t, J = 7.6 Hz, 2H, C^4-H), 7.29 (app. t, J = 7.3 Hz, 1H, C^5-H), 7.09 (d, J = 8.4 Hz, 2H, C^7-H), 6.35 (d, J = 8.4 Hz, 2H, C^8-H), 3.98 (*br* s, 1H, NH), 1.69 (s, 6H, C^{12}-H), 1.29 (s, 9H, C^{11}-H).

^{13}C{^{1}H} NMR ($CDCl_3$, 126 MHz): δ 147.9 (C^1), 143.8 (C^3), 139.8 (C^2), 128.6 (C^4), 126.4 (C^5), 125.8 (C^6), 125.6 (C^7), 115.2 (C^8), 55.9 (C^9), 33.9 (C^{10}), 31.6 (C^{11}), 30.9 (C^{12}).

HRMS (ESI+): calcd for $[M, C_{19}H_{25}N]^+$ 268.2060, found 268.2063.

IR (Neat): 3411, 2960, 1893, 1617, 1517, 814, 697 cm^{-1}.

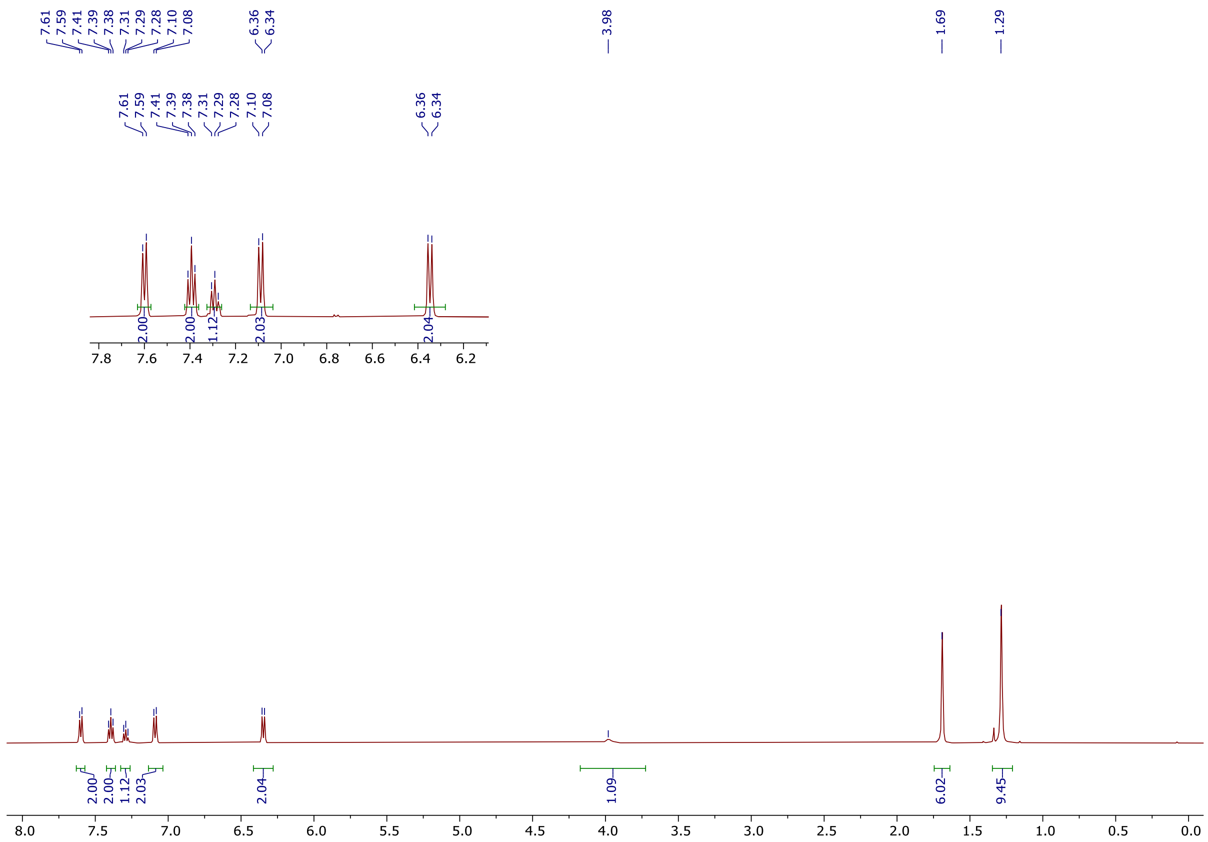

Figure S59: ^{1}H NMR Spectrum of **7** in $CDCl_3$ after isolation via column chromatography.

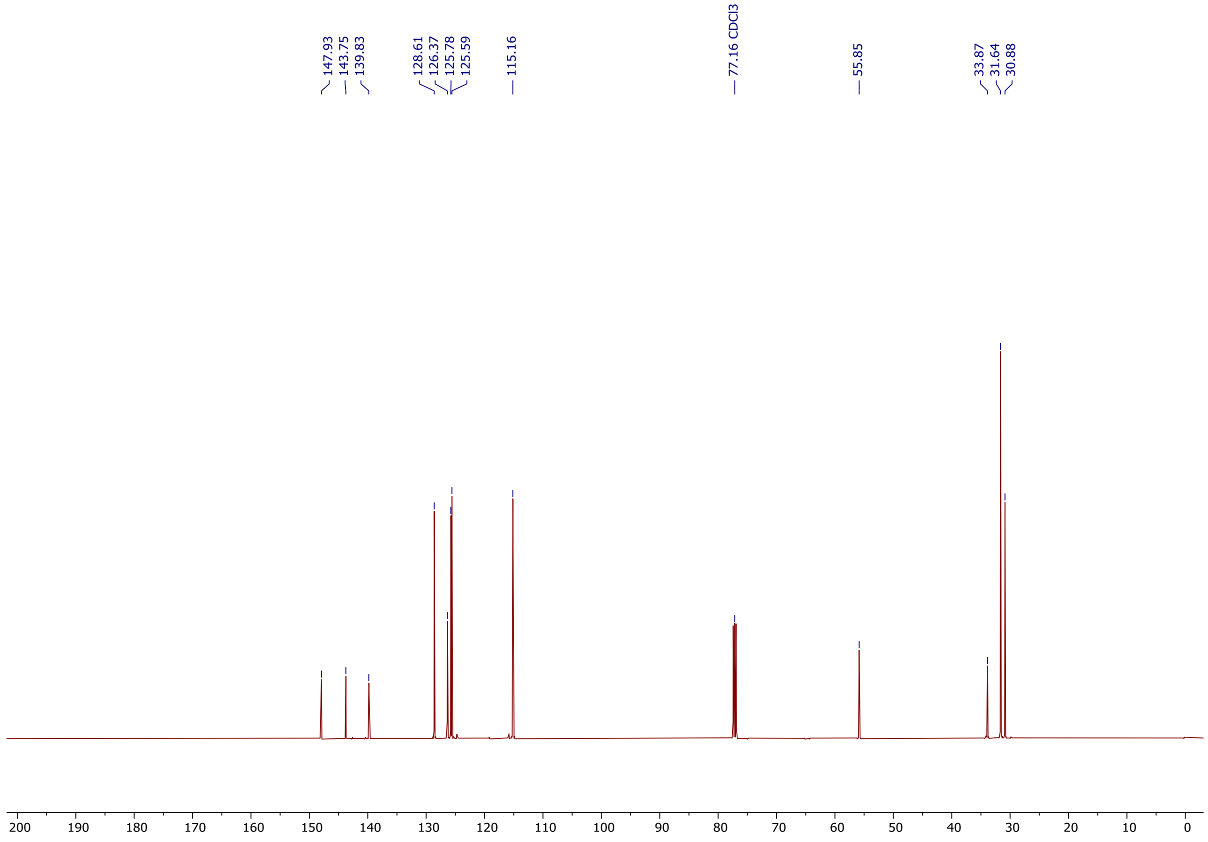

Figure S60: ^{13}C NMR Spectrum of **7** in $CDCl_3$ after isolation via column chromatography.

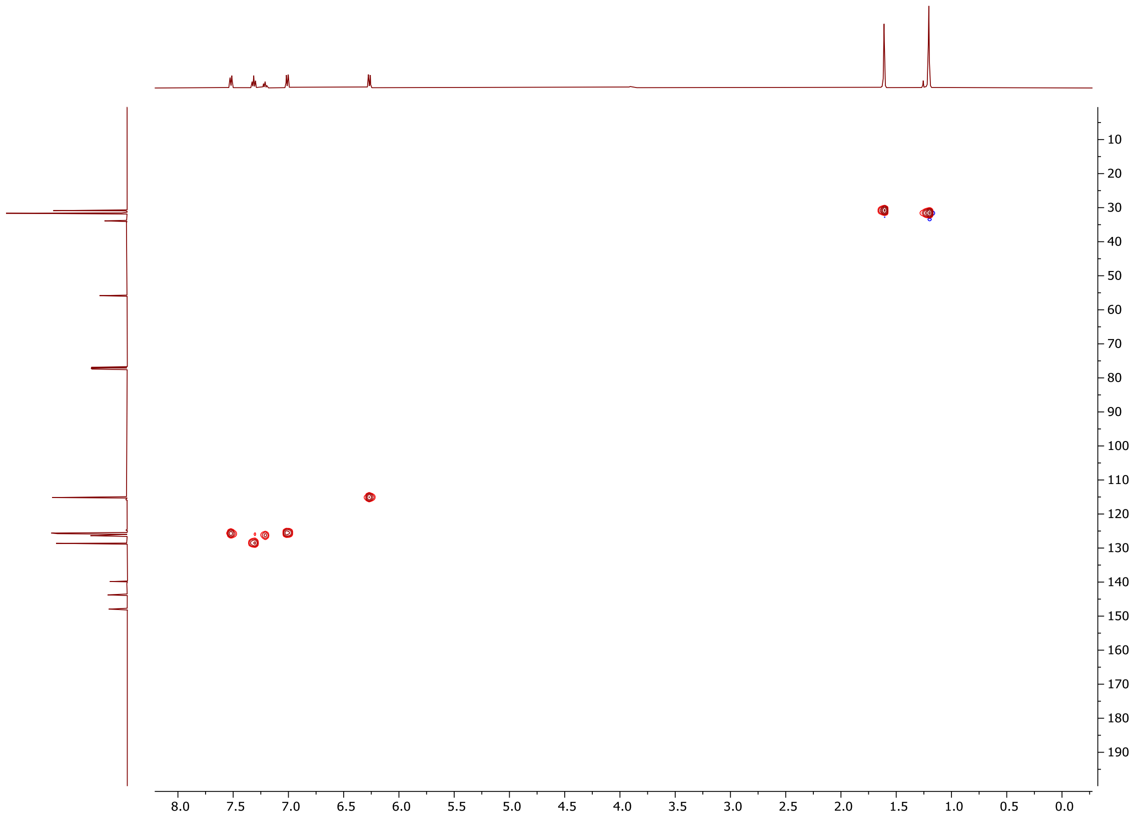

Figure S61: ^{1}H-^{13}C HSQC NMR Spectrum of **7** in $CDCl_3$ after isolation via column chromatography.

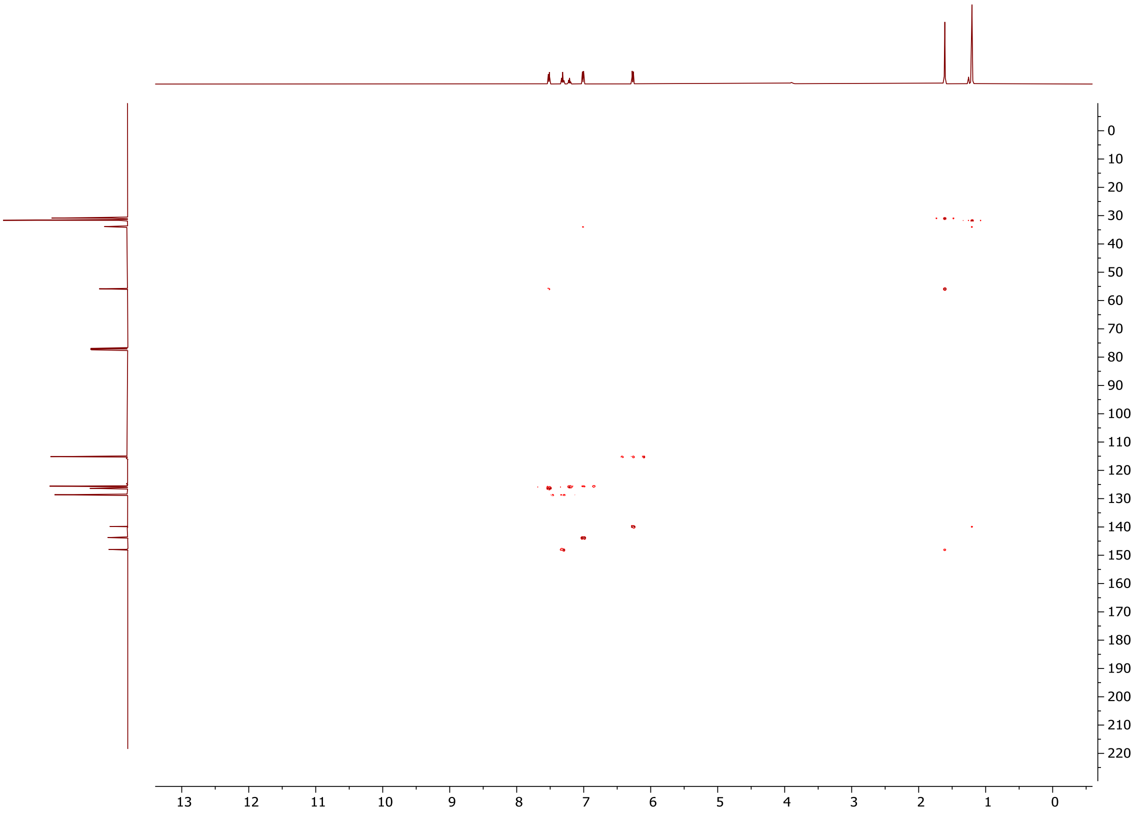

Figure S62: ^{1}H-^{13}C HMBC NMR Spectrum of **7** in $CDCl_3$ after isolation via column chromatography.

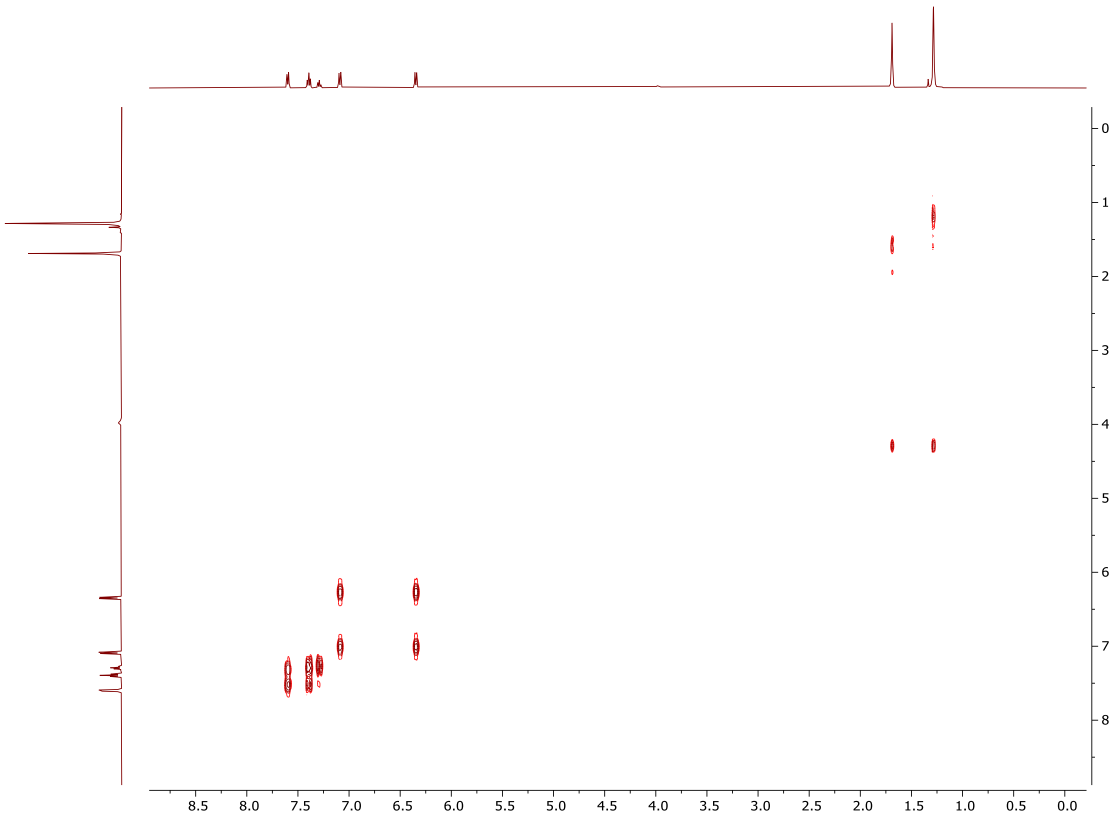

Figure S63: ^{1}H-^{1}H COSY NMR Spectrum of **7** in $CDCl_3$ after isolation via column chromatography.

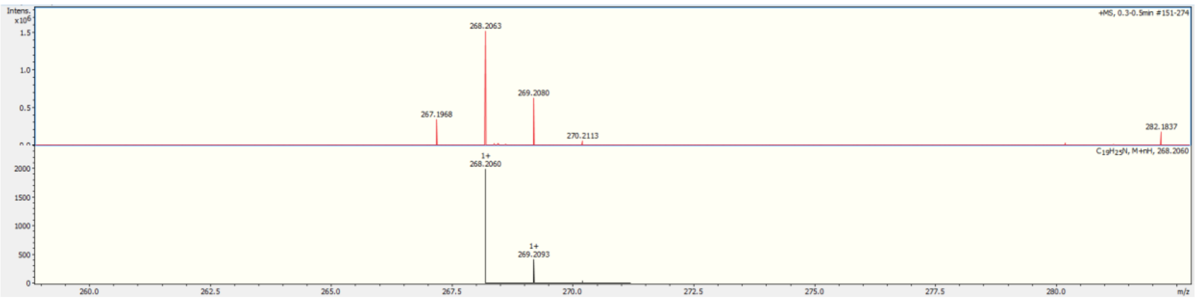

Figure S64: HRMS spectra for compound **7**.

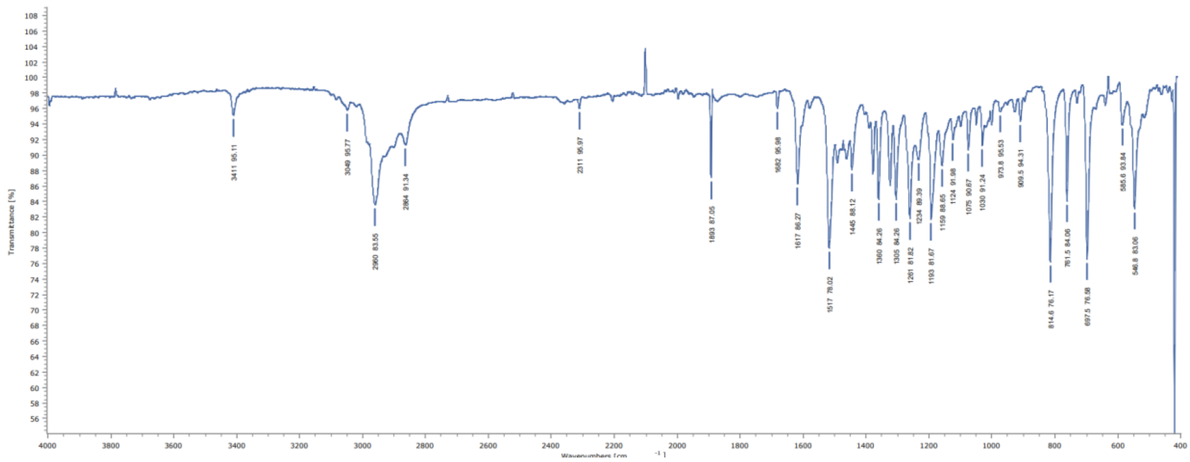

Figure S65: IR spectra for compound **7**.

3.1.6. 4-(tert-butyl)-N-(4-methyl-2,4-diphenylpentan-2-yl)aniline **7’**

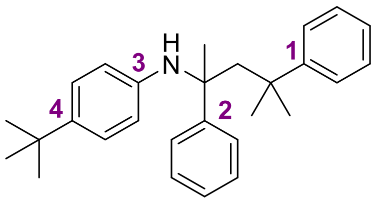

Colourless oil

R_f = 0.51 (20% DCM / 80% hexane)

^{1}H NMR ($CDCl_3$, 400 MHz): δ 7.48 – 7.36 (m, 6H), 7.33 – 7.27 (m, 3H), 7.20 (t, *J* = 7.2 Hz, 1H), 6.92 – 6.84 (m, 2H), 5.76 (d, *J* = 8.6 Hz, 2H), 3.72 (s, 1H), 2.31 (d, *J* = 14.7 Hz, 1H), 2.21 (d, *J* = 14.8 Hz, 1H), 1.61 (s, 3H), 1.37 (s, 3H), 1.26 (s, 3H), 1.16 (s, 9H).

^{13}C{^{1}H} NMR ($CDCl_3$, 126 MHz): δ 148.61, 148.33, 143.27, 139.07, 128.72, 128.31, 126.64, 126.45, 126.32, 126.21, 125.28, 115.09, 60.18, 60.08, 38.64, 33.78, 31.61, 31.31, 31.25, 26.10.

HRMS (ESI+): calcd for [M, $C_{28}H_{35}N$]$^+$ 385.2770, found 385.2766.

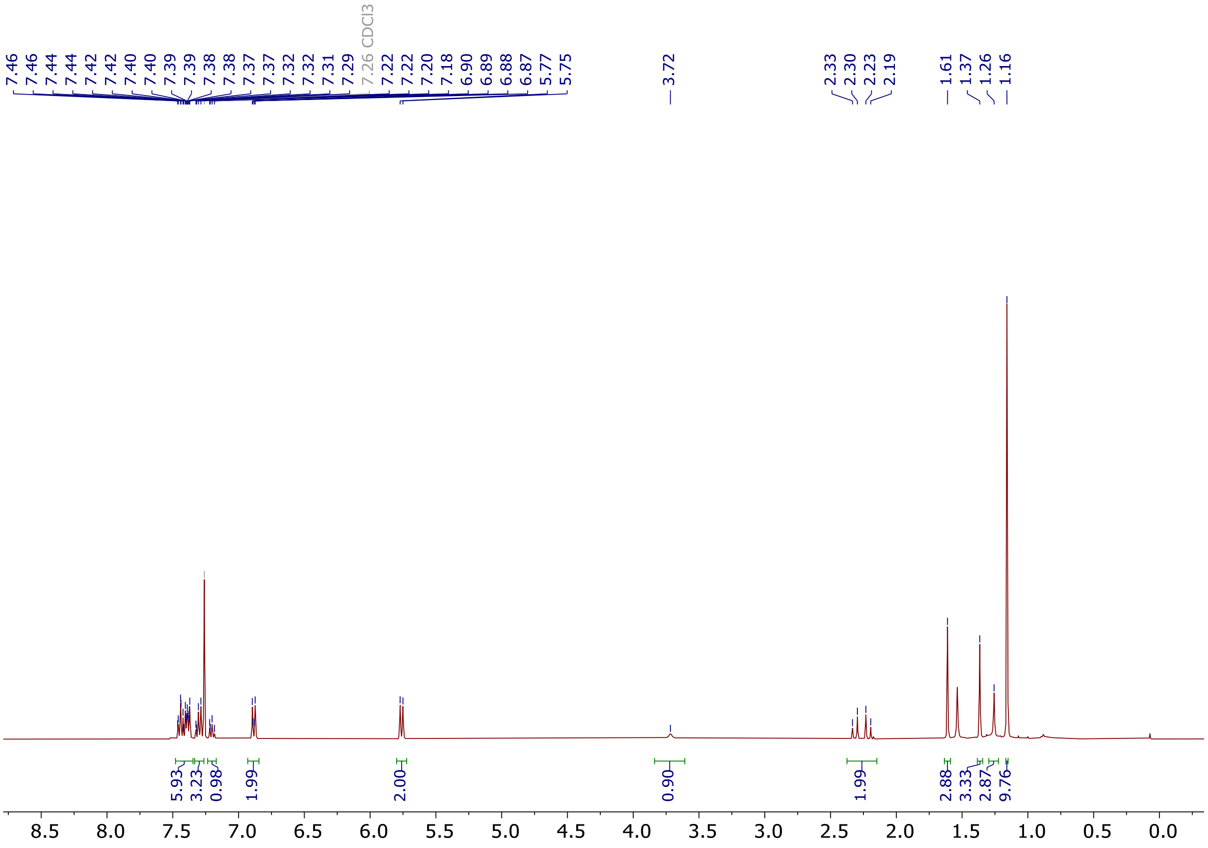

Figure S66: ^{1}H NMR Spectrum of **7’** in $CDCl_3$ after isolation via column chromatography.

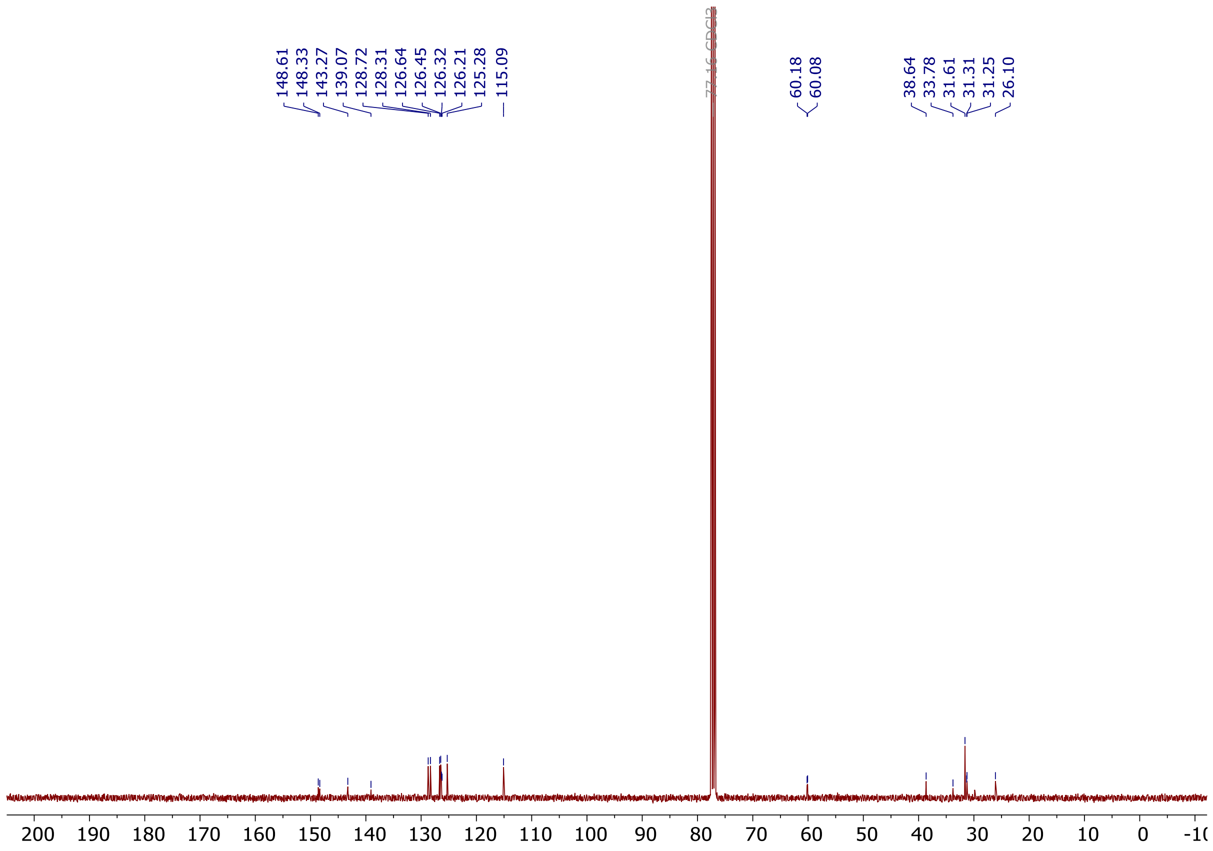

Figure S67: ^{13}C NMR Spectrum of **7’** in $CDCl_3$ after isolation via column chromatography.

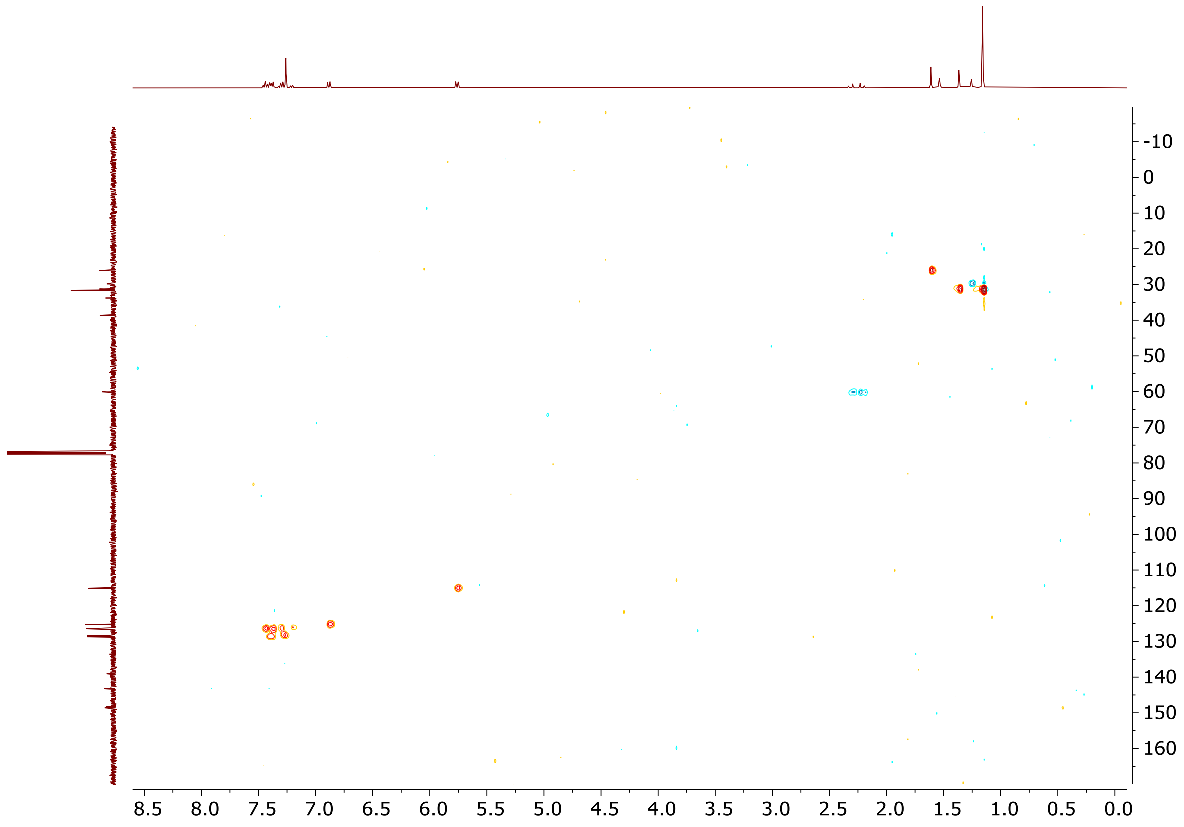

Figure S68: ^{1}H-^{13}C HSQC NMR Spectrum of **7’** in $CDCl_3$ after isolation via column chromatography.

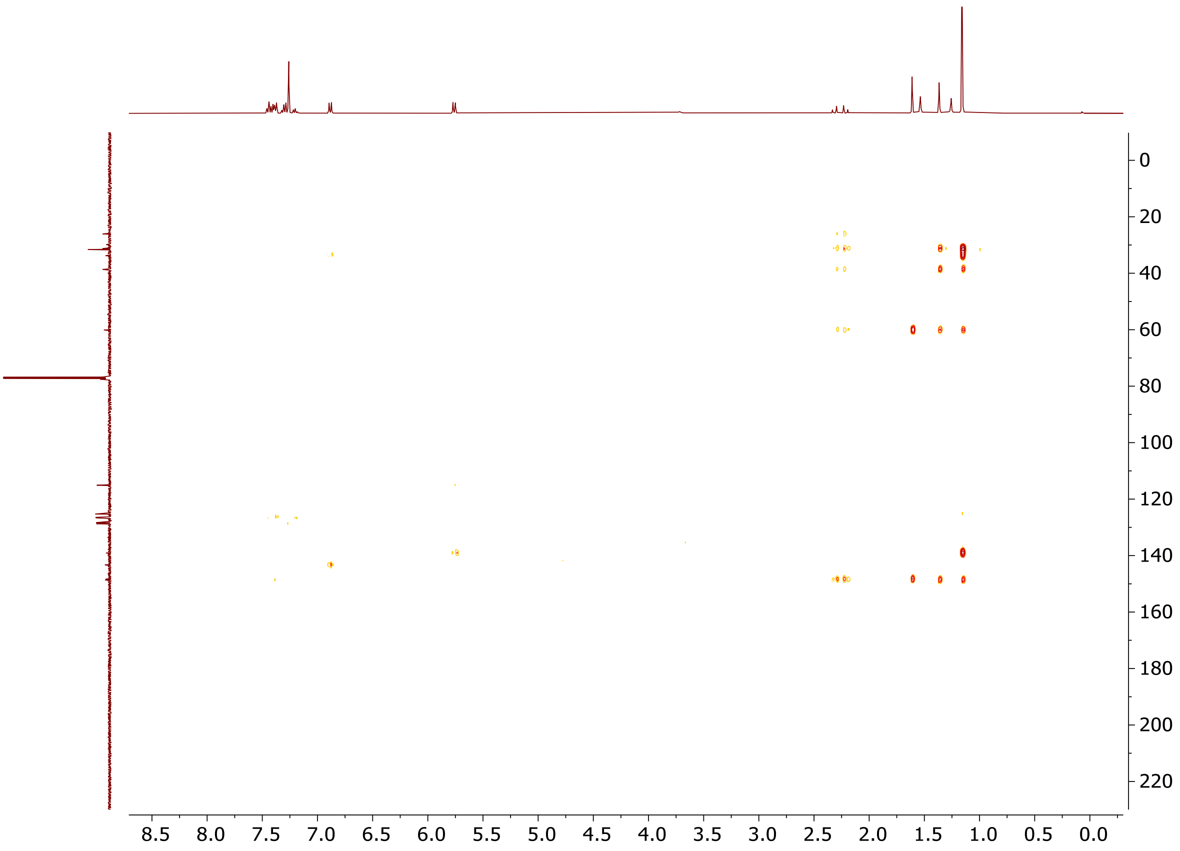

Figure S69: ^{1}H-^{13}C HMBC NMR Spectrum of **7’** in $CDCl_3$ after isolation via column chromatography.

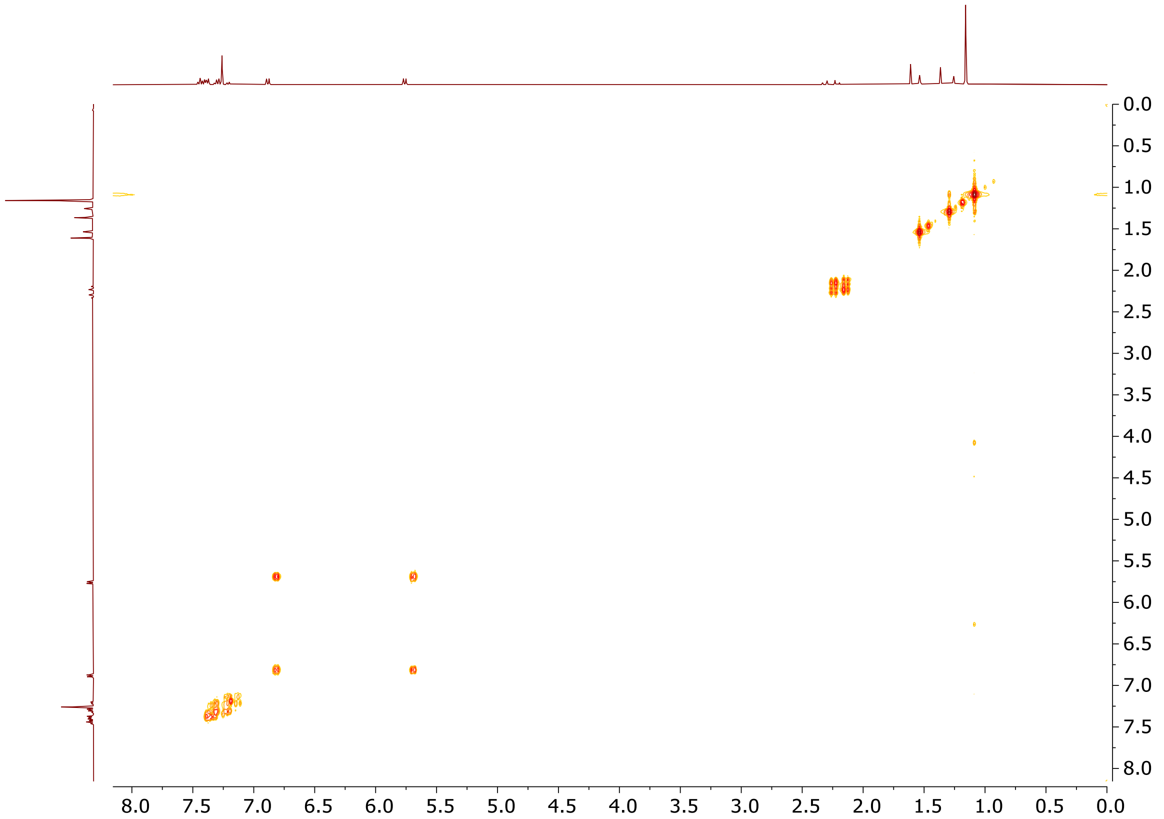

Figure S70: ^{1}H-^{1}H COSY NMR Spectrum of **7’** in $CDCl_3$ after isolation via column chromatography.

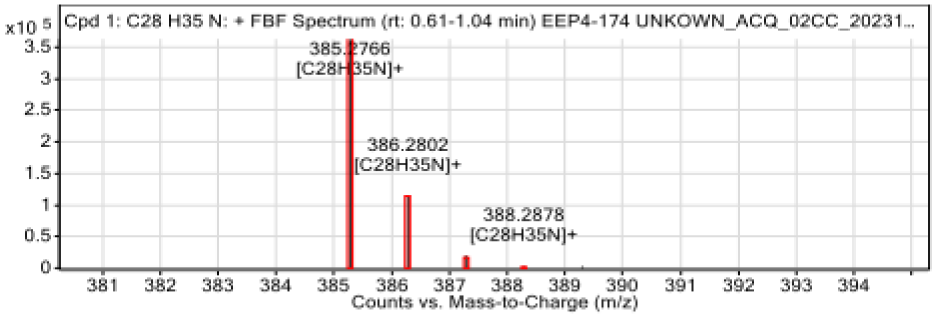

Figure S71: HRMS spectra for compound **7’**.

3.1.7. N-(4-(tert-butyl)phenyl)-N,O-bis(2-phenylpropan-2-yl)hydroxylamine **8**

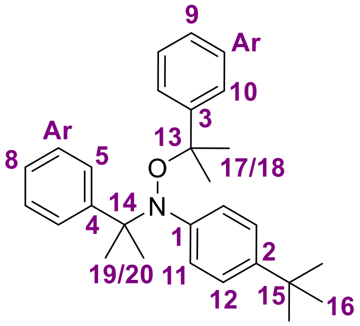

Yellow oil

R_f = 0.64 (30% DCM / 70% hexane)

^{1}H NMR (CDCl$_3$, 500 MHz): δ 7.52 (d, J = 7.6 Hz, 2H, C^5-H), 7.49 (d, J = 7.9 Hz, 2H, C^{10}-H), 7.30 – 7.21 (m, 6H, Ar, Ar, C^8-H, C^9-H), 7.09 (d, J = 8.1 Hz, 2H, C^{12}-H), 6.93 (d, J = 8.1 Hz, 2H, C^{11}-H), 1.62 (s, 3H, $C^{19/20}$-H), 1.48 (app. d, 6H, C^{17-20}), 1.30 (s, 9H, C^{16}-H), 1.25 (2, 3H, $C^{17/18}$-H).

^{13}C{^{1}H} NMR (CDCl$_3$, 126 MHz): δ 149.1 (C^1), 147.2 (C^2). 147.1 (C^3), 146.2 (C^4), 128.2 (C^5), 127.8 (Ar), 127.4 (Ar), 126.8 (C^8), 126.7 (C^9), 125.8 (C^{10}), 125.4 (C^{11}), 123.8 (C^{12}), 81.2 (C^{13}), 65.1 (C^{14}), 34.3 (C^{15}), 31.6 (C^{16}), 28.3 ($C^{17/18}$), 27.4 ($C^{17/28}$), 26.5 ($C^{19/20}$), 25.0 ($C^{19/20}$).

HRMS (ESI+): calcd for [M, $C_{28}H_{35}NO]^+$ 402.2791, found 402.2809.

IR (Neat): 2961, 2865, 1611, 1516, 816, 696 cm^{-1}.

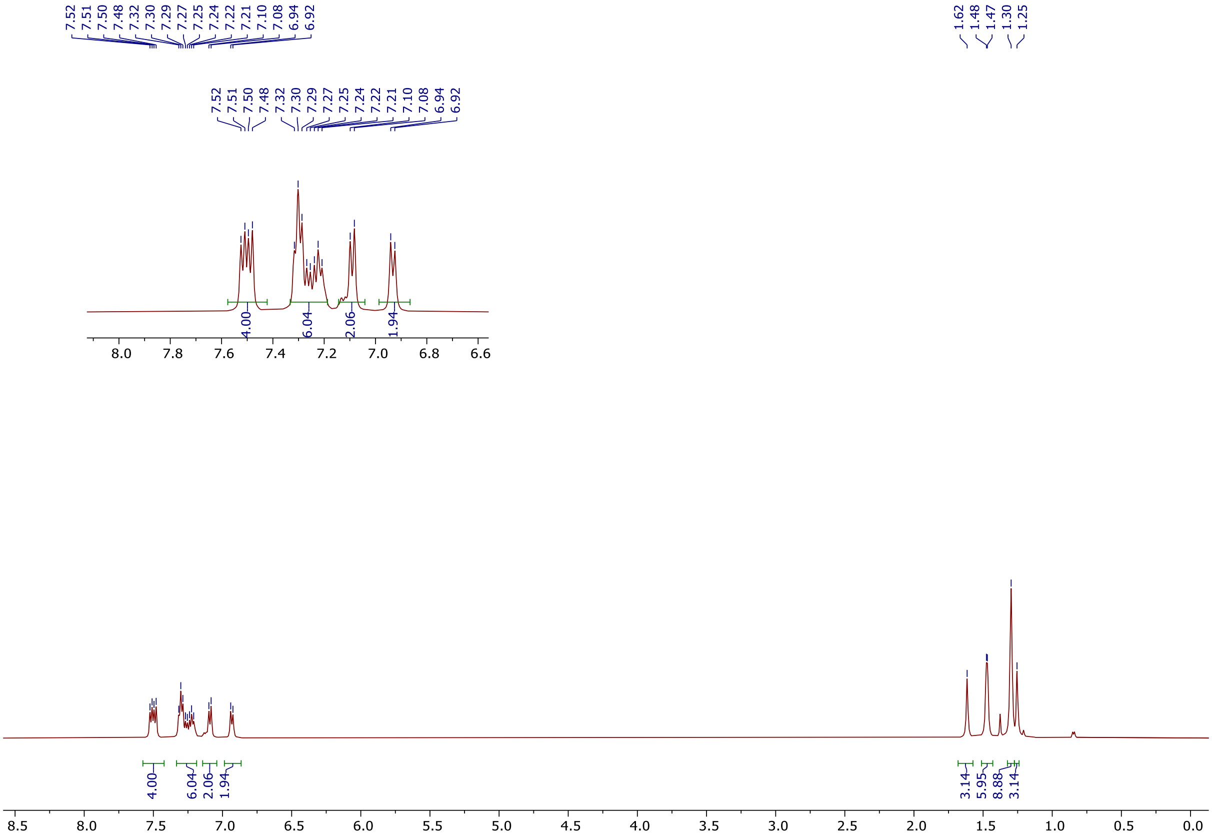

Figure S72: ^{1}H NMR Spectrum of **8** in $CDCl_3$ after isolation via column chromatography.

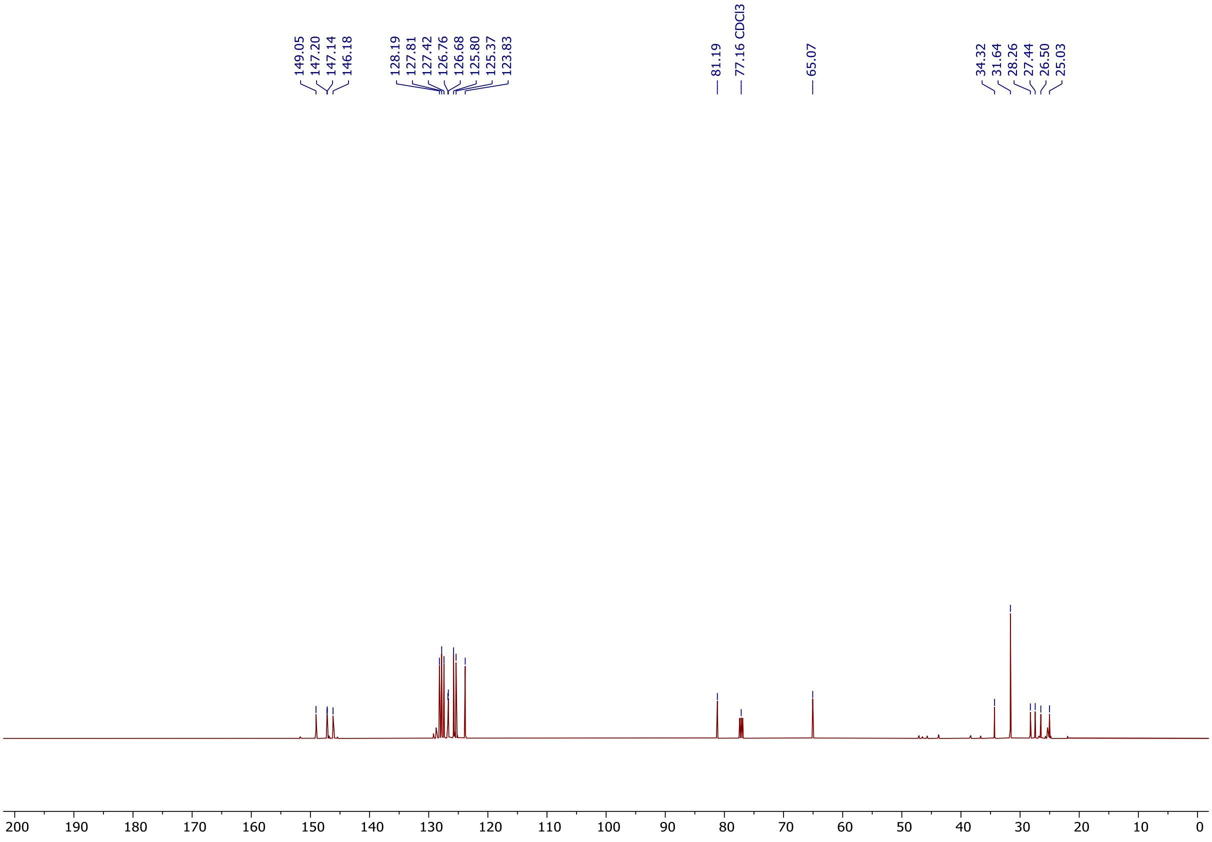

Figure S73: ^{13}C NMR Spectrum of **8** in $CDCl_3$ after isolation via column chromatography.

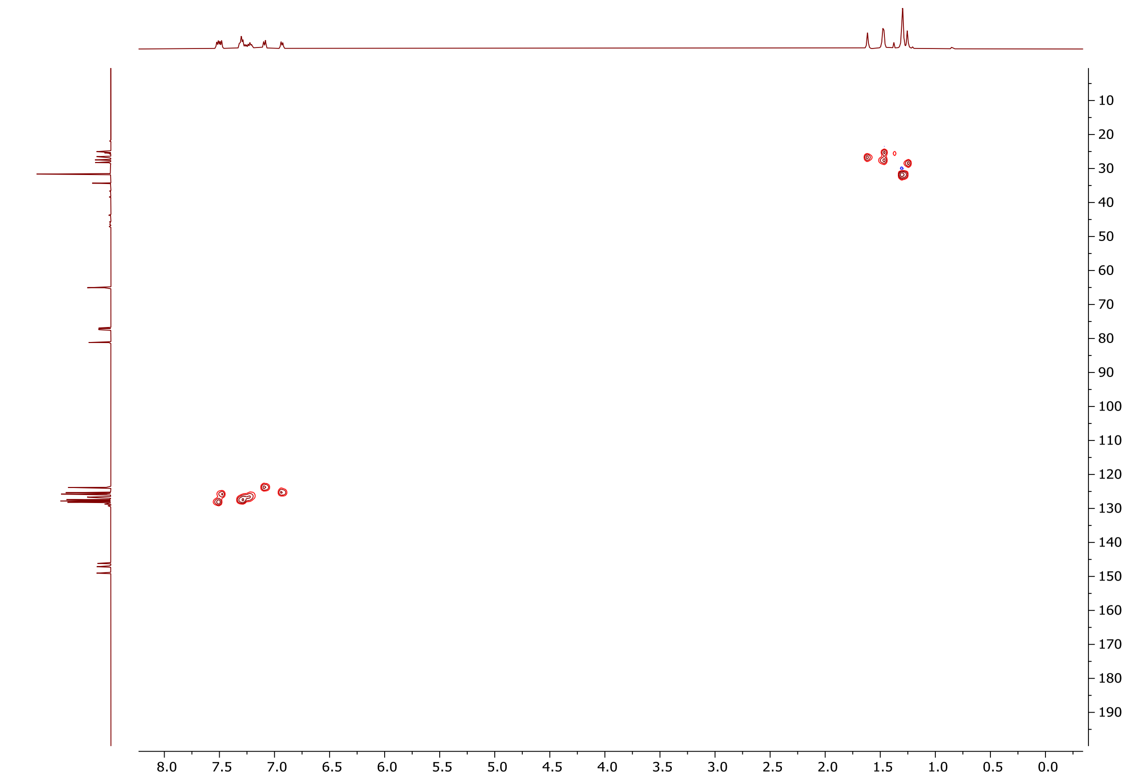

Figure S74: ^{1}H-^{13}C HSQC NMR Spectrum of **8** in $CDCl_3$ after isolation via column chromatography.

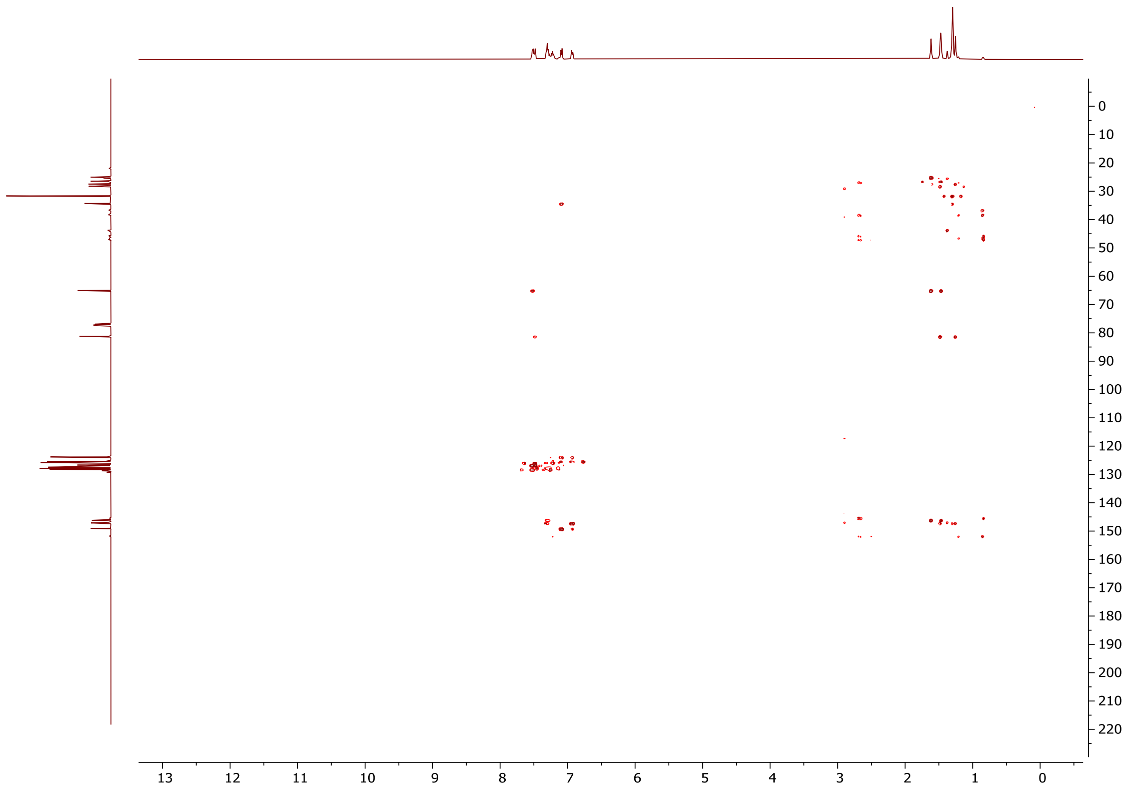

Figure S75: ^{1}H-^{13}C HMBC NMR Spectrum of **8** in $CDCl_3$ after isolation via column chromatography.

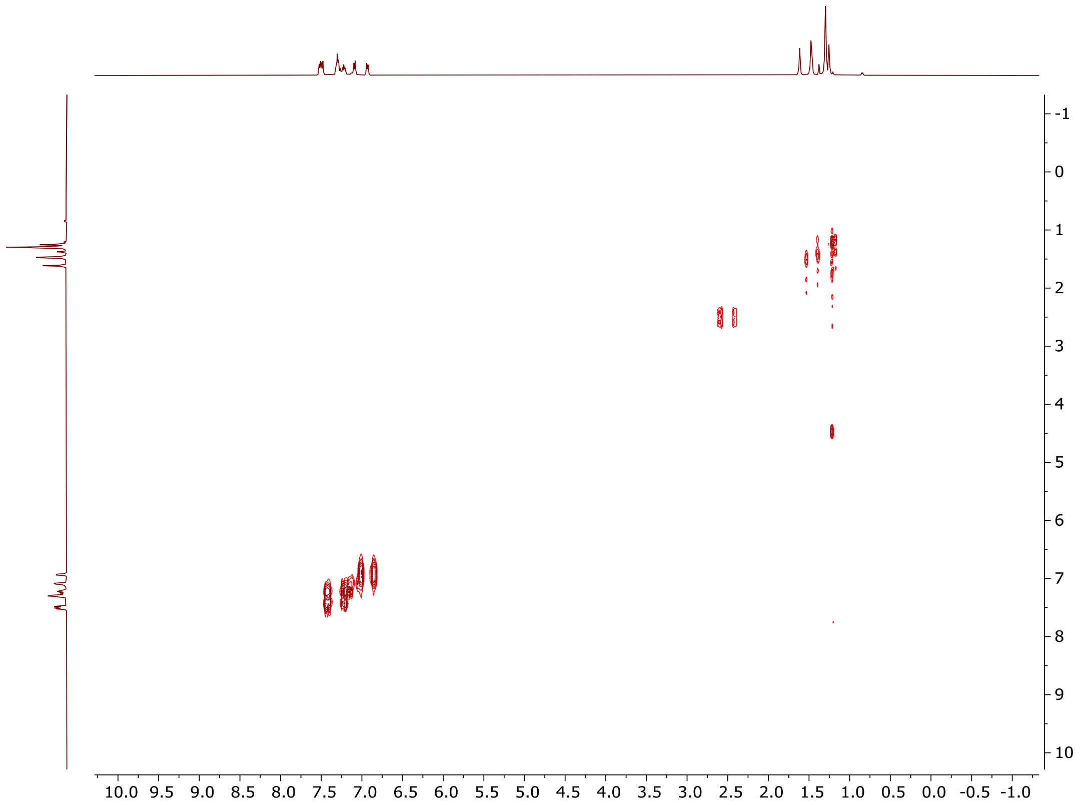

Figure S76: ^{1}H-^{1}H COSY NMR Spectrum of **8** in $CDCl_3$ after isolation via column chromatography.

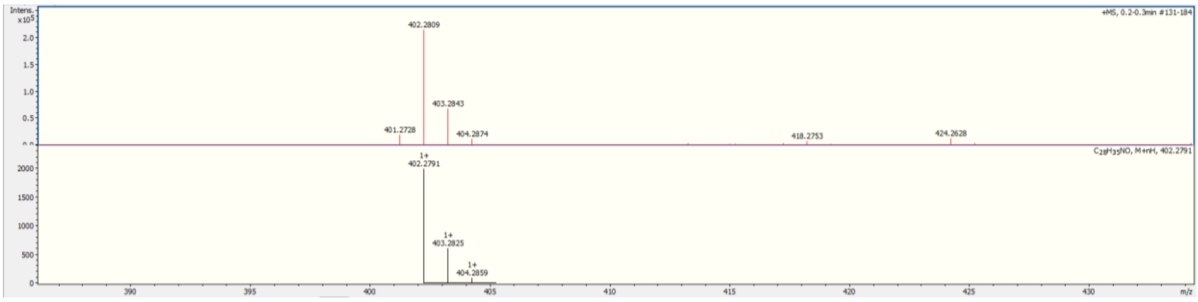

Figure S77: HRMS spectra for compound **8**.

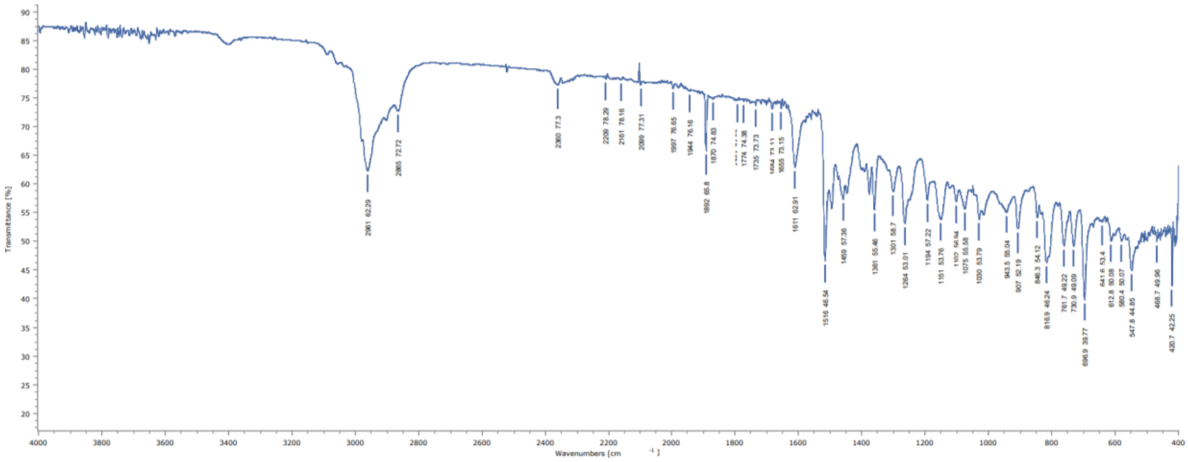

Figure S78: IR spectra for compound **8**.

3.1.8. 4-(tert-butyl)-N-(hexan-2-yl)aniline **9**

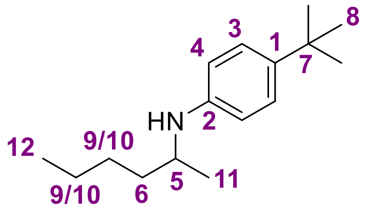

Light yellow oil

R_f = 0.22 (30% DCM / 70% hexane)

^{1}H NMR ($CDCl_3$, 400 MHz): δ 7.19 (d, J = 8.8 Hz, 2H, C^3-H), 6.53 (d, J = 8.8 Hz, 2H, C^4-H), 3.42 (app. p, J = 6.22, 1H, C^5-H), 3.35 (*br* s, 1H, NH), 1.56 – 1.53 (m, 2H, C^6-H), 1.43 – 1.32 (m, 4H, $C^{9/10}$-H), 1.28 (s, 9H, C^8-H), 1.17 (d, J = 6.3 Hz, 3H, C^{11}-H), 0.91 (t, J = 6.9 Hz, 3H, C^{12}-H).

$^{13}C\{^1H\}$ NMR ($CDCl_3$, 126 MHz): δ 145.5 (C^2), 139.6 (C^1), 126.2 (C^3), 112.8 (C^4), 48.7 (C^5) 37.2 (C^6), 33.9 (C^7), 31.7 (C^8), 28.6 (C^9), 22.9 (C^{10}), 21.1 (C^{11}), 14.3 (C^{12}).

HRMS (ESI+): calcd for [M, $C_{16}H_{28}N]^+$ 234.2216, found 234.2228.

IR (Neat): 2958, 2929, 1893, 1614, 1518, 1074, 817, 697 cm^{-1}.

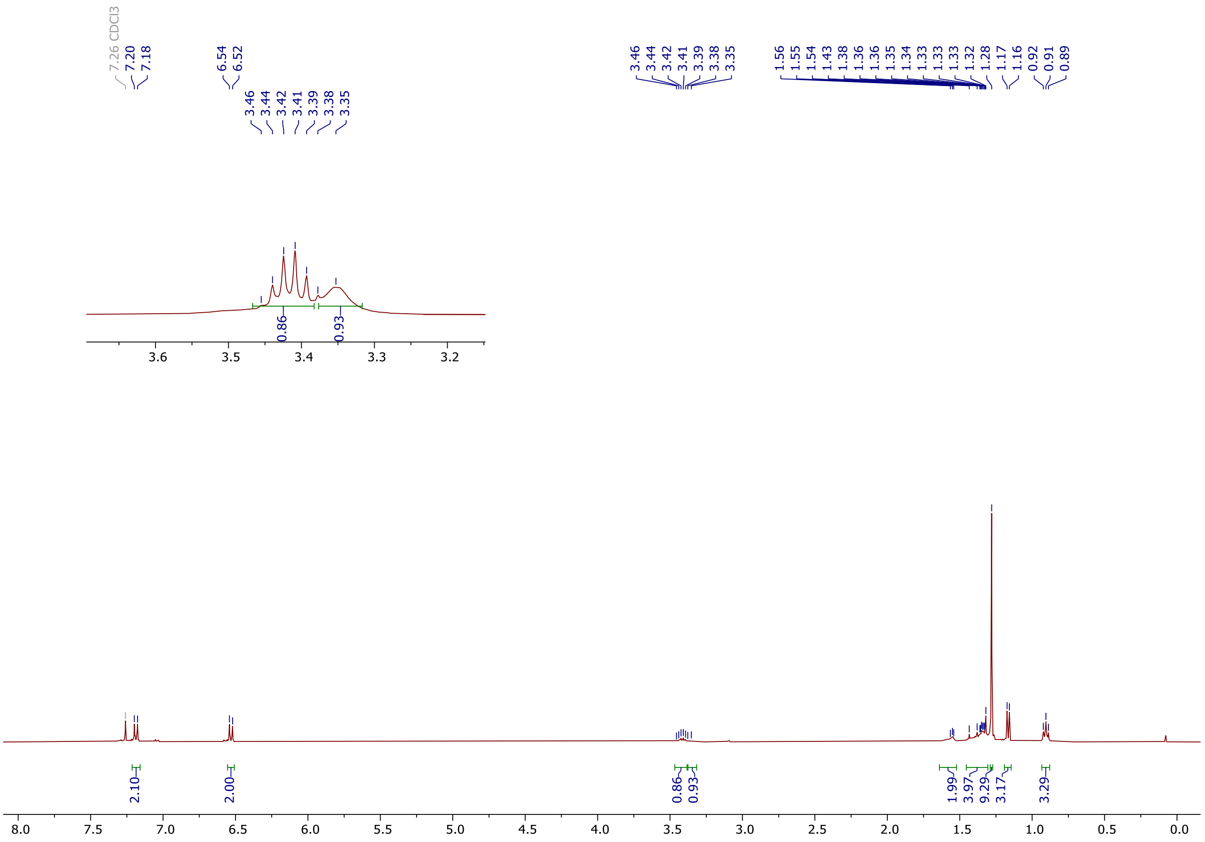

Figure S79: ^{1}H NMR Spectrum of **9** in $CDCl_3$ after isolation via column chromatography.

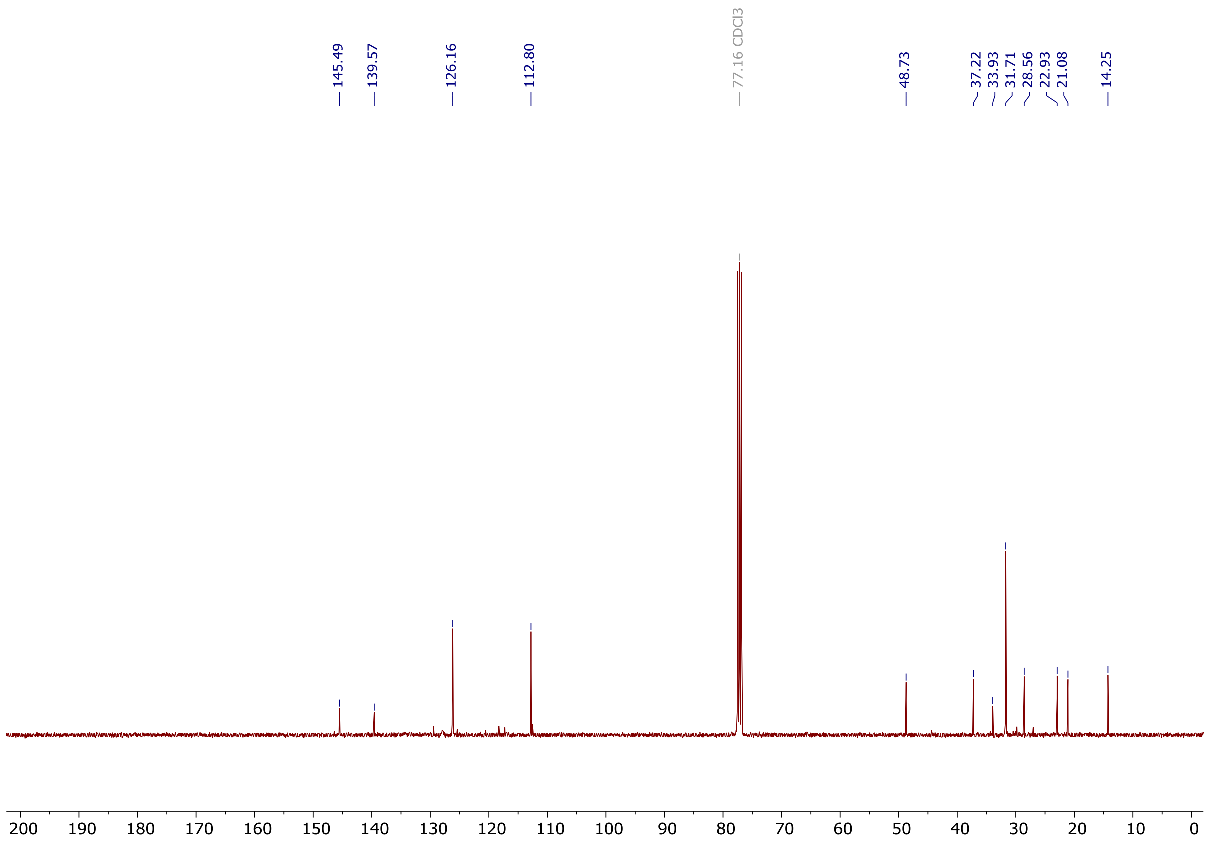

Figure S80: ^{13}C NMR Spectrum of **9** in $CDCl_3$ after isolation via column chromatography.

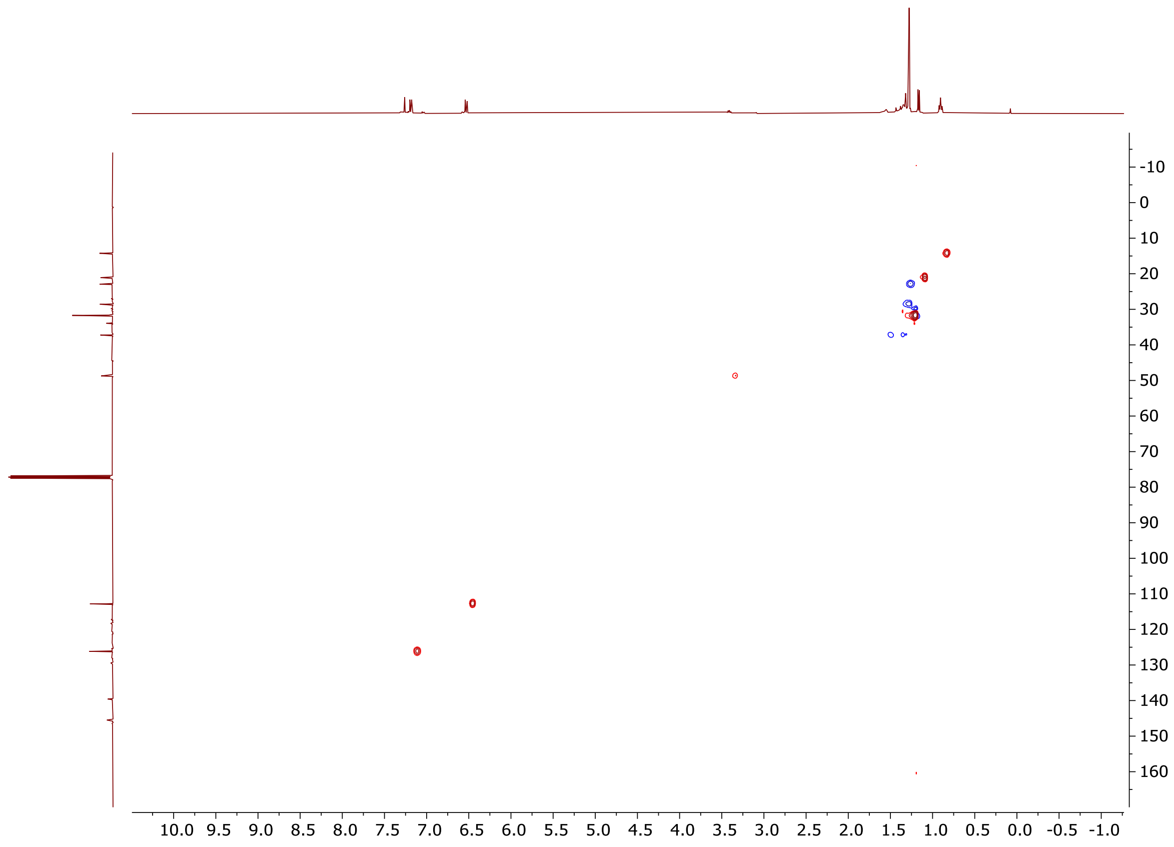

Figure S81: ^{1}H-^{13}C HSQC NMR Spectrum of **9** in $CDCl_3$ after isolation via column chromatography.

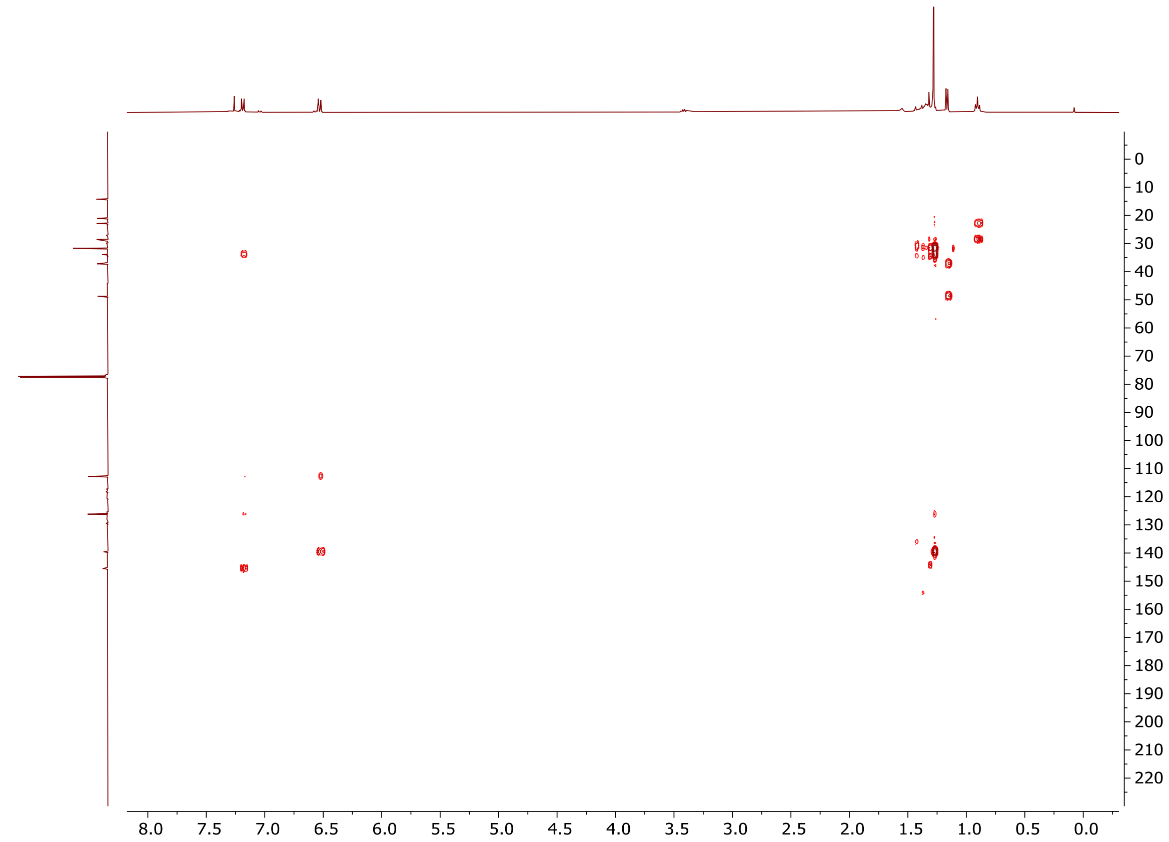

Figure S82: ^{1}H-^{13}C HMBC NMR Spectrum of **9** in $CDCl_3$ after isolation via column chromatography.

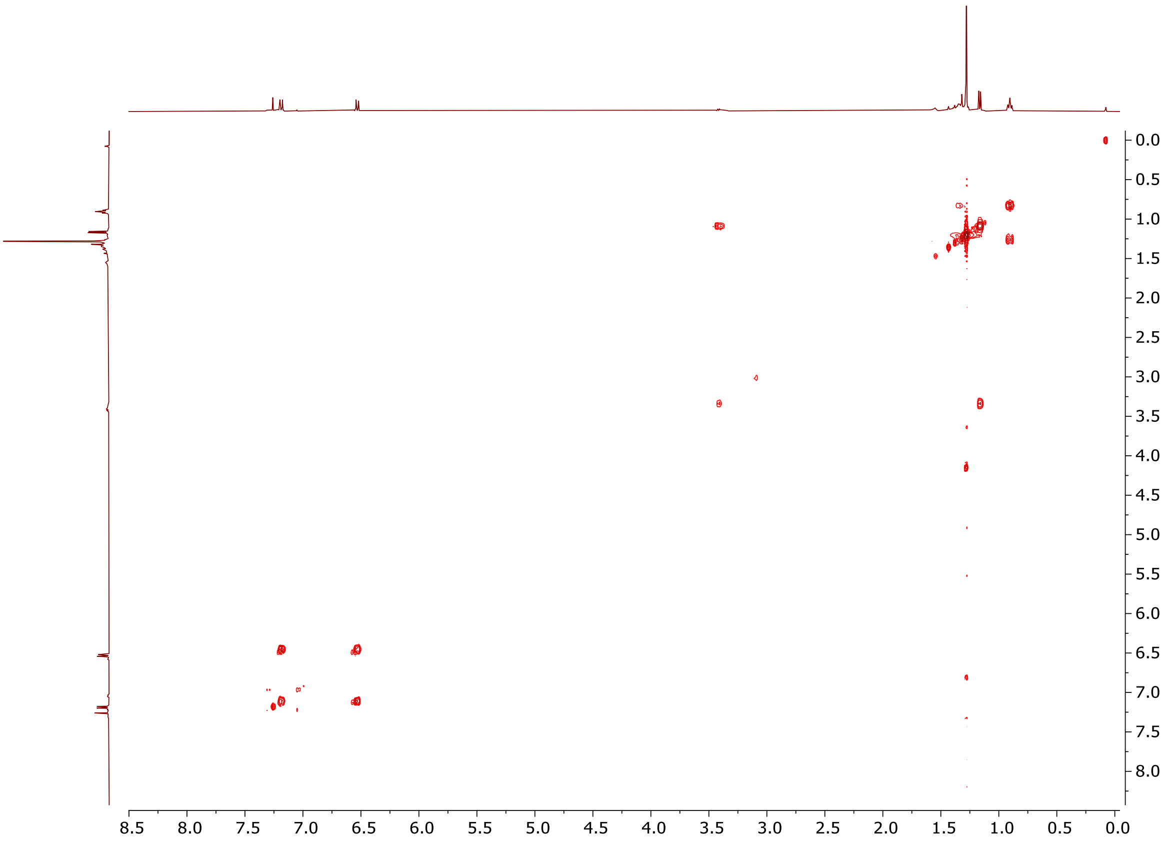

Figure S83: ^{1}H-^{1}H COSY NMR Spectrum of **9** in $CDCl_3$ after isolation via column chromatography.

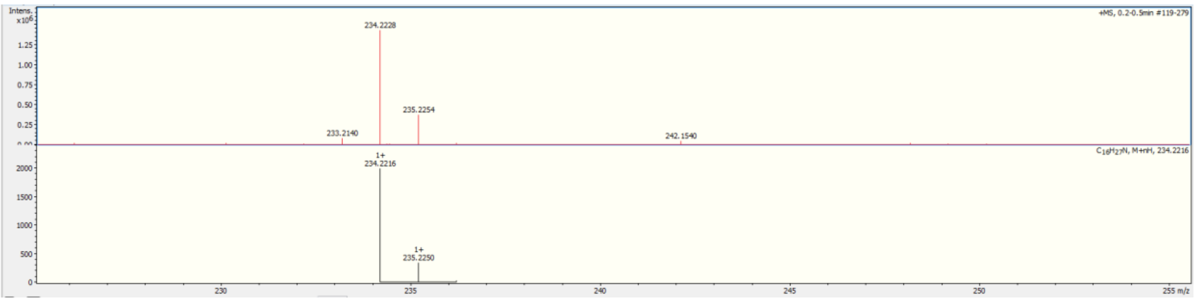

Figure S84: HRMS spectra for compound **9**.

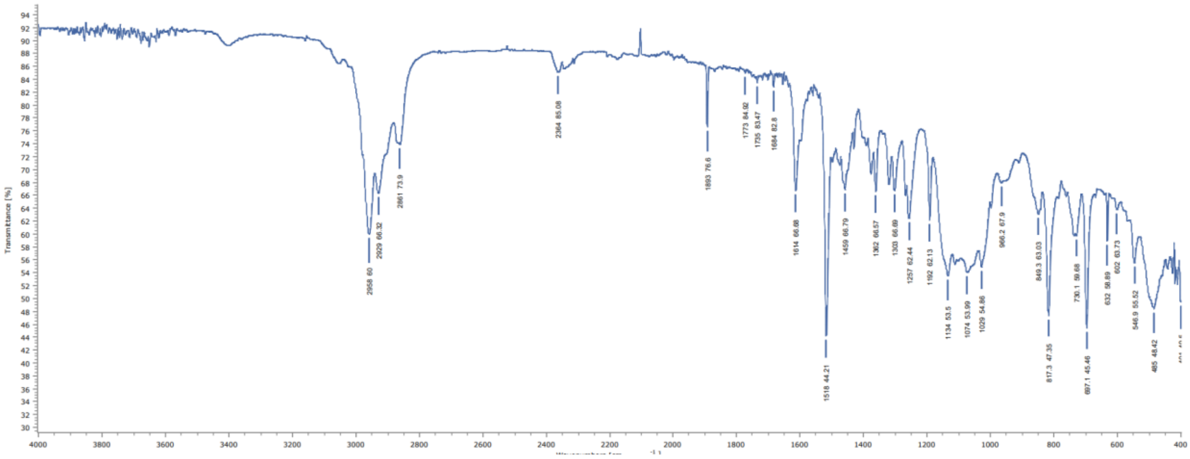

Figure S85: IR spectra for compound **9**.

3.1.9. N-(4-(tert-butyl)phenyl)-N,O-di(hexan-2-yl)hydroxylamine **10**

Mixture of diastereoisomers

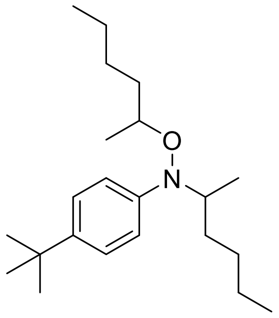

Colourless oil

R_f = 0.81 (30% DCM / 70% hexane)

^{1}H NMR ($CDCl_3$, 400 MHz): δ7.24 (dd, *J* = 9.1, 3.0 Hz, 2H), 7.00 (d, *J* = 8.7 Hz, 2H), 3.69 (app. dp, *J* = 12.5, 6.1 Hz, 1H), 3.25 (app. hept, *J* = 7.1, 6.6 Hz, 1H), 1.76 – 1.60 (m, 2H), 1.41 – 1.25 (m, 20H), 1.10 (dd, *J* = 15.4, 6.2 Hz, 2H), 1.01 (dd, *J* = 19.6, 6.5 Hz, 2H), 0.92 – 0.81 (m, 7H).

^{13}C{^{1}H} NMR ($CDCl_3$, 126 MHz): δ 150.36, 149.94, 144.83, 144.66, 125.15, 125.10, 118.95, 118.74, 64.56, 64.36, 35.52, 35.28, 34.26, 34.24, 31.66, 29.86, 29.47, 28.16, 28.04, 23.10, 23.07, 22.99, 22.96, 19.52, 19.28, 14.32, 14.30, 14.23.

HRMS (ESI+): calcd for [M, $C_{22}H_{40}NO]^+$ 334.3110, found 334.3106.

IR (Neat): 2957.3, 2930.8, 1609.7, 1504.6, 1371.8, 1112.6, 835.6 cm^{-1}.

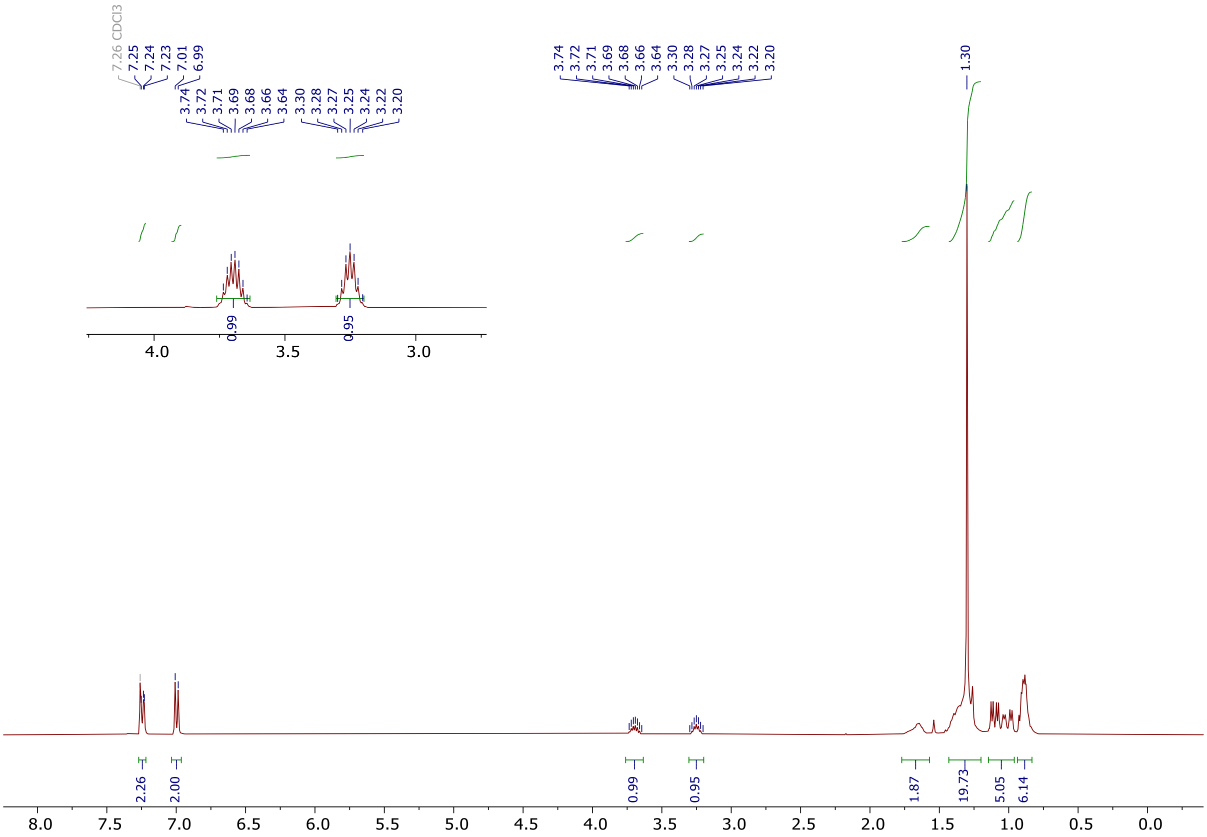

Figure S86: ^{1}H NMR Spectrum of **10** in $CDCl_3$ after isolation via column chromatography.

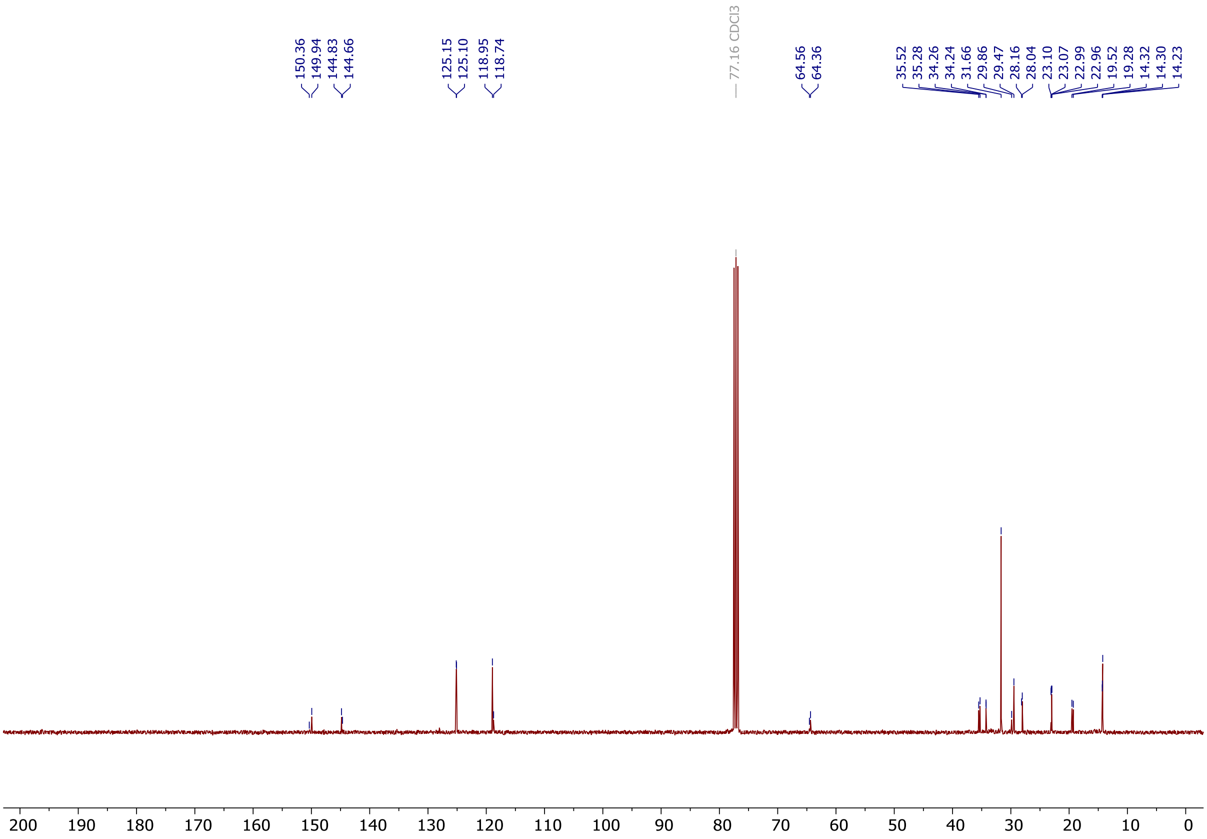

Figure S87: ^{13}C NMR Spectrum of **10** in $CDCl_3$ after isolation via column chromatography.

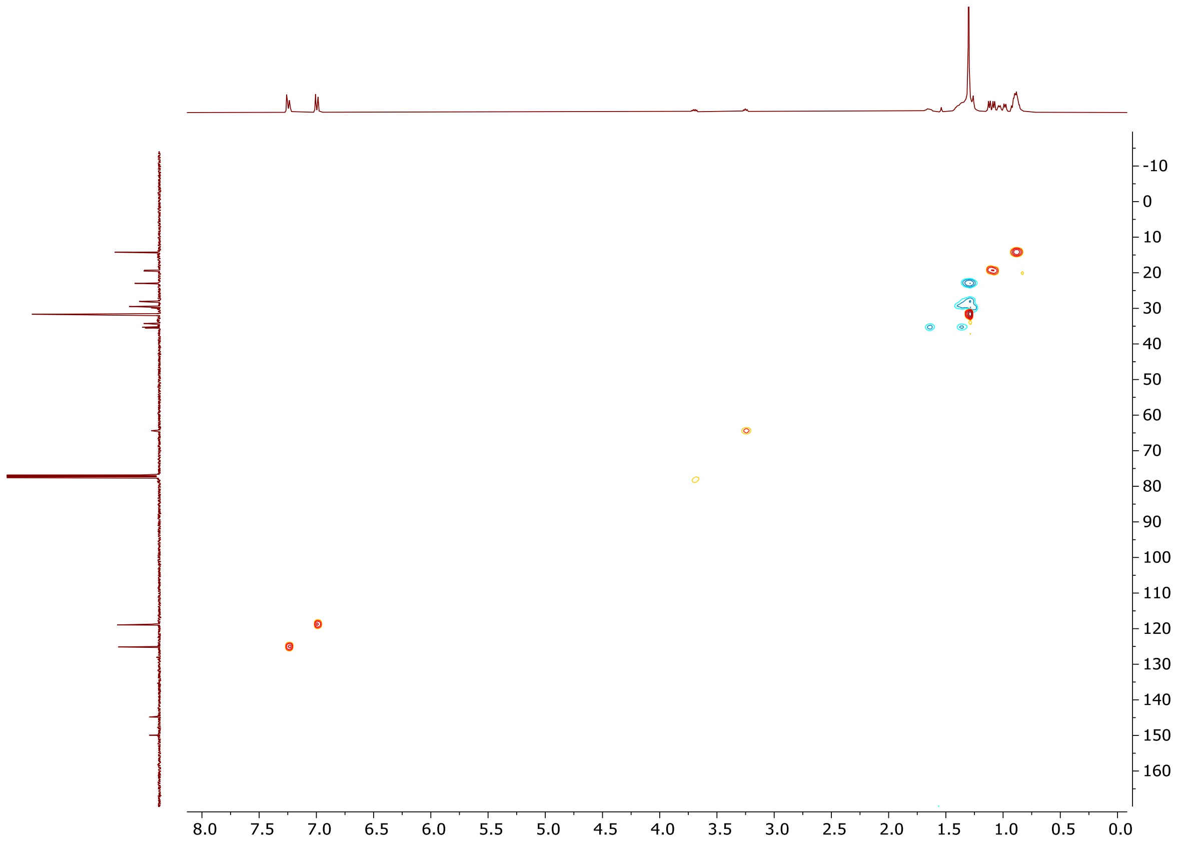

Figure S88: ^{1}H-^{13}C HSQC NMR Spectrum of **10** in $CDCl_3$ after isolation via column chromatography.

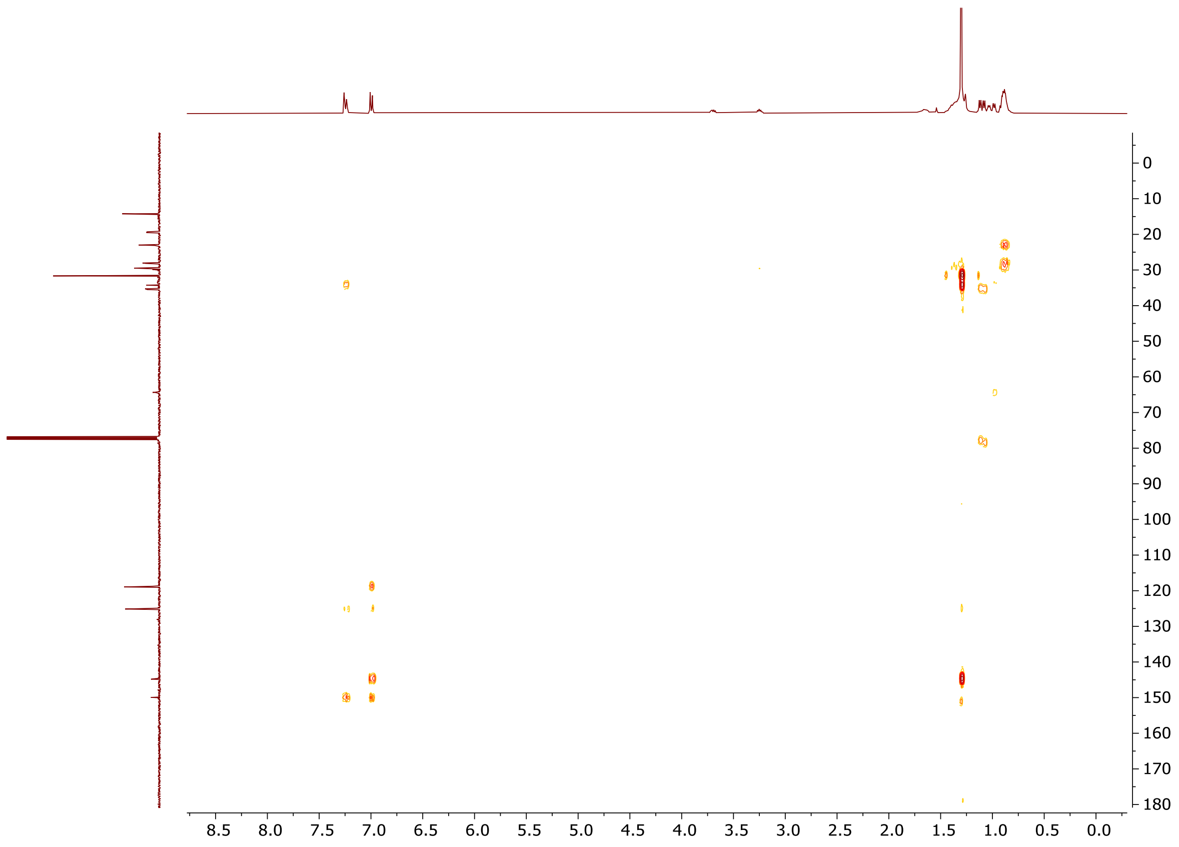

Figure S89: ^{1}H-^{13}C HMBC NMR Spectrum of **10** in $CDCl_3$ after isolation via column chromatography.

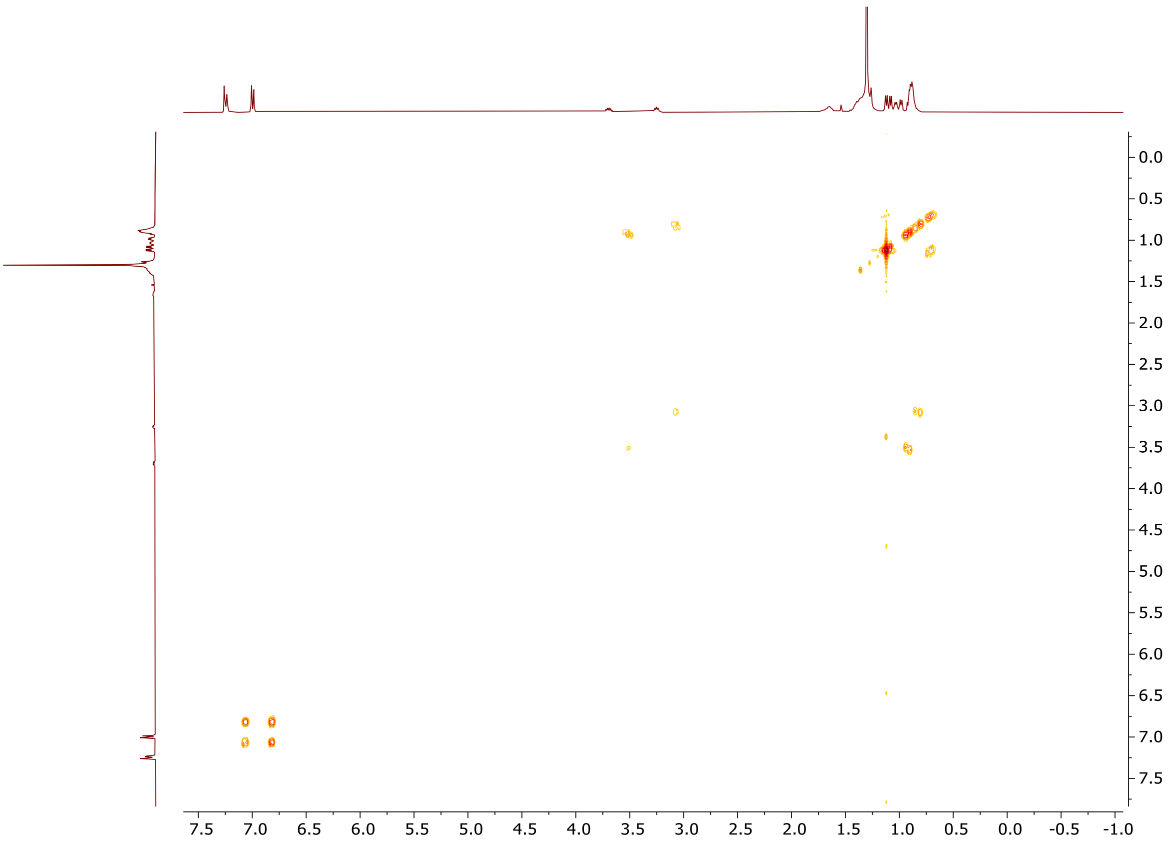

Figure S90: ^1H-^1H COSY NMR Spectrum of **10** in $CDCl_3$ after isolation via column chromatography.

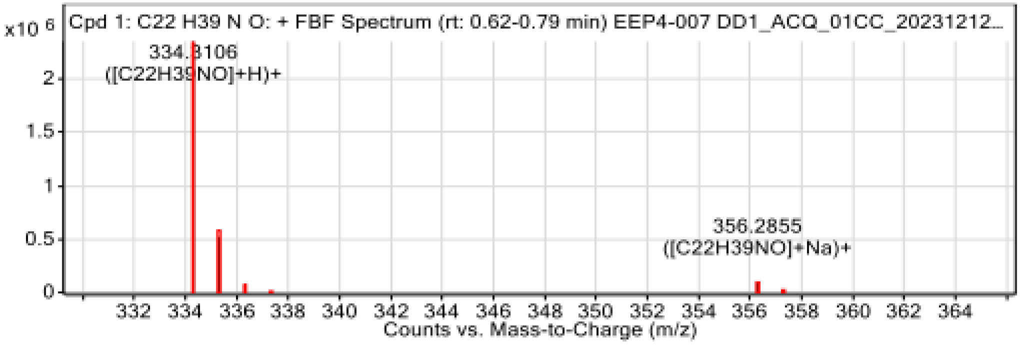

Figure S91: HRMS spectra for compound **10**.

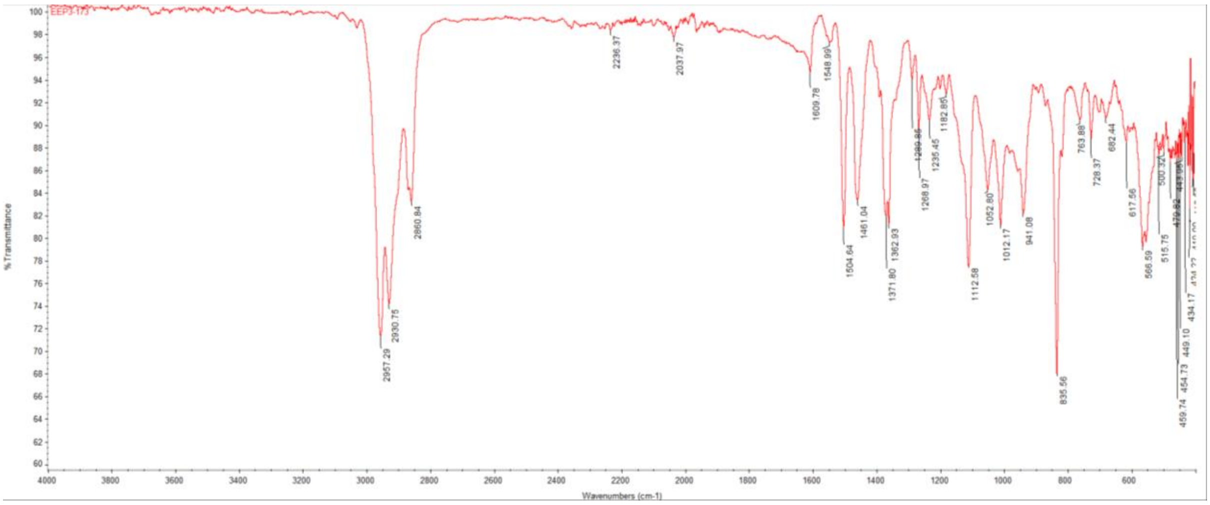

Figure S92: IR spectra for compound **10**.

3.1.10. 1,2-bis(4-(tert-butyl)phenyl)diazene **11**

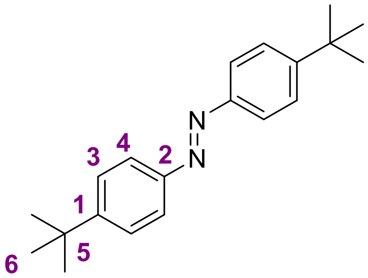

Bright Orange Solid

R_f = 0.83 (30% DCM / 70% hexane)

^{1}H NMR ($CDCl_3$, 500 MHz): δ 7.84 (d, *J* = 8.6 Hz, 4H), 7.53 (d, *J* = 8.6 Hz, 4H), 1.38 (s, 18H).

^{13}C{^{1}H} NMR ($CDCl_3$, 126 MHz): δ 154.4, 150.9, 126.1, 122.6, 35.1, 31.4.

HRMS (ESI+): calcd for [M, $C_{20}H_{27}N_2$]$^+$ 295.2169, found 295.2171.

IR (Neat): 2954.8, 2924.2, 1600.7, 1267.3, 843.7 cm^{-1}.

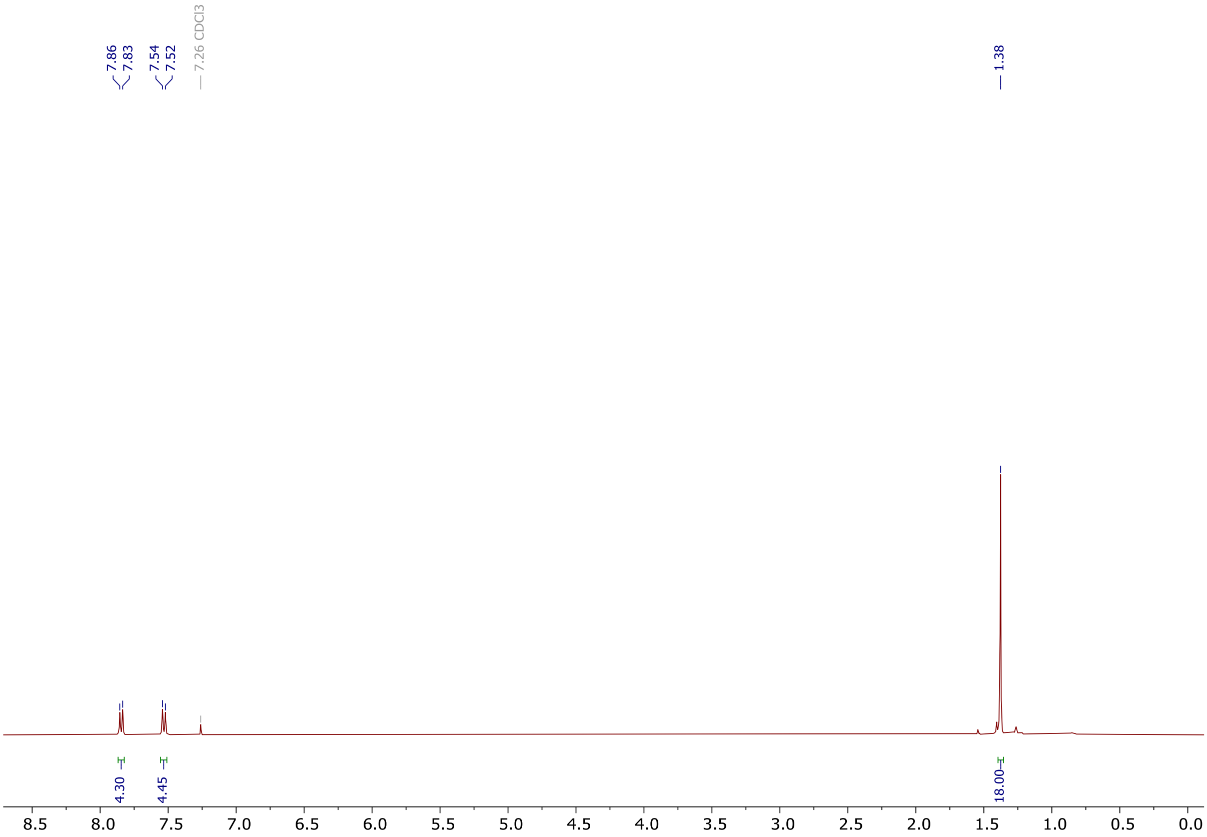

Figure S93: ^{1}H NMR Spectrum of **11** in $CDCl_3$ after isolation via column chromatography.

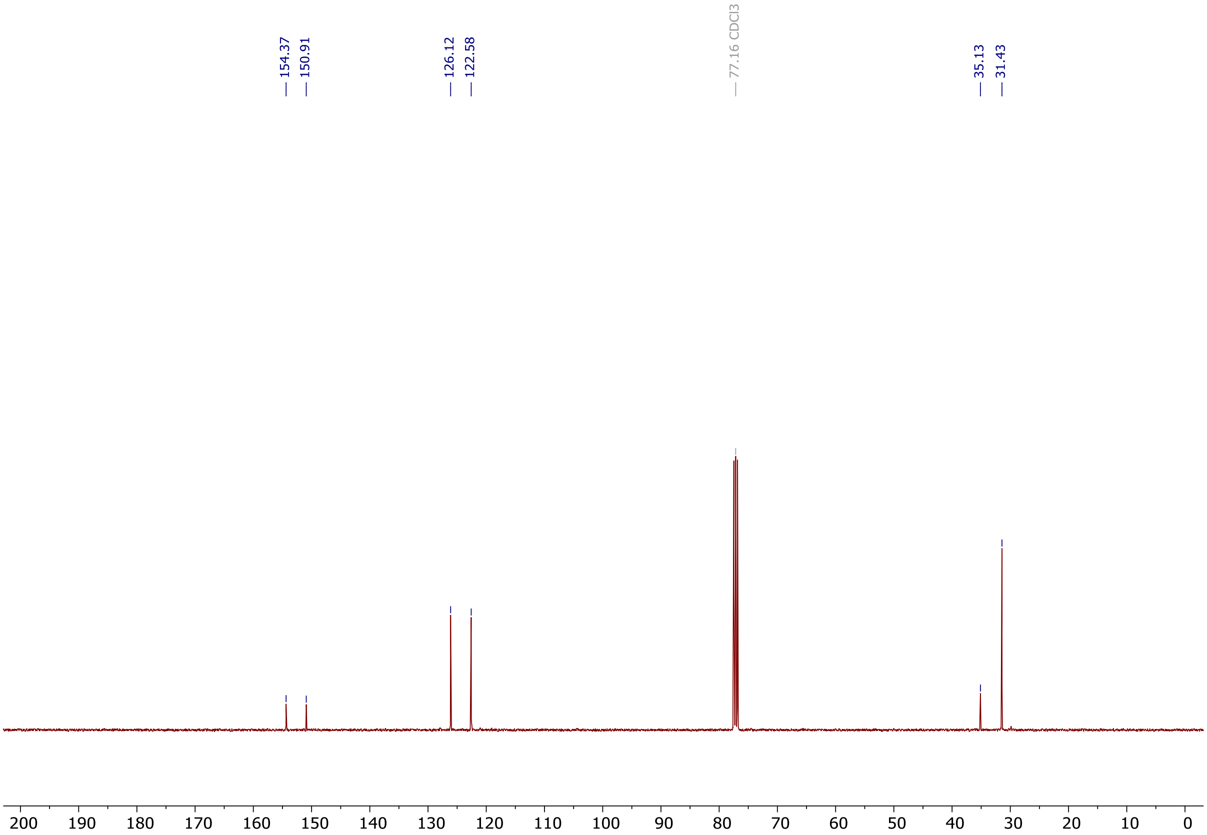

Figure S94: ^{13}C NMR Spectrum of **11** in $CDCl_3$ after isolation via column chromatography.

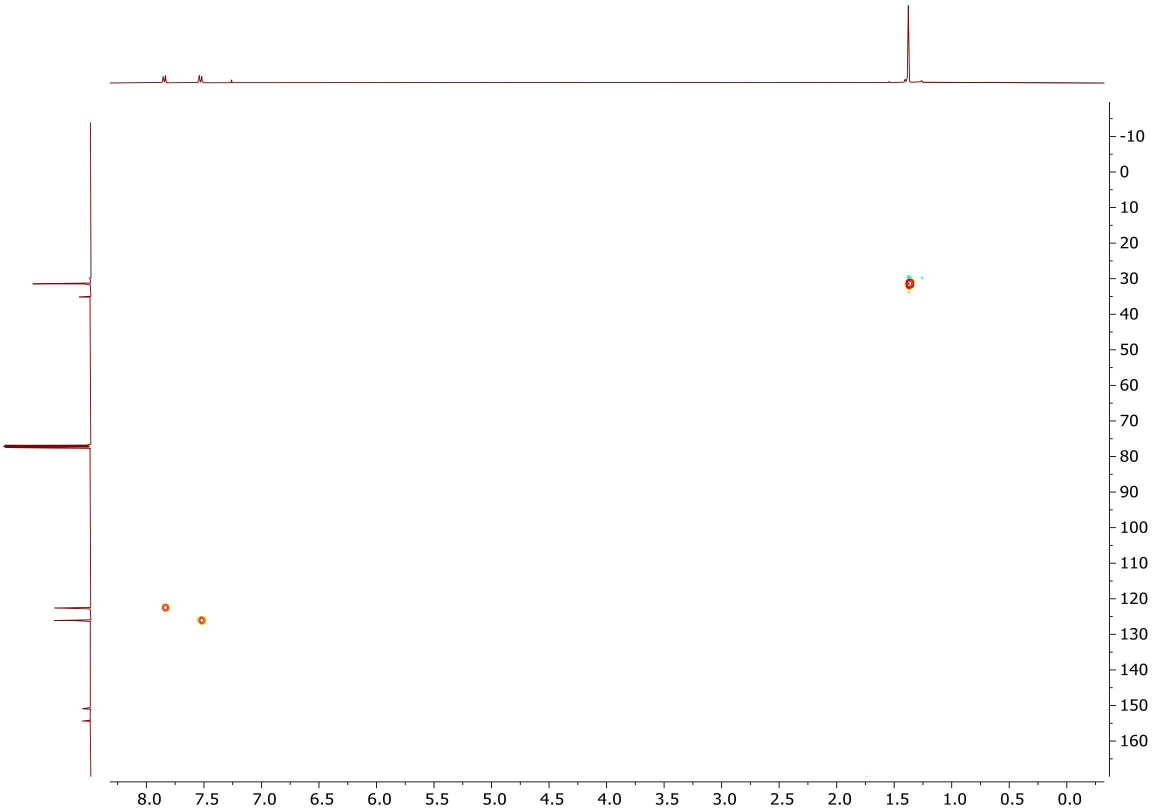

Figure S95: ^{1}H-^{13}C HSQC NMR Spectrum of **11** in $CDCl_3$ after isolation via column chromatography.

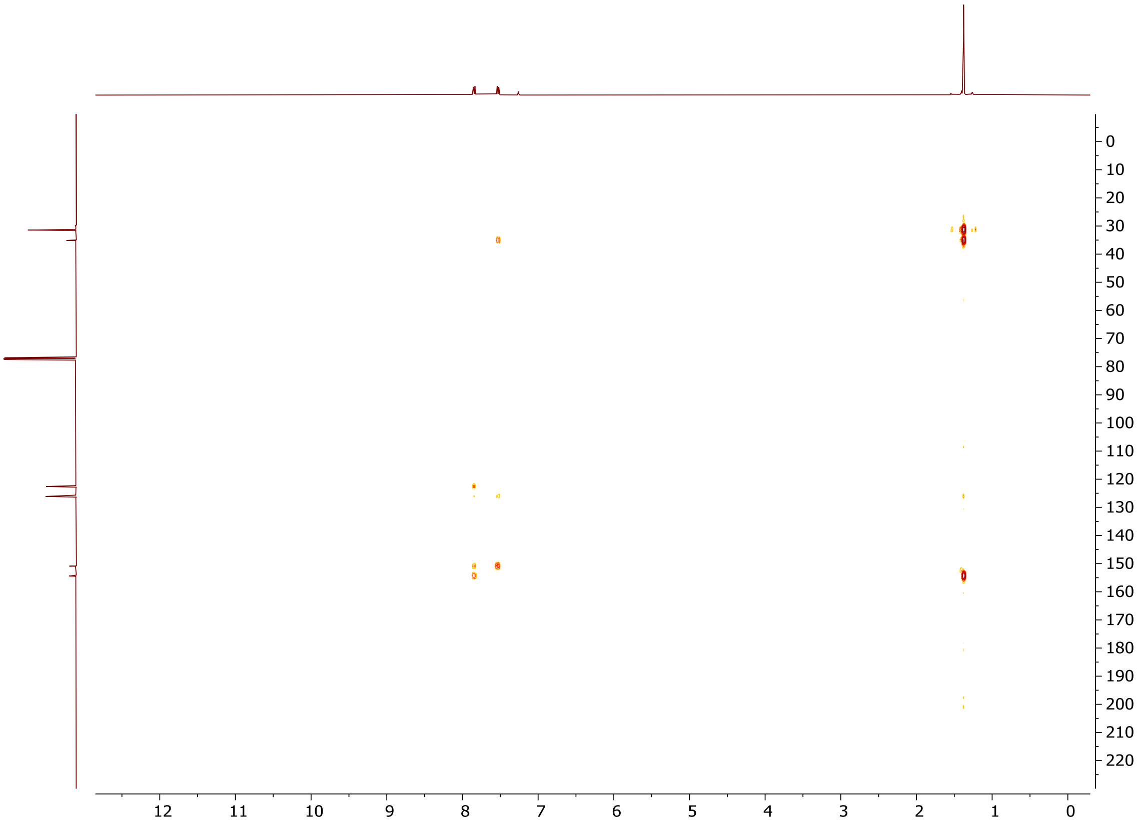

Figure S96: ^{1}H-^{13}C HMBC NMR Spectrum of **11** in $CDCl_3$ after isolation via column chromatography.

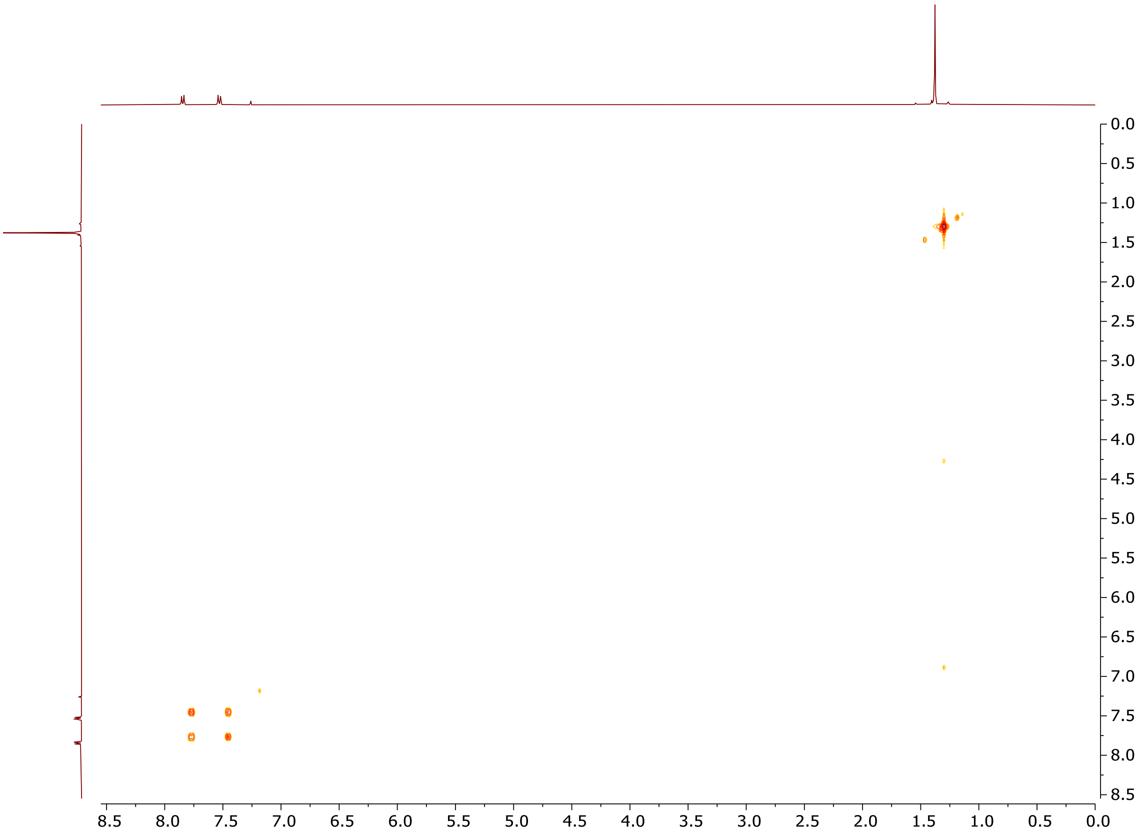

Figure S97: ^{1}H-^{1}H COSY NMR Spectrum of **11** in $CDCl_3$ after isolation via column chromatography.

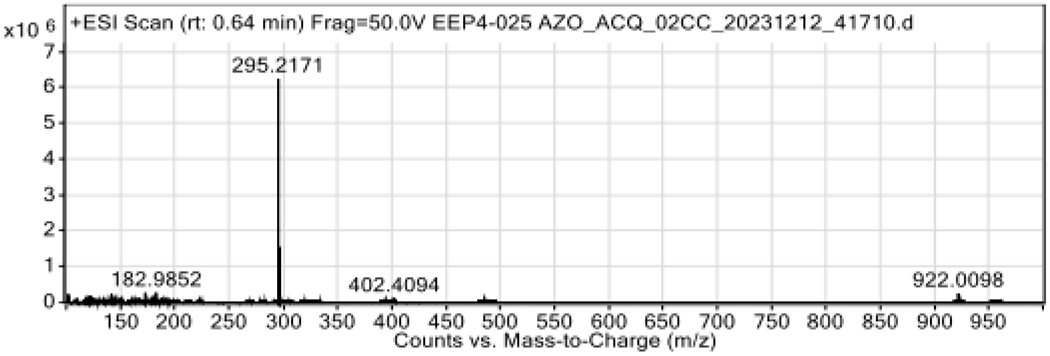

Figure S98: HRMS spectra for compound **11**.

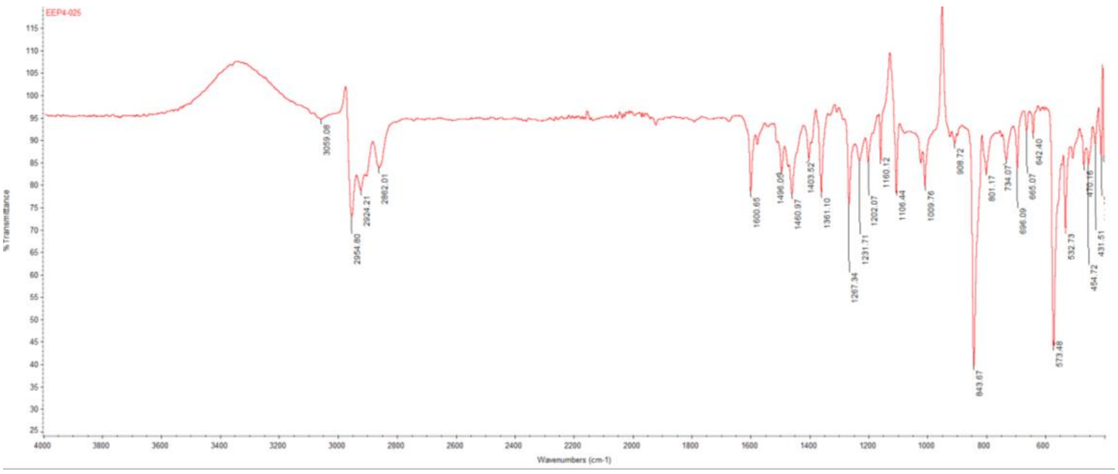

Figure S99: IR spectra for compound **11**.

3.1.11. 4-(tert-butyl)-N-(1,2-diphenylethyl)aniline **12**

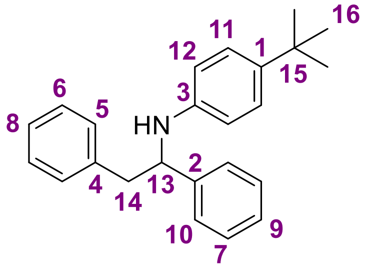

Yellow oil

R_f = 0.33 (30% DCM / 70% hexane)

^{1}H NMR ($CDCl_3$, 400 MHz): δ 7.29 – 7.16 (m, 8H, C^{5-10}-H), 7.07 (dd, J = 8.3, 1.6 Hz, 2H, $C^{6/7}$-H), 7.02 (d, J = 8.7 Hz, 2H, C^{11}-H), 6.35 (d, J = 8.7 Hz, 2H, C^{12}-H), 4.47 (dd, J = 8.5, 5.6 Hz, 1H, C^{13}-H), 3.98 (*br* s, 1H, NH), 3.05 (dd, J = 13.9, 5.6 Hz, 1H, C^{14}-H), 2.92 (dd, J = 13.9, 8.5 Hz, 1H, C^{14}-H), 1.15 (s, 9H, C^{16}-H).

^{13}C{^{1}H} NMR ($CDCl_3$, 101 MHz): δ 145.2 (C^1), 144.0 (C^2), 140.3 (C^3), 138.0 (C^4), 129.3 (C^5), 128.7 (C^6), 128.7 (C^7), 127.2 (C^8), 126.8 (C^9), 126.6 (C^{10}), 125.9 (C^{11}), 113.5 (C^{12}), 59.8 (C^{13}), 45.5 (C^{14}), 33.9 (C^{15}) 31.6 (C^{16})

HRMS (ESI+): calcd for [M, $C_{24}H_{27}N$]$^+$ 330.2216, found 330.2229.

IR (Neat): 3406, 2959, 1893, 1615, 1517, 818, 697 cm^{-1}.

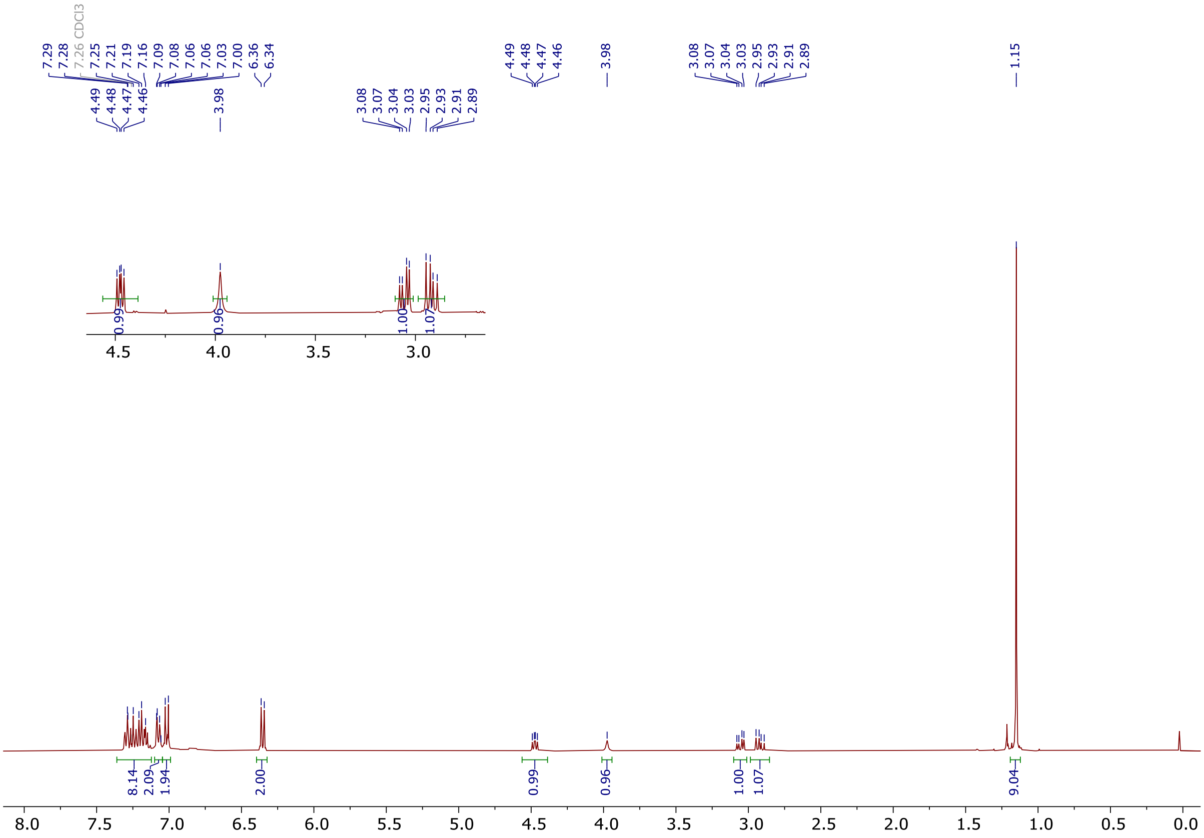

Figure S100: ^{1}H NMR Spectrum of **12** in $CDCl_3$ after isolation via column chromatography.

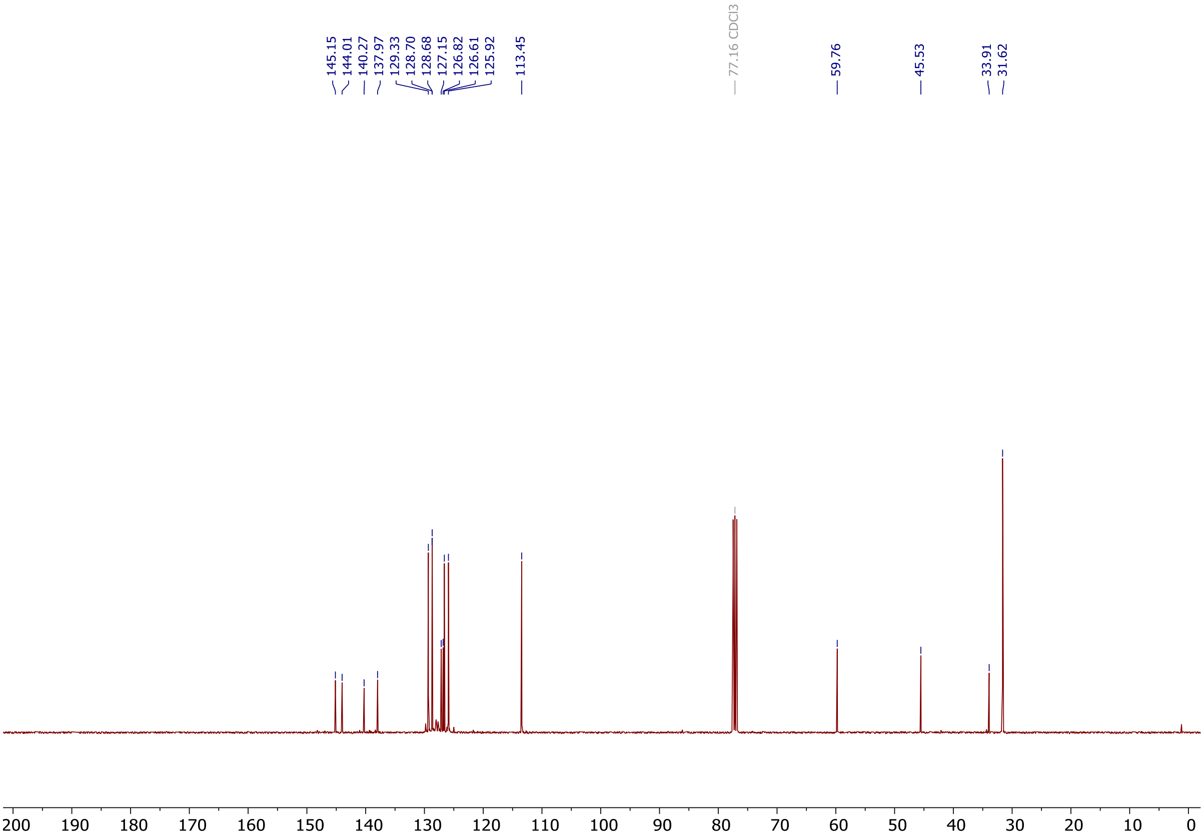

Figure S101: ^{13}C NMR Spectrum of **12** in $CDCl_3$ after isolation via column chromatography.

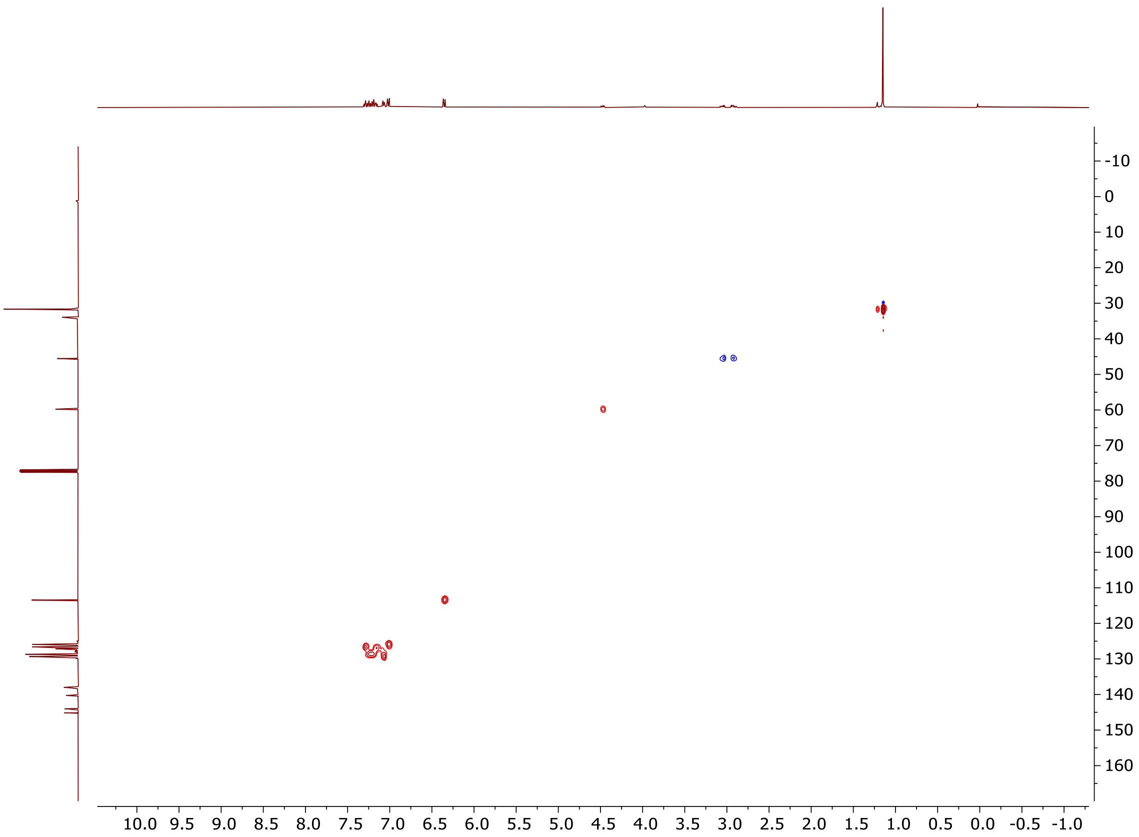

Figure S102: ^{1}H-^{13}C HSQC NMR Spectrum of **12** in $CDCl_3$ after isolation via column chromatography.

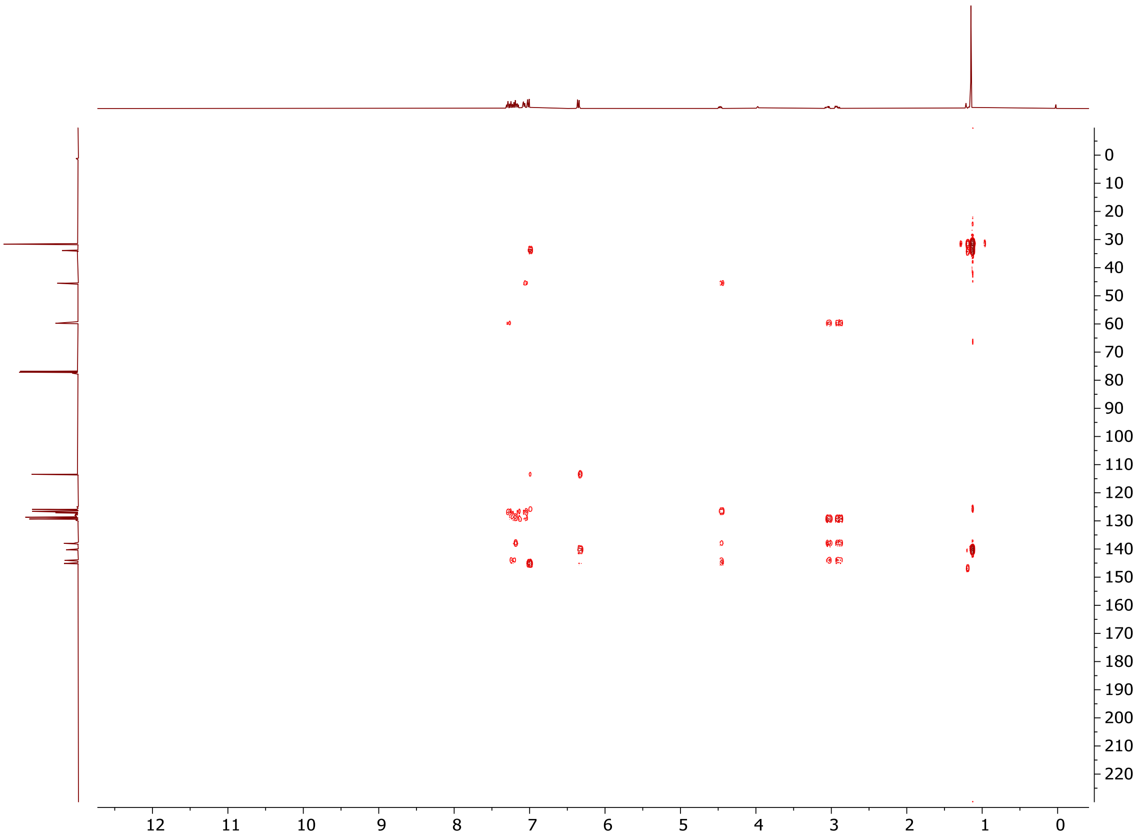

Figure S103: ^{1}H-^{13}C HMBC NMR Spectrum of **12** in $CDCl_3$ after isolation via column chromatography.

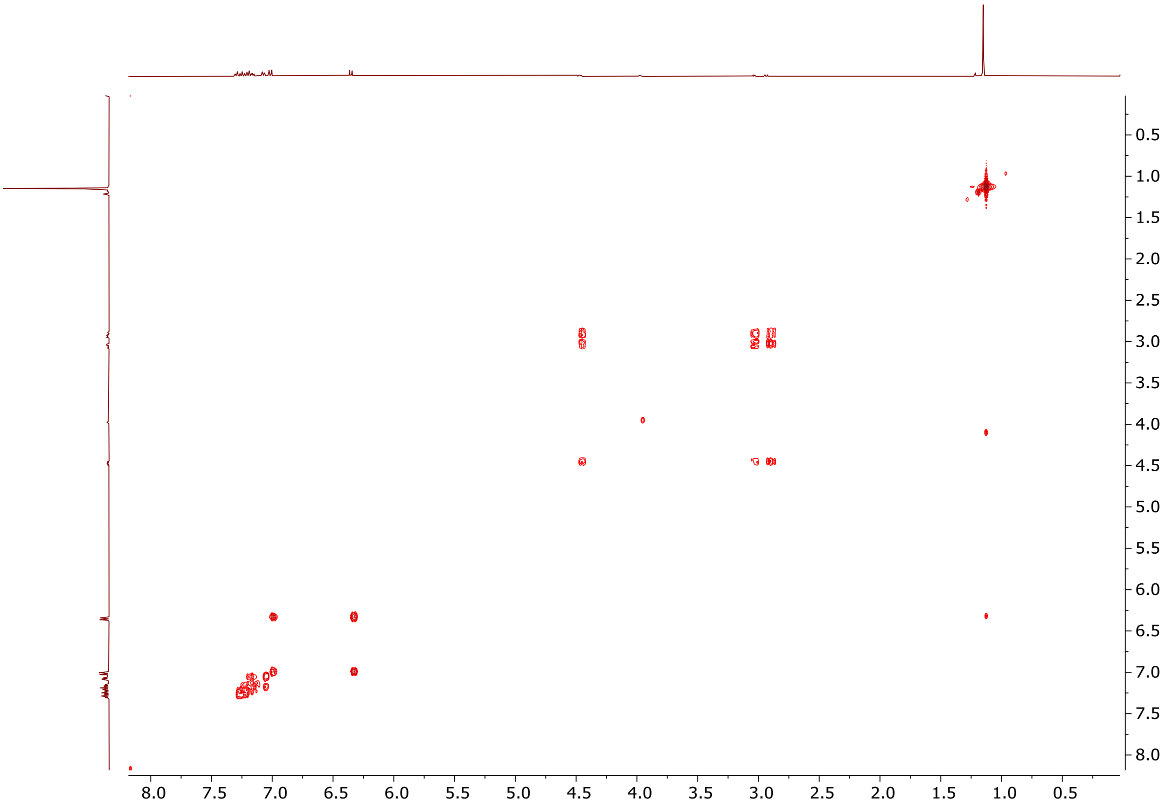

Figure S104: ^{1}H-^{1}H COSY NMR Spectrum of **12** in $CDCl_3$ after isolation via column chromatography.

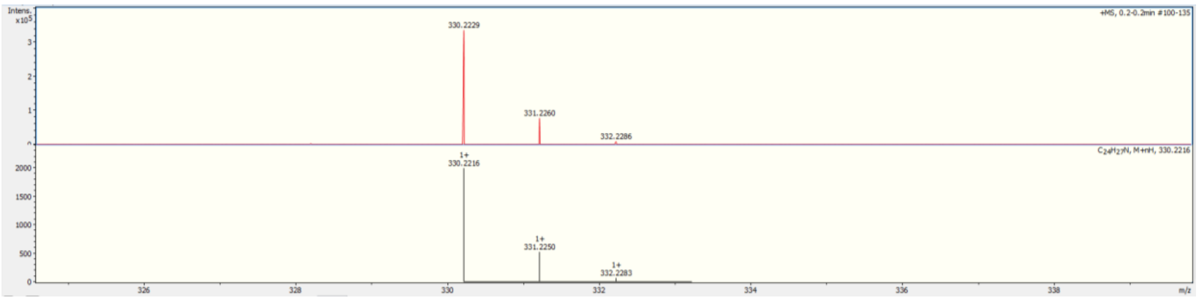

Figure S105: HRMS spectra for compound **12**.

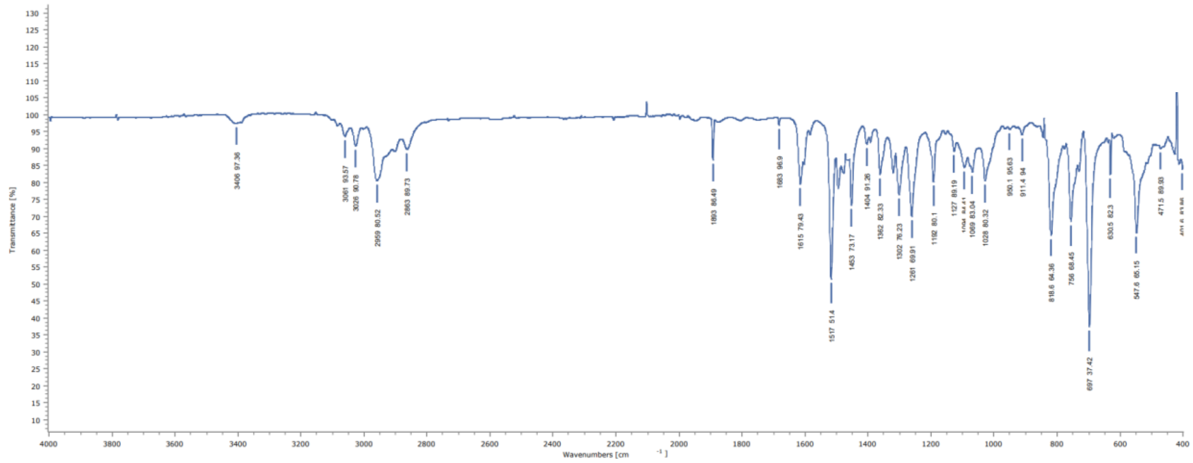

Figure S106: IR spectra for compound **12**.

3.1.12. N-phenyl-N,O-bis(2-phenylpropan-2-yl)hydroxylamine **13**

<u>Mixture of diastereoisomers</u>

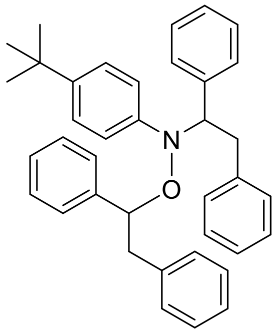

Colourless oil

R_f = 0.46 (30% DCM / 70% hexane)

^{1}H NMR ($CDCl_3$, 500 MHz): δ 7.15 – 7.12 (m, 4H, Ar-H), 7.08 – 6.91 (m, 32H, Ar-H), 6.84 – 6.68 (m, 12H, Ar – H), 4.45 (t, J = 7.1 Hz, 2H, CH), 4.41 – 4.33 (m, 2H, CH), 3.20 – 2.88 (m, 6H, CH_2), 2.67 – 2.56 (m, 2H, CH_2), 1.19 (app. d, J = 2.6 Hz, 18H, $(CH_3)_3$).

^{13}C{^{1}H} NMR ($CDCl_3$, 126 MHz): δ 148.2, 148.0, 147.0, 146.6, 141.2, 141.0, 139.7, 139.4, 139.3, 139.0, 138.4, 138.4, 129.9, 129.8, 129.7, 129.3, 128.1, 128.1, 128.0, 127.9, 127.9, 127.7, 127.7, 127.6, 127.6, 127.4, 127.4, 127.2, 126.6, 126.0, 126.0, 125.9, 125.8, 125.1, 125.0, 121.7, 121.0, 113.5, 86.1, 85.2, 74.4, 74.2, 42.1, 42.0, 34.4, 34.3, 31.6, 31.6.

HRMS (ESI+): calcd for [M, $C_{38}H_{39}NO]^+$ 526.3110, found 526.3106.

IR (Neat): 3028, 2950, 1495, 1452, 12117, 908 cm^{-1}.

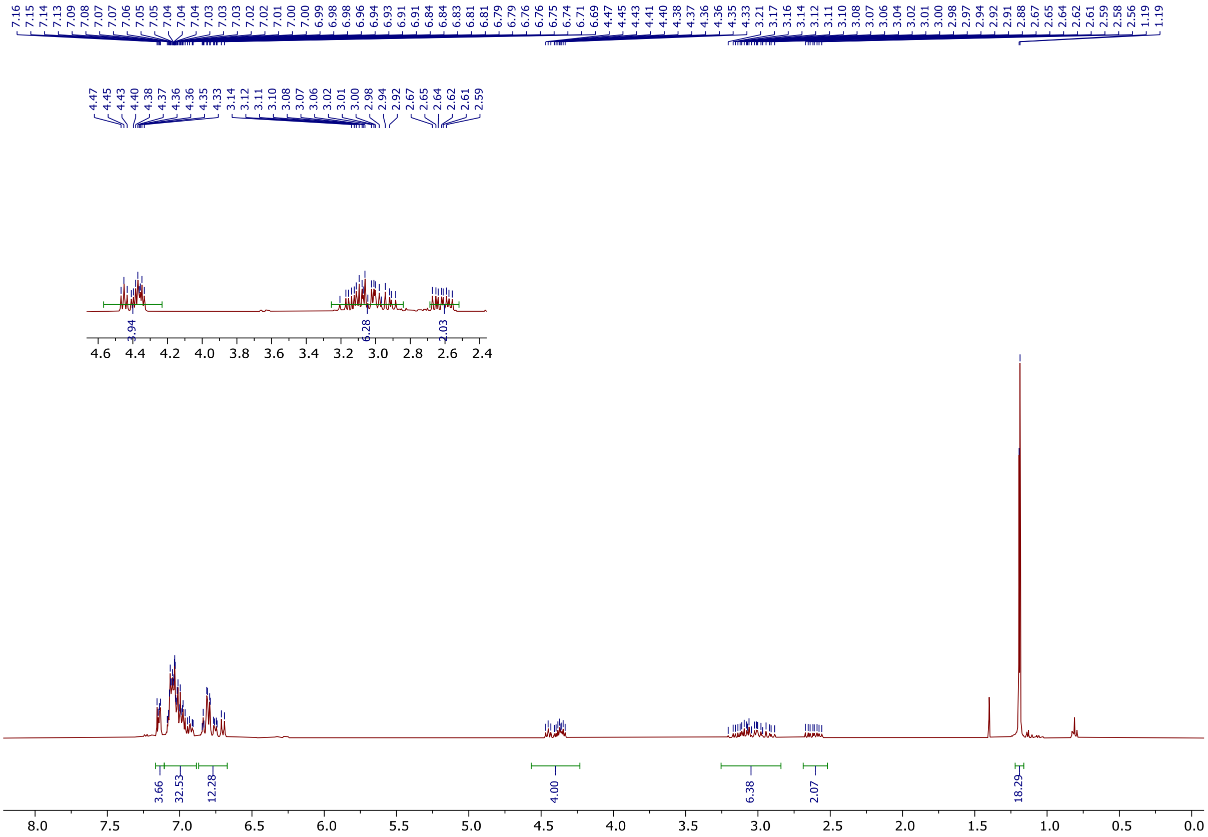

Figure S107: ^{1}H NMR Spectrum of **13** in $CDCl_3$ after isolation via column chromatography.

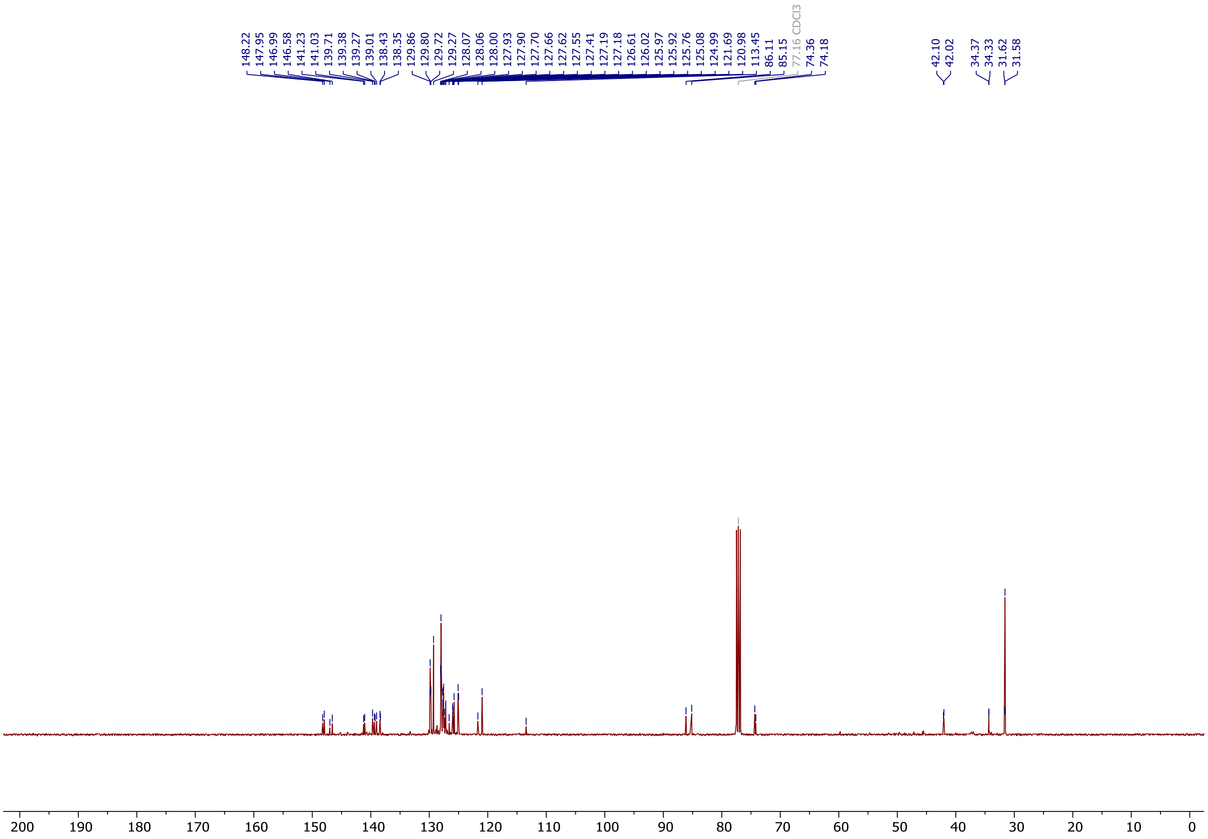

Figure S108: ^{13}C NMR Spectrum of **13** in $CDCl_3$ after isolation via column chromatography.

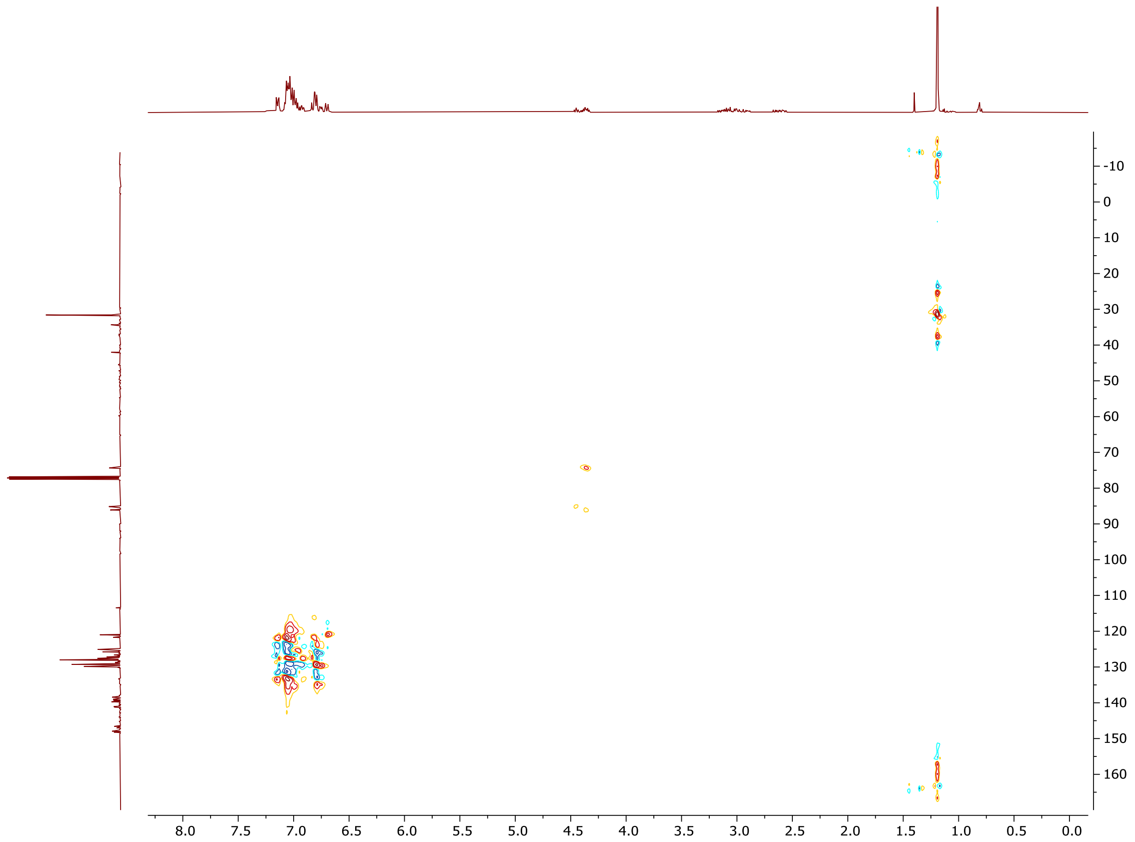

Figure S109: ^{1}H-^{13}C HSQC NMR Spectrum of **13** in $CDCl_3$ after isolation via column chromatography.

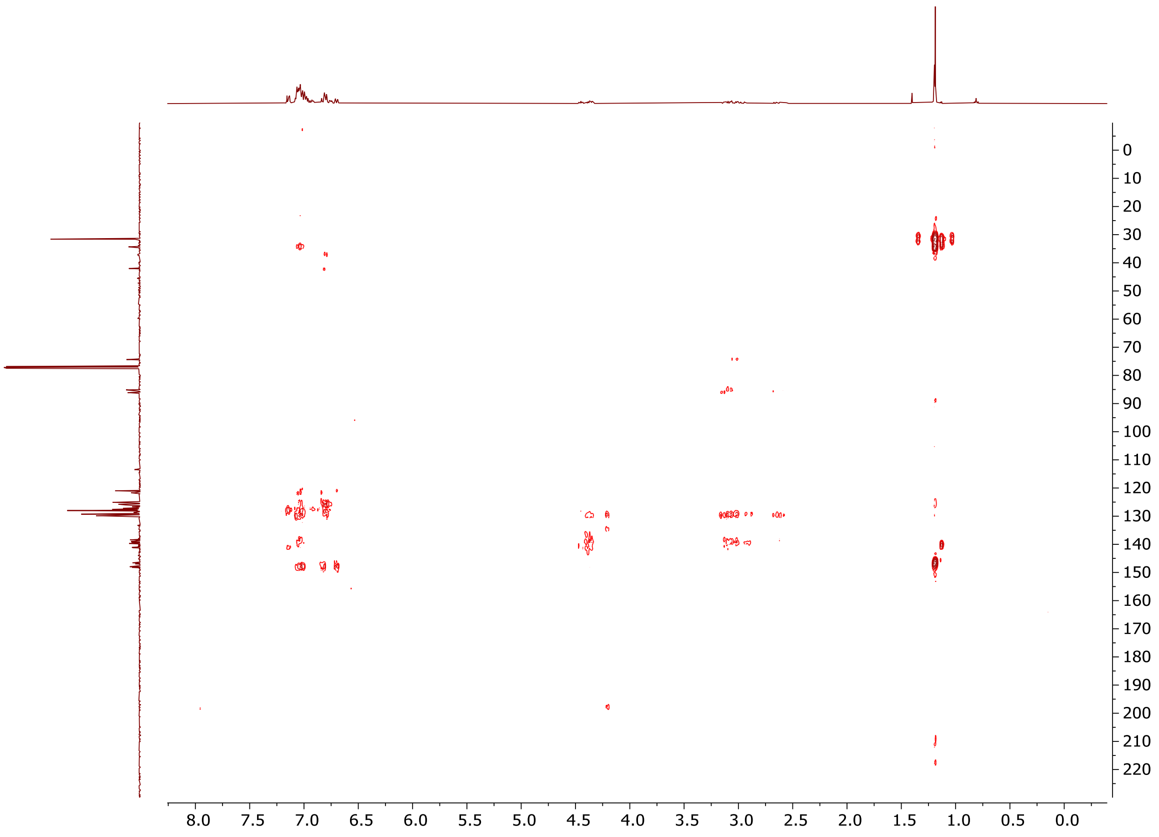

Figure S110: ^{1}H-^{13}C HMBC NMR Spectrum of **13** in $CDCl_3$ after isolation via column chromatography.

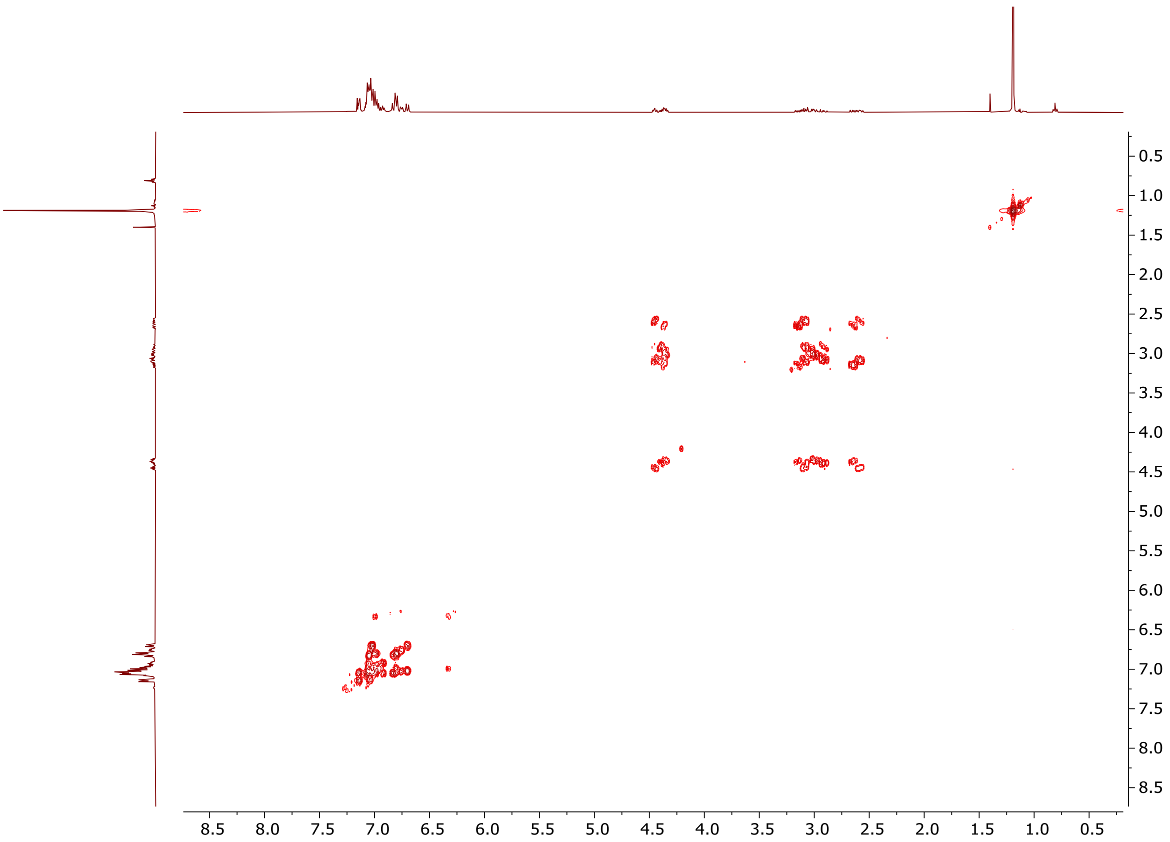

Figure S111: ^{1}H-^{1}H COSY NMR Spectrum of **13** in $CDCl_3$ after isolation via column chromatography.

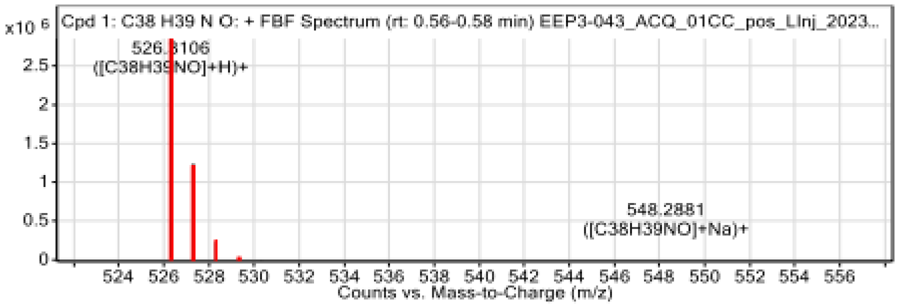

Figure S112: HRMS spectra of compound **13**.

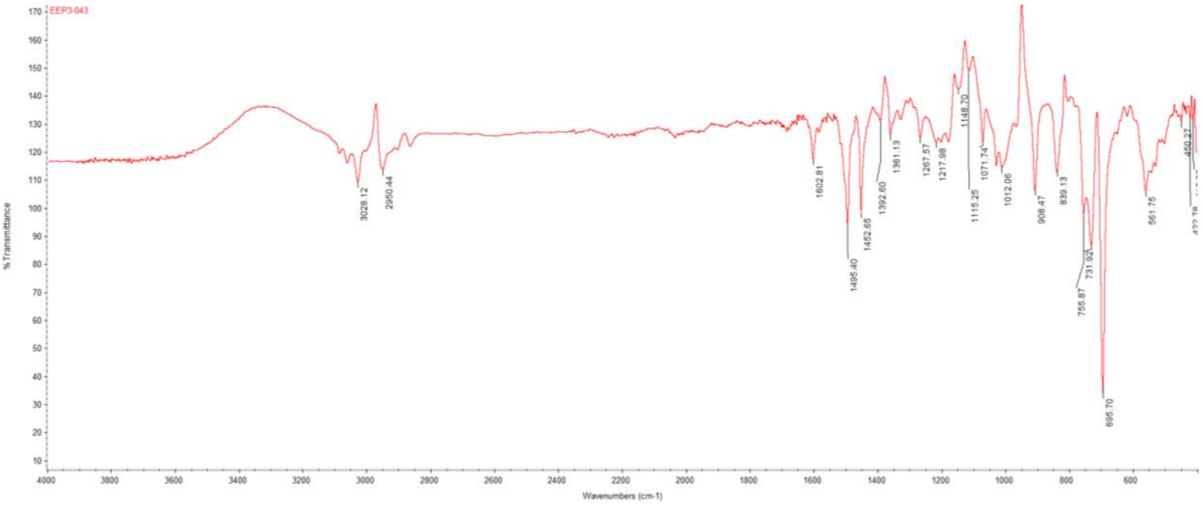

Figure S113: IR spectra for compound **13**.

3.1.13. 4-(tert-butyl)-N-(1-(4-methoxyphenyl)-2-phenylethyl)aniline
Mixture of Regioisomers

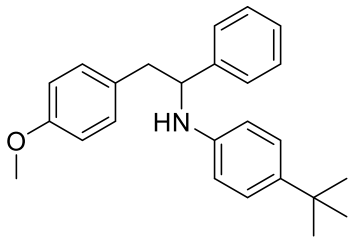

&

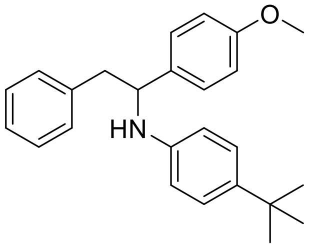

Colourless oil

0.1566g, yield = 57% (0.76 mmol scale)

R_f = 0.35 (20% DCM / 80% hexane)

^{1}H NMR ($CDCl_3$, 400 MHz): δ 7.28 – 7.09 (m, 5H), 6.99 (ddd, J = 18.3, 15.8, 7.8 Hz, 4H), 6.74 (dd, J = 16.1, 8.1 Hz, 2H), 6.33 (dd, J = 8.9, 3.5 Hz, 2H), 4.40 (t, J = 7.1 Hz, 1H), 3.91 (s, 1H), 3.69 (dd, J = 3.5, 1.0 Hz, 3H), 2.91 (dddd, J = 43.6, 21.9, 14.0, 7.1 Hz, 2H), 1.13 (d, J = 1.5 Hz, 8H).

^{13}C{^{1}H} NMR ($CDCl_3$, 126 MHz): δ 158.70, 158.51, 145.20, 144.07, 140.23, 140.20, 138.08, 135.96, 130.31, 129.92, 129.36, 128.67, 128.64, 127.63, 127.08, 126.75, 126.63, 125.90, 114.08, 114.05, 113.46, 59.87, 59.17, 55.36, 55.34, 45.59, 44.63, 33.90, 31.62.

HRMS (ESI+): calcd for [M, $C_{25}H_{30}NO]^+$ 360.2322, found 360.2319.

IR (Neat): 2957.1, 2862.9, 1612.7, 1510.6, 1320.5, 1243.8, 1177.1, 819.2 cm^{-1}.

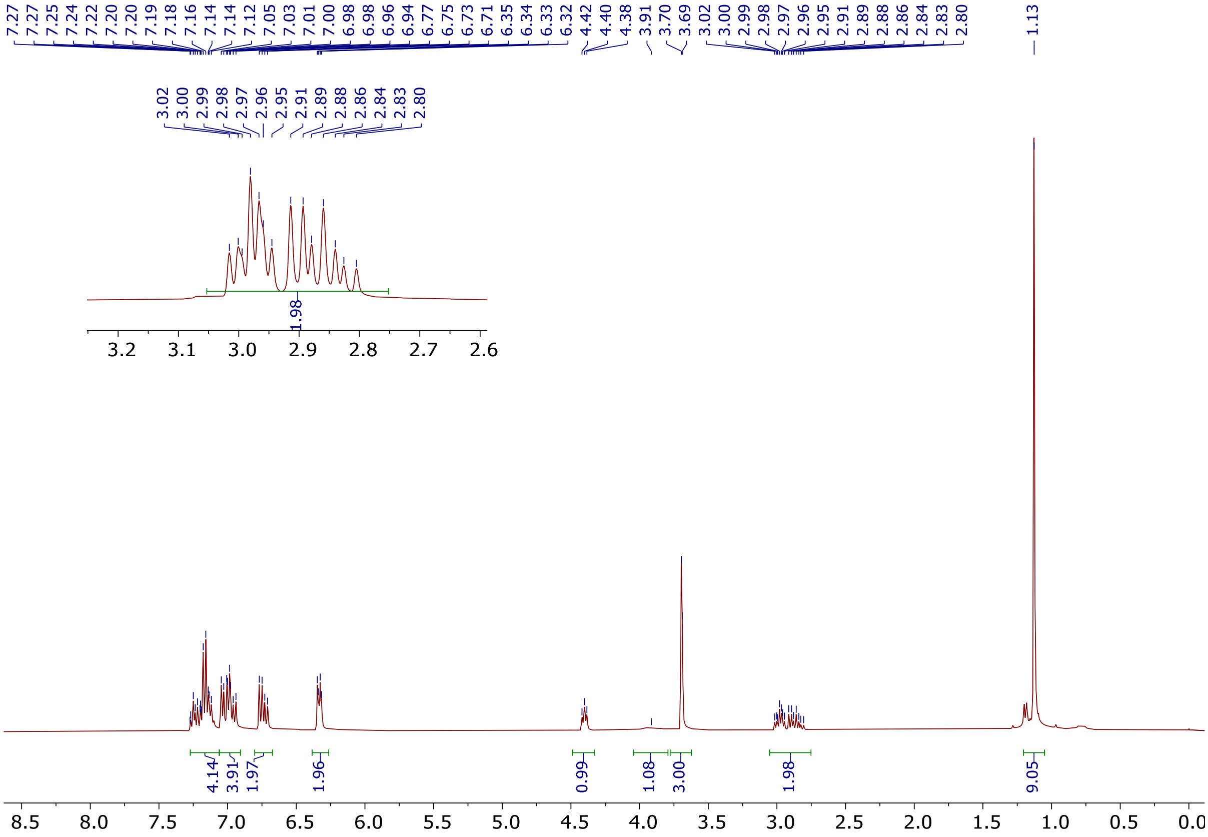

Figure S114: ^{1}H NMR Spectrum of *4-(tert-butyl)-N-(1-(4-methoxyphenyl)-2-phenylethyl)aniline* in $CDCl_3$ after isolation via column chromatography.

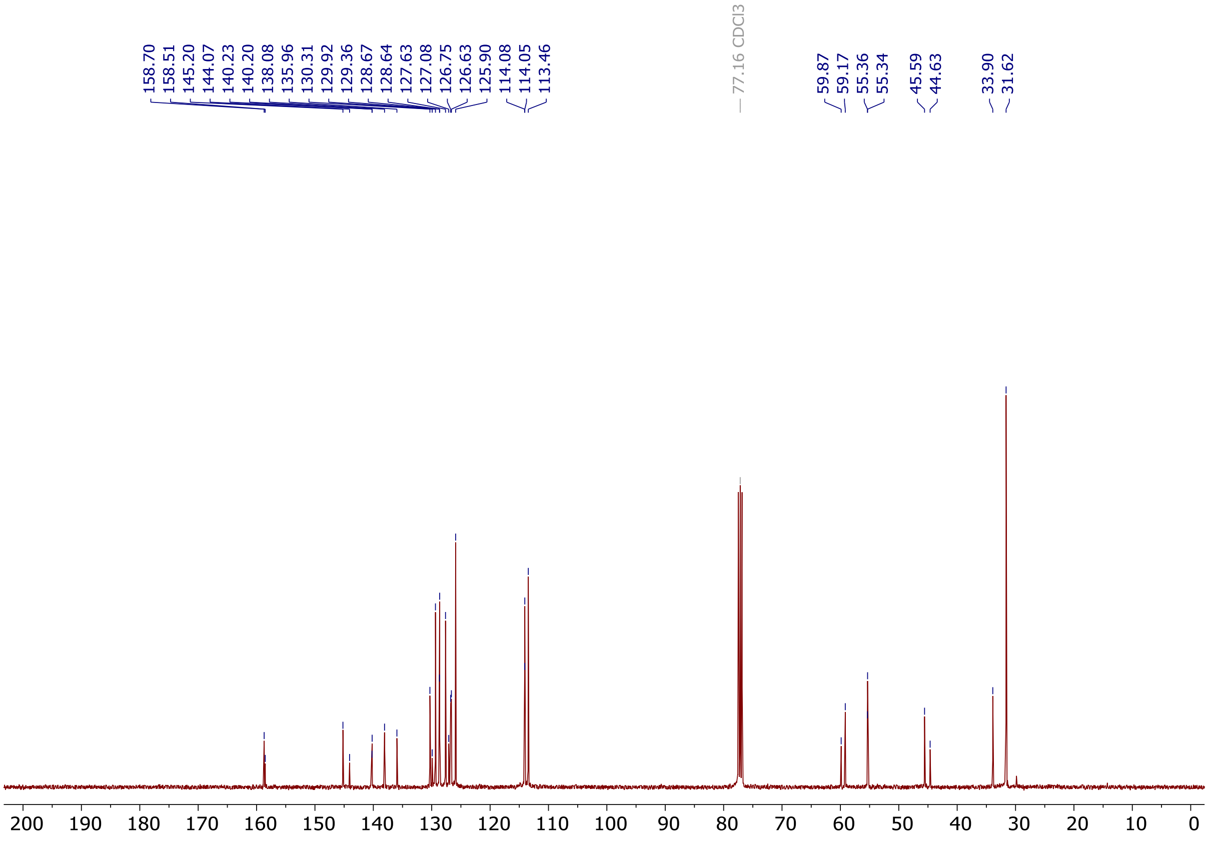

Figure S115: ^{13}C NMR Spectrum of *4-(tert-butyl)-N-(1-(4-methoxyphenyl)-2-phenylethyl)aniline* in $CDCl_3$ after isolation via column chromatography.

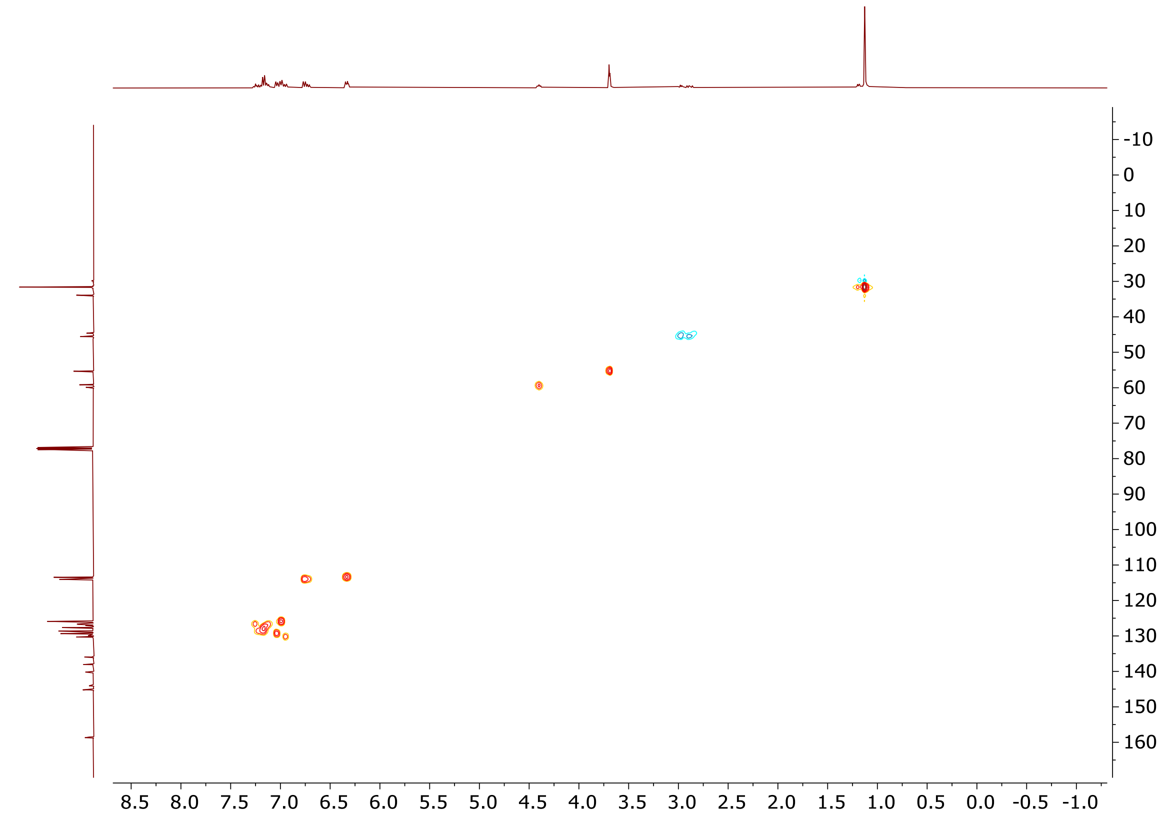

Figure S116: ^{1}H-^{13}C HSQC NMR Spectrum of *4-(tert-butyl)-N-(1-(4-methoxyphenyl)-2-phenylethyl)aniline* in $CDCl_3$ after isolation via column chromatography.

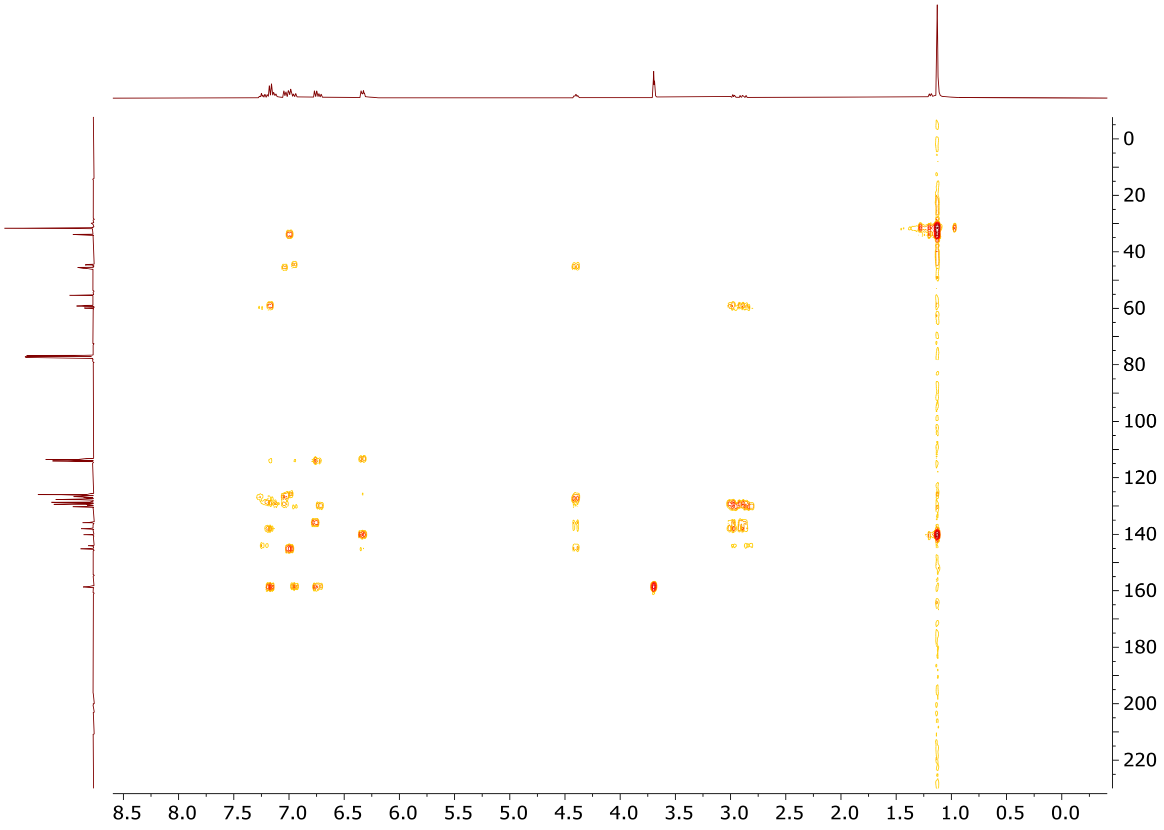

Figure S117: ^{1}H-^{13}C HMBC NMR Spectrum of *4-(tert-butyl)-N-(1-(4-methoxyphenyl)-2-phenylethyl)aniline* in $CDCl_3$ after isolation via column chromatography.

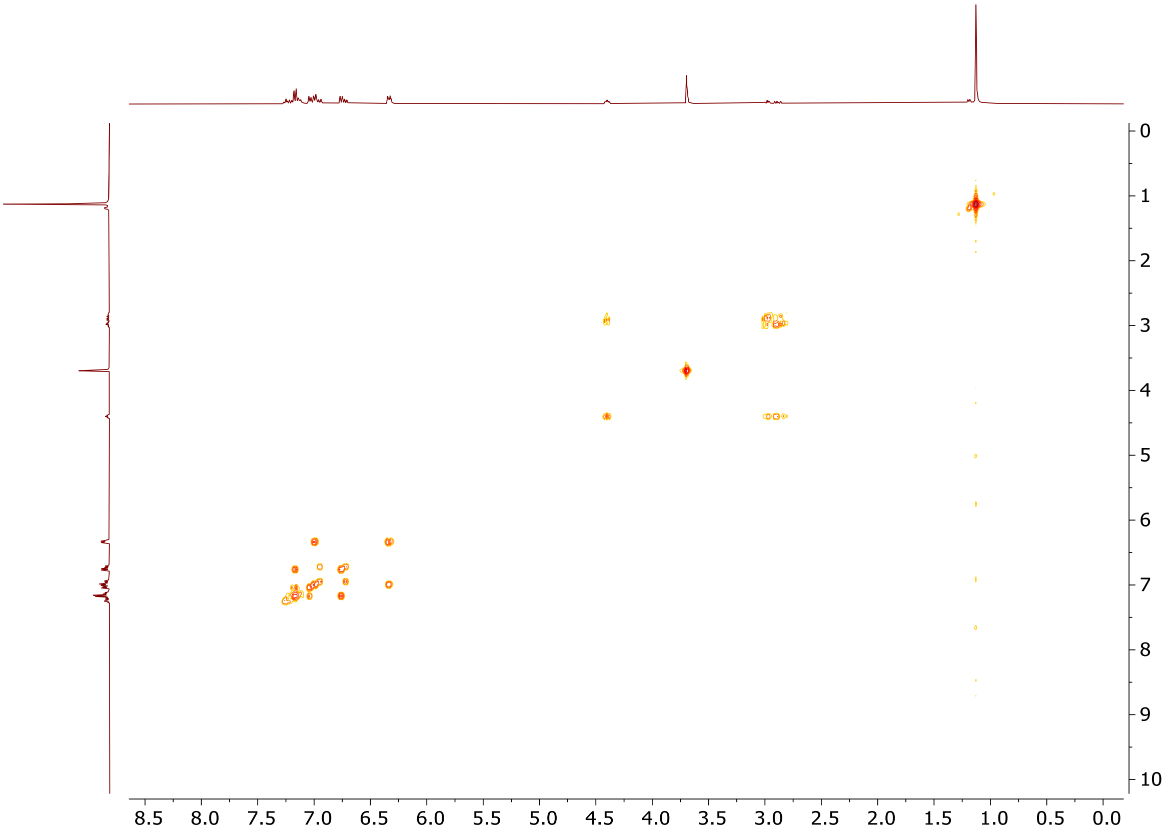

Figure S118: ^{1}H-^{1}H COSY NMR Spectrum of *4-(tert-butyl)-N-(1-(4-methoxyphenyl)-2-phenylethyl)aniline* in $CDCl_3$ after isolation via column chromatography.

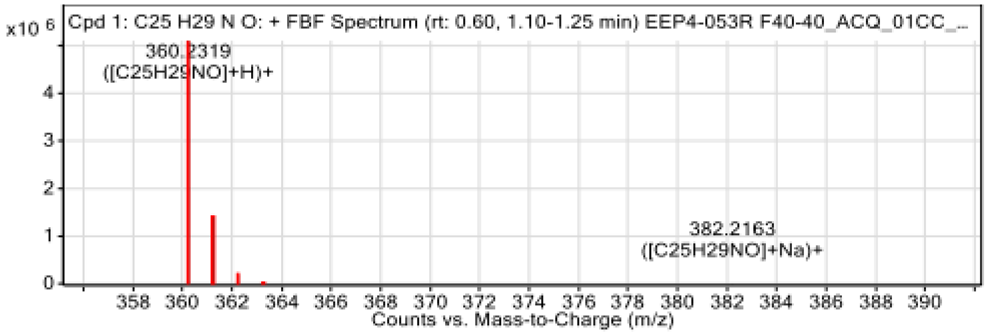

Figure S119: HRMS spectra for *4-(tert-butyl)-N-(1-(4-methoxyphenyl)-2-phenylethyl)aniline.*

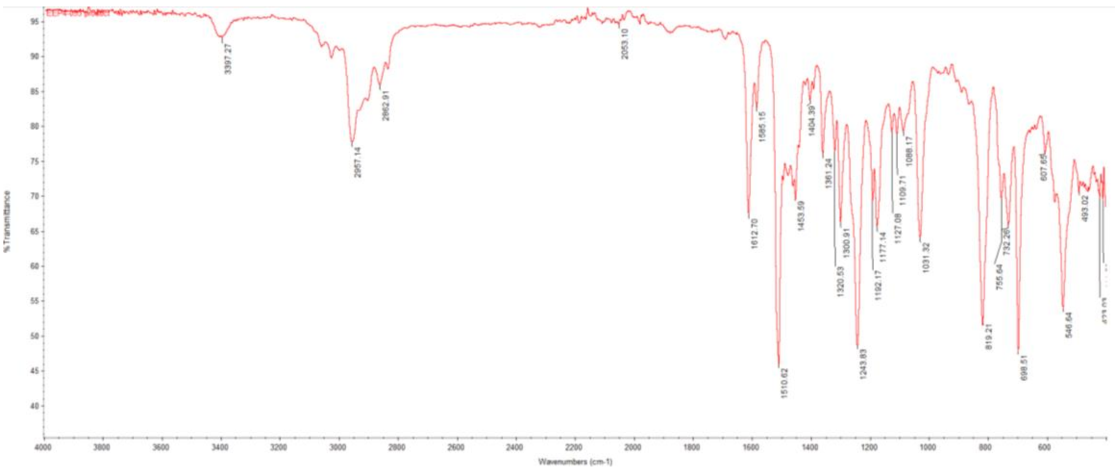

Figure S120: IR spectra for *4-(tert-butyl)-N-(1-(4-methoxyphenyl)-2-phenylethyl)aniline.*

3.1.14. 4-(tert-butyl)-N-(1,2-di-p-tolylethyl)aniline

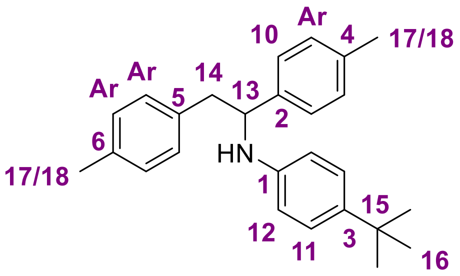

Yellow oil

0.129 g, yield = 36%

R_f = 0.41 (20% DCM / 80% hexane)

^{1}H NMR ($CDCl_3$, 400 MHz): δ 7.30 (d, *J* = 8.1 Hz, 2H, C^{10}-H), 7.17 (d, *J* = 8.0 Hz, 2H, Ar-H), 7.14 – 7.06 (m, 6H, Ar-H, C^{11}-H), 6.45 (d, *J* = 8.7 Hz, 2H, C^{12}-H), 4.52 (dd, *J* = 8.7, 5.3 Hz, 1H, C^{13}-H), 4.06 (s, 1H, NH), 3.11 (dd, *J* = 14.0, 5.3 Hz, 1H, C^{14}-H), 2.95 (dd, *J* = 14.0, 8.6 Hz, 1H, C^{14}-H), 2.38 (s, 3H, $C^{17/18}$-H), 2.35 (s, 3H, $C^{17/18}$-H), 1.25 (s, 9H, C^{16}-H).

^{13}C{^{1}H} NMR ($CDCl_3$, 126 MHz): δ 145.3 (C^1), 141.2 (C^2), 140.1 (C^3), 136.6 (C^4), 136.7 (C^5), 135.0 (C^6), 129.4 (Ar), 129.4 (Ar), 129.2 (Ar), 126.5 (C^{10}), 125.9 (C^{11}), 113.4 (C^{12}), 59.5 (C^{13}), 45.2 (C^{14}), 33.9 (C^{15}), 31.6 (C^{16}), 21.5 (C^{17}), 21.2 (C^{18}).

HRMS (ESI+): calcd for [M, $C_{26}H_{32}N]^+$ 358.2529, found 358.2529.

IR (Neat): 2952.3, 2920.7, 1614.9, 1514.3, 1301.5, 814.5 cm^{-1}.

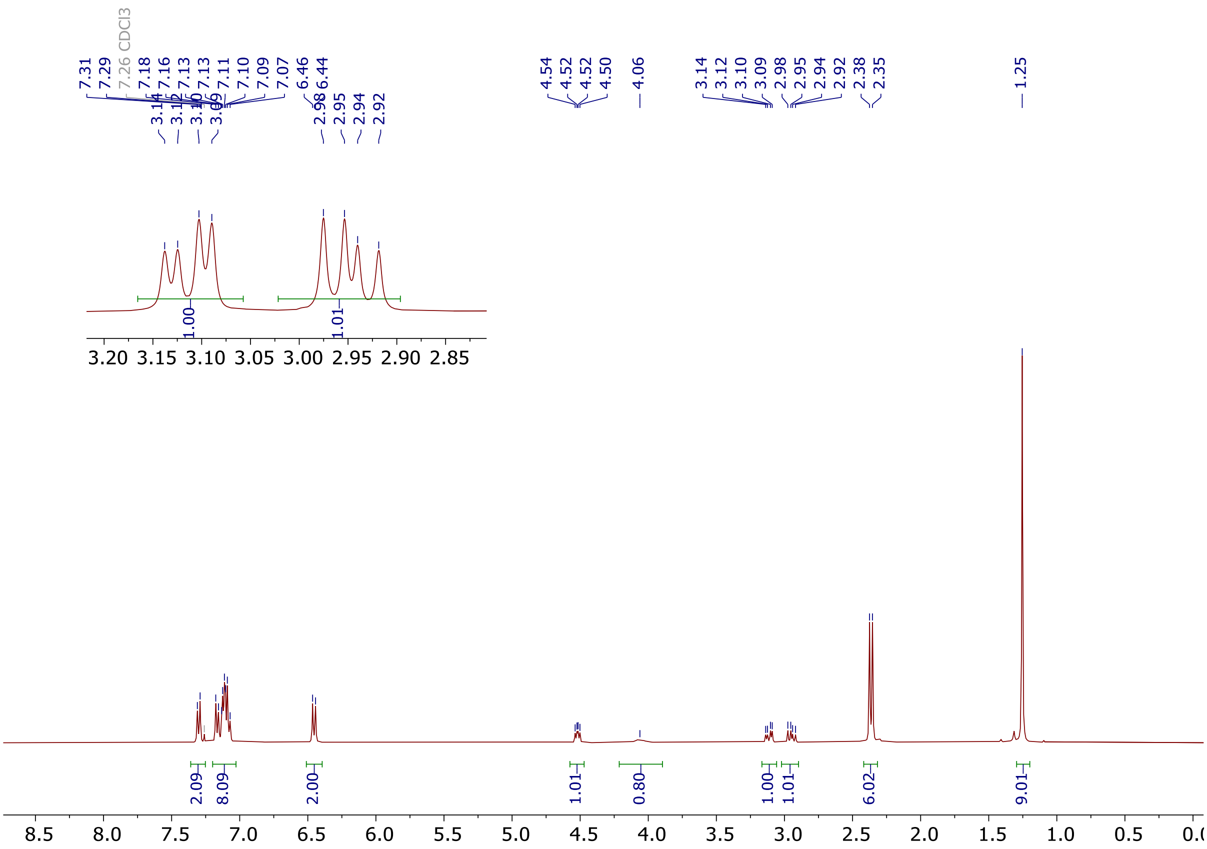

Figure S121: ^{1}H NMR Spectrum of *4-(tert-butyl)-N-(1,2-di-p-tolylethyl)aniline* in $CDCl_3$ after isolation via column chromatography.

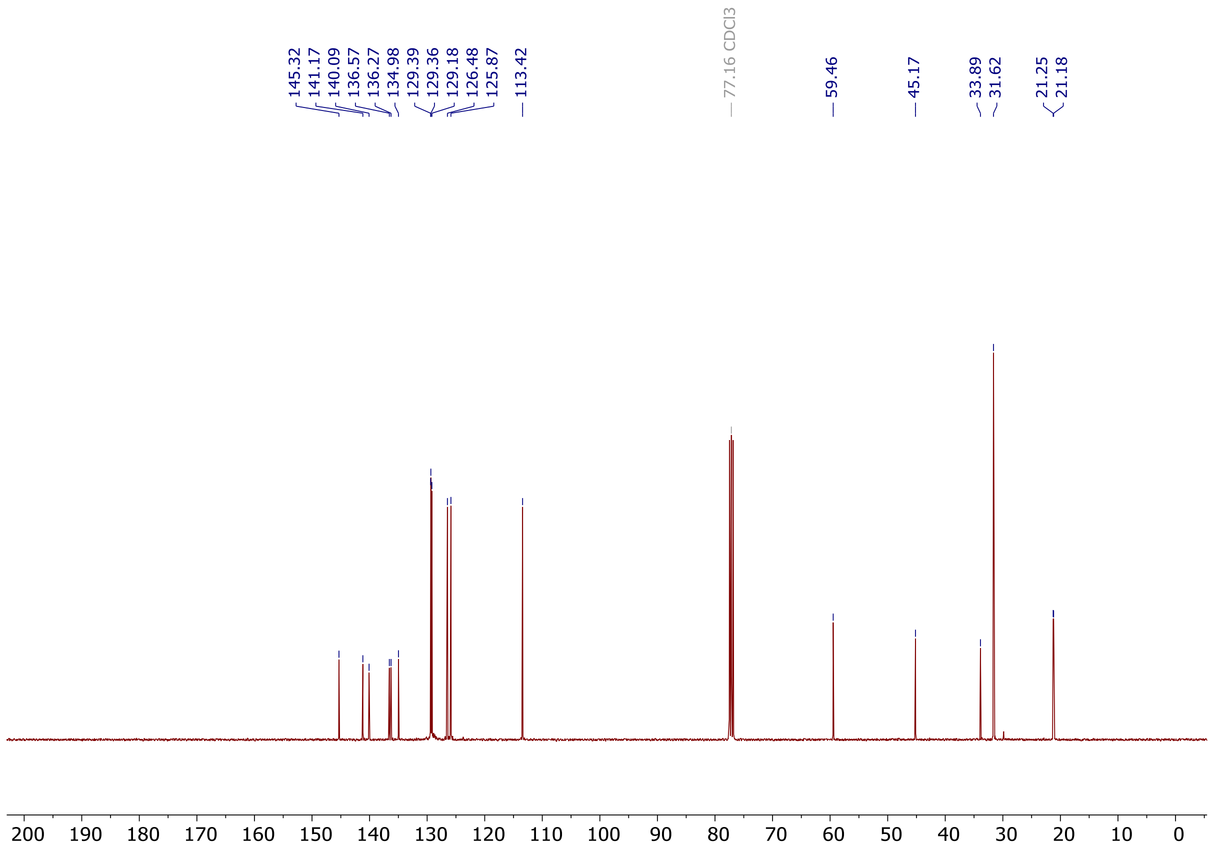

Figure S122: ^{13}C NMR Spectrum of *4-(tert-butyl)-N-(1,2-di-p-tolylethyl)aniline* in $CDCl_3$ after isolation via column chromatography.

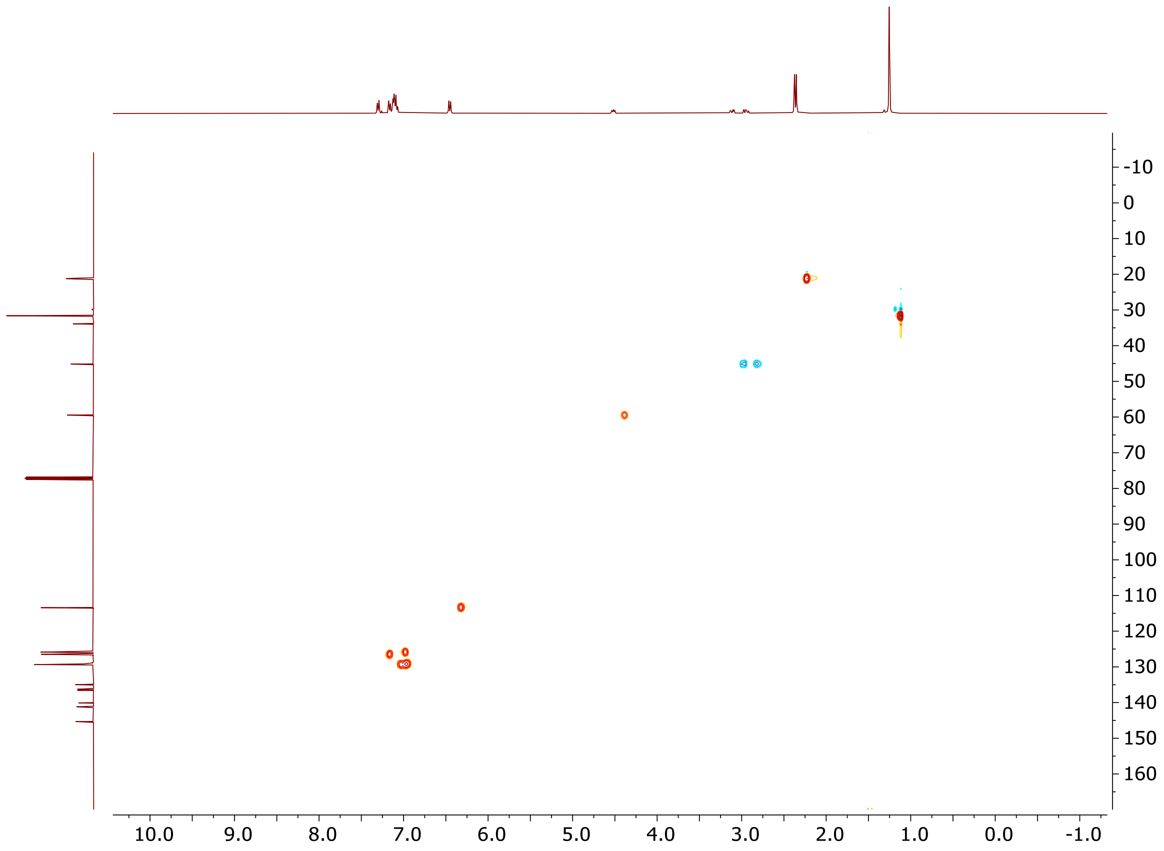

Figure S123: ^{1}H-^{13}C HSQC NMR Spectrum of *4-(tert-butyl)-N-(1,2-di-p-tolylethyl)aniline* in $CDCl_3$ after isolation via column chromatography.

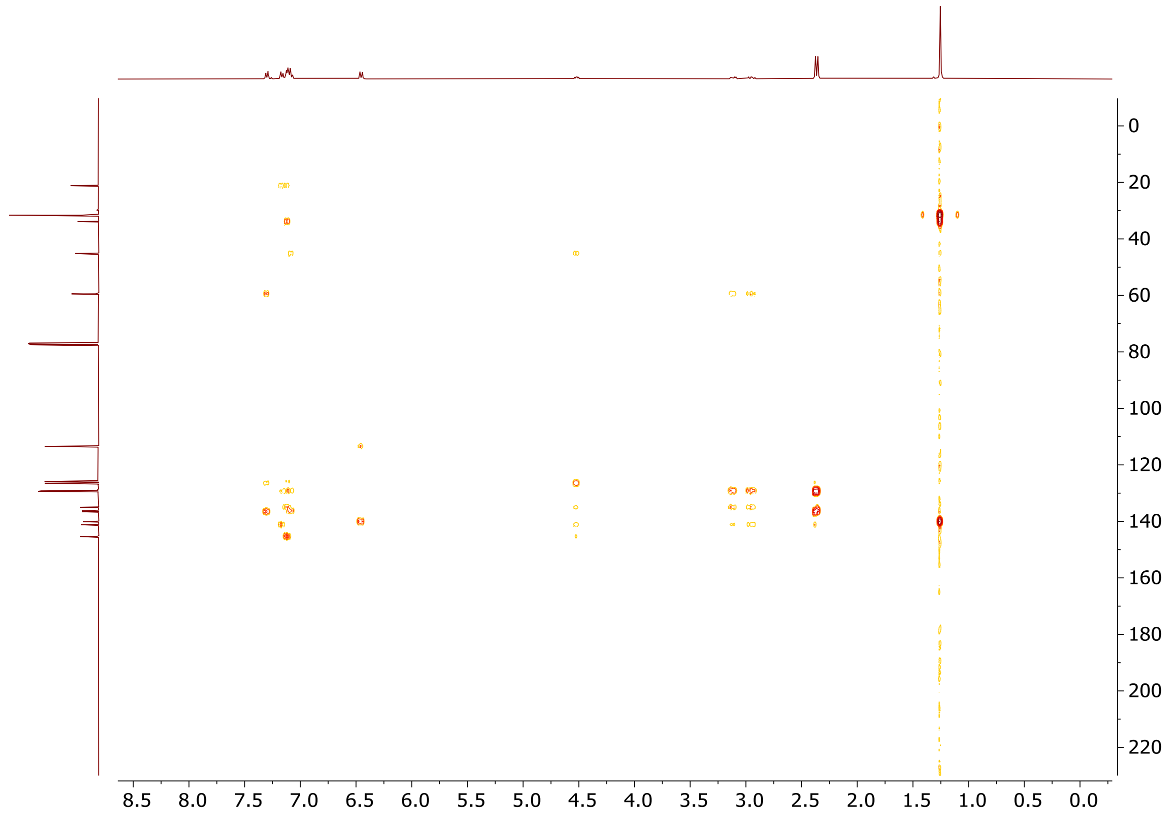

Figure S124: ^{1}H-^{13}C HMBC NMR Spectrum of *4-(tert-butyl)-N-(1,2-di-p-tolylethyl)aniline* in $CDCl_3$ after isolation via column chromatography.

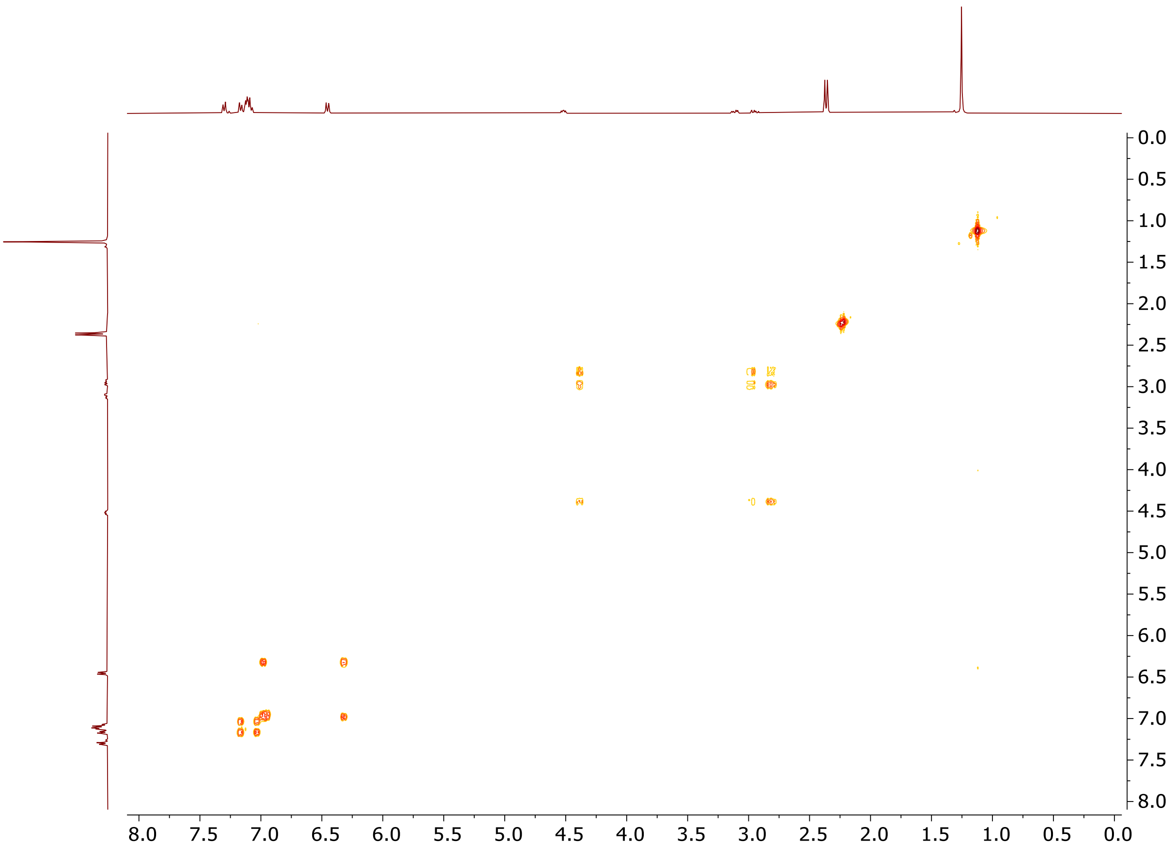

Figure S125: ^{1}H-^{1}H COSY NMR Spectrum of *4-(tert-butyl)-N-(1,2-di-p-tolylethyl)aniline* in $CDCl_3$ after isolation via column chromatography.

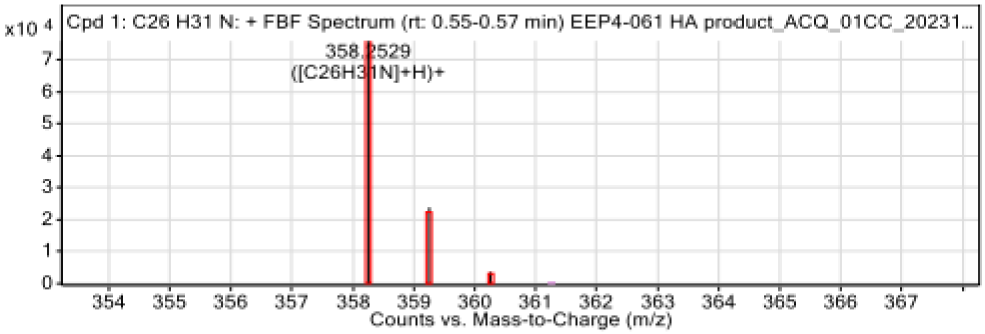

Figure S126: HRMS spectra for *4-(tert-butyl)-N-(1,2-di-p-tolylethyl)aniline.*

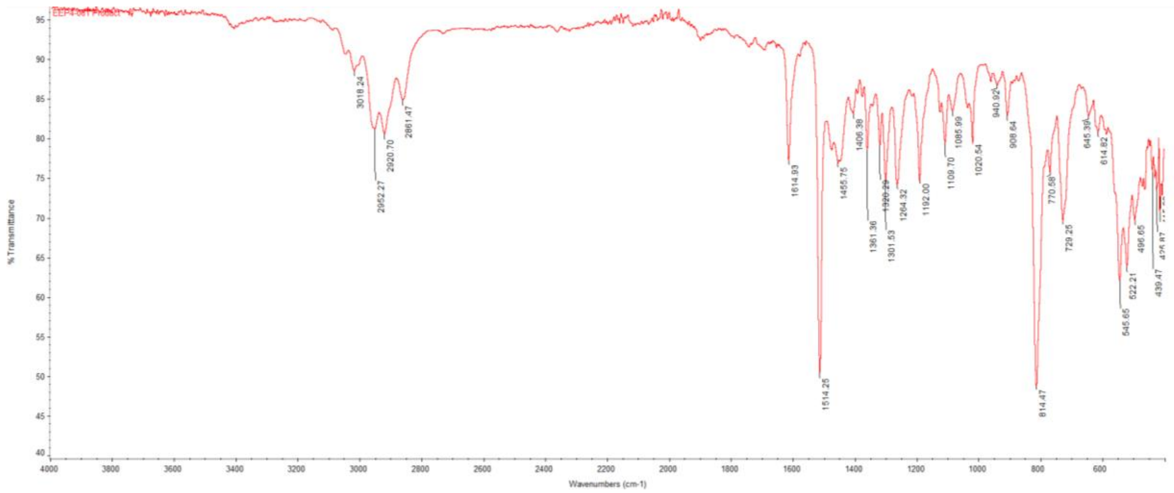

Figure S127: IR spectra for *4-(tert-butyl)-N-(1,2-di-p-tolylethyl)aniline.*

4. Quantum-Chemical Calculations

4.1. General Considerations Computational Methods

Quantum chemical calculations were carried out with the Gaussian 16 programme.[5] With Gaussian 16, molecular geometries were optimized at the density functional theory (DFT) level employing the generalized gradient approximation (GGA) via the PBE[6] functional in conjunction with the D3 atom-pairwise dispersion correction without damping[7] and an implicit polarizable continuum solvent model[8] utilizing acetonitrile as the solvent. The split-valence double-zeta def2-SVP basis set[9] was used together with the corresponding auxiliary Coulomb-fitting basis set of Weigend.[10] At this GGA DFT level, abbreviated as PBE-D3(PCM)/def2-SVP, frequency calculations were performed on the optimized stationary points to characterize minima and transition structures, and to extract thermal contributions to enthalpies and Gibbs energies at 298.15 K. For improved relative energies single-point energy calculations were performed at the hybrid DFT level with 25 % admixture of Fock-exchange via the PBE0[11, 12] functional employing the triple-zeta valence polarized def2-TZVP basis set[9] and the same dispersion and solvent corrections as above. The final relative Gibbs energies and orbital eigenvalues are thus reported at the PBE0-D3(PCM)/def2-TZVP // PBE-D3(PCM)/def2-SVP level.

5. One-Pot Hydroaminations Quantum Chemical Calculations

5.1. Summary of SOMO energies and BDFE trends for catalysts 2a to 2c and 14a to 14c

The SOMO eigenvalues were calculated (PBE0-D3/def2-TZVP//PBE-D3/def2-SVP including an implicit solvation model for acetonitrile; see below for SOMO isosurface plots). The (highest) SOMO is always partly localized on the Fe–H moiety in all three species **2a** to **2c**, where the molecular orbital coefficient on the hydride is largest in **2b** and smallest in **2c**. A better SOMO-LUMO overlap during the key HAT step should manifest itself in improved HA product selectivity. Our calculations show that the SOMO values decrease as electron density decreases at the iron centre OMe > H > CF_3 (*i.e.* **2a** = −6.08 eV; **2b** = −6.35 eV; **2c** = −5.70 eV. See below). The ordering of these data match the UV-vis and cyclic voltammetry data obtained for **1a** to **1c** (see manuscript, Table 2). We can assume that the smallest SOMO-LUMO gap between the iron-hydride and the alkene does not necessarily lead to the highest yield of HA product, but instead there is an optimal SOMO-LUMO gap. We could hypothesize that this is the SOMO-LUMO gap that exists between **2b** and α-methyl styrene (where the gap is 5.19 eV and 97% combined yield of **7** and **8** is obtained). However, the magnitude of the gap does not explain why, for example, 89% **7** and **8** is obtained with **1c** (where the **2c** SOMO-LUMO gap is 4.54 eV) or why there is only 60% **3** and **4** when **1c** is employed (and the SOMO-LUMO gap is closer to the hypothesized optimal value at 4.79 eV).

We also considered that the HAT event from the compounds **2** to the olefin or the nitro is most likely to be affected by a change in the electronics of the iron. Calculating the homolytic BDFE energy for the loss of the hydrogen atom would give us another way to explore how the trends in our data could be explained.[2] To determine the BDFE for each catalyst first the spin state for the corresponding Fe^{II}(salen) complexes was determined (**14a** to **14c**, see manuscript Table 8), which confirmed the HS configuration as the ground state

The homolytic BDFE (Fe–H) for **2a** to **2c** is computed with respect to the ground state energies of the corresponding Fe(II) species **14a** to **14c** and the energy of the hydrogen atom (see the Supporting Information). The catalyst possessing the *p*-CF_3 group **2b** has the smallest BDFE energy (17.8 kcal mol^{-1} compared to 18.7 kcal mol^{-1} for **2a** and 19.4 kcal mol^{-1} for **2c**). This suggests that it is easiest for this catalyst to lose its hydride as a hydrogen radical. This change would have an effect in multiple steps within the catalytic cycle, including the HAT step that generates an alkyl radical. It could also affect the hydride transfer event to the nitro, which results in the formation of the nitrosoThe differences in BDFE for **2a** to **2c** are small, approximately 0.9 kcal mol^{-1}. However, by considering the HAT reactions of both **2a** and **2b** as competing reactions the difference in BDFE could be used to calculate a theoretical equilibrium constant. In doing this a value for K is obtained, K = 4.5. As the theoretical equilibrium constant is > 1 it indicates that the *p*CF_3 derivative is favors cleavage of the Fe–H bond. Compared to **1a**, qualitatively, pre-catalyst **1b** could favor nitroso formation and the generation of the alkyl radical, and with these two processes working complimentary to one another, the HA products might be favored.

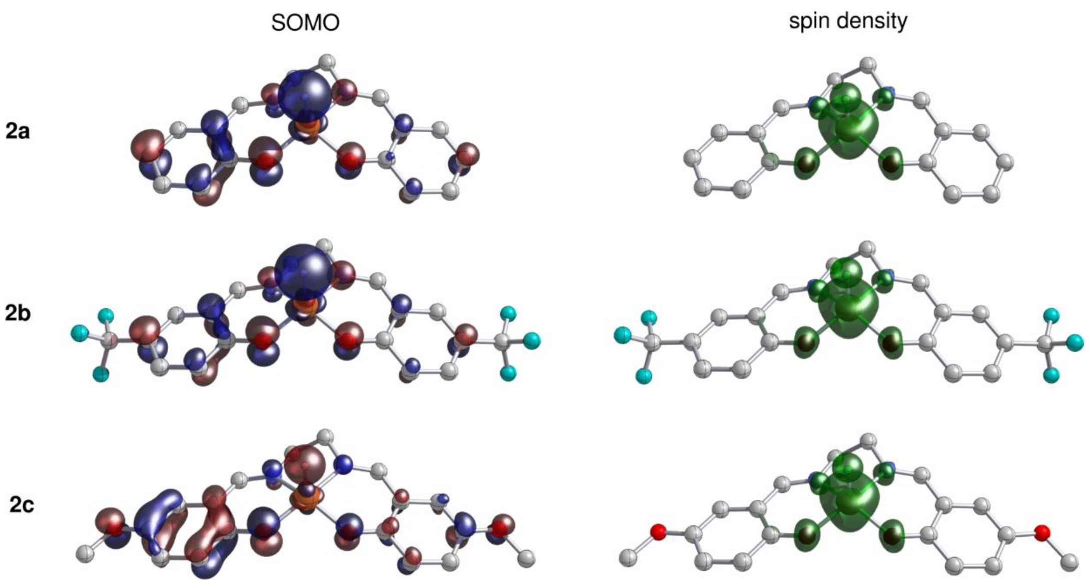

Figure S128: Isosurface plots of the highest singly occupied molecular orbital (SOMO isovalue 0.05 a_0^{-3/2}) and of the spin density (isovalue 0.01 a_0^{-3}) for species 2a-2c, computed at the PBE0-D3(PCM)/def2-TZVP level.

Table S1: Comparison of (highest) SOMO eigenvalues and total energies for **2a** to **2c** on the HS surface.

Species	⟨S^2⟩	E_{tot}	H_{tot}^{298}	G_{tot}^{298}	SOMO/ eV
**2a**	8.762	-2141.572241	0.287305	0.218769	- 6.08 eV
**2b**	8.761	-2815.339667	0.303285	0.211468	- 6.35 eV
**2c**	8.763	-2370.458873	0.354932	0.273799	- 5.70 eV

Table S2: Comparison of BDFE (Fe–H) energies for the Fe^{II}(salen) species (**14a** to **14c**) on the HS surface.

Species	⟨S^2⟩	E_{tot}	H_{tot}^{298}	G_{tot}^{298}	BDFE/ kcal mol^{-1}
**14a**	6.033	-2141.027 249	0.280 768	0.215 265	18.7
**14b**	6.032	-2814.7981	0.29698	0.209938	17.8
**14c**	6.036	-2369.912454	0.348494	0.269982	19.4

Table S3: Comparison of Spin expectation values and total energies (Hartree) for the corresponding iron II species of **1a-1c** on the high-spin surface and their corresponding BDFE energies.

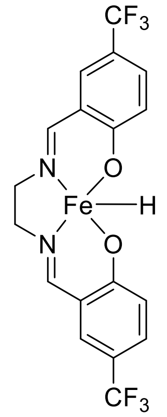

2b

PBE0-D3(Acetonitrile)/def2TZVP // PBE-D3(Acetonitrile)/def2SVP/W06							
$State$	$\langle S^2 \rangle$	E_{tot}	H_{tot}^{298}	G_{tot}^{298}	E_{rel}	H_{rel}	G_{rel}
HS (Sextet)	8.761	-2815.339667	0.303285	0.211468	0	0	0
IS (quartet)	3.869	-2815.330748	0.304323	0.217154	5.6	6.25	9.16
LS (doublet)	0.8	-2815.340973	0.306188	0.221783	-0.82	1	5.65

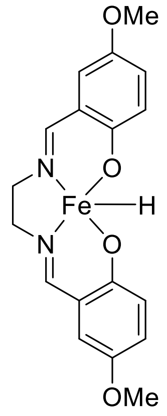

2c

PBE0-D3(Acetonitrile)/def2TZVP // PBE-D3(Acetonitrile)/def2SVP/W06							
$State$	$\langle S^2 \rangle$	E_{tot}	H_{tot}^{298}	G_{tot}^{298}	E_{rel}	H_{rel}	G_{rel}
HS (Sextet)	8.763	-2370.458873	0.354932	0.273799	0	0	0

IS (quartet)	3.814	-2370.447571	0.355959	0.27762	7.09	7.74	9.49
LS (doublet)	0.802	-2370.460972	0.357811	0.282352	-1.32	0.49	4.05

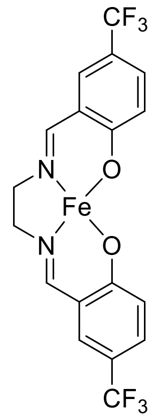

14b

PBE0-D3(Acetonitrile)/def2TZVP // PBE-D3(Acetonitrile)/def2SVP/W06							
$State$	$\langle S^2 \rangle$	E_{tot}	H_{tot}^{298}	G_{tot}^{298}	E_{rel}	H_{rel}	G_{rel}
HS (quintet)	6.032	-2814.7981	0.29698	0.209938	0	0	0
IS (triplet)	2.035	-2814.788782	0.298279	0.213276	5.85	6.66	7.94
LS (singlet)	0	-2814.726408	0.298277	0.214224	44.99	45.8	47.68

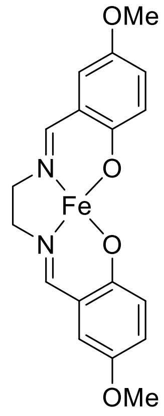

14c

PBE0-D3(Acetonitrile)/def2TZVP // PBE-D3(Acetonitrile)/def2SVP/W06							
$State$	$\langle S^2 \rangle$	E_{tot}	H_{tot}^{298}	G_{tot}^{298}	E_{rel}	H_{rel}	G_{rel}
HS (quintet)	6.036	-2369.912454	0.348494	0.269982	0	0	0
IS (triplet)	2.039	-2369.903448	0.349808	0.27333	5.65	6.48	7.75
LS (singlet)	0	-2369.848854	0.350103	0.275759	39.91	40.92	43.53

^{6}[Fe] 2b

E_{tot} (PBE0-D3(Acetonitrile)/def2TZVP // PBE-D3(Acetonitrile)/def2SVP/W06) = -2815.3396671900

$\langle S^2 \rangle$ = *8.7610*

Fe	0.014269987151	0.239868869864	-0.804421848485
O	1.481655964282	-0.988116157464	-0.474052848485
O	-1.449629035086	-0.954157102873	-0.393966848485
N	-1.355436983489	1.816380895373	-0.380619848485
N	1.257312014729	1.720694846715	0.125187151515
C	2.543772012936	1.624411822756	0.301843151515
H	3.097197029594	2.518899812450	0.662636151515
C	3.342830990846	0.438289807875	0.096888151515
C	4.740682992575	0.531103781843	0.309966151515
H	5.172903010683	1.503454773793	0.590135151515
C	5.569829971862	-0.581088233599	0.175199151515
C	5.000919948532	-1.833823223004	-0.172734848485
H	5.648792932068	-2.717905235070	-0.276197848485
C	3.633609946228	-1.957518197540	-0.381308848485
H	3.185213928208	-2.925126189189	-0.649244848485
C	2.757031967156	-0.833772181215	-0.263924848485
C	-2.731087032373	-0.808479079008	-0.204593848485
C	-3.557639054012	-1.970403063615	-0.098662848485
H	-3.067275072271	-2.950847072747	-0.184562848485
C	-4.927933052134	-1.869590038096	0.102723151515
H	-5.534984069180	-2.784882026790	0.178012151515
C	-5.551003028488	-0.599865026492	0.215537151515
C	-4.772221007027	0.552472959005	0.123974151515
H	-5.246302988613	1.541213967834	0.215720151515
C	-3.373311008339	0.482049932952	-0.087504848485
C	-2.631189985263	1.721121919131	-0.155477848485
H	-3.218285967922	2.652256930065	0.003434151515
C	-0.678423959523	3.103238882764	-0.387112848485
H	-1.327458944144	3.929061894852	-0.025270848485
H	-0.375121955247	3.332837877116	-1.432511848485
C	0.572381037839	2.961606859470	0.484954151515
H	1.235247054338	3.847539847125	0.386142151515
H	0.260954036462	2.887651865270	1.550430151515
H	0.084043991466	0.471583868565	-2.470413848485
C	7.053723973665	-0.484280261234	0.393178151515
F	7.447956996870	0.761757731424	0.750568151515
F	7.476712957825	-1.334846269112	1.368799151515
F	7.751681967422	-0.819510274232	-0.727194848485
C	-7.037743027134	-0.527194998804	0.423640151515
F	-7.481364003459	0.744084009458	0.574714151515
F	-7.721447037200	-1.067675986071	-0.623064848485
F	-7.426949040094	-1.223071991556	1.527217151515

[4][Fe] 2b

E_{tot} (PBE0-D3(Acetonitrile)/def2TZVP // PBE-D3(Acetonitrile)/def2SVP/W06) = -2815.3307482500

$\langle S^2 \rangle$ = *3.8695*

Fe	-0.010933560779	0.296816715311	0.526674405859
O	-1.427023214630	-0.901532645392	0.517281881895
O	1.391620869621	-0.990510916727	0.124439426441
N	1.272587003917	1.735959128420	0.445986494495
N	-1.236245661563	1.720305030665	-0.306705926885
C	-2.535452212572	1.650281784638	-0.417175918065
H	-3.098917036883	2.554677133045	-0.730343865046
C	-3.315355934410	0.469467111511	-0.140989815619
C	-4.719926022053	0.523777884943	-0.332197048217
H	-5.170264183997	1.462615184935	-0.687852134788
C	-5.529568485720	-0.581696956494	-0.084262163024
C	-4.940286479302	-1.790603890401	0.373548165352
H	-5.574433123335	-2.668229096123	0.571663170775
C	-3.570481970471	-1.873267660589	0.578378591267
H	-3.103919522439	-2.803111734162	0.934523873254
C	-2.712271769030	-0.757016677847	0.330632674022
C	2.678692531623	-0.842883887806	0.013201110254
C	3.508030910540	-1.982704770548	-0.235336557510
H	3.010888625279	-2.957574686977	-0.344752673176
C	4.888708925018	-1.868857192962	-0.318257750002
H	5.499570565722	-2.766780440214	-0.498799230078
C	5.521377194516	-0.604585208013	-0.174692445758
C	4.743358603045	0.528930076728	0.049311103137
H	5.225423876914	1.513049577247	0.148206450152
C	3.331208937387	0.442019460634	0.148129515871
C	2.574509695817	1.653019508927	0.316757335104
H	3.145608080039	2.602685934449	0.303946214344
C	0.615813987733	3.044029137116	0.450829397963
H	1.329194068426	3.855293192978	0.197563473326
H	0.201839946301	3.231548542769	1.463804148144
C	-0.526560236940	2.967133098010	-0.567131249039
H	-1.191680844129	3.854126176197	-0.499564735114
H	-0.099553177643	2.931675086159	-1.593447998248
H	-0.119650106542	0.578954441099	2.050691026366
C	7.018741358495	-0.521437815200	-0.262623647982
F	7.624476678508	-1.225474103828	0.734575922761
F	7.486612298811	-1.045576479425	-1.429396310153
F	7.477031968721	0.751555163394	-0.184067375215
C	-7.016395996963	-0.526462235202	-0.294846130848
F	-7.427852514836	-1.427867906332	-1.229740167020
F	-7.699457017742	-0.828028221946	0.844157263435
F	-7.441462907254	0.693334311381	-0.704146857546

^{2}[Fe] 2b

E_{tot} (PBE0-D3(Acetonitrile)/def2TZVP // PBE-D3(Acetonitrile)/def2SVP/W06) = -2815.3409728100

$\langle S^2 \rangle = 0.8001$

Fe	-0.003514709124	0.512073722413	0.014150476104
O	-1.266558101873	-0.864093811365	-0.165629861852
O	1.266306107851	-0.869647499813	0.002498219527
N	1.265286891691	1.878606856679	0.112645360400
N	-1.266114199188	1.876293488268	-0.181835514717
C	-2.576374411652	1.749023866949	-0.182239902081
H	-3.188598559651	2.666556296293	-0.282753725989
C	-3.273569331172	0.499577076462	-0.093990260127
C	-4.693315706681	0.505488887693	-0.042252223490
H	-5.220772826290	1.471057657659	-0.035136155520
C	-5.419419033804	-0.680671354376	-0.003119338940
C	-4.732045663196	-1.926192204678	-0.029011247987
H	-5.303869639180	-2.866378624852	-0.003324066148
C	-3.347506911147	-1.962825834339	-0.088210267663
H	-2.804143817516	-2.918908370797	-0.109657273308
C	-2.565638529891	-0.763261124523	-0.111211314785
C	2.565745426622	-0.764651342123	0.002608131288
C	3.349990178171	-1.961964521233	-0.059950874525
H	2.806974075936	-2.917452688738	-0.104507490829
C	4.735371323797	-1.924180920420	-0.060782138541
H	5.308309370522	-2.862935740659	-0.107151621508
C	5.422491263276	-0.679287508167	0.000869022659
C	4.694347702136	0.504200820923	0.060566474870
H	5.220768874729	1.469196332216	0.108163282133
C	3.273165454182	0.497610743298	0.060922525296
C	2.575685734304	1.747364866081	0.127940205778
H	3.188838512327	2.666499100921	0.204996849301
C	0.671108847691	3.207750317484	0.315169390719
H	1.336349199566	4.014555116505	-0.054882543949
H	0.518647770101	3.359314245817	1.405490620938
C	-0.668851503144	3.201906581667	-0.404793445051
H	-1.334878527055	4.016278546974	-0.052761419298
H	-0.513585781694	3.334377356050	-1.497441111875
H	-0.174340125510	0.599878988168	1.479981830594
C	-6.919632199228	-0.676503134253	0.077538090195
F	-7.445453872497	0.569655532340	-0.008332846137
F	-7.480844151567	-1.418904483851	-0.916740263015
F	-7.365423316126	-1.216624841315	1.246805367642
C	6.924683508599	-0.674863878056	0.004100999918
F	7.445241612598	0.575551514614	0.049845174608
F	7.430186793706	-1.357384622999	1.069593740701
F	7.434301169711	-1.280232538103	-1.105000984870

^{5}[Fe] 14b

E_{tot} (PBE0-D3(Acetonitrile)/def2TZVP // PBE-D3(Acetonitrile)/def2SVP/W06) = -2814.7980997000

$\langle S^2 \rangle$ = *6.0318*

Fe	0.000000663223	0.055113104242	-0.000003369434
O	1.557176955038	-1.104581223869	-0.064500275722
O	-1.557176708876	-1.104582575779	0.064492741010
N	-1.309864770678	1.655962651373	-0.277756919618
N	1.309863058801	1.655959895600	0.277761106024
C	2.616445930696	1.620674473449	0.288194632282
H	3.176910688888	2.571548587199	0.426320632065
C	3.419909731783	0.432277570240	0.125372245890
C	4.831370848720	0.575874018191	0.135098267031
H	5.259543500382	1.580810452324	0.270601065897
C	5.679296277587	-0.518126128475	-0.024251259858
C	5.119737786219	-1.812440138000	-0.200323252869
H	5.782635958315	-2.682338015110	-0.326908972751
C	3.743322110928	-1.986577457679	-0.213835634490
H	3.299742342654	-2.984272798298	-0.348833526100
C	2.839696447277	-0.885920910382	-0.052451733989
C	-2.839695607231	-0.885921180920	0.052449585973
C	-3.743321808597	-1.986576806827	0.213839000398
H	-3.299741956227	-2.984271912031	0.348838858810
C	-5.119737400349	-1.812439660007	0.200328241073
H	-5.782634927547	-2.682337428432	0.326917897261
C	-5.679296417495	-0.518126431896	0.024252350376
C	-4.831370862824	0.575873025596	-0.135101549429
H	-5.259543807032	1.580808985885	-0.270607026453
C	-3.419909920804	0.432276760828	-0.125376282758
C	-2.616446993546	1.620674352499	-0.288199647371
H	-3.176913659401	2.571546556939	-0.426332742575
C	-0.615571100186	2.925002350692	-0.456056114387
H	-1.270370687104	3.798208043027	-0.242536251289
H	-0.280162920916	3.002609551452	-1.514395993813
C	0.615570453118	2.924999785843	0.456062729852
H	1.270371593263	3.798205423395	0.242547804027
H	0.280159664442	3.002603336800	1.514402091547
C	7.171655387218	-0.361782665783	-0.010849593218
F	7.563432313377	0.927957267923	0.136207431670
F	7.749271728376	-1.070614276619	1.000959267233
F	7.741611879153	-0.823118087006	-1.160365806032
C	-7.171655304157	-0.361782847068	0.010852063195
F	-7.563432320276	0.927956781822	-0.136206767590
F	-7.749273132104	-1.070616289860	-1.000954921414
F	-7.741610509618	-0.823116239249	1.160369735613

^{3}[Fe] 14b

E_{tot} (PBE0-D3(Acetonitrile)/def2TZVP // PBE-D3(Acetonitrile)/def2SVP/W06) = -2814.7887823100

$\langle S^2 \rangle$ = 2.0346

Fe	-0.000001304166	0.476100176149	0.000007096404
O	1.296252559234	-0.909569184509	0.043389168295
O	-1.296251683985	-0.909569109205	-0.043356973962
N	-1.270168068248	1.859554610221	-0.162116611273
N	1.270168966159	1.859554065652	0.162123458773
C	2.580177960981	1.732762738744	0.176597018299
H	3.190718066950	2.652182990181	0.275015879746
C	3.286905503019	0.487680412792	0.085164723019
C	4.707810409660	0.511337436113	0.068885970251
H	5.221911411911	1.483169980763	0.115474054002
C	5.452920889115	-0.661308535211	-0.004072667505
C	4.780790760926	-1.914436962486	-0.059196014899
H	5.364693158564	-2.846031260967	-0.114951542573
C	3.395941794950	-1.969426866333	-0.041512297945
H	2.865274986779	-2.932185726682	-0.082371795767
C	2.592404664951	-0.784107700964	0.028946424429
C	-2.592405039728	-0.784106423344	-0.028924583683
C	-3.395941858913	-1.969425144129	0.041537370282
H	-2.865274229312	-2.932183150883	0.082407994419
C	-4.780791081326	-1.914435882523	0.059210857374
H	-5.364692801623	-2.846030340203	0.114969890142
C	-5.452920772101	-0.661307751078	0.004073977502
C	-4.707809432869	0.511337722522	-0.068886735013
H	-5.221910671293	1.483169723867	-0.115484192630
C	-3.286904523669	0.487680778075	-0.085155854621
C	-2.580176532371	1.732763287066	-0.176590094979
H	-3.190717632234	2.652182747173	-0.275011263746
C	-0.664156529891	3.182542728279	-0.371983655163
H	-1.330944278273	4.001128879391	-0.029541534996
H	-0.487467597851	3.3180085514394	-1.461481137379
C	0.664159222593	3.182543789708	0.371985648731
H	1.330946814729	4.001127795884	0.029538191218
H	0.487471276724	3.318092662229	1.461482545813
C	-6.953417034597	-0.635645451570	0.029587784586
F	-7.491176567593	-1.355968905892	-0.995178250042
F	-7.451723630224	-1.185712633515	1.173417737638
F	-7.458957635887	0.619193028744	-0.058895894245
C	6.953416651058	-0.635645718971	-0.029610779743
F	7.491192765201	-1.356021465571	0.995109352785
F	7.451704404843	-1.185654358543	-1.173477865980
F	7.458958642011	0.619188388493	0.058927183459

[1][Fe] 14b

E_{tot} (PBE0-D3(Acetonitrile)/def2TZVP // PBE-D3(Acetonitrile)/def2SVP/W06) = -2814.7264078800

$\langle S^2 \rangle = 0$

Fe	-0.000000346814	0.440691181946	-0.000007636850
O	-1.296316838223	-0.795528091887	0.402091475463
O	1.296319684498	-0.795524686849	-0.402109125147
N	1.208658638837	1.766995962522	0.360497394353
N	-1.208659074954	1.766996198657	-0.360506604093
C	-2.531920678132	1.681998748111	-0.423692090466
H	-3.102691668820	2.599164454789	-0.660635860782
C	-3.270060759356	0.487104404596	-0.156040929205
C	-4.688011753398	0.488721265771	-0.257010193114
H	-5.197766801638	1.403810419607	-0.592789140857
C	-5.432408149968	-0.642371415063	0.064065216254
C	-4.778042896233	-1.820770654577	0.518380002553
H	-5.369081565754	-2.713056496403	0.773844357431
C	-3.395719253588	-1.845573885541	0.642687047384
H	-2.870509926655	-2.745421426572	0.994906313309
C	-2.600878729078	-0.713217484091	0.293353772129
C	2.600880909288	-0.713211821617	-0.293370297669
C	3.395724002691	-1.845562493220	-0.642717609937
H	2.870516265384	-2.745404983927	-0.994952141550
C	4.778047102968	-1.820760276878	-0.518405354320
H	5.369087517182	-2.713041066128	-0.773883364694
C	5.432409553146	-0.642367012547	-0.064070581473
C	4.688011353014	0.488721155420	0.257015868492
H	5.197764683626	1.403806638909	0.592807744292
C	3.270060982768	0.487105568251	0.156039509238
C	2.531919386036	1.681997738421	0.423696415348
H	3.102688013449	2.599161426093	0.660653314287
C	0.572327464495	3.086490274046	0.504523701177
H	1.293205676024	3.910605832452	0.325017375184
H	0.170669758803	3.189355726167	1.535345833884
C	-0.572328338058	3.086491113279	-0.504532463270
H	-1.293206528294	3.910606147572	-0.325024297131
H	-0.170672302426	3.189356278579	-1.535355109845
C	-6.932315605846	-0.645855168457	-0.053256922443
F	-7.427850552385	0.540020894887	-0.481204532613
F	-7.365473157211	-1.598806855214	-0.923951002586
F	-7.529108028127	-0.923149552608	1.138624761567
C	6.932314697644	-0.645856794258	0.053276563402
F	7.427851970597	0.540045813028	0.481147722076
F	7.365448369921	-1.598748526793	0.924049057502
F	7.529123669266	-0.923243567737	-1.138574975310

[6][Fe] 2c

E_{tot} (PBE0-D3(Acetonitrile)/def2TZVP // PBE-D3(Acetonitrile)/def2SVP/W06) = -2370.4588729200

$\langle S^2 \rangle$ = *8.7626*

Fe	-0.013922943366	0.225166792980	0.718611331368
O	-1.487377604573	-0.977976046812	0.401307689590
O	1.448682952601	-0.954399330671	0.316978761840
N	1.358413414941	1.815955444396	0.316555413444
N	-1.252352979989	1.724804360484	-0.211558456685
C	-2.539938949555	1.638296298831	-0.389312610433
H	-3.087231736957	2.538375643995	-0.747287270859
C	-3.347637210177	0.456686570075	-0.189117915498
C	-4.743016747044	0.561523807141	-0.412474519448
H	-5.180967986836	1.530997927155	-0.696619178341
C	-5.593476039699	-0.544785937219	-0.286230082417
C	-5.029753579029	-1.799274721453	0.063034123673
H	-5.665155058774	-2.689914880179	0.166215694744
C	-3.655553056136	-1.924030596179	0.280124513120
H	-3.222540653229	-2.898708765482	0.550107932750
C	-2.768238508231	-0.816469588116	0.175561202382
C	2.738548563708	-0.802883681324	0.126583368323
C	3.573092204667	-1.949740144133	0.013683612314
H	3.093297199107	-2.936580015793	0.093433914900
C	4.952042469798	-1.851205403608	-0.185961501095
H	5.543417411196	-2.774444001342	-0.260539288282
C	5.575677994780	-0.581165246808	-0.291930507636
C	4.779294985488	0.567274720965	-0.194083699787
H	5.263654475823	1.552187355767	-0.282045688452
C	3.380710975586	0.487828934239	0.016465586332
C	2.634826964823	1.724700014690	0.089612179891
H	3.219271914582	2.658568034903	-0.067260594036
C	0.681085421024	3.101455778314	0.323090826922
H	1.332162188747	3.931331692097	-0.027453533849
H	0.365257212965	3.325901663098	1.366232985571
C	-0.562069781581	2.964218423915	-0.561899823710
H	-1.223069416017	3.852908765584	-0.469303119043
H	-0.237794124043	2.893109741619	-1.624108783160
H	-0.074769267562	0.419830641353	2.400259687524
O	-6.920768405159	-0.327652741588	-0.518790023278
C	-7.815724218606	-1.424884951783	-0.409093018086
H	-7.574499732368	-2.233023728961	-1.135291001378
H	-7.821049957999	-1.856521030800	0.616696217661
H	-8.823199762351	-1.029905759537	-0.635690643488
O	6.912490851036	-0.386066709980	-0.486996779700
C	7.755287705358	-1.524650902845	-0.587315286078
H	7.732675333644	-2.141968293474	0.338464153064
H	7.483335888973	-2.169091730622	-1.453026342693
H	8.781602031671	-1.141310126832	-0.736074335742

4[Fe] 2c

E_{tot} (PBE0-D3(Acetonitrile)/def2TZVP // PBE-D3(Acetonitrile)/def2SVP/W06) = -2370.4475713500

$\langle S^2 \rangle$ = *3.8137*

Fe	0.030459166423	0.555254696639	0.684015203136
O	-1.378016029606	-0.657103671224	1.023199143553
O	1.146678023340	-0.750986879918	-0.234609601054
N	1.325998890499	1.919328742535	0.410209410902
N	-1.153506458153	1.774163913984	-0.363037939283
C	-2.418650523412	1.592775546952	-0.627502174197
H	-2.969727561592	2.387754012187	-1.172842468259
C	-3.176431139280	0.428350958137	-0.244960280100
C	-4.532197715583	0.354979916077	-0.660006982208
H	-4.959156115849	1.165484911898	-1.270896618144
C	-5.351494546303	-0.725713497731	-0.317629373361
C	-4.803792607118	-1.766186828026	0.478906542303
H	-5.415563903031	-2.631355836343	0.770443661394
C	-3.478732221962	-1.704194756875	0.910176236350
H	-3.059570547394	-2.508926256671	1.532550390607
C	-2.609976854322	-0.625938324028	0.570152092588
C	2.459663281288	-0.733350559414	-0.190047569845
C	3.190185060396	-1.933938342112	-0.423562461267
H	2.609911691442	-2.851404630873	-0.603353714917
C	4.585794254873	-1.973837961351	-0.422597799798
H	5.088659711038	-2.935179741808	-0.598008033869
C	5.339854294703	-0.790110037887	-0.207240070713
C	4.656258518910	0.414131640374	-0.000017253229
H	5.243922476427	1.333362203817	0.147431346336
C	3.240403328555	0.469969702764	0.031235178913
C	2.598111319871	1.756038116710	0.148191106241
H	3.209575744600	2.660508920263	-0.043062546235
C	0.672720407224	3.221568172057	0.265252912765
H	1.382645055165	4.001922044403	-0.078710713359
H	0.257235167039	3.526802258772	1.249256794621
C	-0.469601156560	3.013571123393	-0.740453974373
H	-1.158382867915	3.884122018038	-0.763531987403
H	-0.036581042797	2.887897273983	-1.757377391114
H	0.390886975427	0.543781882534	2.214871901048
O	-6.636978084708	-0.695736416698	-0.778626850829
C	-7.500984819083	-1.771111160529	-0.442950281838
H	-7.130224421452	-2.743165880590	-0.838620176362
H	-7.642423484547	-1.864609061411	0.657131727417
H	-8.477324736502	-1.545142833254	-0.910138303865
O	6.704651599264	-0.731388256903	-0.195303998132
C	7.435788665585	-1.927830321678	-0.417264712578
H	7.225438968714	-2.695538888461	0.360660600105
H	7.223189684159	-2.366751861690	-1.417802821446
H	8.505914500647	-1.653949766101	-0.367953591642

[2][Fe] 2c

E_{tot} (PBE0-D3(Acetonitrile)/def2TZVP // PBE-D3(Acetonitrile)/def2SVP/W06) = -2370.4609718300

$\langle S^2 \rangle$ = *0.8021*

Fe	-0.002719911764	0.292952119643	0.069539126542
O	-1.267461422905	-1.077487005321	-0.089838137440
O	1.267827550003	-1.080237728853	0.035874035495
N	1.268702073671	1.653705184992	0.109858851860
N	-1.265355564007	1.648109069640	-0.153912760053
C	-2.579965826133	1.524679672081	-0.166580674205
H	-3.184595206337	2.444943376139	-0.289855648913
C	-3.284694360860	0.283130250302	-0.065746544790
C	-4.713375599293	0.299342853082	-0.031599069826
H	-5.218287248650	1.275626416826	-0.045641048303
C	-5.441592188407	-0.889824029467	0.016459633757
C	-4.743095436772	-2.132399122047	0.018884323817
H	-5.335237695507	-3.059608567164	0.054180348225
C	-3.357912831480	-2.169873639987	-0.020907491141
H	-2.818570890724	-3.128991442342	-0.018294853074
C	-2.573170754504	-0.973554044207	-0.053399233773
C	2.573730156069	-0.973566463084	0.015341866103
C	3.358348338431	-2.169158115914	-0.046390644812
H	2.818313379474	-3.127590757899	-0.070330968535
C	4.743736314608	-2.132498378041	-0.070792386857
H	5.335323322715	-3.059705912205	-0.114542465505
C	5.443335328417	-0.890911152422	-0.036638192334
C	4.715429574569	0.297644818170	0.020952717678
H	5.220709307418	1.273485903264	0.048295207875
C	3.286054982755	0.282535891600	0.045758436693
C	2.583487166961	1.527631564923	0.102678962797
H	3.190952509323	2.453066571872	0.147161963353
C	0.679669439230	2.986589204021	0.298912834144
H	1.339448575433	3.787255178869	-0.095273935720
H	0.543512929501	3.159932730268	1.388551565469
C	-0.671362709719	2.971246401991	-0.400210346615
H	-1.332005776928	3.790150987067	-0.046674766856
H	-0.532042005174	3.096046009750	-1.496373303195
H	-0.139144847367	0.409800347034	1.540947441873
O	-6.803071845716	-0.981132986025	0.059275940819
O	6.805361721340	-0.982986287071	-0.064099873115
C	-7.552731674961	0.223775654895	0.054926356229
H	-7.375828169605	0.817875431381	-0.869768376419
H	-8.618492214009	-0.067342583089	0.096902074528
H	-7.316299410944	0.861361723578	0.936353892585
C	7.555550207614	0.221106176025	-0.034112844999
H	7.365260844947	0.804355757756	0.894848947198
H	8.621682070743	-0.070216282004	-0.063792595533
H	7.332703882994	0.869467024381	-0.911255349071

^{5}[Fe] 14c

E_{tot} (PBE0-D3(Acetonitrile)/def2TZVP // PBE-D3(Acetonitrile)/def2SVP/W06) = -2369.9124535000

$\langle S^2 \rangle$ = *6.0360*

Fe	0.000002291539	0.087250345419	0.000008690395
O	1.549631444881	-1.063626124982	-0.063141870532
O	-1.549633009637	-1.063625509515	0.063165937837
N	-1.306660749968	1.689146179527	-0.284095110139
N	1.306662757237	1.689151365498	0.284094773885
C	2.615624722604	1.655982207165	0.306785530434
H	3.171592899261	2.608732448715	0.453112900671
C	3.423118196765	0.471086062362	0.146180461728
C	4.835943365595	0.617929018403	0.167811356378
H	5.276932806170	1.617223986214	0.310720549160
C	5.696596974239	-0.472822082987	0.009288129345
C	5.134866475521	-1.763642133692	-0.177898419850
H	5.779981952581	-2.644310264919	-0.305022170585
C	3.749142849884	-1.931707087984	-0.201834428398
H	3.314877357939	-2.932944451999	-0.345449186089
C	2.841419688503	-0.843804537185	-0.042342323576
C	-2.841419628057	-0.843802296396	0.042356350086
C	-3.749145154884	-1.931703884314	0.201849838049
H	-3.314879683659	-2.932940026807	0.345473924206
C	-5.134868257777	-1.763639713453	0.177905013990
H	-5.779984141101	-2.644306885477	0.305032567764
C	-5.696598019755	-0.472820449036	-0.009293249463
C	-4.835943568570	0.617929256606	-0.167816902494
H	-5.276931162412	1.617223864514	-0.310734388634
C	-3.423118103681	0.471087003691	-0.146176712094
C	-2.615623522263	1.655981475284	-0.306782539767
H	-3.171588297599	2.608733572480	-0.453108784186
C	-0.613569623088	2.958419445463	-0.458844025721
H	-1.270107405135	3.831560535554	-0.247207534397
H	-0.272040429514	3.040340352657	-1.515298162244
C	0.613570458215	2.958423063412	0.458838876199
H	1.270105982251	3.831565126054	0.247198607161
H	0.272041829495	3.040348444065	1.515292908330
O	7.040388921615	-0.208949868231	0.046242878503
C	7.943604164540	-1.289994258884	-0.115609907610
H	7.830999308110	-2.054200154352	0.686407855182
H	7.823661717497	-1.792296923554	-1.102140632739
H	8.961116567959	-0.860525560590	-0.056799944238
O	-7.040390212046	-0.208947603318	-0.046253282241
C	-7.943607567306	-1.289996130608	0.115557564672
H	-7.830973686864	-2.054193098262	-0.686464616549
H	-7.823697650784	-1.792309630934	1.102086909661
H	-8.961119456485	-0.860530028141	0.056718621053

^{3}[Fe] 14c

E_{tot} (PBE0-D3(Acetonitrile)/def2TZVP // PBE-D3(Acetonitrile)/def2SVP/W06) = -2369.9034483700

$\langle S^2 \rangle$ = *2.0391*

Fe	-0.000000405805	0.459315531268	0.000009926139
O	-1.293951543928	-0.919008533281	-0.049724818573
O	1.293951633133	-0.919009543390	0.049759736570
N	1.270353784918	1.839980654706	0.159305803980
N	-1.270352316913	1.839980289585	-0.159304465222
C	-2.583803430120	1.719205706660	-0.175386275699
H	-3.188464840401	2.642783390692	-0.273739826308
C	-3.295836218079	0.478195295217	-0.084965083975
C	-4.718375827585	0.505513378049	-0.066032991980
H	-5.245488574928	1.471442474347	-0.109126622864
C	-5.475670709929	-0.665529554779	0.007692528302
C	-4.799852273164	-1.916248263609	0.059485407850
H	-5.364282615495	-2.857559888267	0.116216550261
C	-3.406788135710	-1.965190982270	0.038195926100
H	-2.883890643942	-2.932913202414	0.077476176713
C	-2.598727547935	-0.791123671036	-0.031822045066
C	2.598727078945	-0.791124233400	0.031843600089
C	3.406787406519	-1.965192021129	-0.038175422293
H	2.883888821799	-2.932914143730	-0.077443991614
C	4.799851165461	-1.916249526942	-0.059481757808
H	5.364281091812	-2.857561334696	-0.116213683160
C	5.475670200873	-0.665530410482	-0.007704496935
C	4.718376099341	0.505512794498	0.066023181400
H	5.245489148920	1.471442309670	0.109104213119
C	3.295836619077	0.478194880475	0.084970182911
C	2.583804788283	1.719206099944	0.175386257994
H	3.188466689046	2.642784315867	0.273730926260
C	0.665174076227	3.162715476891	0.370242197521
H	1.330827630358	3.981807782397	0.025117099606
H	0.490419033897	3.302362793051	1.459934279848
C	-0.665171764750	3.162712967884	-0.370250382869
H	-1.330824739689	3.981808370652	-0.025131156229
H	-0.490416721802	3.302352778515	-1.459943436240
O	-6.837486231474	-0.521058394621	0.023877599691
C	-7.639499531525	-1.688144678369	0.101240317367
H	-7.484699427393	-2.361979603979	-0.771504073659
H	-7.449221505722	-2.266462997857	1.033475598961
H	-8.691303088244	-1.346233942005	0.103792993041
O	6.837485741229	-0.521059102868	-0.023907279325
C	7.639498434841	-1.688144688028	-0.101284900392
H	7.484714147537	-2.361980892533	0.771461332032
H	7.449204714470	-2.266462108908	-1.033517581260
H	8.691301778382	-1.346233347973	-0.103855611424

[1][Fe] 14c

E_{tot} (PBE0-D3(Acetonitrile)/def2TZVP // PBE-D3(Acetonitrile)/def2SVP/W06) = -2369.8488537800

$\langle S^2 \rangle = 0$

Fe	0.000001338160	0.260961062878	-0.000039461824
O	1.282276510505	-1.020490441791	-0.300013250557
O	-1.282267278486	-1.020498232943	0.299926693338
N	-1.227004554044	1.584087760276	-0.290878477770
N	1.226994551309	1.584086340482	0.290845987424
C	2.555371900072	1.493274630666	0.332537744978
H	3.133063570072	2.416957159864	0.526294056242
C	3.283867699520	0.285842555786	0.112081256079
C	4.710360049163	0.294209744122	0.174419011052
H	5.211610892856	1.237259817536	0.433764614057
C	5.443727206415	-0.863347309945	-0.095931176997
C	4.759949427915	-2.064569684724	-0.452096671810
H	5.361390175574	-2.961346586861	-0.663612640464
C	3.376425722973	-2.090845100336	-0.528855389596
H	2.840900981241	-3.011798510767	-0.804153445453
C	2.590623681071	-0.934866407979	-0.233847261155
C	-2.590616941719	-0.934872662870	0.233805446547
C	-3.376408937970	-2.090856466939	0.528820146623
H	-2.840874410566	-3.011813497739	0.804087046000
C	-4.759935167904	-2.064580850655	0.452104088776
H	-5.361369518175	-2.961361090508	0.663624189768
C	-5.443724253304	-0.863353300375	0.095978372634
C	-4.710366081706	0.294208810802	-0.174375312938
H	-5.211625046067	1.237263085144	-0.433690019114
C	-3.283872150013	0.285841449178	-0.112081097133
C	-2.555382682006	1.493278374212	-0.332536615833
H	-3.133078320449	2.416965232175	-0.526260524428
C	-0.610844800842	2.910260444607	-0.456202430490
H	-1.320934425970	3.726720065298	-0.207145700399
H	-0.294571552019	3.037458597407	-1.514308084881
C	0.610831671025	2.910256277190	0.456186999152
H	1.320920664962	3.726718761999	0.207138669437
H	0.294560283097	3.037441221312	1.514294854211
O	6.801909970080	-0.957348207482	-0.057504582099
O	-6.801908147757	-0.957354101004	0.057590928673
C	7.548021296580	0.201988361566	0.285057859205
H	7.295947927130	0.567278963392	1.305415795689
H	8.613535586265	-0.091163235547	0.259386568202
H	7.381239252543	1.028740796515	-0.440815943756
C	-7.548029812815	0.201988002540	-0.284930588205
H	-7.295986667689	0.567295261491	-1.305289984119
H	-8.613543268657	-0.091164252499	-0.259232415195
H	-7.381226290628	1.028728536366	0.440951803871

5.1.1. Olefin Substrates for Hydroamination

E-1-2-Di-p-tolylethene

E_{tot} (RPBE0-D3(Acetonitrile)/def2TZVP // PBE-D3(Acetonitrile)/def2SVP/W06) =-618.8190117920 *E*(LUM0) = -0.06280

C	2.807096015892	-1.370895916342	-0.000001484434
C	1.936050666007	-0.253341549659	-0.000001839354
C	4.200123177821	-1.214185951465	-0.000002732014
H	4.845489260965	-2.107314318461	-0.000003367155
C	2.535714261296	1.034371321778	-0.000003925341
C	4.791275132784	0.065508605893	-0.000004138596
H	1.903307601342	1.935275967759	-0.000006119501
C	3.924406568441	1.184870486453	-0.000005274487
H	4.355693157023	2.199145086446	-0.000008397191
H	2.375402719674	-2.384829084838	-0.000001432873
C	-3.924405978593	-1.184870711506	0.000005343151
C	-4.791274990876	-0.065509257071	0.000004533346
C	-2.535713685857	-1.034370883223	0.000003328731
H	-1.903306791709	-1.935275302611	0.000005202505
C	-4.200123535804	1.214185670059	0.000004476743
C	-1.936050776385	0.253342114017	0.000001383121
H	-4.845490240086	2.107313592130	0.000006879067
C	-2.807096544098	1.370896170228	0.000002590243
H	-2.375403285922	2.384829306767	0.000003614774
H	-4.355692042295	-2.199145552461	0.000008786194
C	0.488424428704	-0.475505384895	-0.000001391954
H	0.190884586996	-1.538719322122	-0.000002733775
C	-0.488424836159	0.475506618491	0.000000907992
H	-0.190885098811	1.538720456683	0.000002426960
C	-6.287610024019	-0.249541183720	-0.000007454687
H	-6.622460363915	-0.823757876075	-0.889577648626
H	-6.622443273192	-0.824047360656	0.889380335748
H	-6.821044428750	0.720553683466	0.000149913335
C	6.287610114077	0.249540179262	0.000006207146
H	6.622459086452	0.823780644471	0.889561378848
H	6.821044336535	-0.720554765525	-0.000125626697
H	6.622444816306	0.824022875236	-0.889396389237

trans-4-methoxystilbene

E_{tot} (RPBE0-D3(Acetonitrile)/def2TZVP // PBE-D3(Acetonitrile)/def2SVP/W06) =-654.7005554620 *E*(LUM0) = -0.06174

C	-3.818904270756	-1.267369003054	-0.000015814937
C	-2.902709087202	-0.183394313511	0.000001853363
C	-5.204543626229	-1.053127352722	-0.000019430295
H	-5.890121351856	-1.914747261387	-0.000033556626
C	-3.440922045005	1.131097232895	0.000018846163
C	-5.716600905880	0.255220598001	-0.000004495803
H	-2.766298536194	2.000669638220	0.000038809409
H	-6.803674751804	0.427950446878	-0.000006821898
C	-4.824354915280	1.344504840222	0.000015106341
H	-5.214580233441	2.374228398648	0.000029318216
H	-3.425550865119	-2.296753425888	-0.000027838409
C	2.913343030651	-1.362893600222	0.000018226359
C	3.811199644955	-0.266237274467	0.000001728557
C	1.537175109502	-1.152519522534	0.000018439445
O	5.132604692530	-0.577834456247	0.000002465222
H	0.871284946923	-2.028668565051	0.000034104543
C	3.291439291301	1.046155210992	-0.000012181174
C	0.987238853109	0.159776768888	0.000001615703
H	3.956801551482	1.919872816987	-0.000022430439
C	1.902829238791	1.239938972801	-0.000012102377
H	1.511659708546	2.270090281063	-0.000023209008
H	3.332919059417	-2.379985052296	0.000031870756
C	-1.465557569636	-0.470278233603	0.000001014952
H	-1.215541647216	-1.545561444894	-0.000003214762
C	-0.448290469211	0.438144125609	-0.000000674521
H	-0.701614001049	1.512862678079	-0.000007984643
C	6.083535828340	0.480707799006	-0.000010017265
H	5.987024974623	1.120337675801	0.904591342704
H	7.080561309058	0.003707528222	-0.000005480626
H	5.987023651692	1.120314445785	-0.904627318067

***Cis*-Stilbene**

E_{tot} (RPBE0-D3(Acetonitrile)/def2TZVP // PBE-D3(Acetonitrile)/def2SVP/W06) =-540.2449409840 *E*(LUM0) = -0.06011

C	-2.900025645742	0.869475312362	0.560253092047
C	-1.638580776722	0.740900358367	-0.072140721703
C	-3.852238269931	-0.158662604990	0.495528245334
H	-4.820739529654	-0.039191385737	1.005411080923
C	-1.384118923578	-0.441442376069	-0.811948720690
C	-3.574708991719	-1.332660331620	-0.224580962156
H	-0.428646603891	-0.549062541310	-1.345833918498
H	-4.322504607506	-2.138396941382	-0.282469018825
C	-2.339362785227	-1.463474782628	-0.885402975723
H	-2.121536794637	-2.370051366358	-1.471150553763
H	-3.128126586196	1.791904654691	1.117912475517
H	1.158312833318	2.855918574574	-0.053044912784
C	-0.681499115788	1.860681989831	0.002137509191
H	-1.158311319675	2.855920183094	0.053035961295
C	0.681500080814	1.860681181250	-0.002141844380
C	1.638578462117	0.740898223566	0.072141909262
C	1.384117357890	-0.441445642696	0.811945999835
C	2.900027754611	0.869477085372	-0.560245627543
H	0.428645484998	-0.549070772142	1.345830942101
H	3.128129409153	1.791910356320	-1.117898154516
C	2.339361496812	-1.463479362167	0.885397156737
C	3.852240074303	-0.158659891228	-0.495522906899
H	2.121532469777	-2.370057106677	1.471141843073
H	4.820743742553	-0.039186734795	-1.005400571807
C	3.574708673871	-1.332662272588	0.224579797984
H	4.322505151495	-2.138398240854	0.282465119512

4-4-vinylenedipyridine

E_{tot} (RPBE0-D3(Acetonitrile)/def2TZVP // PBE-D3(Acetonitrile)/def2SVP/W06) =-572.3175019940 *E*(LUM0) = -0.08917

C	2.850638460843	-1.274468730697	-0.000001946527
C	1.937006404357	-0.193239009178	0.000000473968
C	4.229335735509	-1.018533989406	-0.000002238855
H	4.941667719260	-1.863297233562	-0.000004273268
C	2.501715994797	1.106310045920	0.000002700353
N	4.761359522904	0.216905897955	-0.000000303149
H	1.868371859178	2.005479552968	0.000005168917
C	3.894116422868	1.249321433216	0.000002031721
H	4.336129794426	2.261947992598	0.000003976273
H	2.482555361222	-2.312232232366	-0.000003631139
C	-3.894116418338	-1.249321436046	0.000002007150
N	-4.761359522620	-0.216905900515	-0.000000212279
C	-2.501715993654	-1.106310044251	0.000002190798
H	-1.868371850820	-2.005479546461	0.000004280898
C	-4.229335738953	1.018533984397	-0.000002298658
C	-1.937006405184	0.193239014193	-0.000000223928
H	-4.941667721226	1.863297230233	-0.000004243908
C	-2.850638461787	1.274468731443	-0.000002519474
H	-2.482555372126	2.312232236636	-0.000004437457
H	-4.336129792274	-2.261947994020	0.000004017424
C	0.497329931559	-0.464573429542	0.000000659146
H	0.231719726446	-1.535175641533	0.000001347326
C	-0.497329932093	0.464573431652	-0.000000334615
H	-0.231719725595	1.535175643227	-0.000001603559

6. Single-crystal X-ray diffraction

Single-crystal X-ray diffraction data for **1b** and **1c** were collected on a Bruker D8-QUEST diffractometer, equipped with an Incoatec IμS Cu microsource (λ = 1.5418 Å) and a PHOTON-III detector operating in shutterless mode. Crystals were mounted on a MiTeGen crystal mount using polyfluoroether oil and measurements were carried out under an Oxford Cryosystems open-flow N_2 Cryostream. The control and processing software was Bruker APEX5. Diffraction images were integrated using SAINT in APEX5, and a multi-scan correction was applied using SADABS. The final unit-cell parameters were refined against all reflections. Structures were solved using SHELXT and refined using SHELXL.

Red crystals of **1b**·2MeCN were grown by slow evaporation of a solution of **1b** in MeCN. The X-ray analysis was largely straightforward, except that three out of four CF_3 groups exhibit rotational disorder that appears to be essentially continuous around a ring. Each disordered CF_3 group was modelled using three components, with the site occupancies of the three components restrained tightly to sum to 1.0. To control the geometry, all C–F distances were restrained to a single common value and all F···F distances in the same component were restrained to 1.633 times that value. Anisotropic ADPs were refined for all F atoms, restrained to resemble isotropic behaviour (ISOR in SHELXL). The other CF_3 group in the complex could be modelled satisfactorily as a single component with only moderately elongated displacement ellipsoids.

Crystals of **1c**·toluene were obtained from a toluene solution layered with pentane. Crystals grew as thin, fragile orange needles within a drop of solution on a microscope slide. Diffraction was weak at higher angle, and the data are truncated to 0.90 Å resolution. The precision of the resulting crystal structure is limited accordingly. Two separate crystals were measured, with the stronger data set being reported. When the drop of solution was allowed to evaporate to dryness, yellow plate-like material was deposited: this yielded the crystal structure of the isolated salen ligand. This structure has been deposited as a Private Communication to the CSD.

SHELXT: G. M. Sheldrick, Acta Cryst. Sect. A, 2015, 71, 3–8.
SHELXL: G. M. Sheldrick, Acta Cryst. Sect. C, 2015, 71, 3–8.

Table S4: Summary of crystallographic and refinement data for **1b·2MeCN** and **1c·toluene**

	**1b·2MeCN**	**1c·toluene**
CCDC number	2477562	2477561
Cambridge data number	RW_B1_0037	RW_B1_0045
Chemical formula	$C_{40}H_{30}F_{12}Fe_2N_6O_5$	$C_{43}H_{44}Fe_2N_4O_9$
Formula weight	1014.40	872.52
Temperature / K	180(2)	180(2)
Crystal system	monoclinic	monoclinic
Space group	P 2_1/n	I 2/a
a / Å	10.8304(3)	12.5111(9)
b / Å	34.6420(10)	23.1694(18)
c / Å	11.1519(3)	14.2543(17)
a / °	90	90
b / °	102.1540(13)	94.795(2)
g / °	90	90
Unit-cell volume / $Å^3$	4090.3(2)	4117.5(7)
Z	4	4
Calc. density / g cm^{-3}	1.647	1.408
F(000)	2048	1816
Radiation type	Cu Ka	Cu Ka
Absorption coefficient / mm^{-1}	6.665	6.152
Crystal size / mm^3	0.20 x 0.16 x 0.10	0.20 x 0.01 x 0.01
2-Theta range / °	8.50–133.27	7.30–117.83
Completeness to max 2-theta	0.997	0.999
No. of reflections measured	66783	26598
No. of independent reflections	7218	2948
R(int)	0.0586	0.1202
No. parameters / restraints	760 / 237	285 / 199
Final R1 values (I > 2s(I))	0.0378	0.0594
Final wR(F^2) values (all data)	0.0954	0.1588
Goodness-of-fit on F^2	1.052	1.039
Largest difference peak & hole / e $Å^{-3}$	0.473, –0.333	0.323, –0.299

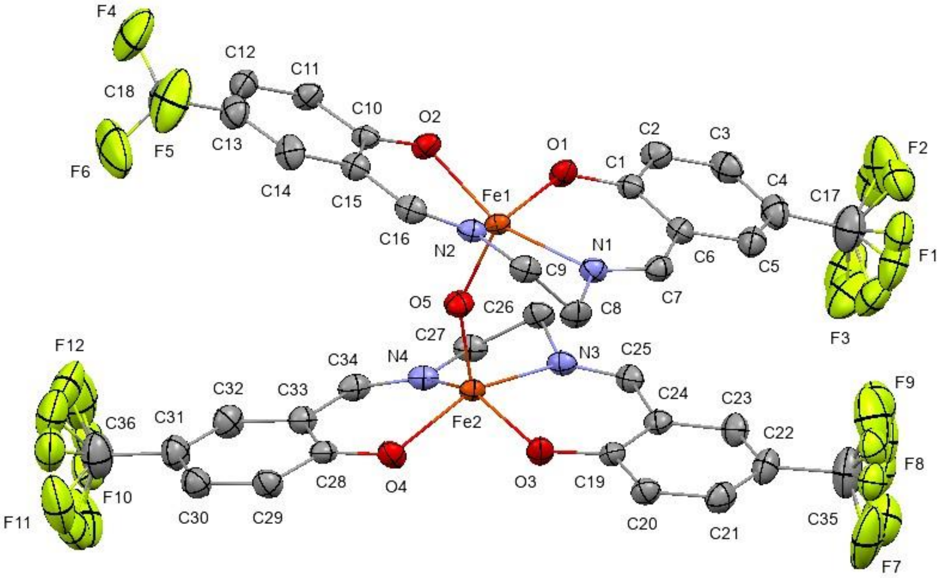

Figure S129. Molecular structure of **1b** in the crystal structure **1b**·2MeCN. Displacement ellipsoids are shown at 50% probability and H atoms are omitted. Three CF_3 groups are modelled with multiple components, representing rotational disorder.

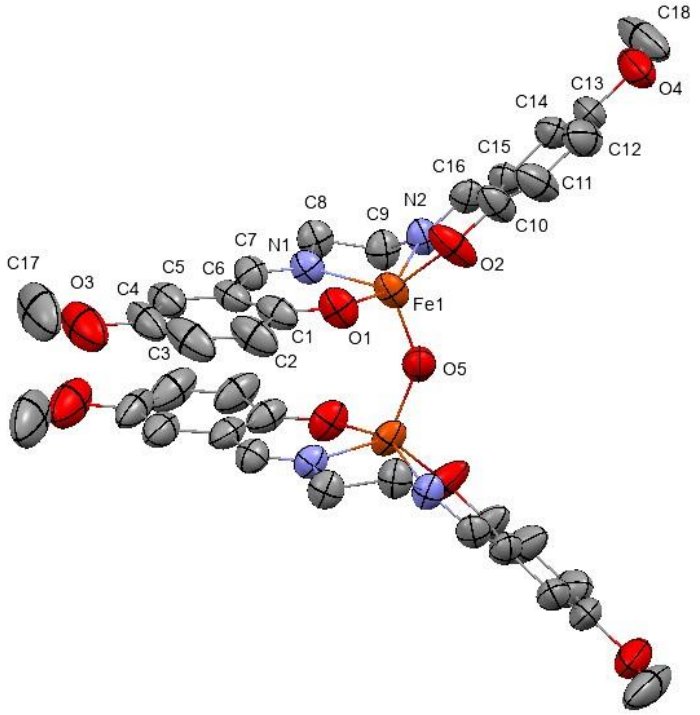

Figure S130. Molecular structure of **1c** in the crystal structure **1c**·toluene. Displacement ellipsoids are shown at 50% probability and H atoms are omitted. The molecule is located a crystallographic 2-fold axis – only the asymmetry unit is labelled.

7. References

1. K. J. Gallagher, M. Espinal-Viguri, M. F. Mahon and R. L. Webster, *Adv. Synth. Catal.*, 2016, **358**, 2460-2468.
2. K. Kang, J. Fuller, A. H. Reath, J. W. Ziller, A. N. Alexandrova and J. Y. Yang, *Chem. Sci.*, 2019, 10, 10135–10142.
3. N. F. Choudhary, N. G. Connelly, P. B. Hitchcock and G. Jeffery Leigh, *J. Chem. Soc., Dalton Trans.*, 1999, 4437–4446.
4. K. Kałduńska CCDC 2384681: Experimental Crystal Structure Determination, 2024, DOI: 10.5517/ccdc.csd.cc2l1g6b
5. M. J. Frisch, G. W. Trucks, H. B. Schlegel, G. E. Scuseria, M. A. Robb, J. R. Cheeseman, G. Scalmani, V. Barone, G. A. Petersson, H. Nakatsuji, X. Li, M. Caricato, A. V. Marenich, J. Bloino, B. G. Janesko, R. Gomperts, B. Mennucci, H. P. Hratchian, J. V. Ortiz, A. F. Izmaylov, J. L. Sonnenberg, Williams, F. Ding, F. Lipparini, F. Egidi, J. Goings, B. Peng, A. Petrone, T. Henderson, D. Ranasinghe, V. G. Zakrzewski, J. Gao, N. Rega, G. Zheng, W. Liang, M. Hada, M. Ehara, K. Toyota, R. Fukuda, J. Hasegawa, M. Ishida, T. Nakajima, Y. Honda, O. Kitao, H. Nakai, T. Vreven, K. Throssell, J. A. Montgomery Jr., J. E. Peralta, F. Ogliaro, M. J. Bearpark, J. J. Heyd, E. N. Brothers, K. N. Kudin, V. N. Staroverov, T. A. Keith, R. Kobayashi, J. Normand, K. Raghavachari, A. P. Rendell, J. C. Burant, S. S. Iyengar, J. Tomasi, M. Cossi, J. M. Millam, M. Klene, C. Adamo, R. Cammi, J. W. Ochterski, R. L. Martin, K. Morokuma, O. Farkas, J. B. Foresman and D. J. Fox, *Journal*, 2016.
6. J. P. Perdew, K. Burke and M. Ernzerhof, *Phys. Rev. Lett.*, 1996, **77**, 3865-3868.
7. S. Grimme, J. Antony, S. Ehrlich and H. Krieg, *J. Chem. Phys.*, 2010, **132**, 154104.
8. J. Tomasi, B. Mennucci and R. Cammi, *Chem. Rev.*, 2005, **105**, 2999-3093.
9. F. Weigend and R. Ahlrichs, *Phys. Chem. Chem. Phys.*, 2005, **7**, 3297-3305.
10. F. Weigend, *Phys. Chem. Chem. Phys.*, 2006, **8**, 1057-1065.
11. J. P. Perdew, M. Ernzerhof and K. Burke, *J. Chem. Phys.*, 1996, **105**, 9982-9985.
12. C. Adamo and V. Barone, *J. Chem. Phys.*, 1999, **110**, 6158-6170.
